# Discovery of Novel Pentacyclic Triterpene Acid Amide Derivatives as Excellent Antimicrobial Agents Dependent on Generation of Reactive Oxygen Species

**DOI:** 10.3390/ijms241310566

**Published:** 2023-06-24

**Authors:** Yihong Yang, Kunlun Chen, Guangdi Wang, Hongwu Liu, Lihui Shao, Xiang Zhou, Liwei Liu, Song Yang

**Affiliations:** National Key Laboratory of Green Pesticide, Key Laboratory of Green Pesticide and Agricultural Bioengineering, Ministry of Education, Center for R&D of Fine Chemicals of Guizhou University, Guiyang 550025, China; yhyang420@163.com (Y.Y.); ifkplp@outlook.com (K.C.); gdwang520@163.com (G.W.); 18798764392@163.com (H.L.); slihuistar@163.com (L.S.); lwliu@gzu.edu.cn (L.L.)

**Keywords:** 18*β*-glycyrrhetinic acid, ursolic acid, antibacterial activities, reactive oxygen species

## Abstract

Developing new agricultural bactericides is a feasible strategy for stopping the increase in the resistance of plant pathogenic bacteria. Some pentacyclic triterpene acid derivatives were elaborately designed and synthesized. In particular, compound A_22_ exhibited the best antimicrobial activity against *Xanthomonas oryzae* pv. *oryzae* (*Xoo*) and *Xanthomonas axonopodis* pv. *citri* (*Xac*) with EC_50_ values of 3.34 and 3.30 mg L^−1^, respectively. The antimicrobial mechanism showed that the compound A_22_ induced excessive production and accumulation of reactive oxygen species (ROS) in *Xoo* cells, leading to a decrease in superoxide dismutase and catalase enzyme activities and an increase in malondialdehyde content. A_22_ also produced increases in *Xoo* cell membrane permeability and eventual cell death. In addition, in vivo experiments showed that A_22_ at 200 mg L^−1^ exhibited protective activity against rice bacterial blight (50.44%) and citrus canker disease (84.37%). Therefore, this study provides a paradigm for the agricultural application of pentacyclic triterpene acid.

## 1. Introduction

Plant diseases caused by the bacterial infestations of plant pathogens are the primary types of crop diseases [1,2]. The literature reports that yield losses due to plant diseases account for 10% to 15% of total yield each year, which causes enormous losses to agricultural production [3,4,5]. In particular, the *Xanthomonas oryzae* pv. *oryzae* (*Xoo*) is one of the main culprits of rice diseases and poses a severe threat to worldwide rice quality and yield [6,7]. Various control measures have been used to respond to pathogenic bacterial attacks. Of note, chemical pesticides are currently the most common and effective method for controlling plant bacterial diseases, providing an essential guarantee for global food production in recent decades [8,9]. However, the use of such traditional chemicals also brings some intractable problems, such as bacterial resistance [10], environmental pollution [11], ecological problems, and human safety concerns [12]. Therefore, an increasing need to develop agricultural bactericides that are both environmentally friendly and highly effective in controlling plant bacterial diseases is present.

Reactive oxygen species (ROS) are vital in bacterial resistance and antibiotic sterilization [13,14]. In recent years, antimicrobial drug employing ROS as a component of their mechanism of action are being developed in the pharmaceutical field and have been successfully applied for the prevention of surgical site infections [15,16], intra-vascular line care [17], and the eradication of multi-resistant organisms [18]. However, few studies on initiators that attack plant pathogens by inducing intracellular ROS have been published. Thus, discovering antimicrobial agents that cause oxidative stress mechanisms in plant bacteria is an appealing method for addressing such bacteria.

**Figure 1:**
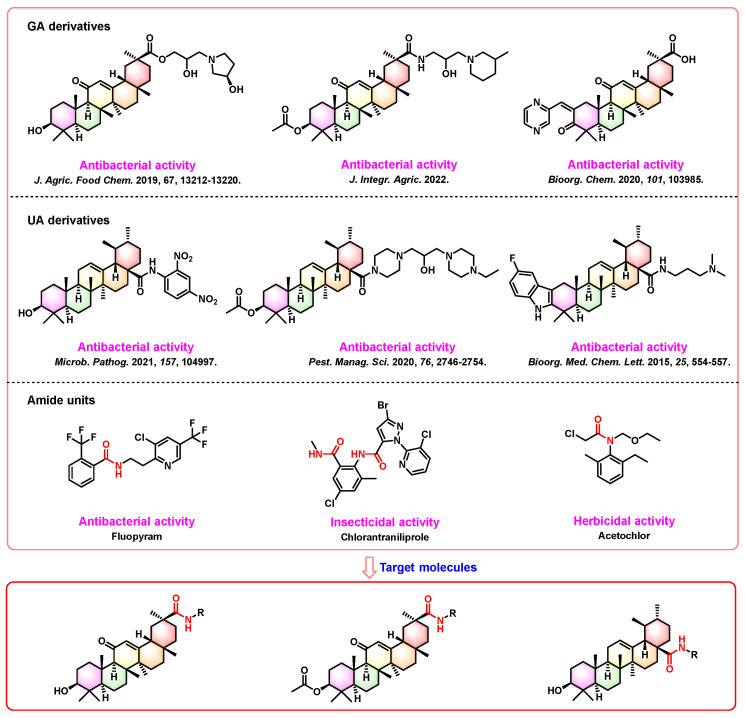
Design conception of the title compounds [26,28,32,33,34,35].

Interestingly, natural products have some excellent advantages, such as biodegradability, extensive sources, and a low susceptibility to drug resistance [19,20,21]. Thus, developing new pesticides based on natural products is a sustainable agricultural disease treatment strategy. Moreover, using natural products as active leads for structural derivation to optimize their molecular structures is gradually becoming essential for developing green, efficient, and novel pesticides. 18*β*-glycyrrhetinic acid (18*β*-GA) and ursolic acid (UA) belong to the pentacyclic triterpenes, which are active ingredients of herbal medicine with various biological properties, such as antioxidant [22], antibacterial [23], anticancer [24], and anti-inflammatory [25]. Our previous work found that the title compounds obtained by the structural derivatization of 18*β*-GA and UA could lead to a reduction in bacterial growth and even death by causing an excessive accumulation of ROS in *Xoo* and *Xanthomonas axonopodis* pv. *citri* (*Xac*) [26,27,28]. In addition, among agrochemicals, the synthesis and biological activity research of compounds containing amide skeletons have been a hot spot in agrochemical research because of their wide range of biological activities, such as antibacterial [29], insecticidal [30], and herbicidal activities [31]. Based on these properties, an active fragment splicing approach was used to introduce amide groups into the 18*β*-GA and UA backbones to obtain a series of title compounds and to investigate whether they could induce bacterial death by causing an inducement of the ROS pathway (Figure 1).

This study further explores the potential of 18*β*-GA with UA for agricultural applications. A series of title molecules containing amide groups were prepared, and their antibacterial activities were evaluated in vitro and in vivo. Finally, a series of biochemical experiments investigated their possible antibacterial mechanism.

## 2. Results and Discussion

### 2.1. Synthesis of Title Compounds

**Scheme 1:**
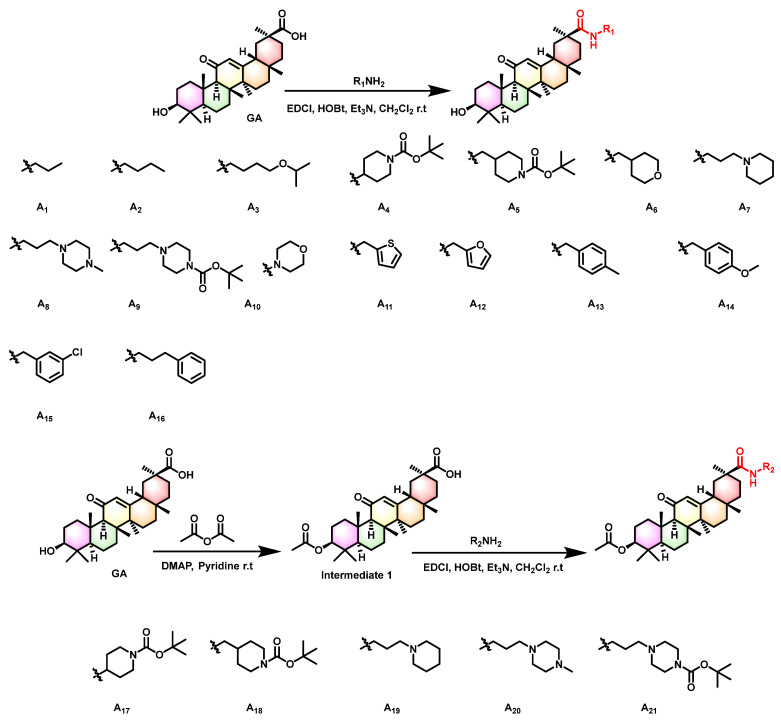
Synthetic route for title compounds A_1_–A_21_.

**Scheme 2:**
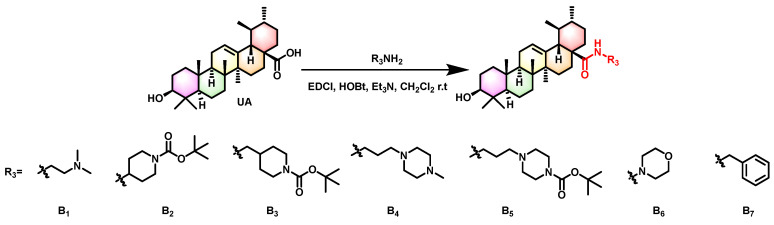
Synthetic route for title compounds B_1_–B_7_.

The previously reported method synthesized a series of novel 18*β*-GA amide derivatives and UA amide derivatives. The target compounds were designed and synthesized as shown in Scheme 1 and Scheme 2. In brief, those compounds were synthesized by acid–amine condensation [36]. Nuclear magnetic resonance (^1^H NMR, ^13^C NMR) and high-resolution mass spectrometry (HRMS) were used to confirm the structures of the title compounds (See Figure S1–S114). (The synthesis method of the title compounds are described in the supporting information).

### 2.2. Results of Anti-Xoo and Anti-Xac of the Title Compounds In Vitro

**Scheme 3:**
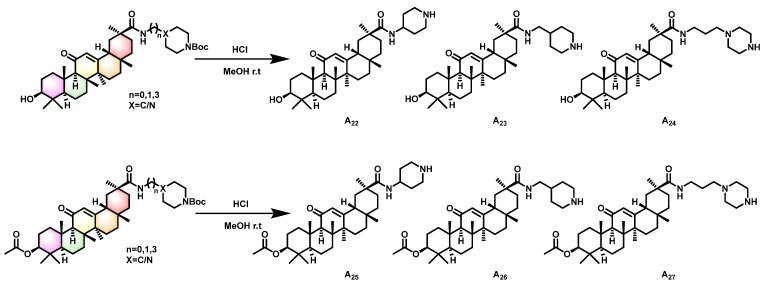
Synthetic route for title compounds A_22_–A_27_.

**Scheme 4:**
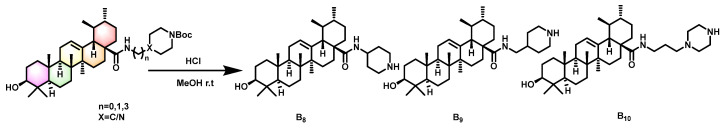
Synthetic route for title compounds B_8_–B_10_.

The preliminary antibacterial activity test results (Table 1) showed that most studied compounds were less effective anti-*Xoo* and anti-*Xac* agents. However, compounds A_7_, A_19_, and A_20_ led to the effective inhibition of the above two phytopathogenic bacteria at 100 and 50 mg L^−1^, respectively. In addition, the literature studies have shown that due to the exposure of the piperazine and piperidine groups, NH favors antimicrobial activity, for example, as seen in the structure of xacin [37]. Therefore, based on the above antimicrobial activity test results and the literature research, compounds in the -Boc groups were considered to have improved antibacterial activities (Scheme 3 and Scheme 4). The results are shown in Table 2, and the antibacterial activities of the compounds obtained after -Boc had all increased and showed excellent activity.

The effective concentrations of 50% maximum antibacterial activity against *Xoo* and *Xac* (EC_50_) were further determined based on the title compounds with initial screening inhibition rates greater than 50%. The results (Table 3) showed that compound A_22_ exhibited optimal antibacterial effects with EC_50_ values of 3.34 and 3.30 mg L^−1^, respectively, which were significantly better than the positive control bismerthiazol (BT, anti-*Xoo*: EC_50_ = 28.34 mg L^−1^, anti-*Xac*: EC_50_ = 101.64 mg L^−1^) and thiodiazole copper (TC, anti-*Xoo*: EC_50_ = 119.87 mg L^−1^, anti-*Xac*: EC_50_ = 86.54 mg L^−1^). This finding suggests that amines containing piperazine and piperidine rings can significantly promote the antibacterial activity of 18*β*-GA and UA after the removal of the -Boc group. Their corresponding structure–activity relationship (SAR) was analyzed for A-series title compounds: (1) the title compounds containing a piperidine ring showed a better inhibitory activity against *Xoo* and *Xac* than those containing piperazine rings; (2) the studied compounds esterified at the C-3 position hydroxyl group of 18*β*-GA showed a reduced inhibitory activity against *Xoo* and *Xac*, suggesting that the C-3 position hydroxyl group may be a crucially active group; and (3) the antibacterial activity of the target compounds with NH exposed on the piperidine and piperazine rings was superior to that of compounds without NH groups, such as compounds A_22_ (anti-*Xoo*: EC_50_ = 3.34 mg L^−1^, anti-*Xac*: EC_50_ = 3.30 mg L^−1^), A_23_ (anti-*Xoo*: EC_50_ = 6.41 mg L^−1^, anti-*Xac*: EC_50_ = 4.03 mg L^−1^), and A_24_ (anti-*Xoo*: EC_50_ = 7.89 mg L^−1^, anti-*Xac*: EC_50_ = 5.18 mg L^−1^), with significantly better antibacterial activity than compound A_7_ (anti-*Xoo*: EC_50_ = 20.84 mg L^−1^, anti-*Xac*: EC_50_ = 4.37 mg L^−1^) and A_8_ (anti-*Xoo*: EC_50_ > 100 mg L^−1^, anti-*Xac*: EC_50_ = 84.62 mg L^−1^), suggesting that NH is an important reactive moiety. For B-series title compounds, several features were observed: (1) UA derivatives with the same structure exhibited antibacterial activity comparable to 18*β*-GA derivatives and (2) the compounds, especially compound B_10_ (anti-*Xoo*: EC_50_ = 4.42 mg L^−1^, anti-*Xac*: EC_50_ = 4.53 mg L^−1^), containing piperazine, piperidine, and morpholine, showed a significant antibacterial activity against *Xoo* and *Xac*, which showed a relatively optimal activity against both plant bacteria.

### 2.3. ROS Fluorescence Imaging

**Figure 2:**
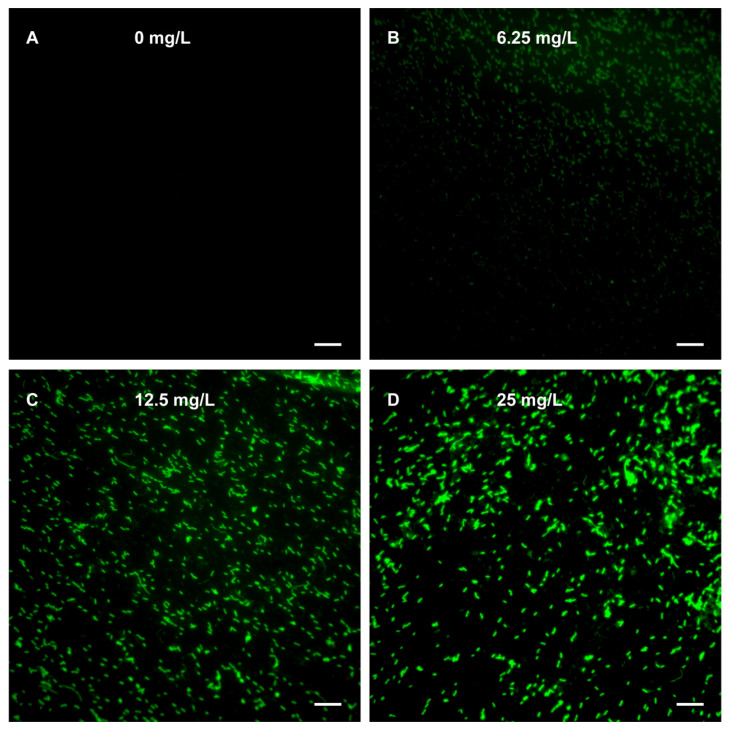
Fluorescence pattern of *Xoo* cells after incubation with compound A_22_ at (**A**) 0 mg L^−1^, (**B**) 6.5 mg L^−1^, (**C**) 12.5 mg L^−1^, and (**D**) 25 mg L^−1^, respectively. Scale bars for (**A**–**D**) are 10 μm.

It has been reported that pentacyclic triterpenoid derivatives often inhibit the growth of cancer cells and are dependent on ROS activity [38,39]. However, their mechanism of action in the field of pesticides has rarely been reported. Therefore, we chose compound A_22_ to investigate whether it could induce intracellular ROS accumulation and cause oxidative damage. We used 2′,7′-dichlorofluorescein diacetate (DCFH-DA) fluorescence analysis to measure the level of ROS production. The results are shown in Figure 2, in which ROS production showed a significant increase in a concentration-dependent manner, and the fluorescence intensity of *Xoo* cells gradually increased with increasing concentrations of compound A_22_ (12.5 and 25 mg L^−1^) compared to the control (0 mg L^−1^). This finding suggests that compound A_22_ can interfere with the balance of the oxidation–reduction system of the *Xoo* cell and induce ROS overproduction, thus causing oxidative stress and cell damage.

### 2.4. CAT and SOD Enzyme Activity Assay Results

**Figure 3:**
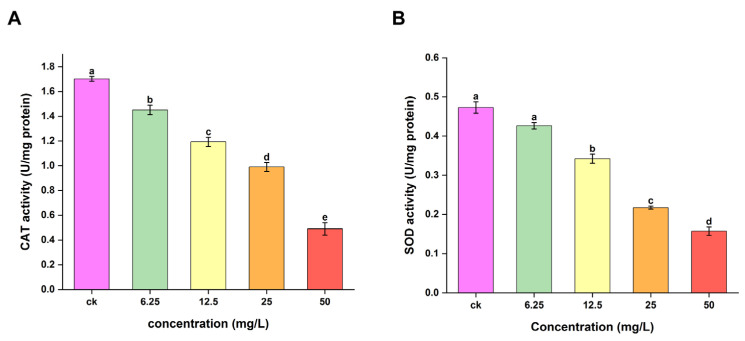
The effect of compound A_22_ on enzyme activities. (**A**) Catalase (CAT); (**B**) superoxide dismutate (SOD) (Different lowercase letters indicate the enzymatic activity with a significant difference among different treatment groups at *p* < 0.05).

Catalase and superoxide dismutase (CAT and SOD, respectively) are the two major antioxidant enzymes in living organisms and are the most direct enzymes for scavenging ROS [40]. As shown in Figure 3, CAT and SOD enzyme activities showed a significant decrease after the action of compound A_22_ (50 mg L^−1^, 25 mg L^−1^) compared to the control (0 mg L^−1^). Therefore, this finding further suggests that compound A_22_ interfered with the oxidoreductase activity and disturbed the redox state in *Xoo* cells, causing a slight amount of oxidative damage.

### 2.5. Lipid Peroxidation

**Figure 4:**
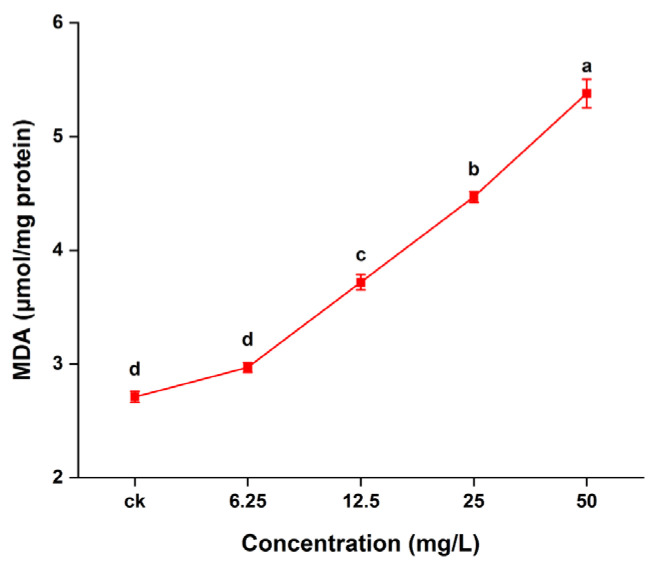
Malondialdehyde production in *Xoo* cells treated with compound A_22_ (Different lowercase letters indicate the enzymatic activity with a significant difference among different treatment groups at *p* < 0.05).

One of the hazards of ROS accumulation is its triggering or exacerbating cell membrane lipid peroxidation, the induction of which plays a crucial role in cell death [41]. Malondialdehyde (MDA) is a highly reactive compound produced during lipid peroxidation under conditions associated with oxidative stress and can reflect the extent of cellular peroxidative damage [42]. As shown in Figure 4, the MDA content of compound A_22_ was 2–3-fold higher at high drug concentrations (25 mg L^−1^ and 50 mg L^−1^) compared to the control (0 mg L^−1^), and MDA production corresponded to ROS levels, indicating that compound A_22_ can induce excessive ROS production, accelerate lipid peroxidation, and eventually lead to bacterial death.

### 2.6. H_2_O_2_ Plate Assay Results

**Figure 5:**
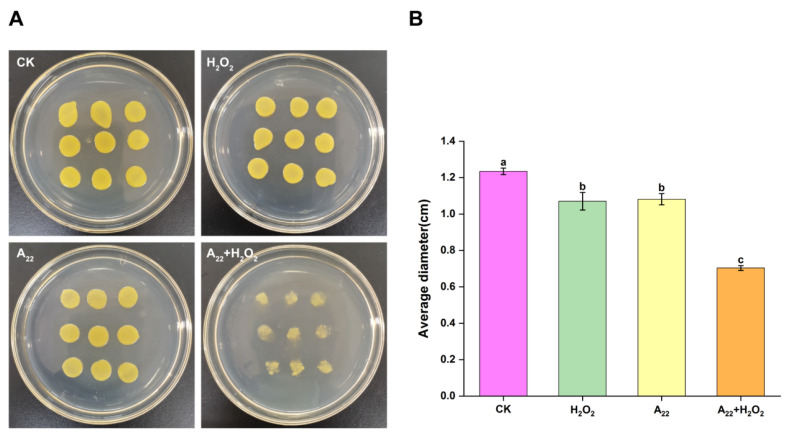
Effect of exogenous hydrogen peroxide (H_2_O_2_) on antibacterial efficiency of compound A_22_. (**A**) Images of plate assay after treatment with 0 mg L^−1^, H_2_O_2_ (0.05 mM), compound A_22_ (12.5 mg L^−1^), and compound A_22_ (12.5 mg L^−1^) + H_2_O_2_ (0.05 mM). (**B**) Average cell diameter after treatment with A_22_ and H_2_O_2_ (Different lowercase letters indicate the enzymatic activity with a significant difference among different treatment groups at *p* < 0.05).

It has been shown that oxidative stress can be triggered intracellularly by adding exogenous hydrogen peroxide (H_2_O_2_) [43,44]. Therefore, we investigated whether adding exogenous H_2_O_2_ would affect the bactericidal efficiency of compound A_22_. Results are shown in Figure 5 in which bacterial growth inhibition was not significant in the Petri dishes containing only H_2_O_2_ or compound A_22_. However, interestingly, in the Petri dishes containing a mixture of H_2_O_2_ and compound A_22_, we observed that the fungus cake shrank and became lighter in color. It is possible that the addition of exogenous H_2_O_2_ caused an acceleration of the burst of oxidative stress induced by compound A_22_ in *Xoo* and accelerated a more rapid bacterial death.

### 2.7. Cell Membrane Damage Detection Results

**Figure 6:**
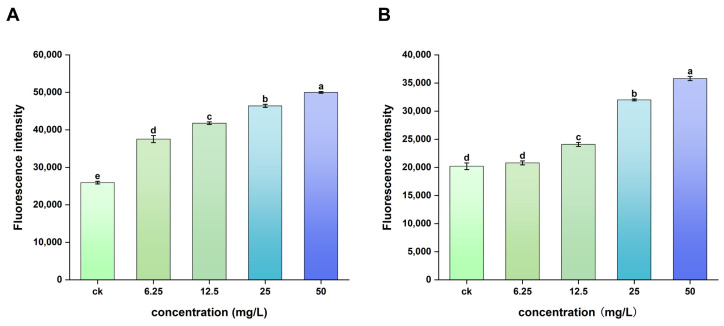
Cell membrane damage of *Xoo* was detected by monitoring compound A_22_ at different concentrations. (**A**) Effects of compound A_22_ on extracellular model of *Xoo* at different concentrations. (**B**) The permeabilization inner membrane of *Xoo* after treatment with increasing concentrations of compound A_22_ (Different lowercase letters indicate the enzymatic activity with a significant difference among different treatment groups at *p* < 0.05).

The generation of intracellular oxidative stress can modify phospholipids and proteins on the membrane through a process involving peroxidation and lead to changes in membrane permeability and the disruption of the phospholipid bilayer, which eventually result in cell death [45]. Among bacteria, the outer membrane of Gram-negative bacteria consists of lipopolysaccharides, phospholipids, outer membrane proteins, and lipoproteins [46]. The outer membrane is the primary physical barrier for bacteria and is closely associated with bacterial pathogenicity and drug resistance [47]. Therefore, the effect of compound A_22_ on the permeability of the *Xoo* outer membrane was determined using the hydrophobic probe, *N*-phenyl-1-naphthylamine (NPN). The results are shown in Figure 6A. The fluorescence intensity was significantly enhanced as the compound concentration increased, which indicated that compound A_22_ effectively destroyed the outer membrane of the *Xoo* cells. Because the propidium iodide (PI) dye does not penetrate live bacterial cell membranes, it then binds to DNA. It emits fluorescence when cell membrane damage occurs, and the increased fluorescence is measured as an indicator of cell membrane permeability [48]. Therefore, to study the inhibitory effect of compound A_22_ on the *Xoo* cell membrane, *Xoo* was incubated with different concentrations of compound A_22_ and fluorescent nucleic acid dye PI. The results are shown in Figure 6B in which the fluorescence intensity gradually increased in a drug concentration-dependent manner, indicating that compound A_22_ could disrupt the permeability of the *Xoo* cell membrane. The above results reveal that compound A_22_ can cause damage to cell membranes and a loss of activity and eventually lead to bacterial death.

### 2.8. In Vivo Anti-Xoo Effect of Compound A_22_

**Figure 7:**
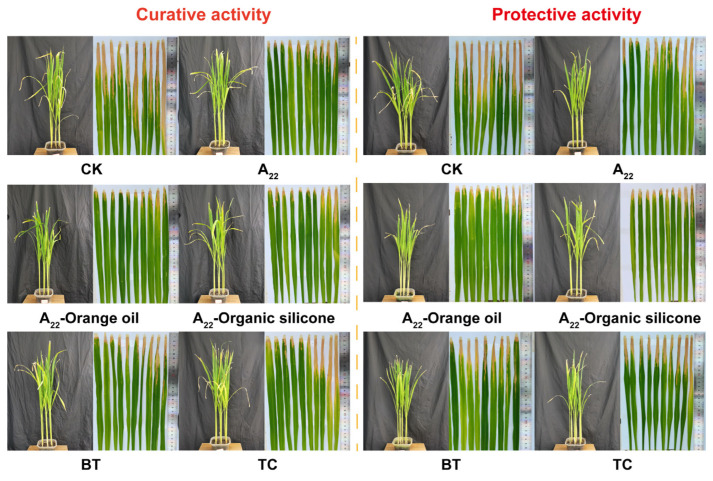
Curative and protective activities of compounds A_22_, A_22_-OPO, A_22_-OSi, BT, and TC anti-*Xoo* agents under greenhouse conditions at 200 mg L^−1^.

The aim of our work was to demonstrate that the title compounds exert excellent antimicrobial efficacy in plants; therefore, we validated them using pot experiments. Table 4 and Figure 7 show that compound A_22_ exhibited excellent in vivo antibacterial activity against the rice bacterial leaf blight. Its therapeutic activity was 44.74%, and its protective activity was 50.44%; both superior to the control BT (curative activity: 39.47%, protective activity: 40.87%) and TC (curative activity: 38.60, protective activity: 37.39%). In particular, the control effect was found to significantly improve after the addition of 0.1% (*v*/*v*) organosilicon (OSi) or orange peel essential oil (OPO) additives. The curative activity was 59.65% (A_22_-OPO) and 60.53% (A_22_-OSi) against the rice bacterial leaf blight. Furthermore, the toxicity results (See Figure S115) of title compound A_22_ were low or even non-toxic toward rice plants.

The Tukey’s HSD test was used to perform one-way ANOVA analysis between the different treatments; different uppercase letters indicate the values of control efficiency with a significant difference among different treatment groups at a level of *p* < 0.05.

### 2.9. In Vivo Anti-Xac Effect of Compound A_22_

**Figure 8:**
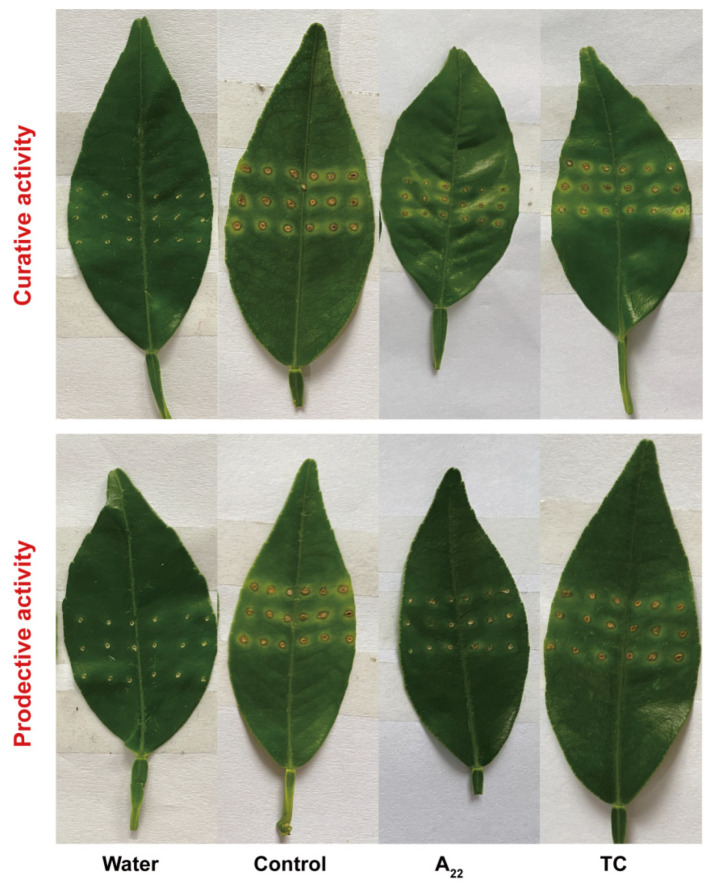
In vivo control efficiency (14 days after spraying) of A_22_ against citrus bacterial canker.

As shown in Table 5 and Figure 8, compound A_22_ exhibited excellent in vivo antibacterial activity against *Xac*. Its curative activity was 58.86%, and its protective activity was 84.37%, which were superior to the control drug TC (curative activity: 51.51%, protective activity: 74.44%). In summary, the compound A_22_ exhibited excellent antibacterial activity in vitro and a good control effect in vivo.

## 3. Materials and Methods

### 3.1. Instruments and Chemical Substances

The JEOL-ECX-500 spectrometer (500 MHz, JEOL Ltd., Tokyo, Japan) or a Bruker Biospin AG-400 spectrometer (400 MHz, Bruker Optics, Ettlingen, Germany) was used to measure the ^1^H NMR and ^13^C NMR with tetramethylsilane (TMS) as an internal standard and deuterated chloroform as the solvent. High-resolution mass spectrometry (HRMS) data were obtained on a Q-Exactive Orbitrap MS apparatus (Thermo Fisher Scientific, Waltham, MA, USA). Olympus BX53 microscope (Olympus, Tokyo, Japan) and a FluoroMax^®^-4P fluorescence spectrophotometer (HORIBA Scientific, Paris, France) tested bacterial fluorescence images and fluorescence intensity, respectively. Bacteria enzymatic activity test and in vitro antibacterial test were performed using a Cytation™ 5 multi-mode readers (BioTek Instruments, Inc., Winooski, VT, USA). 18*β*-GA and UA were the starting material purchased from Energy-Chemical (Anhui Zesheng Technology Co., Ltd., Anhui, China).

### 3.2. General Protocols

The wild-type (WT) *Xanthomonas oryzae* pv. *oryzae* (*Xoo*) strain ZJ173 was kindly provided by Ming-Guo Zhou (Nanjing Agricultural University, Nanjing, China). The *Xanthomonas axonopodis* pv. *citri* (*Xac*) strain was preserved in our lab. In vitro and in vivo antimicrobial bioassays of the title molecules and measurement of enzyme activities were performed according to previously reported methods [49,50,51,52].

### 3.3. ROS Detection Assay

The ability of compound A_22_ to affect redox homeostasis in *Xoo* cells was assessed by measurements of ROS levels. The ROS assay kit (S0033, Beyotime, Shanghai, China) was used to evaluate *Xoo* cellular ROS levels. First, plant pathogens were cultured to logarithmic growth (OD_595_ = 0.2) and then treated with compound A_22_ (0, 6.25, 12.5, and 25.0 mg L^–1^) for 12 h at 28 °C. Next, bacterial cells were harvested by centrifugation (6000 rpm, 3.5 min, and 4 °C), washed twice with sterile water, and resuspended. Finally, 100 μL of the bacterial solution containing 1 μL of S0033 staining solution was incubated for 20 min at 28 °C and further observed under an Olympus BX53 microscope.

### 3.4. Lipid Peroxidation Assay

Malondialdehyde (MDA) is living organisms’ natural lipid oxidation product [53]. Some fatty acids are oxidized and gradually degraded into complex compounds, including MDA [42]. Therefore, MDA levels can be detected as an indicator of lipid peroxidation. *Xoo* cells were collected and washed with precooled phosphate-buffered saline (PBS) at a pH of 7.2. After that step, bacteria were resuspended in PBS and were subject to ultrasonication (SONICS ultrasonic crusher VCX150 (Newtown, CT, USA), power 150 W (30%), sonication 3 s, interval 10 s, repeated 60 times at 0 °C). The supernatant was centrifuged at 8000× *g*, 4 °C for 10 min, and the MDA content was measured according to the kit instructions (S0131S, Beyotime, Shanghai, China). Protein concentrations were determined using the Bradford method. The optical density (OD) value was measured using an enzyme marker.

### 3.5. Plate Assay

The hydrogen peroxide (H_2_O_2_) plate assay was based on the method reported in the literature with some modifications [54,55]. Nutrient agar (NA) containing 15 g L^−1^ agar powder, H_2_O_2_ (0.05 mM) and compound A_22_ (12.5 mg L^−1^), NA with H_2_O_2_ (0.05 mM) and compound A_22_ (12.5 mg L^−1^), and a blank control were prepared. A 10 µL sample of bacterial solution was spread on the NA Petri dish, covered in a 28 °C incubator, incubated for 48 h, after which the size and growth of the bacterial cake were observed and photographs were taken. Three replicates were performed for each treatment, and the experiment was repeated three times.

### 3.6. Assay of Outer Membrane Damage

The effect of the title compound A_22_ on the *Xoo* cell outer membrane was investigated according to the method reported in the literature with some modifications [56]. First, compound A_22_ was added to bacteria at different concentrations (6.25, 12.5, 25.0, and 50.0 mg L^−1^) for incubation. Next, *Xoo* were centrifuged (6000 rpm, 3.5 min, 4 °C), washed, and resuspended in PBS to obtain a *Xoo* cell resuspension. *N*-phenyl-1-naphthylamine (NPN) dye (10 M, 50 μL) was added to the resulting resuspension solution and incubated at 28 °C for 30 min. After incubation, the bacterial suspension was removed and washed twice with PBS. Finally, the fluorescence intensity was measured using a fluorescence spectrophotometer (λ_ex_ = 350 nm, λ_em_ = 415 nm). Three parallel experiments were conducted for each group.

### 3.7. Study of Inner Membrane Permeabilization

The cell membrane permeability experiment was performed according to the previously reported method with slight modifications [57]. *Xoo* cells (OD_595_ = 0.2) were cultured with different doses of A_22_ (6.25, 12.5, 25.0, and 50.0 mg L^−1^) and then collected (6000 rpm, 3.5 min, 4 °C) and washed twice with PBS. After that step, propidium iodide (PI, 20 μM, 10 μL) was added to stain the bacteria, and fluorescence intensity was tested using a fluorescence spectrophotometer (λ_ex_ = 535 nm, 5 nm slit width; λ_em_ = 617, 5 nm slit width). Three parallel experiments were performed for each group.

## 4. Conclusions

This paper describes the synthesis of 18*β*-GA and UA derivatives, which were screened for their antibacterial activity against two phytopathogenic bacteria. Compound A_22_ showed the best activity against *Xoo* and *Xac* with EC_50_ values of 3.34 mg L^−1^ and 3.30 mg L^−1^, respectively. Compound A_22_ was then used to investigate the antimicrobial mechanism of action of *Xoo*. First, the assay results, such as increased ROS levels, decreased CAT and SOD enzyme activities, and increased MDA content, indicate that compound A_22_ could disrupt the redox system in *Xoo* cells, trigger oxidative stress, and cause lipid peroxidation. Second, NPN and PI staining revealed that compound A_22_ also triggered *Xoo* cell outer membrane damage and increased its permeability, ultimately leading to bacterial death. Finally, we demonstrated the excellent antibacterial activity of compound A_22_ both in vitro and in vivo using pot tests. In summary, the novel 18*β*-GA and UA derivatives can be used as lead compounds for pesticide development by inducing an ROS burst.

## Figures and Tables

**Table 1:** Anti-*Xoo* and anti-*Xac* preliminary activities’ results of title compounds A_1_–A_21_ and B_1_–B_7_.

No.	*Xoo*, Inhibition (%)		*Xac*, Inhibition (%)	
	100 mg L^−1^	50 mg L^−1^	100 mg L^−1^	50 mg L^−1^
A_1_	14.01 ± 0.48	0	27.39 ± 1.82	16.97 ± 0.35
A_2_	25.05 ± 0.48	16.59 ± 0.34	24.82 ± 0.83	10.78 ± 1.18
A_3_	28.35 ± 0.62	16.76 ± 0.43	24.10 ± 1.74	14.89 ± 0.16
A_4_	25.82 ± 1.48	25.07 ± 1.22	13.59 ± 0.68	11.34 ± 0.11
A_5_	14.53 ± 0.61	0	12.44 ± 0.54	0
A_6_	0	0	18.32 ± 1.02	0
A_7_	100	100	100	100
A_8_	26.20 ± 0.69	4.80 ± 1.54	54.97 ± 0.54	42.66 ± 0.43
A_9_	49.01 ± 1.31	43.22 ± 2.16	45.65 ± 1.10	14.51 ± 1.69
A_10_	11.48 ± 0.40	0	14.11 ± 0.56	0
A_11_	26.76 ± 0.49	18.08 ± 0.32	25.43 ± 0.90	13.23 ± 0.53
A_12_	14.34 ± 1.15	0	26.48 ± 0.74	19.86 ± 0.20
A_13_	0	0	28.08 ± 0.38	19.93 ± 0.96
A_14_	12.47 ± 0.73	0	25.92 ± 0.65	17.41 ± 0.37
A_15_	27.53 ± 1.17	26.81 ± 0.37	18.38 ± 0.64	13.92 ± 0.58
A_16_	20.93 ± 1.42	11.10 ± 1.63	24.76 ± 1.12	19.37 ± 2.40
A_17_	20.46 ± 0.57	13.41 ± 0.29	17.31 ± 0.65	11.64 ± 0.64
A_18_	22.37 ± 0.34	16.54 ± 0.42	19.15 ± 0.74	14.62 ± 0.44
A_19_	100	100	54.42 ± 1.13	46.86 ± 0.63
A_20_	83.86 ± 0.95	46.38 ± 0.32	53.20 ± 0.90	42.96 ± 1.53
A_21_	12.58 ± 0.14	0	26.99 ± 0.87	4.51 ± 0.84
B_1_	65.71 ± 0.54	50.05 ± 0.46	36.92 ± 1.03	0
B_2_	39.61 ± 0.17	23.37 ± 0.26	33.21 ± 0.54	26.42 ± 0.70
B_3_	35.99 ± 0.35	17.63 ± 0.19	24.64 ± 0.46	16.78 ± 0.34
B_4_	100	96.15 ± 0.55	100	100
B_5_	24.25 ± 0.46	13.63 ± 0.28	20.15 ± 0.32	16.22 ± 0.16
B_6_	100	100	100	100
B_7_	47.25 ± 0.29	47.25 ± 0.29	33.89 ± 0.75	23.44 ± 0.56
BT	100	98.32 ± 0.41	37.91 ± 0.77	25.11 ± 0.39
TC	45.85 ± 0.43	23.14 ± 0.73	43.91 ± 0.11	24.12 ± 0.49

**Table 2:** Anti-*Xoo* and anti-*Xac* preliminary activities’ results of title compounds A_22_–A_27_ and B_8_–B_10_.

No.	*Xoo*, Inhibition (%)		*Xac*, Inhibition (%)	
	100 mg L^−1^	50 mg L^−1^	100 mg L^−1^	50 mg L^−1^
A_22_	100	100	100	100
A_23_	100	100	100	100
A_24_	100	100	100	100
A_25_	100	100	100	100
A_26_	100	100	100	100
A_27_	72.76 ± 0.75	49.29 ± 0.90	73.31 ± 1.78	63.56 ± 0.82
B_8_	100	100	100	100
B_9_	100	100	100	100
B_10_	100	100	100	100
BT	100	98.32 ± 0.41	37.91 ± 0.77	25.11 ± 0.39
TC	45.85 ± 0.43	23.14 ± 0.73	43.91 ± 0.11	24.12 ± 0.49

**Table 3:** Effective concentrations of 50% maximum antibacterial activity (EC_50_) values of title compounds against *Xoo* and *Xac* in vitro.

No.	*Xoo*		*Xac*	
	Regression Equation	EC_50_ (mg L^−1^)	Regression Equation	EC_50_ (mg L^−1^)
A_7_	y = 3.5452x + 0.3240	20.84 ± 0.13	y = 1.7082x + 3.9066	4.37 ± 0.04
A_8_		>100	y = 1.2135x + 2.6610	84.62 ± 2.12
A_19_	y = 1.8221x + 2.4622	24.71 ± 0.36	y = 0.9400x + 3.3694	54.29 ± 1.04
A_20_	y = 1.8413x + 1.9751	43.93 ± 014	y= 1.1593x + 2.9623	57.24 ± 0.91
A_22_	y = 7.2107x + 1.2254	3.34 ± 0.10	y = 1.7068x + 4.0860	3.30 ± 0.05
A_23_	y = 5.6216x + 0.4634	6.41 ± 0.09	y = 4.5234x + 2.2644	4.03 ± 0.12
A_24_	y = 3.9871x + 1.4228	7.89 ± 0.52	y = 2.6194x + 3.1286	5.18 ± 0.05
A_25_	y = 4.7663x + 1.6672	5.00 ± 0.04	y = 4.8039x + 0.2598	9.70 ± 0.19
A_26_	y = 5.0248x + 1.9465	4.05 ± 0.03	y = 2.4930x + 3.2891	4.86 ± 019
A_27_	y = 2.1043x + 1.7011	36.96 ± 0.39	y= 1.2455x + 3.1459	30.81 ± 0.97
B_1_	y = 0.9824x + 3.4146	41.10 ± 0.47		>100
B_4_	y = 3.0673x + 2.1599	8.43 ± 0.03	y = 2.7998x + 3.0624	4.92 ± 0.10
B_6_	y = 6.1769x − 1.1890	10.05 ± 0.20	y = 3.4013x + 2.6057	5.06 ± 0.06
B_8_	y = 5.0369x + 0.1523	9.17 ± 0.08	y = 2.9158x + 3.0058	4.82 ± 0.03
B_9_	y = 1.3787x + 3.9682	5.60 ± 0.03	y = 4.7978x + 1.7313	4.80 ± 0.05
B_10_	y = 4.3891x + 2.1653	4.42 ± 0.12	y = 3.1919x + 2.9068	4.53 ± 0.07
BT	y = 2.0170x + 2.0704	28.34 ± 0.39	y = 2.6471x − 0.3129	101.64 ± 0.76
TC	y = 2.0774x + 06817	119.87 ± 3.47	y = 3.7668x − 2.2971	86.54 ± 0.18

**Table 4:** Control effects of compound A_22_ against rice bacterial blight under greenhouse conditions at 200 mg L^−1^ in vivo.

Chemicals	Curative Activity			Protective Activity		
	Morbidity (%)	Disease Index (%)	Control Efficiency (%) ^b^	Morbidity (%)	Disease Index (%)	Control Efficiency (%) ^b^
A_22_	100	46.67	44.74 B	100	42.22	50.44 B
A_22_-OPO	100	33.33	59.65 A	100	35.56	58.26 A
A_22_-OSi	100	34.07	60.53 A	100	31.85	62.61 A
BT	100	51.11	39.47 C	100	50.37	40.87 C
TC	100	51.85	38.60 C	100	53.33	37.39 C
CK ^a^	100	84.44		100	85.19	

^a^ Negative contro. ^b^ Statistical analysis.

**Table 5:** In vivo control efficiency (14 days after spraying) of A_22_ against citrus bacterial canker at 200 mg L^−1^.

Chemicals	Protective Efficiency (%) ^a^	Curative Efficiency (%) ^a^
A_22_	84.37 ± 10.13 A	58.86 ± 17.08 A
TC	74.44 ± 14.63 B	51.51 ± 7.49 B

^a^ Statistical analysis. The Tukey’s HSD test was used to perform one-way ANOVA analysis between the different treatments; different uppercase letters indicate the values of control efficiency with a significant difference among different treatment groups at a level of *p* < 0.05.

## Data Availability

Not applicable.

## Supplementary Materials

The following supporting information can be downloaded at: https://www.mdpi.com/article/10.3390/ijms241310566/s1.
