# Supplementary material for: Discovery of Novel Pentacyclic Triterpene Acid Amide Derivatives as Excellent Antimicrobial Agents Dependent on Generation of Reactive Oxygen Species
Source: Int J Mol Sci. 2023 Jun 24;24(13):10566. doi: 10.3390/ijms241310566 (PMC10341837; doi:10.3390/ijms241310566)
Supplement: Supplementary file 1 [file ijms-24-10566-s001.zip › ijms-2433157-supplementary.pdf]

Supporting Information

Discovery of novel pentacyclic triterpene acid amide derivatives as excellent antimicrobial agents dependent on generation of reactive oxygen species

Contents

1.General synthetic process for intermediate and target compounds 2

2.Characterization data of all intermediate and target compounds................................3

3.^{1}H NMR, ^{13}C NMR, HRMS spectra for all compounds (Figures S1 to S114).........29

3. *In vivo* phytotoxicity of rice results (Figure S115). 86

1.General synthetic process for intermediate and target compounds

1.1. Synthesis of the intermediate 1.

Weigh 18*β*-glycyrrhetinic acid (2.00 g, 4.25 mmol) in a 100 mL round bottom flask and add 5 mL of anhydrous pyridine and stir to dissolve. Then DMAP (0.31 g, 0.51 mmol) and acetic anhydride (1.73 g, 17.00 mmol) were added. The reaction was stopped at room temperature until the solution changed from clear to milky white, desolvated under reduced pressure, and acidified with 30 mL of water and hydrochloric acid to pH = 2-3. Finally, the crude product was extracted and dried to obtain intermediate **1**.

1.2. Synthesis of the target compounds A_1-A_{21}.

Weigh 18*β*-glycyrrhetinic acid (0.30 g, 0.64 mmol), EDCI (0.16 g, 0.83 mmol), and HOBT (0.09 g, 0.64 mmol) in a 100 mL round bottom flask and add 10 mL of dichloromethane to dissolve. Afterward, dropwise, propylamine (0.07 g, 1.15 mmol) and 0.18 mL of triethylamine were added. After TLC monitored the reaction, the solvent was removed under vacuum, and the crude product was extracted with ethyl acetate (3×20 mL). The organic phase was further rinsed with water, dried over anhydrous sodium sulfate, and the solvent was removed under vacuum. Finally, the desired product was purified by a silica gel using CH_2Cl_2 and CH_3OH (V : V = 100:1 - 80:1) as the eluent to afford the target compounds **A_1-A_{22}**.

1.3. Synthesis of the target compounds A_{22}-A_{27}.

Weigh 0.2 g of target compound **A_4** in a bottom garden flask, add 5 mL of methanol, and stir to dissolve. Then add 2 mL of hydrochloric acid and 400 *μ*L water

to the reaction at room temperature. Stop the reaction after the solution is clarified and cloudy, extract 30 mL of ethyl acetate, and wash with plenty of water. Adjust the pH of the water layer to about 9 and leave it overnight to obtain the crude product by filtration. Finally, the desired product was purified by a silica gel using CH_2Cl_2 and CH_3OH (V : V = 60:1 - 10:1) as the eluent to afford the target compound **A_{22}-A_{27}**.

1.4. Synthesis of the target compounds B_1-B_{10}.

The following target compounds, **B_1-B_{10}** were synthesized using the above A-series target compounds synthesis steps.

2.Characterization data of all intermediate and target compounds

3*β*-acetoxy-11-oxo-olean-12-en-30-amide (1)

white solid, yield 91.2%, ^{1}H NMR (400 MHz, $CDCl_3$) *δ* 5.71 (s, 1H, 12-CH=C), 4.51 (dd, *J* = 11.6, 4.8 Hz, 1H, 3-<u>CH</u>COOCH$_3$), 2.79 (dd, *J* = 10.2, 3.4 Hz, 1H, 1a-H), 2.36 (s, 1H, 9-H), 2.18 (dd, *J* = 13.3, 3.5 Hz, 1H, 18-H), 2.05 (s, 3H, 3-CHCOO<u>CH$_3$</u>), 2.00–1.90 (m, 2H, 16a-H+19a-H), 1.87–1.77 (m, 1H, 21a-H), 1.75–1.67 (m, 1H, 15a-H), 1.67–1.51 (m, 5H, 6a-H+2-H+7a-H+21b-H), 1.48–1.33 (m, 5H, 6b-H+7b-H+19b-H+22-H), 1.36 (s, 3H, 27-CH_3), 1.22 (s, 3H, 25-CH_3), 1.20–0.99 (m, 3H, 15b-H+16b-H+1b-H), 1.16 (s, 3H, 26-CH_3), 1.12 (s, 3H, 29-CH_3), 0.87 (s, 6H, 23-CH_3+24-CH_3), 0.83 (s, 3H, 28-CH_3), 0.80 (d, *J* = 10.6 Hz, 1H, 5-H); ^{13}C NMR (101 MHz, $CDCl_3$) *δ* 200.5, 181.7, 171.2, 169.6, 128.4, 80.7, 61.7, 55.0, 48.3, 45.5, 43.8, 43.2, 40.8, 38.8, 38.1, 37.7, 36.9, 32.7, 31.9, 30.9, 28.6, 28.5, 28.1, 26.5, 26.4, 23.6, 23.4, 21.4, 18.7, 17.4, 16.7, 16.5. HRMS (ESI): m/z calcd for $C_{33}H_{54}O_3N^+$:

512.4098; found: 512.4104.

***N*-propyl-3*β*-hydroxy-11-oxo-olean-12-en-30-amide (A_1)**

white solid, yield 92.5 %, m. p. 149.1-151.2 °C; ^{1}H NMR (500 MHz, $CDCl_3$) *δ* 5.90 (t, *J* = 5.5 Hz, 1H, CONH), 5.60 (s, 1H, 12-CH=C), 3.27–3.10 (m, 3H, 1′-NH$\underline{CH_2}$$CH_2CH_3$+3-$\underline{CH}$OH), 2.72 (dd, *J* = 10.2, 3.3 Hz, 1H, 1a-H), 2.29 (s, 1H, 9-H), 2.11 (dd, *J* = 13.1, 3.5 Hz, 1H, 18-H), 2.05–1.91 (m, 2H, 16a-H+19a-H), 1.89 (s, 1H, 21a-H), 1.80–1.74 (m, 1H, 15a-H), 1.69–1.53 (m, 5H, 6a-H+2-H+7a-H+21b-H), 1.48 (dd, *J* = 14.5, 7.3 Hz, 2H, 2′-NH$CH_2$$\underline{CH_2}$$CH_3$), 1.43–1.30 (m, 5H, 6b-H+7b-H+19b-H+22-H), 1.33 (s, 3H, 27-CH_3), 1.15–0.91 (m, 3H, 15b-H+16b-H+1b-H), 1.08 (d, *J* = 3.2 Hz, 9H, 25-CH_3+26-CH_3+29-CH_3), 0.96 (s, 3H, 23-CH_3), 0.88 (t, *J* = 7.4 Hz, 3H, 3′-NH$CH_2CH_2$$\underline{CH_3}$), 0.76 (d, *J* = 3.5 Hz, 6H, 24-CH_3+28-CH_3), 0.65 (d, *J* = 11.6 Hz, 1H, 5-H); ^{13}C NMR (126 MHz, $CDCl_3$) *δ* 200.3, 175.7, 169.6, 128.4, 78.7, 61.9, 55.0, 48.3, 45.4, 43.6, 43.3, 41.9, 41.2, 39.2, 39.2, 37.5, 37.1, 32.8, 32.0, 31.5, 29.7, 28.6, 28.2, 27.3, 26.5, 26.4, 23.4, 23.1, 18.7, 17.5, 16.4, 15.7, 11.6; HRMS (ESI): m/z calcd for $C_{33}H_{54}O_3N^+$: 512.4098; found: 512.4104.

***N*-butyl-3*β*-hydroxy-11-oxo-olean-12-en-30-amide (A_2)**

white solid, yield 88.1 %, m. p. 140.2-141.9 °C; ^{1}H NMR (400 MHz, $CDCl_3$) *δ* 5.81 (t, *J* = 5.1 Hz, 1H, CONH), 5.61 (s, 1H, 12-CH=C), 3.32–3.13 (m, 3H, 1′-NH$\underline{CH_2}$$CH_2CH_3$+3-$\underline{CH}$OH), 2.74 (dd, *J* = 10.2, 3.2 Hz, 1H, 1a-H), 2.30 (s, 1H, 9-H), 2.12 (dd, *J* = 12.7, 3.8 Hz, 1H, 18-H), 2.05–1.90 (m, 2H, 16a-H+19a-H), 1.80 (d, *J* = 4.3 Hz, 1H, 21a-H), 1.75 (d, *J* = 11.9 Hz, 1H, 15a-H), 1.62–1.55 (m, 5H,

6a-H+2-H+7a-H+21b-H), 1.50–1.38 (m, 4H, 2′-$NHCH_2\underline{CH_2CH_2}CH_3$), 1.37–1.28 (m, 5H, 6b-H+7b-H+19b-H+22-H), 1.34 (s, 3H, 27-CH_3), 1.16–1.01 (m, 3H, 15b-H+16b-H+1b-H), 1.09 (s, 9H, 25-CH_3+26-CH_3+29-CH_3), 0.97 (s, 3H, 23-CH_3), 0.89 (t, J = 7.3 Hz, 3H, 4′-$NHCH_2CH_2CH_2\underline{CH_3}$), 0.78 (s, 3H, 24-$CH_3$), 0.77 (s, 3H, 28-$CH_3$), 0.66 (d, J = 11.6 Hz, 1H, 5-H); ^{13}C NMR (101 MHz, $CDCl_3$) δ 200.3, 175.6, 169.5, 128.4, 78.7, 61.8, 54.9, 48.2, 45.4, 43.6, 43.2, 41.8, 39.2, 39.2, 37.5, 37.1, 32.8, 31.9, 31.9, 31.5, 29.6, 28.5, 28.1, 27.2, 26.5, 26.4, 23.4, 20.2, 18.7, 17.5, 16.4, 15.6, 13.8; HRMS (ESI): m/z calcd for $C_{34}H_{56}O_3N^+$: 526.4255; found: 526.4264.

***N*-(3-isopropoxypropyl)-3*β*-hydroxy-11-oxo-olean-12-en-30-amide (A_3)**

white solid, yield 90.5 %, m. p. 144.3-145.4 °C; ^1H NMR (400 MHz, $CDCl_3$) δ 6.66 (t, J = 4.8 Hz, 1H, CONH), 5.62 (s, 1H, 12-CH=C), 3.53–3.43 (m, 3H, 4′-$O\underline{CH}CH_3CH_3$+3′-$NHCH_2CH_2\underline{CH_2}O$), 3.37–3.23 (m, 2H, 1′-$NH\underline{CH_2}CH_2CH_2O$), 3.15 (dd, J = 11.0, 5.2 Hz, 1H, 3-$\underline{CH}OH$), 2.70 (dd, J = 10.3, 3.2 Hz, 1H, 1a-H), 2.26 (s, 1H, 9-H), 2.09 (dd, J = 13.2, 3.0 Hz, 1H, 18-H), 2.02–1.94 (m, 1H, 16a-H), 1.83–1.66 (m, 5H, 19a-H+21a-H+15a-H+2′-$NHCH_2\underline{CH_2}CH_2O$), 1.63–1.50 (m, 5H, 6a-H+2-H+7a-H+21b-H), 1.40–1.28 (m, 5H, 6b-H+7b-H+19b-H+22-H), 1.30 (s, 3H, 27-CH_3), 1.14–1.84 (m, 3H, 15b-H+16b-H+1b-H), 1.09 (d, J = 1.7 Hz, 3H, 29-CH_3), 1.07 (d, J = 1.8 Hz, 3H, 25-CH_3), 1.06 (s, 3H, 26-CH_3), 1.05 (d, J = 1.3 Hz, 6H, 5′-$OCH\underline{CH_3}CH_3$+6′-$OCHCH_3\underline{CH_3}$), 0.93 (s, 3H, 23-CH_3), 0.73 (s, 6H, 24-CH_3+28-CH_3), 0.62 (d, J = 11.5 Hz, 1H, 5-H); ^{13}C NMR (101 MHz, $CDCl_3$) δ 200.0, 175.5, 169.3, 128.5, 78.6, 71.9, 68.0, 61.8, 54.9, 48.0, 45.3, 43.4, 43.1, 41.6, 39.2, 39.1, 39.0, 37.5, 37.0, 32.7, 31.84, 31.3, 29.6, 29.3, 28.5, 28.1, 27.2, 26.5, 26.4,

23.4, 22.2, 22.1, 18.6, 17.5, 16.3, 15.7; HRMS (ESI): m/z calcd for $C_{36}H_{60}O_4N^+$: 570.4517; found: 570.4522.

1-(Boc-piperidine)-3*β*-hydroxy-11-oxo-olean-12-en-30-amide (A_4)

white solid, yield 97.1 %, m. p. 136.6-138.2 °C; ^{1}H NMR (400 MHz, $CDCl_3$) *δ* 5.60 (s, 1H, CONH), 5.45 (d, *J* = 8.0 Hz, 1H, 12-CH=C), 4.14–3.87 (m, 14.9 Hz, 3H, 1′-NHCH+3′a-NCH_2), 3.21 (dd, *J* = 10.7, 5.5 Hz, 1H, 3-CHOH), 2.83 (s, 2H, 3′b-NCH_2), 2.80–2.73 (m, 1H, 1a-H), 2.32 (s, 1H, 9-H), 2.16–2.09 (m, 1H, 18-H), 2.02 (td, *J* = 13.5, 4.1 Hz, 2H, 16a-H+19a-H), 1.87 (d, *J* = 5.0 Hz, 3H, 21a-H+2′a-NCH_2CH_2), 1.83–1.78 (m, 1H, 15a-H), 1.73–1.57 (m, 7H, 6a-H+2-H+7a-H+21b-H+2′b-NCH_2CH_2), 1.44 (s, 9H, 5′-$OC(CH_3)_3$), 1.43–1.34 (m, 5H, 6b-H+7b-H+19b-H+22-H), 1.35 (s, 3H, 27-CH_3), 1.18–0.93 (m, 3H, 15b-H+16b-H+1b-H), 1.12 (s, 3H, 29-CH_3), 1.11 (s, 3H, 25-CH_3), 1.10 (s, 3H, 26-CH_3), 0.99 (s, 3H, 23-CH_3), 0.79 (s, 6H, 24-CH_3+28-CH_3), 0.68 (d, *J* = 11.6 Hz, 1H, 5-H); ^{13}C NMR (101 MHz, $CDCl_3$) *δ* 200.1, 175.1, 169.3, 154.6, 128.4, 79.6, 78.6, 61.8, 54.9, 48.2, 46.6, 45.4, 43.4, 43.2, 41.7, 39.1, 37.5, 37.1, 32.7, 32.1, 31.9, 31.4, 29.7, 29.4, 28.6, 28.4, 28.1, 27.2, 26.4, 26.4, 23.3, 18.6, 17.5, 16.3, 15.6; HRMS (ESI): m/z calcd for $C_{40}H_{63}O_5N_2^+$: 651.4731; found: 651.4752.

1-((Boc-4-methy)-piperidine)-3*β*-hydroxy-11-oxo-olean-12-en-30-amide (A_5)

white solid, yield 97.9 %, m. p. 122.4-123.5 °C; ^{1}H NMR (400 MHz, $CDCl_3$) *δ* 5.96 (d, *J* = 18.8 Hz, 1H, CONH), 5.59 (d, *J* = 2.0 Hz, 1H, 12-CH=C), 4.04 (s, 2H, 4′a-NCH_2), 3.21–3.15 (m, 1H, 3-CHOH), 3.12 (s, 2H, 4′b-NCH_2), 2.72 (d, *J* = 13.0 Hz, 1H, 1a-H), 2.63 (s, 2H, 1′-$NHCH_2$), 2.29 (s, 1H, 9-H), 2.11 (d, *J* = 12.8 Hz, 1H,

18-H), 2.06–1.93 (m, 2H, 16a-H+19a-H), 1.88 (d, J = 7.1 Hz, 1H, 21a-H), 1.77 (d, J = 13.4 Hz, 1H, 15a-H), 1.63 (dd, J = 31.4, 14.0 Hz, 10H, 6a-H+2-H+7a-H+21b-H+2′-NHCH$_2$<u>CH</u>+3′-NCH$_2$<u>CH$_2$</u>), 1.42–1.32 (m, 14H, 6b-H+7b-H+19b-H+22-H+6′-OC(CH$_3$)$_3$), 1.33 (s, 3H, 27-CH$_3$), 1.17–0.90 (m, 3H, 15b-H+16b-H+1b-H), 1.08 (s, 9H, 29-CH$_3$+25-CH$_3$+26-CH$_3$), 0.96 (s, 3H, 23-CH$_3$), 0.76 (s, 6H, 24-CH$_3$+28-CH$_3$), 0.65 (d, J = 11.4 Hz, 1H, 5-H); ^{13}C NMR (101 MHz, CDCl$_3$) δ 200.2, 176.0, 169.4, 154.8, 128.4, 79.4, 78.6, 61.8, 54.9, 48.2, 45.4, 44.9, 43.7, 43.2, 41.7, 39.2, 39.1, 37.5, 37.1, 36.5, 32.7, 31.9, 31.5, 29.9, 29.7, 28.5, 28.4, 28.1, 27.2, 26.4, 26.4, 23.3, 18.7, 17.5, 16.3, 15.6; HRMS (ESI): m/z calcd for $C_{41}H_{66}O_5N_2Na^+$: 689.4864; found: 689.4861.

***N*-((tetrahydro-2H-pyran-4-yl)methyl)-3*β*-hydroxy-11-oxo-olean-12-en-30-amide (A$_6$)**

white solid, yield 54.3 %, m. p. 135.8-137.6 °C; ^{1}H NMR (500 MHz, CDCl$_3$) δ 5.94 (t, J = 5.5 Hz, 1H, CONH), 5.60 (s, 1H, 12-CH=C), 3.94 (dd, J = 11.2, 2.9 Hz, 2H, 4′a-O<u>CH$_2$</u>), 3.33 (t, J = 11.7 Hz, 2H, 4′b-O<u>CH$_2$</u>), 3.18 (dd, J = 10.9, 5.0 Hz, 1H, 3-<u>CH</u>OH), 3.14 (t, J = 6.1 Hz, 2H, 1′-NH<u>CH$_2$</u>), 2.73 (d, J = 13.5 Hz, 1H, 1a-H), 2.30 (s, 1H, 9-H), 2.12–2.10 (m, 1H, 18-H), 2.04–1.88 (m, 2H, 16a-H+19a-H), 1.80–1.76 (m, 2H, 21a-H+2′-NHCH$_2$<u>CH</u>), 1.70 (d, J = 13.7 Hz, 1H, 15a-H), 1.66–1.51 (m, 7H, 6a-H+2-H+7a-H+21b-H+3′a-NHCH$_2$CH<u>CH$_2$</u>), 1.44–1.33 (m, 5H, 6b-H+7b-H+19b-H+22-H), 1.34 (s, 3H, 27-CH$_3$), 1.28 (dd, J = 12.4, 3.8 Hz, 2H, 3′b-NHCH$_2$CHCH$_2$), 1.16–0.89 (m, 3H, 15b-H+16b-H+1b-H), 1.10 (s, 3H, 29-CH$_3$), 1.09 (s, 6H, 25-CH$_3$+26-CH$_3$), 0.97 (s, 3H, 23-CH$_3$), 0.78 (d, J = 12.5 Hz, 3H,

24-CH_3), 0.77 (s, 3H, 28-CH_3), 0.66 (d, J = 11.6 Hz, 1H, 5-H); ^{13}C NMR (126 MHz, $CDCl_3$) δ 200.3, 176.0 , 169.5, 128.5, 78.7, 67.7, 61.9, 55.0, 48.3, 45.5, 45.3, 43.8, 43.3, 41.8, 39.2, 37.6, 37.1, 35.5, 32.8, 32.0, 31.6, 30.8, 29.9, 28.6, 28.2, 27.3, 26.5, 26.4, 23.5, 18.7, 17.5, 16.5, 15.7; HRMS (ESI): m/z calcd for $C_{36}H_{56}O_4N^-$: 566.4204; found: 566.4218.

***N*-(3-(piperidin-1-yl)propyl)-3*β*-hydroxy-11-oxo-olean-12-en-30-amide (A_7)**

white solid, yield 97.56 %, m. p. 129.3-130.8 °C; ^{1}H NMR (400 MHz, $CDCl_3$) δ 7.61 (s, 1H, CONH), 5.63 (s, 1H, 12-CH=C), 3.44–3.34 (m, 1H, 1′a-NH$\underline{CH_2}$), 3.31–3.23 (m, 1H, 1′b-NH$\underline{CH_2}$), 3.19 (dd, J = 10.9, 5.2 Hz, 1H, 3-$\underline{CH}$OH), 2.74 (d, J = 13.5 Hz, 1H, 1a-H), 2.49–2.31 (m, 6H, 3′-$NHCH_2CH_2\underline{CH_2}$+4′-$NCH_2$), 2.28 (s, 1H, 9-H), 2.12 (d, J = 12.7 Hz, 1H, 18-H), 2.04 – 1.91 (m, 2H, 16a-H+19a-H), 1.85 (d, J = 13.5 Hz, 1H, 21a-H), 1.79 (dd, J = 13.5, 4.2 Hz, 1H, 15a-H), 1.69–1.50 (m, 11H, 6a-H+2-H+7a-H+21b-H+2′-$NHCH_2\underline{CH_2}CH_2$+5′-$NCH_2$), 1.46–1.29 (m, 7H, 6b-H+7b-H+19b-H+22-H+6′-$NCH_2\underline{CH_2}$), 1.34 (s, 3H, 27-CH_3), 1.18–0.91 (m, 3H, 15b-H+16b-H+1b-H), 1.09 (s, 9H, 25-CH_3+26-CH_3+29-CH_3), 0.97 (s, 3H, 23-CH_3), 0.77 (s, 6H, 24-CH_3+28-CH_3), 0.66 (d, J = 11.4 Hz, 1H, 5-H); ^{13}C NMR (101 MHz, $CDCl_3$) δ 200.0, 175.9, 169.2, 128.3, 78.7, 61.8, 59.2, 54.9, 54.9, 48.3, 45.3, 43.4, 43.2, 41.7, 40.4, 39.1, 37.4, 37.0, 32.8, 31.9, 31.4, 29.6, 28.4, 28.1, 27.3, 26.4, 25.8, 24.4, 24.3, 23.3, 18.6, 17.5, 16.3, 15.6; HRMS (ESI): m/z calcd for $C_{38}H_{63}O_3N_2^+$: 595.4833; found: 595.4824.

***N*-(3-(4-methylpiperazin-1-yl)propyl)-3*β*-hydroxy-11-oxo-olean-12-en-30-amide (A_8)**

white solid, yield 79.53 %, m. p. 144.1-145.7 °C; ^{1}H NMR (400 MHz, $CDCl_3$) δ 7.27 (d, J = 4.5 Hz, 1H, CONH), 5.62 (s, 1H, 12-CH=C), 3.47–3.37 (m, 1H, 1′a-NH$\underline{CH_2}$), 3.32–3.24 (m, 1H, 1′b-NH$\underline{CH_2}$), 3.20 (dd, J = 10.8, 5.3 Hz, 1H, 3-$\underline{CH}$OH), 2.76 (d, J = 13.4 Hz, 1H, 1a-H), 2.72–2.27 (m, 11H, 9-H+3′-$NHCH_2CH_2\underline{CH_2}$+4′-$NCH_2$+5′-$NCH_2$), 2.25 (s, 3H, 6′-$NCH_3$), 2.13 (d, J = 10.4 Hz, 1H, 18-H), 2.05–1.95 (m, 2H, 16a-H+19a-H), 1.85 (d, J = 13.6 Hz, 1H, 21a-H), 1.81–1.72 (m, 1H, 15a-H), 1.71–1.55 (m, 7H, 6a-H+2-H+7a-H+21b-H+2′-$NHCH_2\underline{CH_2}CH_2$), 1.47–1.31 (m, 5H, 6b-H+7b-H+19b-H+22-H), 1.37 (s, 3H, 27-CH_3), 1.18–0.87 (m, 3H, 15b-H+16b-H+1b-H), 1.11 (s, 3H, 29-CH_3), 1.11 (s, 3H, 25-CH_3), 1.10 (s, 3H, 26-CH_3), 0.99 (s, 3H, 23-CH_3), 0.79 (s, 6H, 24-CH_3+28-CH_3), 0.68 (d, J = 11.5 Hz, 1H, 5-H); ^{13}C NMR (101 MHz, $CDCl_3$) δ 200.0, 175.9, 169.1, 128.4, 78.7, 61.9, 58.2, 55.0, 53.4, 48.3, 46.0, 45.4, 43.5, 43.2, 41.8, 40.2, 39.2, 39.2, 37.4, 37.1, 32.8, 31.9, 31.4, 29.7, 28.4, 28.1, 27.3, 26.5, 26.4, 24.6, 23.2, 18.7, 17.5, 16.3, 15.6; HRMS (ESI): m/z calcd for $C_{38}H_{64}O_3N_3^+$: 610.4942; found: 610.4933.

1-((Boc-4-propyl)-piperazine)-3*β*-hydroxy-11-oxo-olean-12-en-30-amide (A_9)

white solid, yield 97.3 %, m. p. 123.7-124.8 °C; ^{1}H NMR (400 MHz, $CDCl_3$) δ 7.02 (t, J = 4.4 Hz, 1H, CONH), 5.58 (d, J = 4.7 Hz, 1H, 12-CH=C), 3.46–3.20 (m, 6H, 1′-NH$\underline{CH_2}$+5′-NCH_2), 3.16 (dd, J = 10.9, 5.2 Hz, 1H, 3-$\underline{CH}$OH), 2.77–2.68 (m, 1H, 1a-H), 2.50–2.32 (m, 6H, 3′-$NHCH_2CH_2\underline{CH_2}$+4′-$NCH_2$), 2.27 (d, J = 4.1 Hz, 1H, 9-H), 2.09 (d, J = 13.1 Hz, 1H, 18-H), 1.93 (dd, J = 37.5, 12.8 Hz, 2H, 16a-H+19a-H), 1.77 (d, J = 10.3 Hz, 1H, 21a-H), 1.61 (dd, J = 20.5, 15.1 Hz, 8H,

6a-H+2-H+7a-H+15a-H+21b-H+2′-NHCH2CH2CH2), 1.45–1.30 (m, 14H, 6b-H+7b-H+19b-H+22-H+7′-OC(CH3)3), 1.31 (s, 3H, 27-CH3), 1.13–0.89 (m, 3H, 15b-H+16b-H+1b-H), 1.07 (d, J = 3.1 Hz, 9H, 29-CH3+25-CH3+26-CH3), 0.95 (d, J = 5.1 Hz, 3H, 23-CH3), 0.75 (d, J = 2.8 Hz, 6H, 24-CH3+28-CH3), 0.64 (d, J = 10.5 Hz, 1H, 5-H); ^{13}C NMR (101 MHz, CDCl3) δ 200.0, 175.8, 169.4, 154.6, 128.3, 79.8, 78.6, 61.8, 57.9, 54.9, 53.2, 48.2, 45.3, 43.4, 43.2, 41.8, 39.6, 39.2, 39.1, 37.4, 37.0, 32.7, 31.9, 31.4, 29.6, 28.5, 28.4, 28.1, 27.2, 26.5, 26.4, 24.9, 23.3, 18.6, 17.5, 16.3, 15.7; HRMS (ESI): m/z calcd for $C_{42}H_{70}O_5N_3^+$: 696.5310; found: 696.5301.

***N*-(morpholino)-3*β*-hydroxy-11-oxo-olean-12-en-30-amide (A_{10})**

white solid, yield 73.4 %, m. p. 150.1-152.7 °C; ^{1}H NMR (400 MHz, CDCl3) δ 6.63 (s, 1H, CONH), 5.59 (s, 1H, 12-CH=C), 3.79 (t, J = 4.5 Hz, 4H, 2′-OCH2), 3.19 (dd, J = 10.5, 5.6 Hz, 1H, 3-CHOH), 2.85–2.70 (m, 5H, 1a-H+1′-NCH2), 2.29 (s, 1H, 9-H), 2.16 (dd, J = 13.0, 3.4 Hz, 1H, 18-H), 2.04–1.88 (m, 2H, 16a-H+19a-H), 1.84–1.77 (m, 1H, 21a-H), 1.70 (d, J = 13.5 Hz, 1H, 15a-H), 1.60 (td, J = 12.9, 6.6 Hz, 5H, 6a-H+2-H+7a-H+21b-H), 1.44–1.32 (m, 5H, 6b-H+7b-H+19b-H+22-H), 1.33 (s, 3H, 27-CH3), 1.19–0.90 (m, 3H, 15b-H+16b-H+1b-H), 1.12 (s, 3H, 29-CH3), 1.09 (s, 3H, 25-CH3), 1.09 (s, 3H, 26-CH3), 0.97 (s, 3H, 23-CH3), 0.78 (t, J = 3.2 Hz, 6H, 24-CH3+28-CH3), 0.66 (d, J = 11.7 Hz, 1H,); ^{13}C NMR (101 MHz, CDCl3) δ 200.2, 173.2, 169.4, 128.4, 78.8, 66.4, 61.8, 55.8, 54.9, 48.1, 45.4, 43.2, 42.9, 41.6, 39.2, 39.2, 37.3, 37.1, 32.7, 31.9, 31.5, 29.4, 28.4, 28.1, 27.3, 26.4, 26.4, 23.4, 18.7, 17.5, 16.4, 15.6; HRMS (ESI): m/z calcd for $C_{34}H_{53}O_4N_2^-$: 553.4000; found: 553.4001.

***N*-(thiophen-2-ylmethyl)-3*β*-hydroxy-11-oxo-olean-12-en-30-amide (A_{11})**

white solid, yield 94.9 %, m. p. 138.7-140.6 °C; ^{1}H NMR (400 MHz, $CDCl_3$) δ 7.20 (dd, J = 4.8, 1.4 Hz, 1H, 5′-SCH), 6.92 (dd, J = 4.8, 3.6 Hz, 2H, 3′-SCCH+4′-SCCH$\underline{CH}$), 6.23 (t, J = 5.5 Hz, 1H, CONH), 5.56 (s, 1H, 12-CH=C), 4.67 (dd, J = 15.2, 5.7 Hz, 1H, 1′a-NH$\underline{CH_2}$), 4.57 (dd, J = 15.2, 5.3 Hz, 1H, 1′b-NH$\underline{CH_2}$), 3.19 (dd, J = 10.6, 5.7 Hz, 1H, 3-$\underline{CH}$OH), 2.74 (d, J = 13.5 Hz, 1H, 1a-H), 2.29 (s, 1H, 9-H), 2.13 (dd, J = 12.7, 4.2 Hz, 1H, 18-H), 2.07–1.92 (m, 2H, 16a-H+19a-H), 1.81 (dd, J = 13.5, 8.8 Hz, 1H, 21a-H), 1.73 (d, J = 13.3 Hz, 1H, 15a-H), 1.67–1.54 (m, 5H, 6a-H+2-H+7a-H+21b-H), 1.46–1.33 (m, 5H, 6b-H+7b-H+19b-H+22-H), 1.34 (s, 3H, 27-CH_3), 1.18–0.86 (m, 3H, 15b-H+16b-H+1b-H), 1.13 (s, 3H, 29-CH_3), 1.10 (s, 6H, 25-CH_3+26-CH_3), 0.98 (s, 3H, 23-CH_3), 0.79 (s, 3H, 24-CH_3), 0.78 (s, 3H, 28-CH_3), 0.66 (d, J = 11.7 Hz, 1H, 5-H); ^{13}C NMR (101 MHz, $CDCl_3$) δ 200.2, 175.5, 169.2, 141.6, 128.5, 126.8, 125.8, 125.2, 78.8, 61.8, 55.0, 48.1, 45.4, 43.6, 43.2, 41.7, 39.2, 38.4, 37.4, 37.1, 32.8, 31.9, 31.4, 29.4, 28.4, 28.1, 27.5, 27.3, 26.5, 26.4, 23.3, 18.7, 17.5, 16.4, 15.6; HRMS (ESI): m/z calcd for $C_{35}H_{52}O_3NS^-$: 566.3662; found: 566.3672.

_N_-(furan-2-ylmethyl)-3_β_-hydroxy-11-oxo-olean-12-en-30-amide (A_{12})

white solid, yield 82.1 %, m. p. 129.0-131.3 °C; ^{1}H NMR (400 MHz, $CDCl_3$) δ 7.36 (dd, J = 1.8, 0.7 Hz, 1H, 5′-OCH), 6.30 (dd, J = 3.2, 1.9 Hz, 1H, 4′-OCCH$\underline{CH}$), 6.20 (d, J = 2.9 Hz, 1H, 3′-OCCH), 6.16 (t, J = 5.3 Hz, 1H, CONH), 5.57 (s, 1H, 12-CH=C), 4.50 (dd, J = 15.4, 5.7 Hz, 1H, 1′a-NH$\underline{CH_2}$), 4.35 (dd, J = 15.4, 5.0 Hz, 1H, 1′b-NH$\underline{CH_2}$), 3.18 (dd, J = 10.6, 5.6 Hz, 1H, 3-$\underline{CH}$OH), 2.74 (d, J = 13.5 Hz, 1H, 1a-H), 2.29 (s, 1H, 9-H), 2.09 (dd, J = 11.9, 5.3 Hz, 1H, 18-H), 2.03–1.92 (m, 2H,

16a-H+19a-H), 1.79 (dd, *J* = 13.6, 3.7 Hz, 1H, 21a-H), 1.70 (d, *J* = 6.9 Hz, 1H, 15a-H), 1.69–1.54 (m, 5H, 6a-H+2-H+7a-H+21b-H), 1.45–1.31 (m, 5H, 6b-H+7b-H+19b-H+22-H), 1.33 (s, 3H, 27-CH_3), 1.16–0.87 (m, 3H, 15b-H+16b-H+1b-H), 1.11 (s, 3H, 29-CH_3), 1.10 (s, 3H, 25-CH_3), 1.09 (s, 3H, 26-CH_3), 0.97 (s, 3H, 23-CH_3), 0.77 (s, 3H, 24-CH_3), 0.77 (s, 3H, 28-CH_3), 0.66 (d, *J* = 11.7 Hz, 1H, 5-H); ^{13}C NMR (101 MHz, $CDCl_3$) δ 200.2, 175.6, 169.2, 151.5, 142.2, 128.4, 110.5, 107.3, 78.8, 61.8, 55.0, 48.1, 45.4, 43.7, 43.2, 41.8, 39.2, 39.2, 37.4, 37.0, 36.6, 32.8, 31.9, 31.4, 29.4, 28.4, 28.1, 27.2, 26.5, 26.4, 23.3, 18.7, 17.5, 16.4, 15.6; HRMS (ESI): m/z calcd for $C_{35}H_{52}O_4N^+$: 550.3891; found: 550.3898.

_N_-(4-methylbenzyl)-3_β_-hydroxy-11-oxo-olean-12-en-30-amide (A_{13})

white solid, yield 84.5 %, m. p. 135.5-136.7 °C; ^1H NMR (500 MHz, $CDCl_3$) δ 7.14–7.10 (m, 4H, phenyl-H), 6.18 (dd, *J* = 9.3, 3.8 Hz, 1H, CONH), 5.53 (s, 1H, 12-CH=C), 4.38 (dd, *J* = 5.4, 2.4 Hz, 2H, 1′-NH<u>CH2</u>), 3.15 (dd, *J* = 10.8, 5.4 Hz, 1H, 3-<u>CH</u>OH), 2.71 (dt, *J* = 13.2, 3.2 Hz, 1H, 1a-H), 2.30 (s, 3H, phenyl-<u>CH3</u>), 2.27 (s, 1H, 9-H), 2.12 (dd, *J* = 13.3, 3.4 Hz, 1H, 18-H), 2.05–1.91 (m, 2H, 16a-H+19a-H), 1.80–1.76 (m, 1H, 21a-H), 1.69 (t, *J* = 13.8 Hz, 1H, 15a-H), 1.63–1.51 (m, 5H, 6a-H+2-H+7a-H+21b-H), 1.42–1.31 (m, 5H, 6b-H+7b-H+19b-H+22-H), 1.32 (s, 3H, 27-CH_3), 1.19–0.85 (m, 3H, 15b-H+16b-H+1b-H), 1.12 (s, 3H, 29-CH_3), 1.08 (s, 3H, 25-CH_3+26-CH_3), 0.96 (s, 3H, 23-CH_3), 0.79 (s, 3H, 24-CH_3), 0.75 (s, 3H, 28-CH_3), 0.64 (d, *J* = 11.7 Hz, 1H, 5-H); ^{13}C NMR (126 MHz, $CDCl_3$) δ 200.2, 175.7, 169.3, 137.2, 135.7, 129.5, 128.4, 127.8, 78.8, 61.9, 55.0, 48.2, 45.4, 43.7, 43.5, 43.2, 41.8, 39.3, 39.2, 37.5, 37.1, 32.8, 32.0, 31.5, 29.6, 28.5, 28.2, 27.3, 26.5, 26.5, 23.4, 21.2,

18.7, 17.5, 16.4, 15.7; HRMS (ESI): m/z calcd for $C_{38}H_{54}O_3N^+$: 572.4098; found: 572.4111.

***N*-(4-methoxybenzyl)-3*β*-hydroxy-11-oxo-olean-12-en-30-amide (A_{14})**

white solid, yield 50.6 %, m. p. 137.0-138.7 °C; ^{1}H NMR (400 MHz, $CDCl_3$) δ 7.17 (d, J = 8.6 Hz, 2H, phenyl-H), 6.84 (d, J = 8.7 Hz, 2H, phenyl-H), 6.05 (t, J = 5.4 Hz, 1H, CONH), 5.54 (s, 1H, 12-CH=C), 4.37 (d, J = 5.4 Hz, 2H, 1′-NH$\underline{CH_2}$), 3.77 (s, 3H, phenyl-$\underline{CH_3}$), 3.17 (dd, J = 10.6, 5.6 Hz, 1H, 3-$\underline{CH}$OH), 2.73 (dt, J = 13.1, 3.1 Hz, 1H, 1a-H), 2.28 (s, 1H, 9-H), 2.11 (dd, J = 13.0, 3.7 Hz, 1H, 18-H), 2.05–1.89 (m, 2H, 16a-H+19a-H), 1.83–1.79 (m, 1H, 21a-H), 1.76 (d, J = 2.1 Hz, 1H, 15a-H), 1.71–1.52 (m, 5H, 6a-H+2-H+7a-H+21b-H), 1.44–1.32 (m, 5H, 6b-H+7b-H+19b-H+22-H), 1.33 (s, 3H, 27-CH_3), 1.17–0.85 (m, 3H, 15b-H+16b-H+1b-H), 1.12 (s, 3H, 29-CH_3), 1.09 (s, 6H, 25-CH_3+26-CH_3), 0.97 (s, 3H, 23-CH_3), 0.78 (s, 3H, 24-CH_3), 0.76 (s, 3H, 28-CH_3), 0.65 (d, J = 11.6 Hz, 1H, 5-H); ^{13}C NMR (101 MHz, $CDCl_3$) δ 200.1, 175.6, 169.2, 159.00, 130.8, 129.1, 128.4, 114.1, 78.7, 61.8, 55.4, 54.9, 48.2, 45.4, 43.6, 43.2, 43.1, 41.7, 39.2, 39.2, 37.5, 37.02, 32.7, 31.9, 31.5, 29.6, 28.5, 28.1, 27.2, 26.5, 26.4, 23.4, 18.7, 17.5, 16.4, 15.6; HRMS (ESI): m/z calcd for $C_{38}H_{56}O_4N^+$: 590.4204; found: 590.4183.

***N*-(3-chlorobenzyl)-3*β*-hydroxy-11-oxo-olean-12-en-30-amide (A_{15})**

white solid, yield 91.2 %, m. p. 147.5-148.3 °C; ^{1}H NMR (400 MHz, $CDCl_3$) δ 7.25–7.18 (m, 3H, phenyl-H), 7.12 (dd, J = 6.5, 2.0 Hz, 1H, phenyl-H), 6.48 (t, J = 5.8 Hz, 1H, CONH), 5.51 (s, 1H, 12-CH=C), 4.44–4.38 (m, 2H, 1′-NH$\underline{CH_2}$), 3.18 (dd, J = 10.6, 5.6 Hz, 1H, 3-$\underline{CH}$OH), 2.71 (dd, J = 10.1, 3.3 Hz, 1H, 1a-H), 2.27 (s, 1H, 9-H),

2.12 (dd, *J* = 13.2, 3.3 Hz, 1H, 18-H), 2.07–1.93 (m, 2H, 16a-H+19a-H), 1.82 (dd, *J* = 13.7, 3.5 Hz, 1H, 21a-H), 1.75 (dd, *J* = 17.1, 9.1 Hz, 1H, 15a-H), 1.67–1.53 (m, 5H, 6a-H+2-H+7a-H+21b-H), 1.45–1.30 (m, 5H, 6b-H+7b-H+19b-H+22-H), 1.33 (s, 3H, 27-CH_3), 1.25–0.86 (m, 3H, 15b-H+16b-H+1b-H), 1.15 (s, 3H, 29-CH_3), 1.07 (s, 3H, 25-CH_3), 1.06 (s, 3H, 26-CH_3), 0.97 (s, 3H, 23-CH_3), 0.80 (s, 3H, 24-CH_3), 0.76 (s, 3H, 28-CH_3), , 0.65 (d, *J* = 11.7 Hz, 1H, 5-H); ^{13} CNMR (126 MHz, $CDCl_3$) δ 200.3, 175.6, 169.3, 141.6, 128.5, 126.9, 125.9, 125.3, 78.8, 61.9, 55.0, 48.1, 45.4, 43.7, 43.3, 41.8, 39.3, 39.2, 38.5, 37.5, 37.1, 32.8, 32.0, 31.5, 29.5, 28.5, 28.2, 27.3, 26.5, 26.4, 23.4, 18.75, 17.6, 16.5, 15.7; HRMS (ESI): m/z calcd for $C_{37}H_{51}O_3NCl^-$: 592.3552; found: 592.3563.

N-(3-phenylpropyl)-3β-hydroxy-11-oxo-olean-12-en-30-amide (A_{16})

white solid, yield 93.4 %, m. p. 124.2-125.9 °C; ^{1}H NMR (400 MHz, $CDCl_3$) δ 7.29 (dd, *J* = 15.3, 7.7 Hz, 2H, phenyl-H), 7.18 (t, *J* = 5.8 Hz, 3H, phenyl-H), 6.18 (s, 1H, CONH), 5.66 (s, 1H, 12-CH=C), 3.37–3.26 (m, 2H, 1′-NH$\underline{CH_2}$), 3.22 (dd, *J* = 10.9, 5.1 Hz, 1H, 3-$\underline{CH}$OH), 2.78 (d, *J* = 13.4 Hz, 1H, 1a-H), 2.64 (t, *J* = 7.6 Hz, 2H, 3′-$NHCH_2CH_2\underline{CH_2}$), 2.34 (s, 1H, 9-H), 2.16 (dd, *J* = 13.0, 2.9 Hz, 1H, 18-H), 2.09–1.99 (m, 1H, 16a-H), 1.91 (d, *J* = 11.6 Hz, 1H, 19a-H), 1.86–1.76 (m, 4H, 21a-H+15a-H+3′-$NHCH_2\underline{CH_2}CH_2$), 1.73–1.59 (m, 5H, 6a-H+2-H+7a-H+21b-H), 1.48–1.33 (m, 5H, 6b-H+7b-H+19b-H+22-H), 1.37 (s, 3H, 27-CH_3), 1.20–0.95 (m, 3H, 15b-H+16b-H+1b-H), 1.13 (s, 3H, 29-CH_3), 1.11 (s, 6H, 25-CH_3+26-CH_3), 1.01 (s, 3H, 23-CH_3), 0.81 (s, 6H, 24-CH_3+28-CH_3), 0.70 (d, *J* = 11.5 Hz, 1H, 5-H); ^{13}C NMR (101 MHz, $CDCl_3$) δ 200.3, 175.8, 169.7, 141.6, 128.5, 128.4, 126.0, 78.6, 61.8,

54.9, 50.5, 48.2, 45.4, 43.6, 43.2, 41.5, 39.3, 39.2, 39.2, 37.5, 37.0, 33.52, 32.7, 31.9, 31.4, 31.4, 29.6, 28.6, 28.2, 27.2, 26.4, 26.4, 23.4, 18.6, 17.5, 16.4, 15.7; HRMS (ESI): m/z calcd for $C_{39}H_{57}O_3NNa$: 610.4231; found: 610.4212.

1-(Boc-piperidine)-3*β*-acetyloxy-11-oxo-olean-12-en-30-amide (A_{17})

white solid, yield 97.4 %, m. p. 123.6-124.7 °C; ^{1}H NMR (500 MHz, $CDCl_3$) *δ* 5.60 (s, 1H, CONH), 5.47 (d, *J* = 8.1 Hz, 1H, 12-CH=C), 4.50 (dd, *J* = 11.7, 4.7 Hz, 1H, 3-CHOOCCH3), 4.07 (d, *J* = 12.7 Hz, 2H, 3′a-NCH2), 3.94 (dd, *J* = 11.2, 7.3 Hz, 1H, 3′a-NHCH), 2.80 (d, *J* = 19.2 Hz, 2H, 3′b-NCH2), 2.76 (d, *J* = 3.2 Hz, 1H, 1a-H), 2.34 (s, 1H, 9-H), 2.15–2.09 (m, 1H, 18-H), 2.04 (s, 3H, 3-CHOOCCH3), 1.86 (dd, *J* = 19.4, 4.3 Hz, 4H, 16a-H+19a-H+2′a-NHCHCH2), 1.80 (dd, *J* = 13.6, 4.3 Hz, 1H, 21a-H), 1.73 (s, 1H, 15a-H), 1.71–1.54 (m, 7H, 6a-H+2-H+7a-H+21b-H+2′b-NHCHCH2), 1.44 (s, 9H, 5′-OC(CH3)3), 1.36 (d, *J* = 10.2 Hz, 5H, 6b-H+7b-H+19b-H+22-H), 1.35 (s, 3H, 27-CH3), 1.17–1.02 (m, 3H, 15b-H+16b-H+1b-H), 1.14 (s, 3H, 29-CH3), 1.10 (s, 6H, 25-CH3+26-CH3), 0.86 (s, 6H, 23-CH3+24-CH3), 0.79 (s, 3H, 28-CH3), 0.77 (d, *J* = 7.7 Hz, 1H, 5-H); ^{13}C NMR (101 MHz, $CDCl_3$) *δ* 200.0, 175.1, 171.1, 169.3, 154.7, 128.4, 80.6, 79.7, 61.8, 55.0, 48.3, 46.6, 45.4, 43.4, 43.2, 41.8, 38.8, 38.0, 37.5, 37.0, 32.7, 31.9, 31.5, 29.7, 29.5, 28.6, 28.4, 28.1, 26.5, 26.4, 23.6, 23.3, 21.3, 18.7, 17.4, 16.7, 16.4; HRMS (ESI): m/z calcd for $C_{43}H_{69}O_6N_2^+$: 709.5150; found: 709.5137.

1-((Boc-4-methy)-piperidine)-3*β*-acetyloxy-11-oxo-olean-12-en-30-amide (A_{18})

white solid, yield 96.8 %, m. p. 102.9-104.7 °C; ^{1}H NMR (400 MHz, $CDCl_3$) *δ* 5.76 (t, *J* = 5.9 Hz, 1H, CONH), 5.63 (s, 1H, 12-CH=C), 4.50 (dd, *J* = 11.6, 4.8 Hz, 1H,

3-$\underline{CH}OOCCH_3$), 4.08 (s, 2H, 4′a-NCH_2), 3.47 (s, 6H, 1′-$NH\underline{CH_2}$+1a-H+4′b-NCH_2), 3.16 (s, 2H, 3′b-$NCH_2\underline{CH_2}$), 2.78 (dt, *J* = 13.2, 3.2 Hz, 1H, 1a-H), 2.34 (s, 1H, 9-H), 2.04 (s, 3H, 3-$CHOOC\underline{CH_3}$), 1.90 (d, *J* = 6.6 Hz, 1H, 18-H), 1.86–1.77 (m, 2H, 16a-H+19a-H), 1.74 (s, 1H, 21a-H), 1.65–1.56 (m, 6H, 6a-H+2-H+7a-H+15a-H+21b-H), 1.44–1.35 (m, 14H, 6b-H+7b-H+19b-H+22-H +6′-$OC(CH_3)_3$), 1.36 (s, 3H, 27-CH_3), 1.20–1.00 (m, 3H, 15b-H+16b-H+1b-H), 1.15 (s, 3H, 29-CH_3), 1.12 (s, 3H, 25-CH_3), 1.11 (s, 3H, 26-CH_3), 0.87 (s, 6H, 23-CH_3+24-CH_3), 0.79 (s, 3H, 28-CH_3), 0.78 (s, 1H, 5-H); ^{13}C NMR (101 MHz, $CDCl_3$) δ 200.0, 175.9, 171.0, 169.3, 154.8, 128.4, 80.6, 79.4, 61.7, 55.0, 48.2, 45.4, 45.0, 43.7, 43.2, 41.8, 38.8, 38.0, 37.5, 36.9, 36.6, 32.7, 31.9, 31.5, 29.9, 29.7, 28.5, 28.4, 28.0, 26.4, 26.4, 23.5, 23.3, 21.3, 18.7, 17.4, 16.7, 16.4; HRMS (ESI): m/z calcd for $C_{42}H_{67}O_6N_2^+$: 695.4994; found: 695.4980.

***N*-(3-(piperidin-1-yl)propyl)-3*β*-acetyloxy-11-oxo-olean-12-en-30-amide (A_{19})**

white solid, yield 97.1 %, m. p. 122.4-124.1 °C; ^1H NMR (400 MHz, $CDCl_3$) δ 7.66 (d, *J* = 4.1 Hz, 1H, CONH), 5.64 (s, 1H, 12-CH=C), 4.49 (dd, *J* = 11.6, 4.8 Hz, 1H, 3-$\underline{CH}OOCCH_3$), 3.42 (td, *J* = 11.6, 5.1 Hz, 1H, 1a′-$NH\underline{CH_2}$), 3.31–3.23 (m, 1H, 1b′-$NH\underline{CH_2}$), 2.76 (dt, *J* = 13.4, 3.3 Hz, 1H, 1a-H), 2.51–2.34 (m, 6H, 3′-$NHCH_2CH_2\underline{CH_2}$+4′-$NCH_2$), 2.32 (s, 1H, 9-H), 2.13 (dd, *J* = 13.3, 2.8 Hz, 1H, 18-H), 2.07–1.94 (m, 5H, 3-$CHOOC\underline{CH_3}$+16a-H+19a-H), 1.90–1.84 (m, 1H, 21a-H), 1.80 (dd, *J* = 13.6, 4.4 Hz, 1H, 15a-H), 1.75–1.45 (m, 13H, 6a-H+2-H+7a-H+21b-H+2′-$NHCH_2\underline{CH_2}CH_2$+5′-$NCH_2\underline{CH_2}$+6′-$NCH_2CH_2\underline{CH_2}$), 1.45–1.34 (m, 5H, 6b-H+7b-H+19b-H+22-H), 1.35 (s, 3H, 27-CH_3), 1.19–0.90 (s, 3H,

15b-H+16b-H+1b-H), 1.14 (s, 3H, 29-CH_3), 1.11 (s, 6H, 25-CH_3+26-CH_3), 0.86 (s, 6H, 23-CH_3+24-CH_3), 0.78 (d, J = 7.8 Hz, 4H, 28-CH_3+5-H). ^{13}C NMR (101 MHz, $CDCl_3$) δ 199.8, 175.9, 171.1, 169.2, 128.3, 80.7, 61.7, 59.2, 55.0, 54.9, 48.4, 45.4, 43.4, 43.2, 41.8, 40.5, 38.8, 38.0, 37.4, 36.9, 32.7, 31.9, 31.4, 29.7, 28.4, 28.0, 26.6, 26.4, 25.8, 24.3, 24.3, 23.5, 23.2, 21.3, 18.7, 17.4, 16.7, 16.4; HRMS (ESI): m/z calcd for $C_{40}H_{65}O_4N_2^+$: 637.4939; found: 637.4930.

***N*-(3-(4-methylpiperazin-1-yl)propyl)-3*β*-acetyloxy-11-oxo-olean-12-en-30-amide (A_{20})**

white solid, yield 84.8 %, m. p. 122.6-123.9 °C; ^{1}H NMR (400 MHz, $CDCl_3$) δ 7.32 (t, J = 4.2 Hz, 1H, CONH), 5.61 (s, 1H, 12-CH=C), 4.51 (dd, J = 11.7, 4.8 Hz, 1H, 3-$\underline{CH}$OOCCH_3), 3.49–3.39 (m, 1H, 1a′-NH$\underline{CH_2}$), 3.32–3.22 (m, 1H, 1b′-NH$\underline{CH_2}$), 2.78 (dt, J = 13.4, 3.3 Hz, 1H, 1a-H), 2.67–2.35 (m, 8H, 4′-N$\underline{CH_2}$+5′-N$\underline{CH_2}$), 2.32 (s, 1H, 9-H), 2.26 (s, 3H, 3-CHOOC$\underline{CH_3}$), 2.24–2.17(m, 2H, 3′-NH$CH_2CH_2\underline{CH_2}$), 2.13 (dd, J = 13.3, 3.0 Hz, 1H, 18-H), 2.06–1.96 (m, 5H, 16a-H+19a-H+6′-NCH_3), 1.86 (dd, J = 10.3, 3.2 Hz, 1H, 21a-H), 1.77–1.72 (m, 1H, 15a-H), 1.72–1.54(m, 7H, 6a-H+2-H+7a-H+21b-H+2′-NH$CH_2\underline{CH_2}CH_2$), 1.47–1.30 (m, 5H, 6b-H+7b-H+19b-H+22-H), 1.37 (s, 3H, 27-CH_3), 1.20–1.00 (m, 3H, 15b-H+16b-H+1b-H), 1.14 (s, 3H, 29-CH_3), 1.11 (s, 3H, 25-CH_3), 1.10 (s, 3H, 26-CH_3), 0.87 (s, 6H, 23-CH_3+24-CH_3), 0.79 (s, 3H, 28-CH_3), 0.77 (s, 1H, 5-H); ^{13}C NMR (101 MHz, $CDCl_3$) δ 199.8, 175.8, 171.1, 169.2, 128.4, 80.6, 61.8, 58.5, 55.0, 55.0, 53.4, 48.4, 46.1, 45.4, 43.5, 43.1, 41.7, 40.4, 38.8, 38.1, 37.4, 36.9, 32.7, 31.9, 31.4, 29.7, 28.4, 28.1, 26.5, 26.4, 24.4, 23.6, 23.1, 21.4, 18.7, 17.4, 16.7, 16.4; HRMS

(ESI): m/z calcd for $C_{40}H_{66}O_4N_3^+$: 652.5048; found: 652.5035.

1-((Boc-4-propyl)-piperazine)-3*β*-acetyloxy-11-oxo-olean-12-en-30-amide (A_{21})

white solid, yield 97.3 %, m. p. 136.6-138.1 °C; ^{1}H NMR (400 MHz, $CDCl_3$) δ 6.96 (t, J = 4.6 Hz, 1H, CONH), 5.62 (s, 1H, 12-CH=C), 4.48 (dd, J = 11.6, 4.8 Hz, 1H, 3-$\underline{CH}OOCCH_3$), 3.48–3.24 (m, 6H, 1′-NH$\underline{CH_2}$+5′-N$\underline{CH_2}$), 2.78 (dd, J = 10.2, 3.4 Hz, 1H, 1a-H), 2.52–2.34 (m, 6H, 3′-$NCH_2CH_2\underline{CH_2}$+4′-N$\underline{CH_2}$), 2.32 (s, 1H, 9-H), 2.13 (dd, J = 13.2, 3.2 Hz, 1H, 18-H), 2.02 (s, 5H, 16a-H+19a-H+3-CHOOC$\underline{CH_3}$), 1.90 (d, J = 10.9 Hz, 1H, 21a-H), 1.80 (d, J = 13.3 Hz, 1H, 15a-H), 1.72–1.55 (m, 7H, 6a-H+2-H+7a-H+21b-H+2′-NHCH$\underline{CH_2}$), 1.44 (s, 9H, 7′-$OC(CH_3)_3$), 1.42–1.32 (m, 5H, 6b-H+7b-H+19b-H+22-H), 1.34 (s, 3H, 27-CH_3), 1.19–1.01 (m, 3H, 15b-H+16b-H+1b-H), 1.13 (s, 3H, 29-CH_3), 1.10 (s, 3H, 25-CH_3), 1.09 (s, 3H, 26-CH_3), 0.86 (s, 6H, 23-CH_3+24-CH_3), 0.79 (s, 3H, 28-CH_3), 0.76 (s, 1H, 5-H); ^{13}C NMR (101 MHz, $CDCl_3$) δ 199.8, 175.7, 170.9, 169.3, 154.7, 128.3, 80.6, 79.8, 61.8, 58.0, 55.1, 53.3, 48.3, 45.4, 43.5, 43.2, 41.9, 39.7, 38.9, 38.0, 37.4, 36.9, 32.7, 31.9, 31.5, 29.7, 28.5, 28.4, 28.1, 26.5, 26.4, 25.1, 23.5, 23.3, 21.3, 18.7, 17.4, 16.7, 16.4; HRMS (ESI): m/z calcd for $C_{44}H_{72}O_6N_3^+$: 738.5416; found: 738.5407.

***N*-(piperidin-4-yl)-3*β*-hydroxy-11-oxo-olean-12-en-30-amide (A_{22})**

white solid, yield 94.61 %, m. p. 132.2-133.6 °C; ^{1}H NMR (400 MHz, $CDCl_3$) δ 5.68 (d, J = 7.0 Hz, 1H, CONH), 5.59 (s, 1H, 12-CH=C), 3.95–3.83 (m, 1H, 1′-$\underline{CH}CH_2$), 3.17 (dd, J = 10.9, 5.3 Hz, 1H, 3-$\underline{CH}$OH), 3.09–3.00 (m, 2H, 3′a-$CHCH_2\underline{CH_2}$), 2.77–2.62 (m, 3H, 3′b-$CHCH_2\underline{CH_2}$+1a-H), 2.30 (s, 1H, 9-H), 2.14 (s, 3H, 2′-CH$\underline{CH_2}$+2′a-CH$\underline{CH_2}$), 2.10 (d, J = 4.8 Hz, 1H, 18-H), 2.06–1.98 (m, 1H, 4′-NH),

1.92–1.83 (m, 3H, 16a-H+19a-H+2′b-CH$\underline{CH_2}$), 1.79 (dd, *J* = 15.0, 4.5 Hz, 1H, 21a-H), 1.72 (s, 1H, 15a-H), 1.69–1.52 (m, 5H, 6a-H+2-H+7a-H+21b-H), 1.42–1.32 (m, 5H, 6b-H+7b-H+19b-H+22-H), (1.35, 1H, 27-CH_3), 1.17–0.86 (m, 3H, 15b-H+16b-H+1b-H), 1.10 (s, 3H, 29-CH_3), 1.09 (s, 6H, 25-CH_3+26-CH_3), 0.97 (s, 3H, 23-CH_3), 0.78 (s, 3H, 24-CH_3), 0.77 (s, 3H, 28-CH_3), 0.65 (d, *J* = 11.6 Hz, 1H, 5-H); ^{13}C NMR (101 MHz, $CDCl_3$) δ 200.1, 174.9, 169.3, 128.4, 78.7, 61.9, 55.0, 48.2, 46.5, 45.4, 43.4, 43.2, 41.8, 39.3, 39.2, 37.5, 37.1, 33.4, 33.2, 32.8, 31.9, 31.5, 29.5, 28.6, 28.2, 27.3, 26.5, 26.4, 23.4, 18.7, 17.5, 16.4, 15.7; HRMS (ESI): m/z calcd for $C_{35}H_{55}O_3N_2^-$: 551.4207; found: 551.4221.

***N*-(piperidin-4-ylmethyl)-3*β*-hydroxy-11-oxo-olean-12-en-30-amide (A_{23})**

white solid, yield 75.1 %, m. p. 137.2-138.6 °C; ^{1}H NMR (400 MHz, $CDCl_3$) δ 5.77 (t, *J* = 5.8 Hz, 1H, CONH), 5.63 (s, 1H, 12-CH=C), 3.20 (dd, *J* = 10.8, 5.5 Hz, 1H, 3-$\underline{CH}$OH), 3.16–3.12 (m, 2H, 1′-NH$\underline{CH_2}$CH), 3.09 (d, *J* = 12.4 Hz, 2H, 4′a-NH$\underline{CH_2}$$CH_2$), 2.79–2.74 (m, 1H, 1a-H), 2.58 (t, *J* = 11.9 Hz, 2H, 4′b-NH$\underline{CH_2}$$CH_2$), 2.32 (s, 1H, 9-H), 2.15 (dd, *J* = 12.4, 5.1 Hz, 1H, 18-H), 2.07–1.96 (m, 2H, 16a-H+19a-H), 1.92 (d, *J* = 7.5 Hz, 2H, 2′-NH$CH_2$$\underline{CH}$+5′-NH), 1.85–1.78 (m, 1H, 21a-H), 1.76–1.73 (m, 1H, 15a-H), 1.68–1.56 (m, 9H, 6a-H+2-H+7a-H+21b-H+3′-NH$CH_2$$\underline{CH_2}$), 1.42–1.35 (m, 5H, 6b-H+7b-H+19b-H+22-H), 1.38 (s, 1H, 27-CH_3), 1.21–1.16 (m, 3H, 15b-H+16b-H+1b-H), 1.13–1.11 (m, 9H, 25-CH_3+26-CH_3+29-CH_3), 0.99 (s, 3H, 23-CH_3), 0.80 (s, 3H, 24-CH_3), 0.79 (s, 3H, 28-CH_3), 0.68 (d, *J* = 12.0 Hz, 1H, 5-H); ^{13}C NMR (101 MHz, $CDCl_3$) δ 200.3, 175.9, 169.5, 128.5, 78.7, 61.9, 54.9, 48.2, 45.9,

45.4, 43.7, 43.3, 41.8, 39.2, 39.2, 37.5, 37.1, 36.4, 32.8, 31.9, 31.5, 30.7, 29.8, 29.7, 28.6, 28.1, 27.3, 26.5, 26.4, 23.4, 18.7, 17.5, 16.4, 15.6; HRMS (ESI): m/z calcd for $C_{36}H_{57}O_3N_2^-$: 565.4364; found: 565.4379.

***N*-(3-(piperazin-1-yl)propyl)-3*β*-hydroxy-11-oxo-olean-12-en-30-amide (A_{24})**

white solid, yield 42.11 %, m. p. 116.7-118.3 °C; ^{1}H NMR (400 MHz, $CDCl_3$) *δ* 7.30 (t, *J* = 4.5 Hz, 1H, CONH), 5.62 (s, 1H, 12-CH=C), 3.47–3.38 (m, 1H, 1′a-NH<u>CH2</u>), 3.33–3.24 (m, 1H, 1′b-NH<u>CH2</u>), 3.20 (dd, *J* = 10.9, 5.3 Hz, 1H, 3-<u>CH</u>OH), 2.86 (s, 4H, 5′-NCH_2), 2.75 (dt, *J* = 13.2, 3.3 Hz, 1H, 1a-H), 2.55–2.34 (m, 6H, 3′-$NHCH_2CH_2$<u>CH2</u>+4′-NCH_2), 2.31 (s, 1H, 9-H), 2.13 (dd, *J* = 13.2, 3.2 Hz, 1H, 18-H), 2.08–1.98 (m, 2H, 16a-H+19a-H), 1.95 (s, 1H, 21a-H), 1.86–1.81 (m, 1H, 15a-H), 1.74–1.57 (m, 7H, 6a-H+2-H+7a-H+21b-H+2′-$NHCH_2$<u>CH2</u>CH_2), 1.48–1.33 (m, 5H, 6b-H+7b-H+19b-H+22-H), 1.36 (s, 3H, 27-CH_3), 1.18–0.90 (m, 3H, 15b-H+16b-H+1b-H), 1.11 (s, 3H, 29-CH_3), 1.10 (s, 3H, 25-CH_3), 1.10 (s, 3H, 26-CH_3), 0.98 (s, 3H, 23-CH_3), 0.79 (s, 3H, 24-CH_3), 0.79 (s, 3H, 28-CH_3), 0.67 (d, *J* = 11.6 Hz, 1H, 5-H); ^{13}C NMR (101 MHz, $CDCl_3$) *δ* 200.0, 175.8, 169.4, 128.3, 78.7, 61.9, 59.1, 55.0, 54.9, 48.3, 45.9, 45.4, 43.5, 43.2, 41.9, 40.2, 39.2, 39.2, 37.4, 37.1, 32.8, 31.9, 31.5, 29.7, 28.5, 28.1, 27.3, 26.5, 26.4, 24.3, 23.4, 18.7, 17.5, 16.4, 15.6; HRMS (ESI): m/z calcd for $C_{37}H_{60}O_3N_3^-$: 594.4629; found: 594.4648.

***N*-(piperidin-4-yl)-3*β*-acetyloxy-11-oxo-olean-12-en-30-amide (A_{25})**

white solid, yield 77.4 %, m. p. 114.1-115.5 °C; ^{1}H NMR (500 MHz, $CDCl_3$) *δ* 5.64 (d, *J* = 8.1 Hz, 1H, CONH), 5.59 (s, 1H, 12-CH=C), 4.48 (dd, *J* = 11.7, 4.7 Hz, 1H, 3-<u>CH</u>$OOCCH_3$), 3.93–3.84 (m, 1H, 1′-NH<u>CH</u>), 3.06 (d, *J* = 12.6 Hz, 2H,

3′a-$NHCH_2\underline{CH_2}$), 2.77–2.65 (m, 3H, 1a-H+3′b-$NHCH_2\underline{CH_2}$), 2.50 (s, 1H, 4′-NH), 2.32 (s, 1H, 9-H), 2.12 (dd, *J* = 13.5, 4.1 Hz, 1H, 18-H), 2.02 (s, 3H, 3-$CHOOC\underline{CH_3}$), 1.99 (dd, *J* = 8.1, 5.6 Hz, 1H, 16a-H), 1.92–1.85 (m, 3H, 19a-H+21a-H+15a-H), 1.78 (ddd, *J* = 14.3, 12.0, 3.5 Hz, 2H, 3′b-$NH\underline{CH_2}$), 1.71–1.53 (m, 5H, 6a-H+2-H+7a-H+21b-H), 1.46–1.38 (m, 2H, 3′a-$NH\underline{CH_2}$), 1.37–1.29 (m, 5H, 6b-H+7b-H+19b-H+22-H), 1.33 (s, 3H, 27-CH_3), 1.18–0.98 (m, 3H, 15b-H+16b-H+1b-H), 1.12 (s, 3H, 29-CH_3), 1.09 (d, *J* = 1.3 Hz, 6H, 25-CH_3+26-CH_3), 0.84 (s, 6H, 23-CH_3+24-CH_3), 0.77 (s, 3H, 28-CH_3), 0.76 (s, 1H, 5-H); ^{13}C NMR (126 MHz, $CDCl_3$) *δ* 200.8, 176.1, 171.2, 170.4, 128.4, 80.6, 61.8, 55.0, 48.2, 45.5, 44.3, 43.8, 43.6, 43.4, 41.4, 38.9, 38.1, 37.7, 37.0, 32.7, 31.9, 31.4, 29.8, 29.3, 28.8, 28.6, 28.1, 26.5, 23.6, 23.4, 21.5, 18.8, 17.4, 16.8, 16.6; HRMS (ESI): m/z calcd for $C_{37}H_{57}O_4N_2^-$: 593.4313; found: 593.4330.

***N*-(piperidin-4-ylmethyl)-3*β*-acetyloxy-11-oxo-olean-12-en-30-amide (A_{26})**

white solid, yield 76.4 %, m. p. 130.4-131.1 °C; ^{1}H NMR (500 MHz, $CDCl_3$) *δ* 6.95 (s, 1H, CONH), 5.62 (s, 1H, 12-CH=C), 3.60–3.56 (m, 1H, 3-$\underline{CH}OOCCH_3$), 3.43 (ddd, *J* = 17.0, 11.7, 5.2 Hz, 1H, 1a′-$NH\underline{CH_2}$), 3.24 (ddd, *J* = 10.9, 9.8, 4.6 Hz, 1H, 1b′-$NH\underline{CH_2}$), 3.11 (dd, *J* = 11.2, 4.9 Hz, 1H, 5′-NH), 2.85–2.69 (m, 3H, 1a-H+4′a-$NHCH_2\underline{CH_2}$), 2.46 (dd, *J* = 8.8, 5.6 Hz, 2H, 4′b-$NHCH_2\underline{CH_2}$), 2.30 (s, 1H, 9-H), 2.26 (s, 4H, 3-$CHOOC\underline{CH_3}$+2′-$NHCH_2\underline{CH}$), 2.09 (dd, *J* = 13.8, 3.6 Hz, 1H, 18-H), 2.03 (d, *J* = 3.8 Hz, 2H, 16a-H+19a-H), 2.00 (d, *J* = 3.4 Hz, 1H, 21a-H), 1.85–1.78 (m, 1H, 15a-H), 1.77–1.66 (m, 2H, 3′a-$NHCH_2CH\underline{CH_2}$), 1.65–1.52 (m, 5H, 6a-H+2-H+7a-H+21b-H), 1.44 (t, *J* = 7.3 Hz, 2H, 3′b-$NHCH_2CH\underline{CH_2}$), 1.42–1.32 (m,

5H, 6b-H+7b-H+19b-H+22-H), 1.36 (s, 3H, 27-CH_3), 1.19–0.84 (m, 3H, 15b-H+16b-H+1b-H), 1.11 (s, 3H, 29-CH_3), 1.10 (s, 6H, 25-CH_3+26-CH_3), 0.96 (s, 3H, 23-CH_3), 0.78 (s, 3H, 24-CH_3), 0.76 (s, 3H, 28-CH_3), 0.62 (d, J = 11.6 Hz, 1H, 5-H); ^{13}C NMR (126 MHz, $CDCl_3$) δ 200.1, 176.3, 169.6, 128.3, 78.6, 62.0, 58.1, 55.2, 52.9, 48.5, 45.6, 45.0, 43.7, 43.2, 41.6, 39.8, 39.3, 37.6, 37.1, 36.5, 32.9, 32.0, 31.4, 29.5, 28.6, 28.3, 27.2, 26.6, 26.4, 23.5, 18.7, 17.5, 16.5, 15.7; HRMS (ESI): m/z calcd for $C_{38}H_{60}O_4N_2Cl$: 643.4236; found: 643.4194.

***N*-(3-(piperazin-1-yl)propyl)-3*β*-acetyloxy-11-oxo-olean-12-en-30-amide (A_{27})**

white solid, yield 32.9 %, m. p. 128.6-129.5 °C; ^{1}H NMR (400 MHz, $CDCl_3$) δ 6.95 (s, 1H, CONH), 5.63 (s, 1H, 12-CH=C), 3.85 (s, 1H, 3-$\underline{CH}OOCCH_3$), 3.59 (s, 3H, 1′-NH$\underline{CH_2}$+3a′-$NHCH_2CH_2\underline{CH_2}$), 3.44–3.34 (m, 2H, 4a′-N$\underline{CH_2}$), 3.22 (dd, J = 10.7, 5.5 Hz, 1H, 3b′-$NHCH_2CH_2\underline{CH_2}$), 2.80–2.74 (m, 1H, 1a-H), 2.62 (s, 4H, 5′-NH$\underline{CH_2}$), 2.32 (s, 1H, 9-H), 2.16–2.11 (m, 1H, 18-H), 2.09 (s, 3H, 3-CHOOC$\underline{CH_3}$), 2.05–1.98 (m, 2H, 16a-H+19a-H), 1.95 (d, J = 10.5 Hz, 1H, 21a-H), 1.79 (dd, J = 21.2, 17.8 Hz, 5H, 15a-H+4b′-N$\underline{CH_2}$+2′-$NHCH_2\underline{CH_2}CH_2$), 1.62 (dd, J = 20.7, 10.1 Hz, 5H, 6a-H+2-H+7a-H+21b-H), 1.44–1.35 (m, 5H, 6b-H+7b-H+19b-H+22-H), 1.36 (s, 3H, 27-CH_3), 1.19–.94 (m, 4H, 15b-H+16b-H+1b-H+5′-$\underline{NH}CH_2$), 1.12 (d, J = 1.9 Hz, 9H, 25-CH_3+26-CH_3+29-CH_3), 0.99 (s, 3H, 23-CH_3), 0.80 (d, J = 2.0 Hz, 6H, 24-CH_3+28-CH_3), 0.69 (d, J = 10.9 Hz, 1H, 5-H); ^{13}C NMR (101 MHz, $CDCl_3$) δ 200.2, 169.6, 169.0, 128.4, 78.7, 61.9, 54.9, 53.2, 52.6, 48.3, 45.4, 43.6, 43.2, 41.9, 39.2, 39.2, 37.5, 37.1, 32.7, 31.9, 31.4, 29.7, 28.6, 28.1, 27.3, 26.5, 26.4, 23.4, 21.3, 18.7, 17.5, 16.4, 15.6; HRMS (ESI): m/z calcd for $C_{39}H_{62}O_4N_3^-$: 636.4735; found:

636.4752.

***N*-(2-(dimethylamino)ethyl)-3*β*-hydroxy-urs-12-en-28-amide (B_1)**

White solid, yield 77.5%, m. p. 133.2-135.1 °C; ^{1}H NMR (400 MHz, $CDCl_3$) *δ* 6.54–6.48 (m, 1H, CONH), 5.27 (t, *J* = 3.4 Hz, 1H, 12-CH=C), 3.36–3.27 (m, 1H, 1′a-NH$\underline{CH_2}$), 3.21 (dd, *J* = 10.8, 4.4 Hz, 1H, 3-$\underline{CH}$OH), 3.17–3.08 (m, 1H, 1′b-NH$\underline{CH_2}$), 2.39–2.32 (m, 2H, 2′-NH$CH_2\underline{CH_2}$), 2.21 (d, *J* = 8.1 Hz, 7H, 18-H+3′-N$(CH_3)_2$), 1.94–1.84 (m, 4H, 11-H+2-H), 1.74–1.44 (m, 11H, 16-H+9-H+22-H+20-H+6-H+1-H+19-H), 1.41–1.23 (m, 4H, 21-H+7-H), 1.10–1.00 (m, 5H, 15-H+27-CH_3), 0.97 (s, 3H, 23-CH_3), 0.94 (s, 3H, 30-CH_3), 0.90 (s, 3H, 25-CH_3), 0.86 (d, *J* = 6.5 Hz, 3H, 29-CH_3), 0.78 (s, 3H, 26-CH_3), 0.77 (s, 3H, 24-CH_3), 0.71 (d, *J* = 12.6 Hz, 1H, 5-H); ^{13}C NMR (101 MHz, $CDCl_3$) *δ* 178.2, 139.3, 125.8, 79.0, 57.6, 55.2, 53.9, 47.7, 47.5, 45.3, 42.4, 39.8, 39.6, 39.1, 38.8, 38.6, 37.2, 36.9, 36.8, 32.9, 31.0, 28.2, 27.9, 27.2, 24.8, 23.4, 23.2, 21.3, 18.3, 17.2, 17.0, 15.7, 15.6; HRMS (ESI): m/z calcd for $C_{34}H_{59}O_2N_2^+$: 527.4571; found: 527.4566.

1-((Boc)-piperidine)-3*β*-hydroxy-urs-12-en-28-amide (B_2)

White solid, yield 45.2%, m. p. 138.2-139.1 °C; ^{1}H NMR (400 MHz, $CDCl_3$) *δ* 5.68 (d, *J* = 7.2 Hz, 1H, CONH), 5.27 (t, *J* = 3.3 Hz, 1H, 12-CH=C), 4.06–3.77 (m, 3H, 1′-NH$\underline{CH}$+3′a-NCH_2), 3.21 (dd, *J* = 10.9, 4.7 Hz, 1H, 3-$\underline{CH}$OH), 2.85 (t, *J* = 11.7 Hz, 2H, 3′b-NCH_2), 2.05–1.75 (m, 9H, 18-H+11-H+2-H+2′-N$CH_2\underline{CH_2}$), 1.71–1.49 (m, 11H, 16-H+9-H+22-H+20-H+6-H+1-H+19-H), 1.45 (s, 9H, 5′-OC$(CH_3)_3$), 1.32 (dd, *J* = 18.3, 13.1 Hz, 4H, 21-H+7-H), 1.09 (s, 3H, 27-CH_3), 1.02 (dd, *J* = 15.7, 5.0 Hz, 2H, 15-H), 0.98 (s, 3H, 23-CH_3), 0.94 (s, 3H, 30-CH_3), 0.92 (s, 3H, 25-CH_3), 0.85 (d, *J* =

6.4 Hz, 3H, 29-CH_3), 0.80 (s, 3H, 26-CH_3), 0.77 (s, 3H, 24-CH_3), 0.71 (d, J = 11.1 Hz, 1H, 5-H); ^{13}C NMR (101 MHz, $CDCl_3$) δ 177.4, 154.8, 139.6, 125.6, 79.7, 79.0, 55.1, 54.0, 47.8, 47.5, 46.5, 42.7, 39.8, 39.6, 39.1, 38.8, 38.7, 37.6, 37.0, 33.0, 32.1, 31.6, 30.9, 28.5, 28.2, 27.9, 27.2, 24.7, 23.4, 23.2, 21.2, 18.3, 17.5, 17.2, 15.7, 15.6; HRMS (ESI): m/z calcd for $C_{40}H_{65}O_4N_2^-$: 637.4939; found: 637.4958.

1-((Boc-4-methy)-piperidine)-3*β*-hydroxy-urs-12-en-28-amide (B$_3$)

White solid, yield 50.0%, m. p. 138.2-139.1 °C; ^{1}H NMR (500 MHz,) δ 6.00 (t, J = 5.3 Hz, 1H, CONH), 5.29 (t, J = 3.4 Hz, 1H, 12-CH=C), 4.08 (d, J = 14.2 Hz, 2H, 4′a-NCH_2), 3.33 (s, 1H, 1′a-NH$\underline{CH_2}$), 3.20 (dd, J = 11.2, 4.3 Hz, 1H, 3-$\underline{CH}$OH), 2.65 (s, 3H, 1′b-NH$\underline{CH_2}$+4′b-NCH_2), 1.97 (dd, J = 13.2, 4.3 Hz, 1H, 18-H), 1.93–1.82 (m, 4H, 11-H+2-H), 1.75–1.47 (m, 15H, 16-H+9-H+22-H+20-H+6-H+1-H+19-H+3′-NC $H_2\underline{CH_2}$), 1.43 (s, 9H, 6′-OC$(CH_3)_3$), 1.33 (ddd, J = 29.6, 12.9, 3.5 Hz, 4H, 21-H+7-H), 1.08 (s, 3H, 27-CH_3), 1.07–1.02 (m, 2H, 15-H), 0.97 (s, 3H, 23-CH_3), 0.94 (s, 3H, 30-CH_3), 0.90 (s, 3H, 25-CH_3), 0.86 (d, J = 6.5 Hz, 3H, 29-CH_3), 0.77 (s, 3H, 26-CH_3), 0.74 (s, 3H, 24-CH_3), 0.71 (d, J = 10.5 Hz, 1H, 5-H); ^{13}C NMR (126 MHz, $CDCl_3$) δ 178.3, 154.9, 140.2, 125.6, 79.5, 79.0, 55.2, 54.1, 48.0, 47.6, 44.8, 42.6, 39.8, 39.6, 39.2, 38.8, 38.7, 37.5, 37.0, 36.3, 32.8, 31.0, 30.0, 29.8, 28.5, 28.2, 27.9, 27.2, 25.0, 23.5, 23.3, 21.3, 18.3, 17.3, 17.1, 15.7, 15.6; HRMS (ESI): m/z calcd for $C_{41}H_{67}O_4N_2^-$: 651.5095; found: 651.5111.

***N*-(3-(4-methylpiperazin-1-yl)propyl)-3*β*-hydroxy-urs-12-en-28-amide (B$_4$)**

White solid, yield 82.6%, m. p. 98.8-100.1 °C; ^{1}H NMR (400 MHz, $CDCl_3$) δ 6.47–6.42 (m, 1H, CONH), 5.26 (t, J = 3.3 Hz, 1H, 12-CH=C), 3.46–3.35 (m, 1H,

1′a-NH$\underline{CH_2}$), 3.18 (dd, *J* = 11.0, 4.7 Hz, 1H, 3-$\underline{CH}$OH), 3.01 (ddd, *J* = 13.3, 10.8, 6.6 Hz, 1H, 1′b-NH$\underline{CH_2}$), 2.77–2.34 (m, 10H, 3′-$NHCH_2CH_2\underline{CH_2}$+4′-$NCH_2$+5′-$NCH_2\underline{CH_2}$), 2.28 (s, 3H, 6′-$NCH_3$), 2.23 (s, 1H, 18-H), 1.97–1.88 (m, 4H, 11-H+2-H), 1.81–1.70 (m, 2H, 2′-$NHCH_2\underline{CH_2}CH_2$), 1.67–1.46 (m, 10H, 16-H+9-H+22-H+20-H+6a-H+1-H+19-H), 1.43–1.26 (m, 5H, 6b-H+21-H+7-H), 1.06 (s, 3H, 27-CH_3), 1.04–0.98 (m, 2H, 15-H), 0.95 (s, 3H, 23-CH_3), 0.93 (s, 3H, 30-CH_3), 0.88 (s, 3H, 25-CH_3), 0.85 (d, *J* = 6.4 Hz, 3H, 29-CH_3), 0.75 (d, *J* = 3.3 Hz, 3H, 26-CH_3), 0.73 (s, 3H, 24-CH_3), 0.68 (d, *J* = 4.7 Hz, 1H, 5-H); ^{13}C NMR (101 MHz, $CDCl_3$) δ 177.9, 139.7, 125.5, 78.9, 57.2, 55.1, 55.0, 53.7, 53.3, 47.5, 46.0, 42.4, 39.7, 39.5, 39.1, 38.8, 38.8, 38.6, 37.4, 36.9, 32.8, 30.9, 28.2, 27.8, 27.2, 25.7, 24.7, 23.4, 21.3, 18.3, 17.3, 17.0, 15.7, 15.5; HRMS (ESI): m/z calcd for $C_{38}H_{64}O_2N_3^-$: 594.4993; found: 594.5006.

1-((Boc-4-propyl)-piperazine)-3*β*-hydroxy-urs-12-en-28-amide (B_5)

White solid, yield 88.6%, m. p. 113.4-114.7 °C; ^{1}H NMR (400 MHz, $CDCl_3$) δ 6.32 (t, *J* = 4.9 Hz, 1H, CONH), 5.26 (s, 1H, 12-CH=C), 3.42 (d, *J* = 4.2 Hz, 4H, 5′-NCH_2), 3.36 (dd, *J* = 13.1, 6.7 Hz, 1H, 1a′-NH$\underline{CH_2}$), 3.19 (dd, *J* = 11.1, 4.2 Hz, 1H, 3-$\underline{CH}$OH), 3.09–2.99 (m, 1H, 1b′-NH$\underline{CH_2}$), 2.36 (d, *J* = 5.7 Hz, 6H, 3′-$NHCH_2CH_2\underline{CH_2}$+4′-$NCH_2$), 2.02–1.96 (m, 1H, 18-H), 1.92–1.76 (m, 4H, 11-H+2-H), 1.69–1.48 (m, 12H, 16-H+9-H+22-H+20-H+6a-H+1-H+19-H+2′-$NHCH_2\underline{CH_2}CH_2$), 1.44 (s, 9H, 7′-$OC(CH_3)_3$), 1.30 (dd, *J* = 26.0, 12.1 Hz, 5H, 6b-H+21-H+7-H), 1.07 (s, 3H, 27-CH_3), 1.01 (dd, *J* = 12.1, 8.2 Hz, 2H, 15-H), 0.96 (s, 3H, 23-CH_3), 0.92 (s, 3H,

30-CH_3), 0.89 (s, 3H, 25-CH_3), 0.84 (d, *J* = 6.4 Hz, 3H, 29-CH_3), 0.76 (s, 3H, 26-CH_3), 0.74 (s, 3H, 24-CH_3), 0.70 (d, *J* = 11.5 Hz, 1H, 5-H); ^{13}C NMR (101 MHz, $CDCl_3$) *δ* 177.9, 154.7, 139.7, 125.5, 79.8, 78.9, 56.9, 55.1, 53.7, 53.2, 47.6, 47.5, 42.4, 39.7, 39.5, 39.1, 38.8, 38.6, 38.5, 37.4, 36.9, 32.8, 30.9, 28.4, 28.2, 27.8, 27.2, 25.9, 24.8, 23.4, 23.4, 21.3, 18.3, 17.3, 17.0, 15.7, 15.5; HRMS (ESI): m/z calcd for $C_{42}H_{70}O_4N_3^-$: 680.5361; found: 680.5379.

***N*-(morpholino)-3*β*-hydroxy-urs-12-en-28-amide (B_6)**

White solid, yield 48.6%, m. p. 108.4-110.1 °C; ^{1}H NMR (500 MHz, $CDCl_3$) *δ* 8.24 (s, 1H, CONH), 5.19 (t, *J* = 3.4 Hz, 1H, 12-CH=C), 3.88–3.61 (m, 2H, 2′a-O<u>CH</u>$_2$), 3.30–3.22 (m, 2H, 2′b-O<u>CH</u>$_2$), 3.18 (dd, *J* = 11.0, 4.7 Hz, 1H, 3-<u>CH</u>OH), 2.65–2.42 (m, 2H, 1′a-N<u>CH</u>$_2$), 2.35 (d, *J* = 4.5 Hz, 2H, 1′b-N<u>CH</u>$_2$), 2.13–2.05 (m, 1H, 18-H), 1.87–1.77 (m, 4H, 11-H+2-H), 1.73–1.41 (m, 10H, 16-H+9-H+22-H+20-H+6a-H+1-H+19-H), 1.38–1.23 (m, 5H, 6b-H+21-H+7-H), 1.06 (s, 3H, 27-CH_3), 1.04–0.98 (m, 2H, 15-H), 0.96 (s, 3H, 23-CH_3), 0.91 (d, *J* = 6.4 Hz, 3H, 30-CH_3), 0.88 (s, 3H, 25-CH_3), 0.83 (d, *J* = 6.4 Hz, 3H, 29-CH_3), 0.75 (s, 3H, 26-CH_3), 0.70 (s, 3H, 24-CH_3), 0.67 (s, 1H, 5-H); ^{13}C NMR (126 MHz, $CDCl_3$) *δ* 180.16, 138.57, 125.44, 79.02, 56.43, 55.26, 51.57, 47.57, 44.48, 42.25, 39.76, 39.53, 38.82, 38.65, 38.40, 37.02, 35.76, 34.79, 32.96, 30.75, 28.33, 28.21, 27.26, 25.99, 24.45, 23.66, 23.34, 21.35, 18.33, 17.48, 17.22, 15.74, 15.58, 14.75; HRMS (ESI): m/z calcd for $C_{34}H_{55}O_3N_2^-$: 539.4207; found: 539.4222.

***N*-(benzyl)-3*β*-hydroxy-urs-12-en-28-amide (B_7)**

White solid, yield 45.2%, m. p. 128.7-130.1 °C; ^{1}H NMR (400 MHz, $CDCl_3$) *δ* 7.36–

7.30 (m, 2H, phenyl-H), 7.28 (dd, J = 5.2, 1.9 Hz, 1H, phenyl-H), 7.25 (d, J = 1.5 Hz, 1H, phenyl-H), 7.23 (s, 1H, phenyl-H), 6.16 (t, J = 5.0 Hz, 1H, CONH), 5.21 (t, J = 3.4 Hz, 1H, 12-CH=C), 4.56 (dd, J = 14.5, 6.1 Hz, 1H, 1′a-NH$\underline{CH_2}$), 4.15 (dd, J = 14.5, 4.2 Hz, 1H, 1′b-NH$\underline{CH_2}$), 3.21 (dd, J = 11.1, 4.6 Hz, 1H, 3-$\underline{CH}$OH), 2.03–1.95 (m, 1H, 3-$\underline{CH}$OH), 1.91–1.75 (m, 4H, 11-H+2-H), 1.67–1.46 (m, 11H, 16-H+9-H+22-H+20-H+6-H+1-H+19-H), 1.42–1.25 (m, 4H, 21-H+7-H), 1.06 (d, J = 15.9 Hz, 5H, 15-H+27-CH_3), 0.98 (s, 3H, 23-CH_3), 0.94 (s, 3H, 30-CH_3), 0.88 (s, 3H, 25-CH_3), 0.84 (d, J = 6.5 Hz, 3H, 29-CH_3), 0.78 (s, 3H, 26-CH_3), 0.71 (d, J = 5.9 Hz, 4H, 24-CH_3+5-H); ^{13}C NMR (126 MHz, $CDCl_3$) δ 178.1, 139.9, 138.4, 128.8, 128.0, 127.5, 125.9, 79.0, 55.2, 54.1, 47.8, 47.6, 43.8, 42.6, 39.8, 39.6, 39.2, 38.8, 38.7, 37.3, 37.0, 32.8, 31.0, 28.2, 28.0, 27.2, 25.0, 23.4, 23.3, 21.3, 18.3, 17.3, 17.1, 15.7, 15.56; HRMS (ESI): m/z calcd for $C_{37}H_{54}O_2N^-$: 544.4149; found: 544.4163.

***N*-(piperidin-4-yl)-3*β*-hydroxy-urs-12-en-28-amide (B_8)**

White solid, yield 53.4%, m. p. 148.2-149.9 °C; ^{1}H NMR (400 MHz, $CDCl_3$) δ 5.70 (d, J = 7.4 Hz, 1H, CONH), 5.26 (t, J = 3.3 Hz, 1H, 12-CH=C), 3.85–3.72 (m, 1H, 1′-NH$\underline{CH}$), 3.19 (dd, J = 10.8, 4.8 Hz, 1H, 3-$\underline{CH}$OH), 3.09–2.99 (m, 2H, 3′a-NH$\underline{CH_2}$), 2.72–2.61 (m, 2H, 3′b-NH$\underline{CH_2}$), 2.09 (s, 3H, 18-H+3′a-NHCH$_2\underline{CH_2}$), 1.99–1.80 (m, 7H, 11-H+2-H+3′-$\underline{NH}$CH$_2$+2′a-NCH$_2\underline{CH_2}$), 1.73–1.40 (m, 12H, 16-H+9-H+22-H+20-H+6a-H+1-H+19-H+2′b-NCH$_2\underline{CH_2}$)1.38–1.25 (m, 5H, 6b-H+21-H+7-H), 1.15–1.00 (m, 5H, 15-H+27-CH_3), 0.97 (s, 3H, 23-CH_3), 0.93 (s, 3H, 30-CH_3), 0.90 (s, 3H, 25-CH_3), 0.85 (d, J = 6.4 Hz, 3H, 29-CH_3), 0.80 (s, 3H, 26-CH_3), 0.76 (s, 3H, 24-CH_3), 0.70 (d, J = 11.5 Hz, 1H, 5-H); ^{13}C NMR (101 MHz,

$CDCl_3$) δ 177.2, 139.5, 125.6, 78.9, 55.1, 54.0, 47.7, 47.5, 46.6, 45.3, 45.3, 42.6, 39.8, 39.6, 39.1, 38.8, 38.7, 37.5, 37.0, 33.3, 33.0, 32.7, 31.0, 28.2, 27.9, 27.2, 24.7, 23.4, 23.1, 21.3, 18.3, 17.6, 17.2, 15.7, 15.6; HRMS (ESI): m/z calcd for $C_{35}H_{57}O_2N_2^-$: 537.4415; found: 537.4428.

***N*-(piperidin-4-ylmethyl)-3*β*-hydroxy-urs-12-en-28-amide (B_9)**

White solid, yield 63.7%, m. p. 116.1-117.3 °C; ^1H NMR (500 MHz, $CDCl_3$) δ 6.05–5.99 (m, 1H, CONH), 5.31–5.27 (m, 1H, 12-CH=C), 3.29 (dt, *J* = 13.3, 6.6 Hz, 1H, 1′a-NH<u>CH₂</u>), 3.19 (dd, *J* = 11.1, 4.4 Hz, 1H, 3-<u>CH</u>OH), 3.12 (d, *J* = 11.6 Hz, 2H, 4′a-NH<u>CH₂</u>), 2.82–2.75 (m, 1H, 1′b-NH<u>CH₂</u>), 2.69–2.55 (m, 3H, 2′-NHCH₂<u>CH</u>+4′b-NH<u>CH₂</u>), 1.99–1.95 (m, 1H, 18-H), 1.94–1.82 (m, 4H, 11-H+2-H), 1.72–1.40 (m, 15H, 16-H+9-H+22-H+20-H+6a-H+1-H+19-H+3′-NHCH₂<u>CH₂</u>), 1.39–1.25 (m, 4H, 21-H+7-H), 1.07 (s, 3H, 27-CH_3), 1.03 (d, *J* = 13.9 Hz, 2H, 15-H), 0.96 (s, 3H, 23-CH_3), 0.93 (s, 3H, 30-CH_3), 0.89 (s, 3H, 25-CH_3), 0.85 (d, *J* = 6.5 Hz, 3H, 29-CH_3), 0.76 (s, 3H, 26-CH_3), 0.74 (s, 3H, 24-CH_3), 0.70 (d, *J* = 10.9 Hz, 1H, 5-H); ^{13}C NMR (126 MHz, $CDCl_3$) δ 178.4, 140.2, 125.6, 78.9, 55.2, 54.1, 48.0, 47.5, 45.84, 45.1, 42.6, 39.8, 39.6, 39.2, 38.8, 38.7, 37.4, 37.0, 36.0, 32.8, 31.0, 30.4, 28.2, 27.9, 27.2, 24.9, 23.5, 23.3, 21.3, 18.3, 17.3, 17.1, 15.7, 15.6; HRMS (ESI): m/z calcd for $C_{36}H_{59}O_2N_2^-$: 551.4571; found: 551.4586.

***N*-(3-(piperazin-1-yl)propyl)-3*β*-hydroxy-urs-12-en-28-amide (B_{10})**

White solid, yield 59.6%, m. p. 118.4-119.9 °C; ^1H NMR (500 MHz, $CDCl_3$) δ 6.42 (t, *J* = 5.1 Hz, CONH), 5.27 (t, *J* = 3.4 Hz, 12-CH=C), 3.44–3.45 (m, 1H, 1′a-NH<u>CH₂</u>), 3.20 (dd, *J* = 11.1, 4.5 Hz, 3-<u>CH</u>OH), 3.08–2.99 (m, 1H, 1′b-NH<u>CH₂</u>), 2.94 (t, *J* = 4.9

Hz, 4H, 5′-NH<u>CH</u>$_2$CH$_2$), 2.44 (s, 4H, 4′-NCH$_2$), 2.40–2.34 (m, 3H, 18-H+3′-NHCH$_2$CH$_2$<u>CH$_2$</u>), 1.99–1.89 (m, 4H, 11-H+2-H), 1.70–1.42 (m, 13H, 16-H+9-H+22-H+20-H+6-H+1-H+19-H+2′-NHCH$_2$<u>CH$_2$</u>CH$_2$), 1.39–1.25 (m, 4H, 21-H+7-H), 1.07 (s, 4H, 27-CH$_3$+5′-NH), 1.05–0.98 (m, 2H, 15-H), 0.97 (s, 3H, 23-CH$_3$), 0.94 (s, 3H, 30-CH$_3$), 0.90 (s, 3H, 25-CH$_3$), 0.86 (d, J = 6.5 Hz, 3H, 29-CH$_3$), 0.76 (s, 3H, 26-CH$_3$), 0.75 (s, 3H, 24-CH$_3$), 0.70 (d, J = 11.5 Hz, 1H, 5-H); ^{13}C NMR (101 MHz, CDCl$_3$) δ 178.0, 139.7, 125.5, 78.9, 57.4, 55.1, 53.7, 47.5, 47.5, 45.5, 42.4, 39.7, 39.5, 39.1, 38.8, 38.6, 38.6, 37.4, 36.9, 32.8, 30.9, 29.7, 28.2, 27.8, 27.2, 25.6, 24.8, 23.4, 23.4, 21.3, 18.3, 17.3, 17.0, 15.7, 15.5; HRMS (ESI): m/z calcd for $C_{37}H_{62}O_2N_3^-$: 580.4837; found: 580.4850.

3.^{1}H NMR, ^{13}C NMR, HRMS spectra for all compounds (Figures S2 to S115)

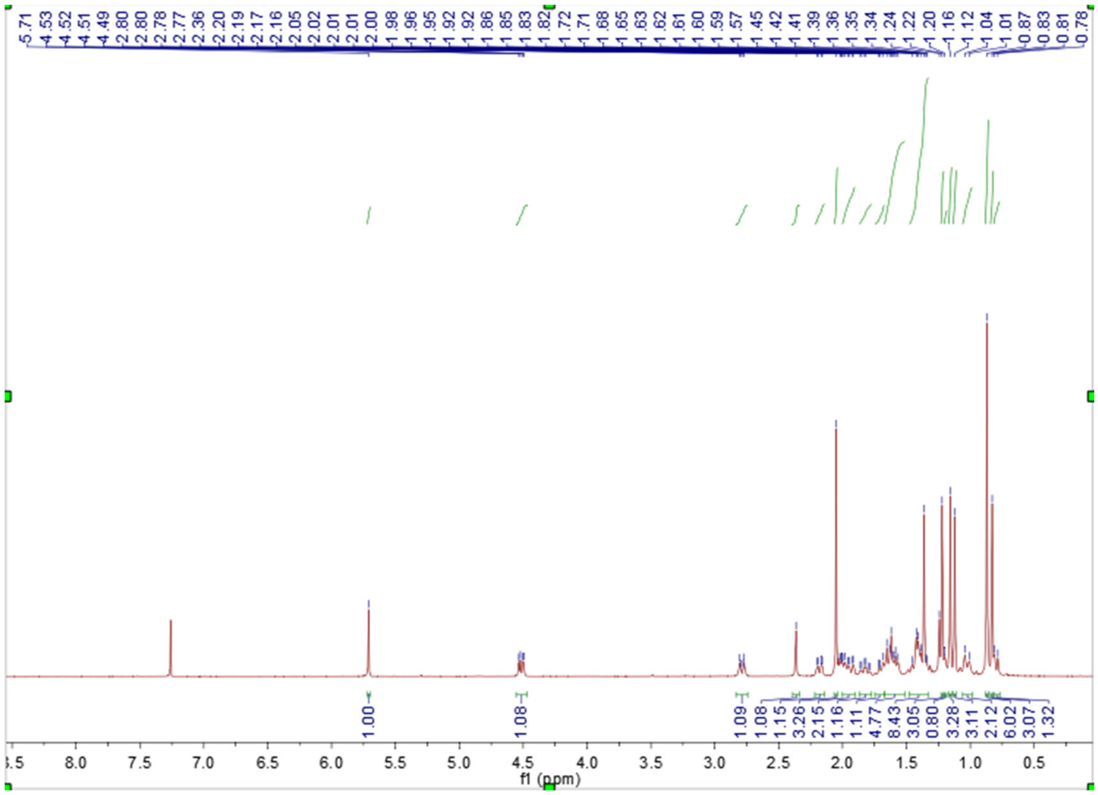

Figure S1. ^{1}H NMR spectrum (CDCl$_3$, 400 MHz) of Intermediate **1.**

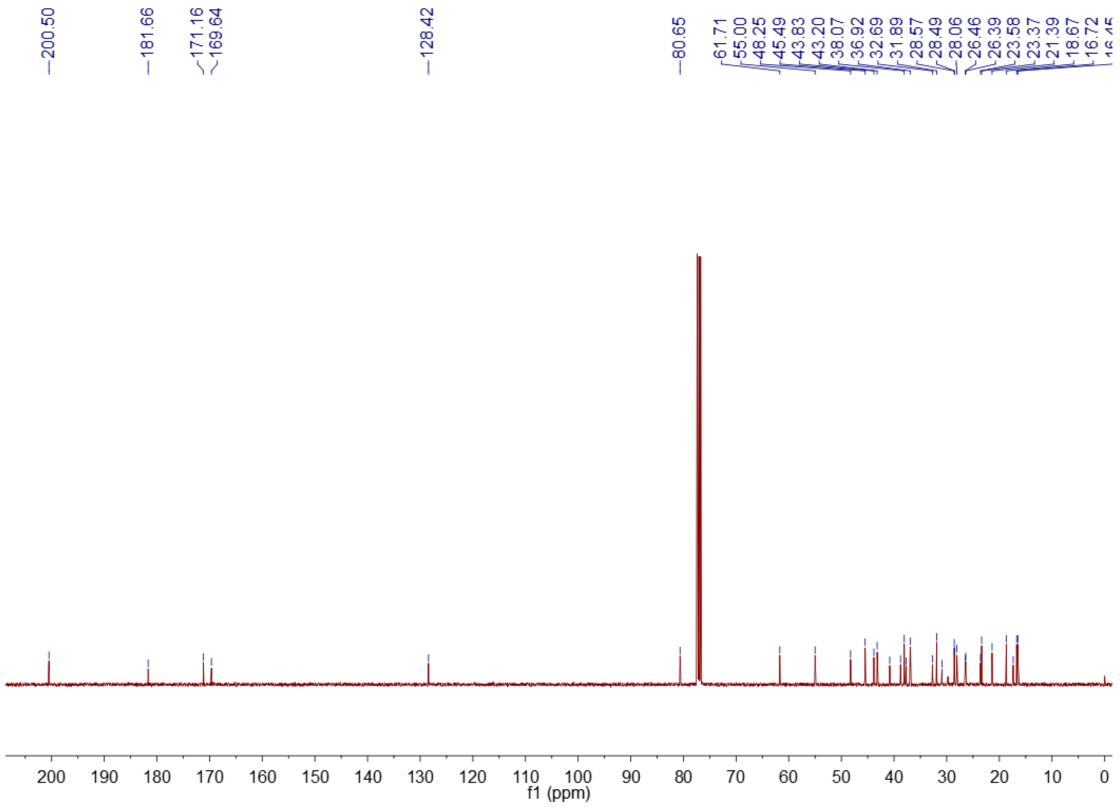

Figure S2. ^{13}C NMR spectrum ($CDCl_3$, 101 MHz) of Intermediate **1.**

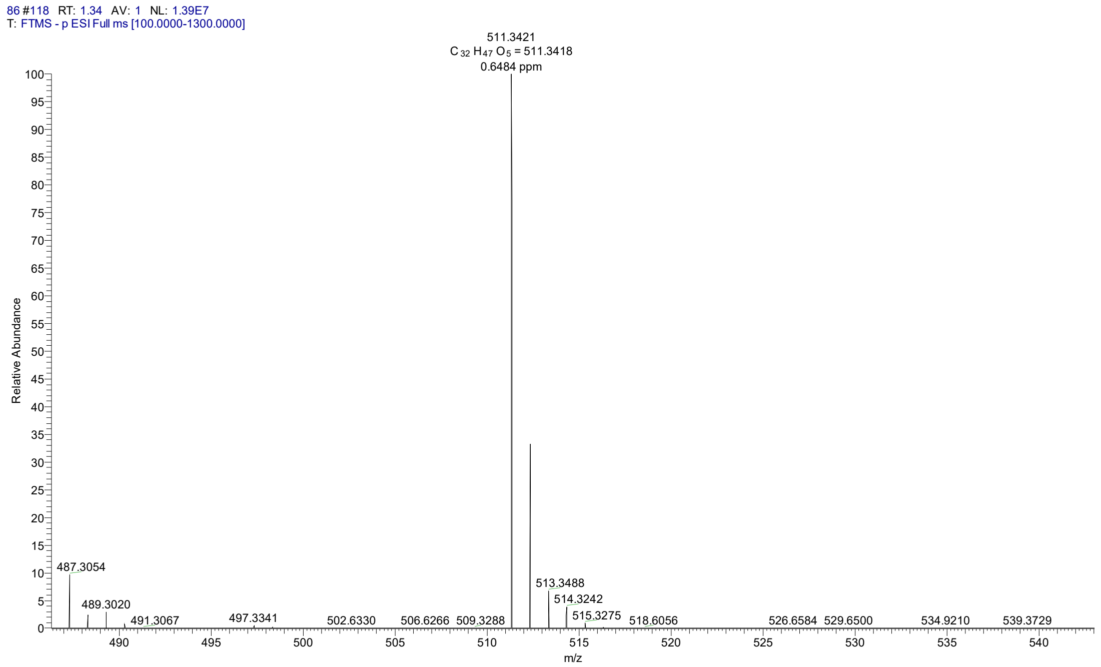

Figure S3. HRMS spectrum of Intermediate **1.**

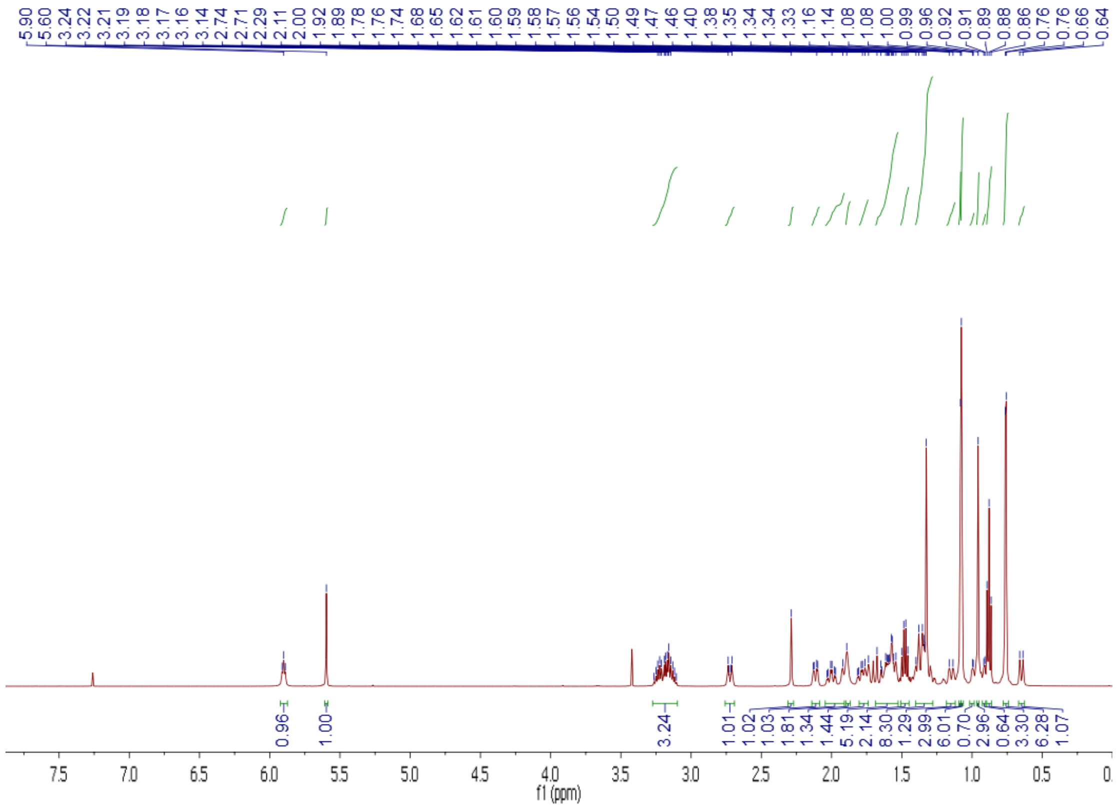

Figure S4. ^{1}H NMR spectrum ($CDCl_3$, 500 MHz) of target compound $\mathbf{A_1}$.

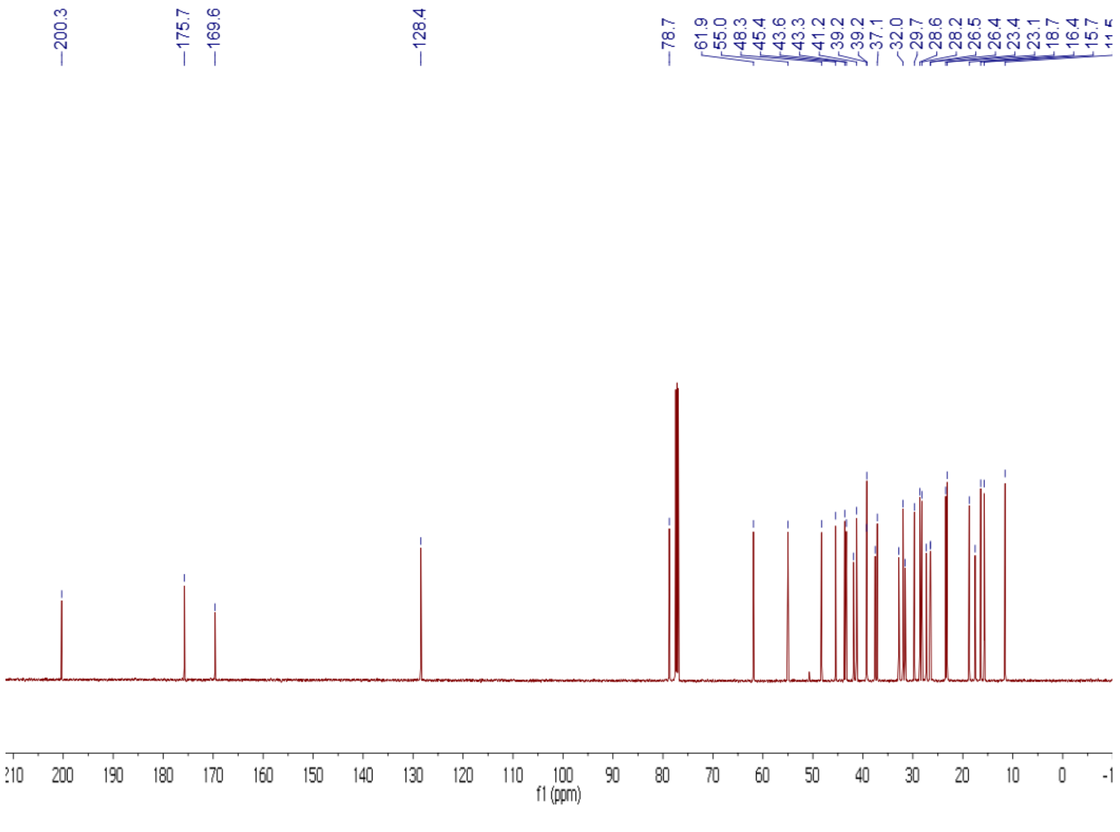

Figure S5. ^{13}C NMR spectrum ($CDCl_3$, 126 MHz) of target compound $\mathbf{A_1}$.

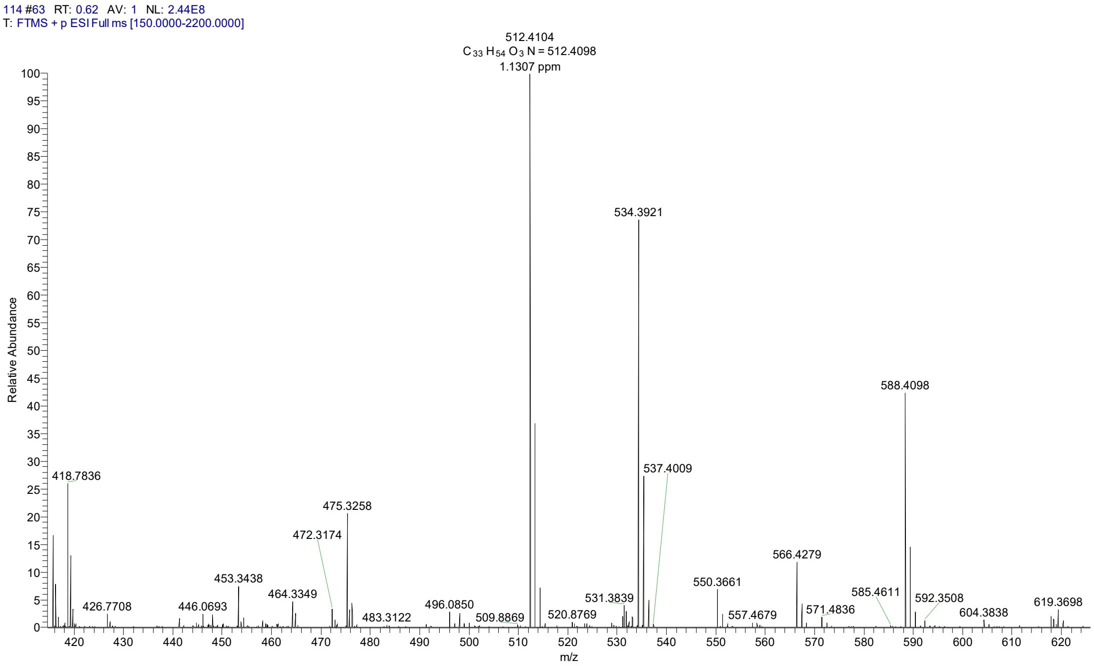

Figure S6. HRMS spectrum of target compound **A₁.**

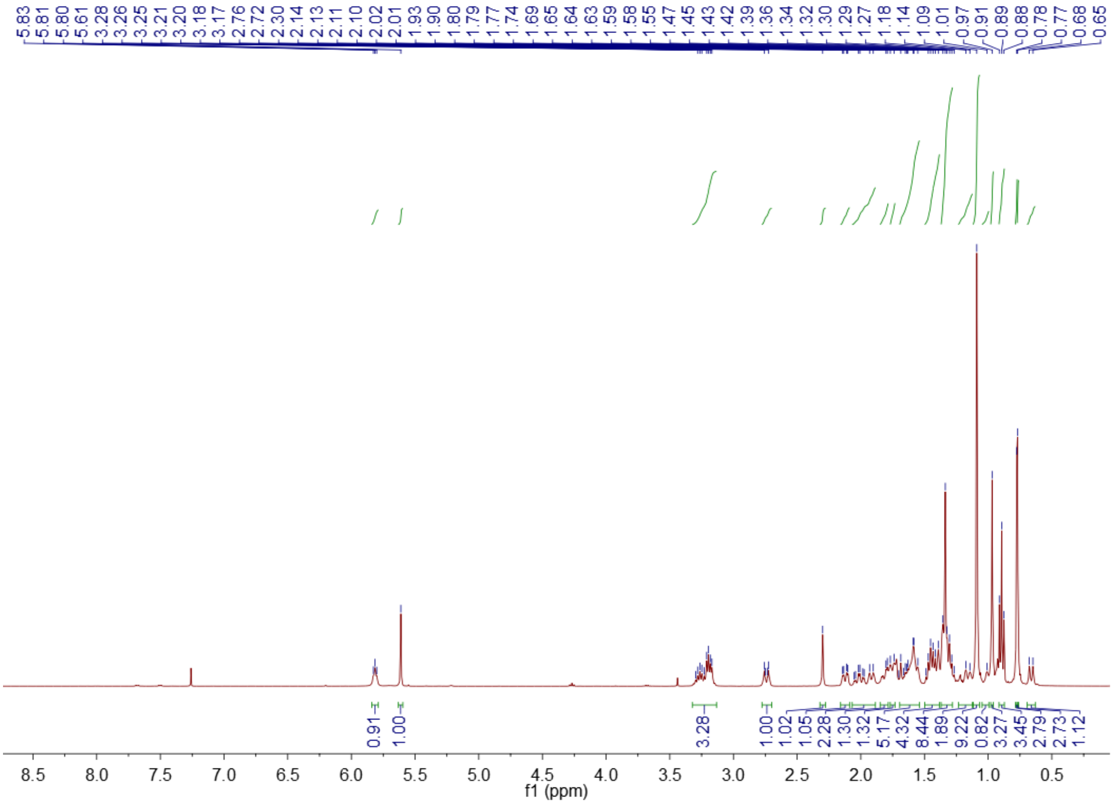

Figure S7. ^{1}H NMR spectrum ($CDCl_3$, 400 MHz) of target compound **A_2.**

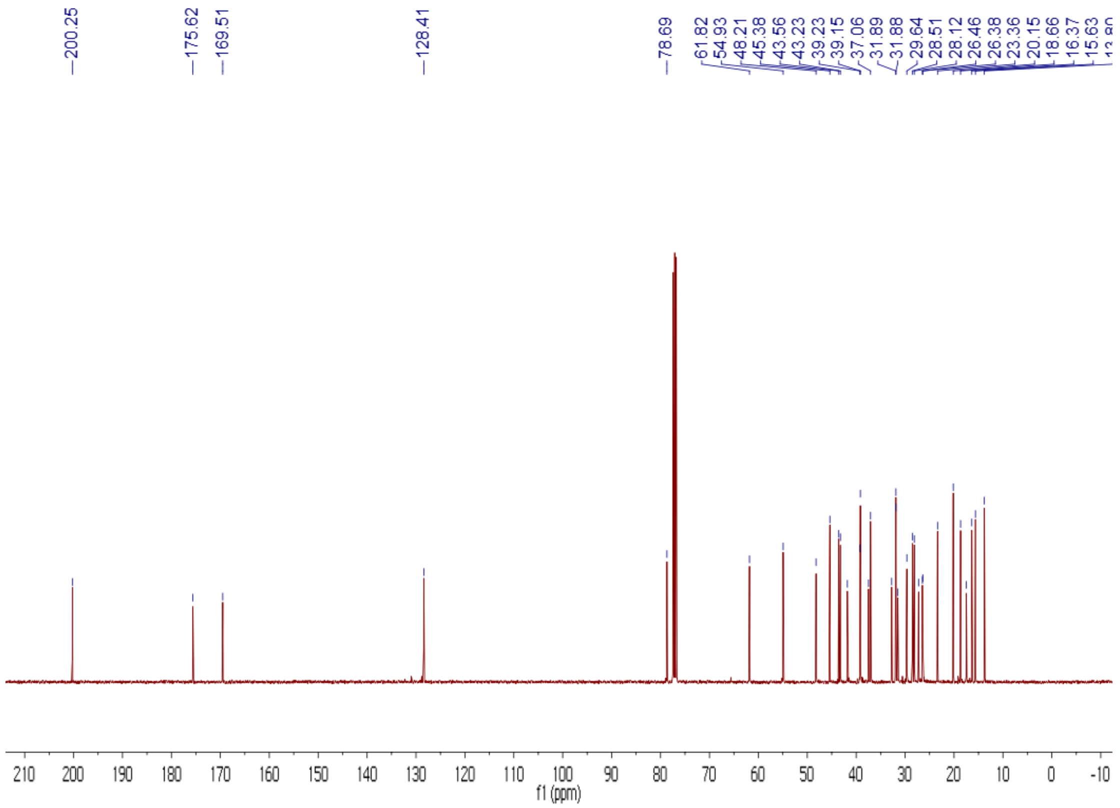

Figure S8. ^{13}C NMR spectrum ($CDCl_3$, 101 MHz) of target compound **A$_2$.**

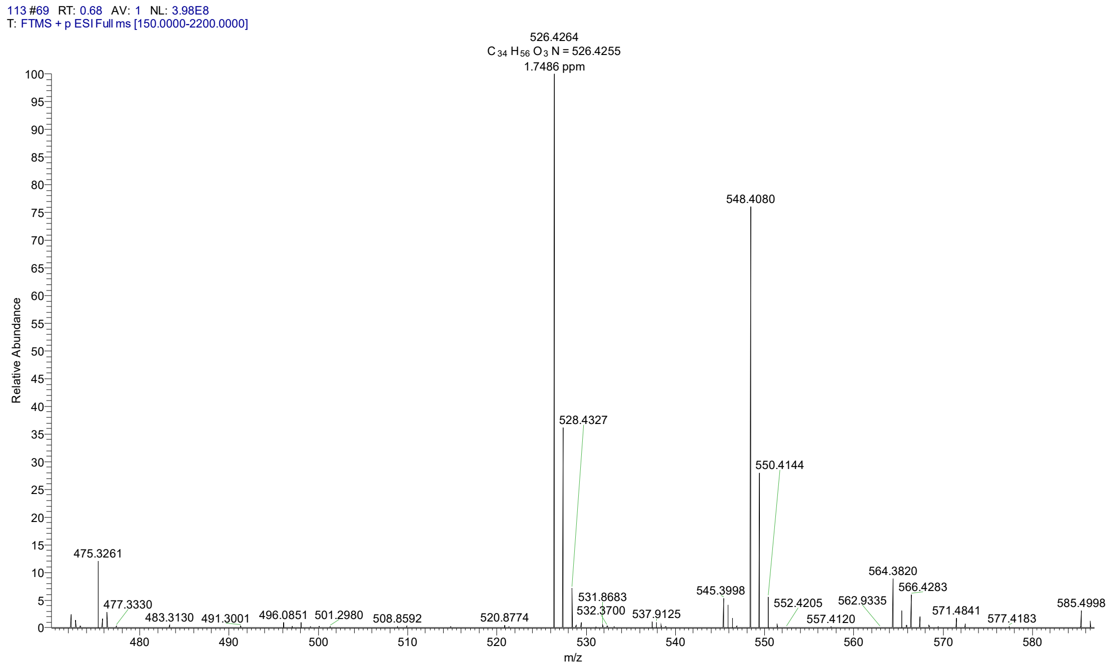

Figure S9. HRMS spectrum of target compound **A$_2$.**

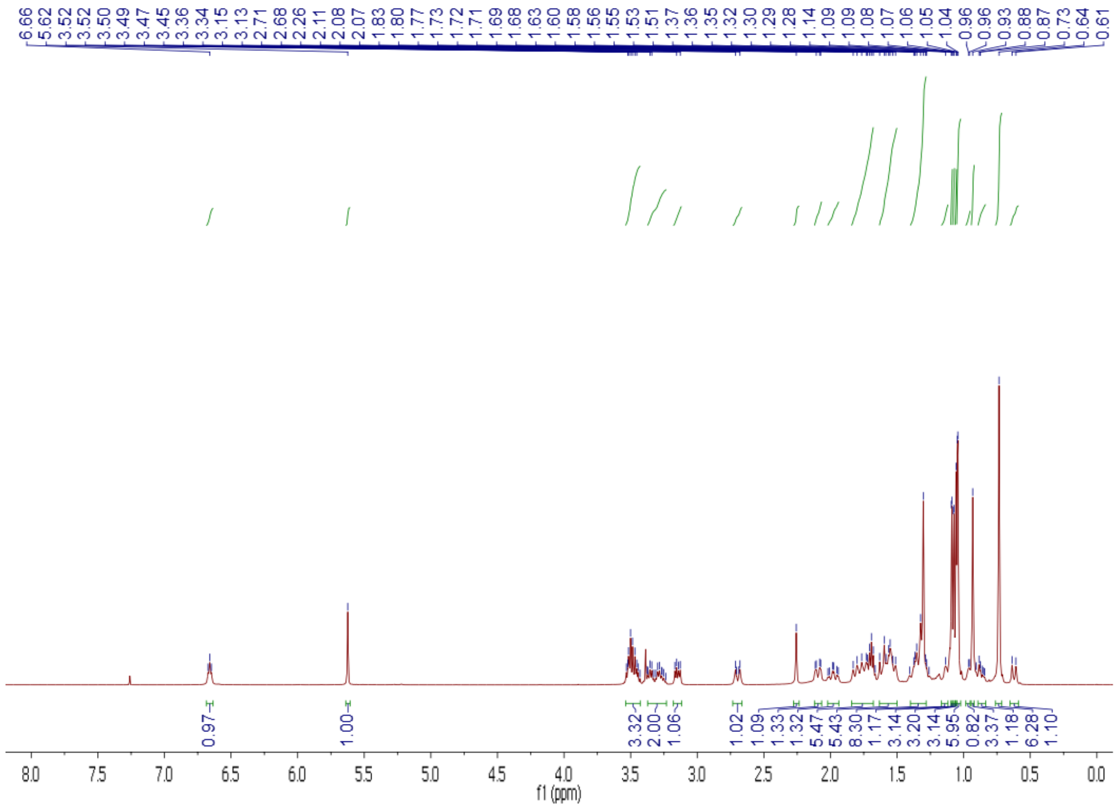

Figure S10. ^{1}H NMR spectrum ($CDCl_3$, 400 MHz) of target compound **A_3.**

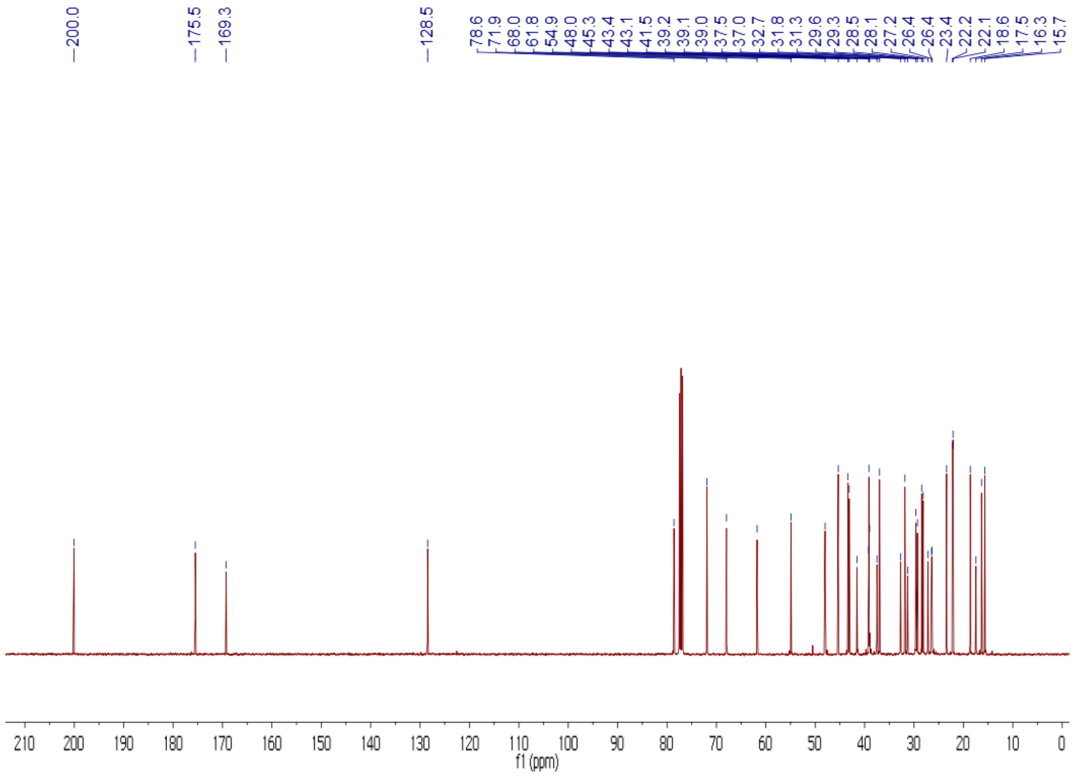

Figure S11. ^{13}C NMR spectrum ($CDCl_3$, 101 MHz) of target compound **A_3.**

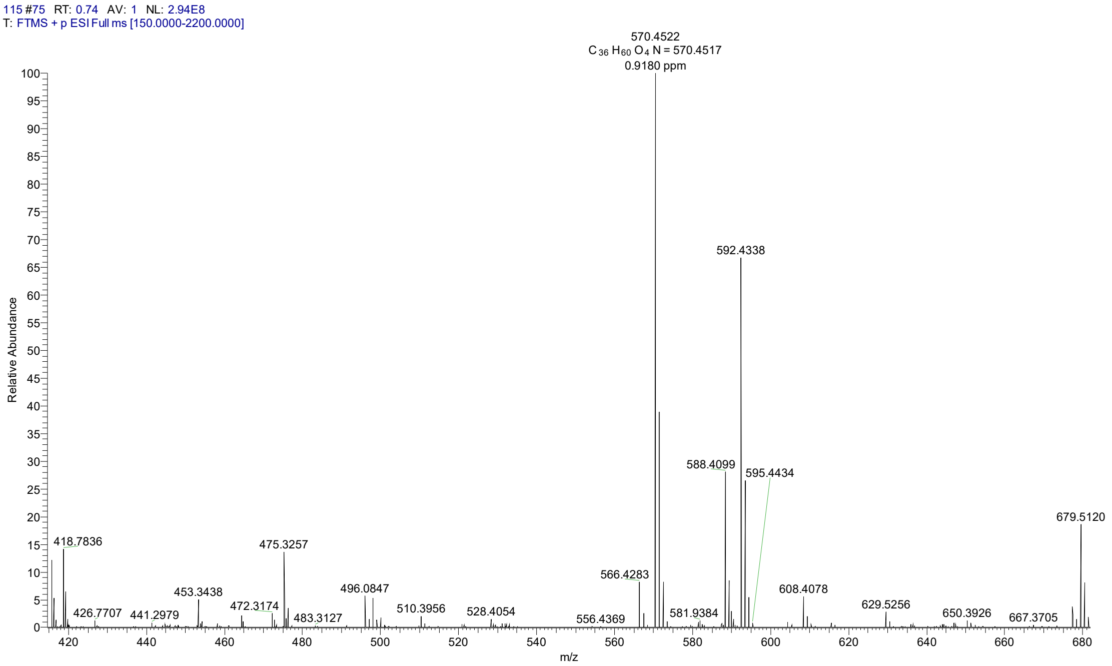

Figure S12. HRMS spectrum of target compound **A$_3$.**

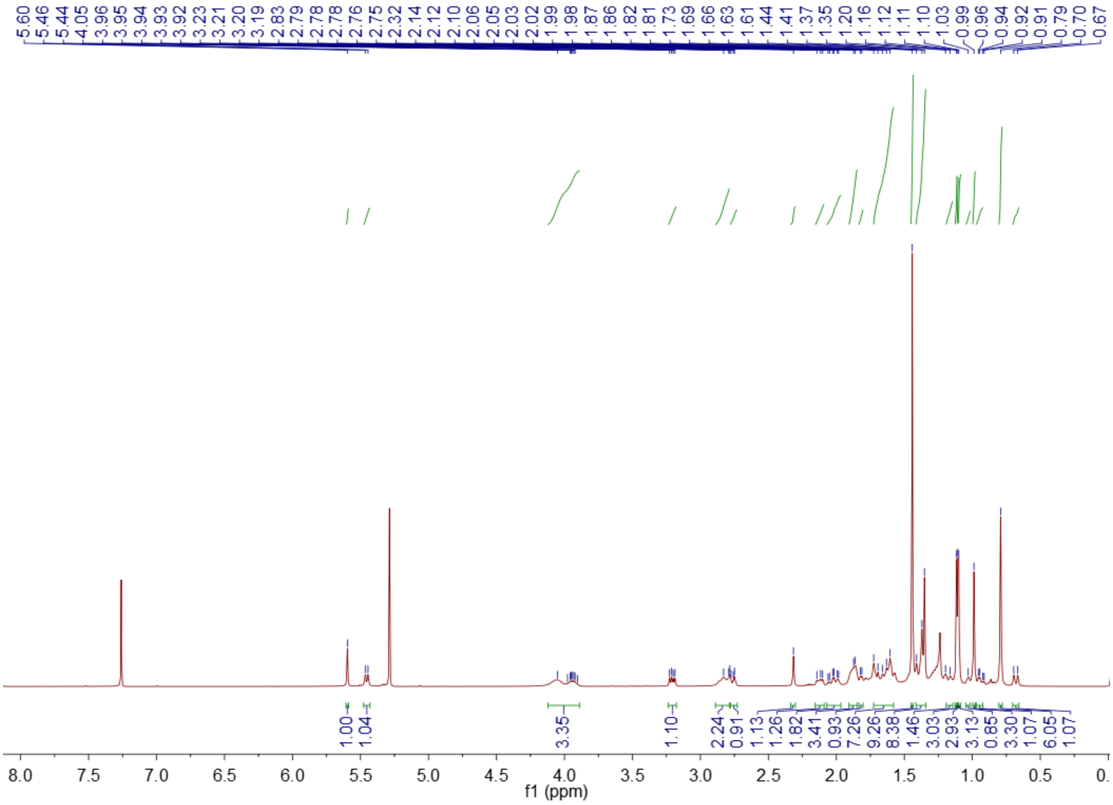

Figure S13. ^{1}H NMR spectrum ($CDCl_3$, 400 MHz) of target compound **A$_4$.**

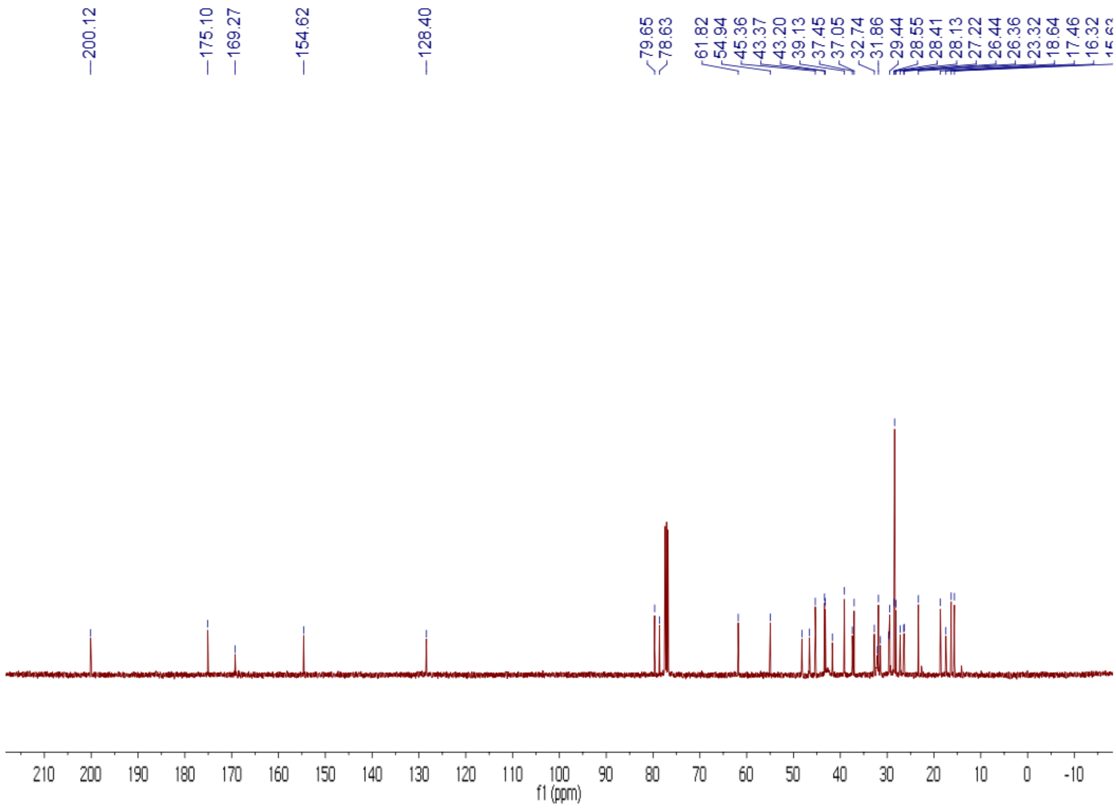

Figure S14. ^{13}C NMR spectrum ($CDCl_3$, 101 MHz) of target compound **A_4.**

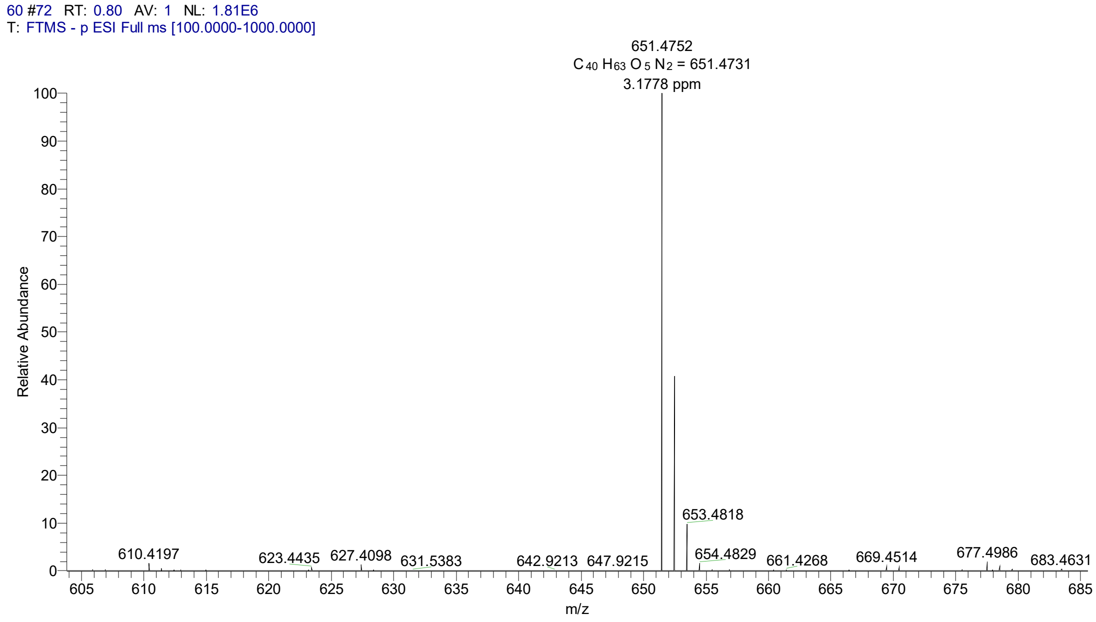

Figure S15. HRMS spectrum of target compound **A_4.**

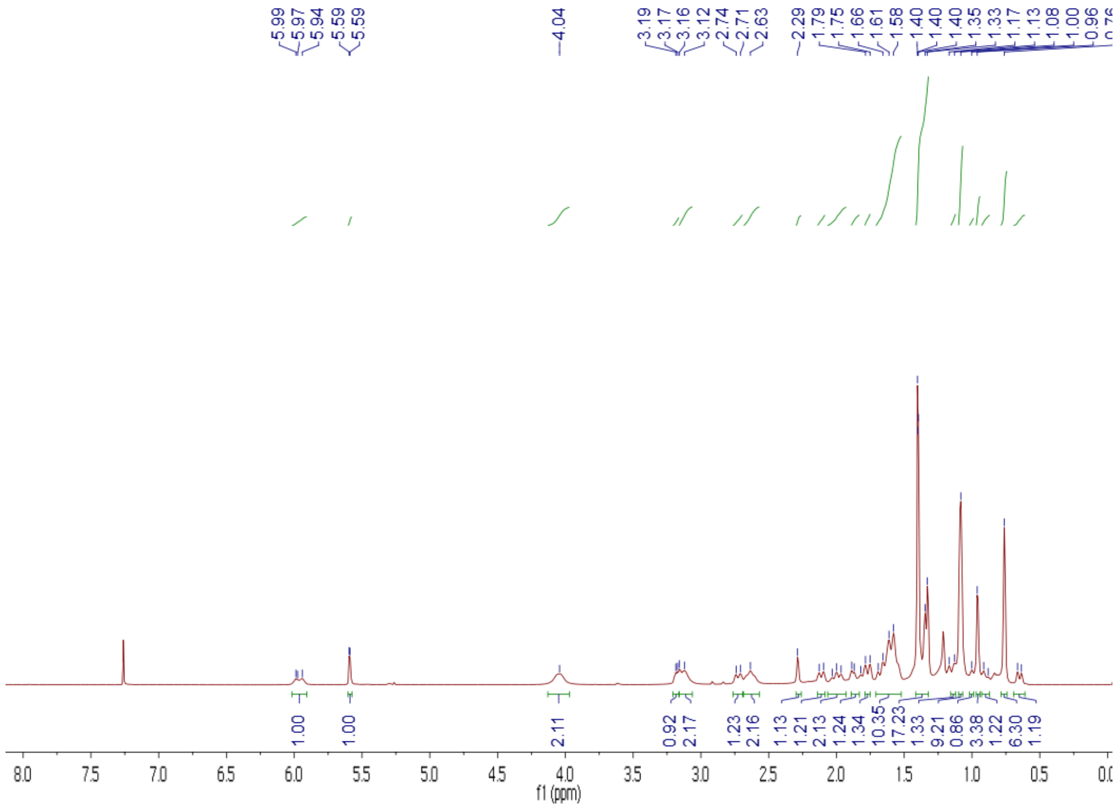

Figure S16. ^{1}H NMR spectrum ($CDCl_3$, 400 MHz) of target compound $\mathbf{A_5}$.

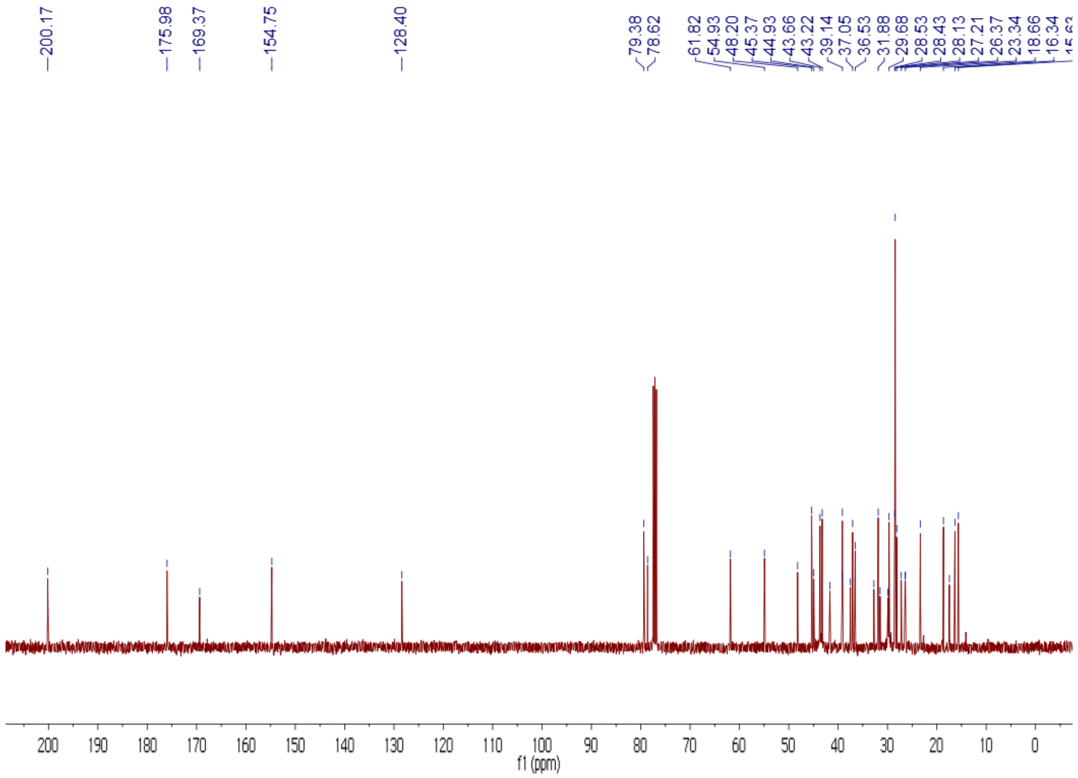

Figure S17. ^{13}C NMR spectrum ($CDCl_3$, 101 MHz) of target compound $\mathbf{A_5}$.

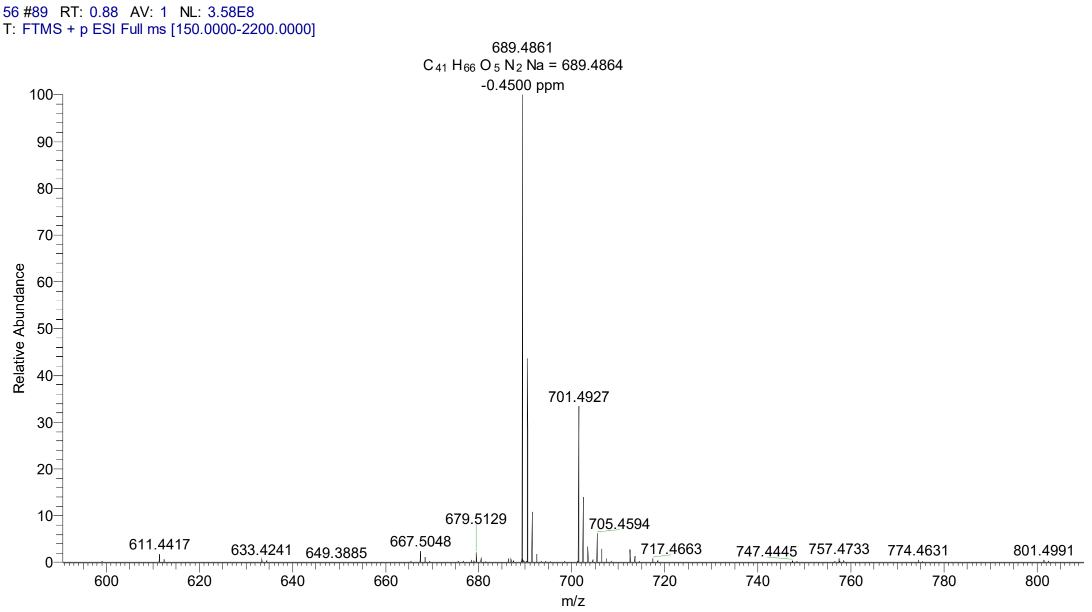

Figure S18. HRMS spectrum of target compound **A$_5$.**

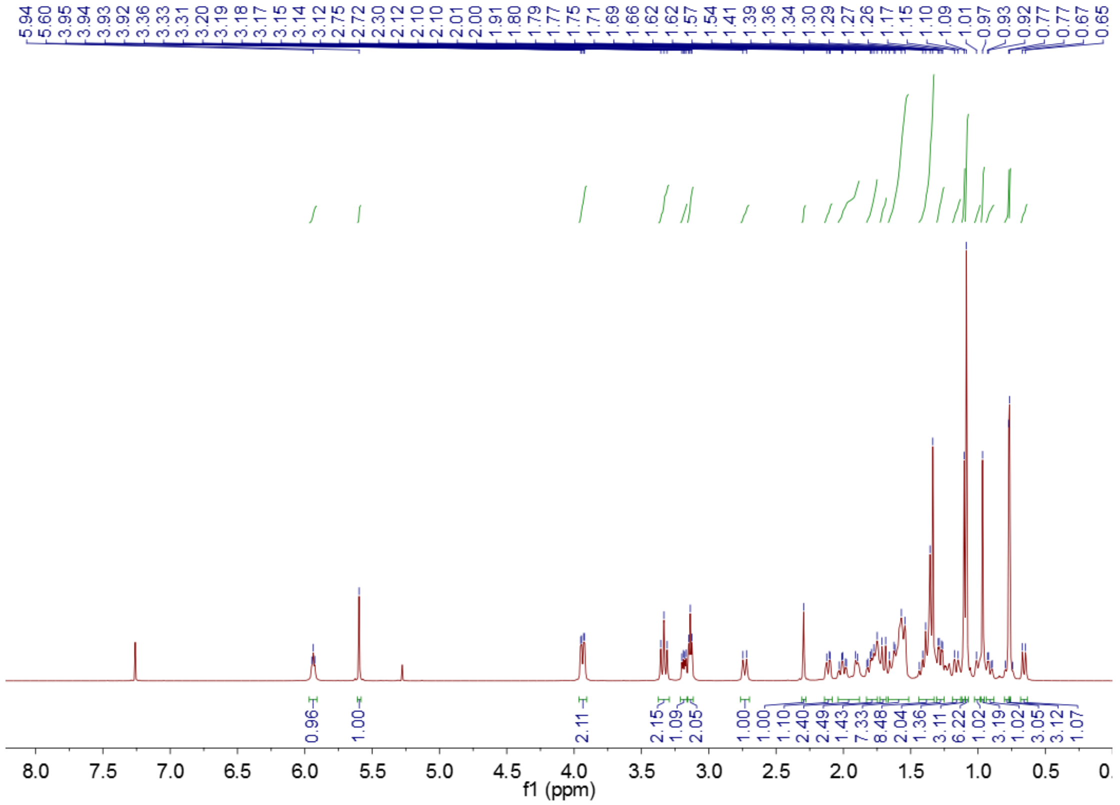

Figure S19. ^{1}H NMR spectrum ($CDCl_3$, 500 MHz) of target compound **A$_6$.**

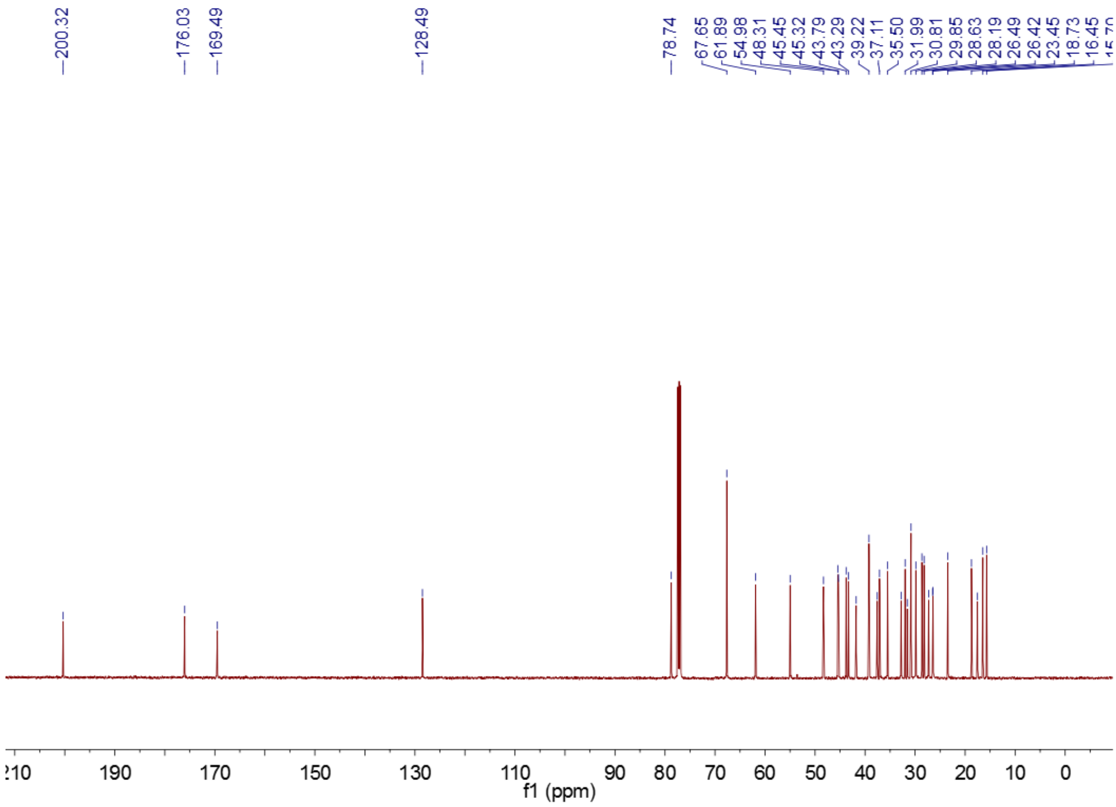

Figure S20. ^{13}C NMR spectrum ($CDCl_3$, 126 MHz) of target compound $\mathbf{A_6}$.

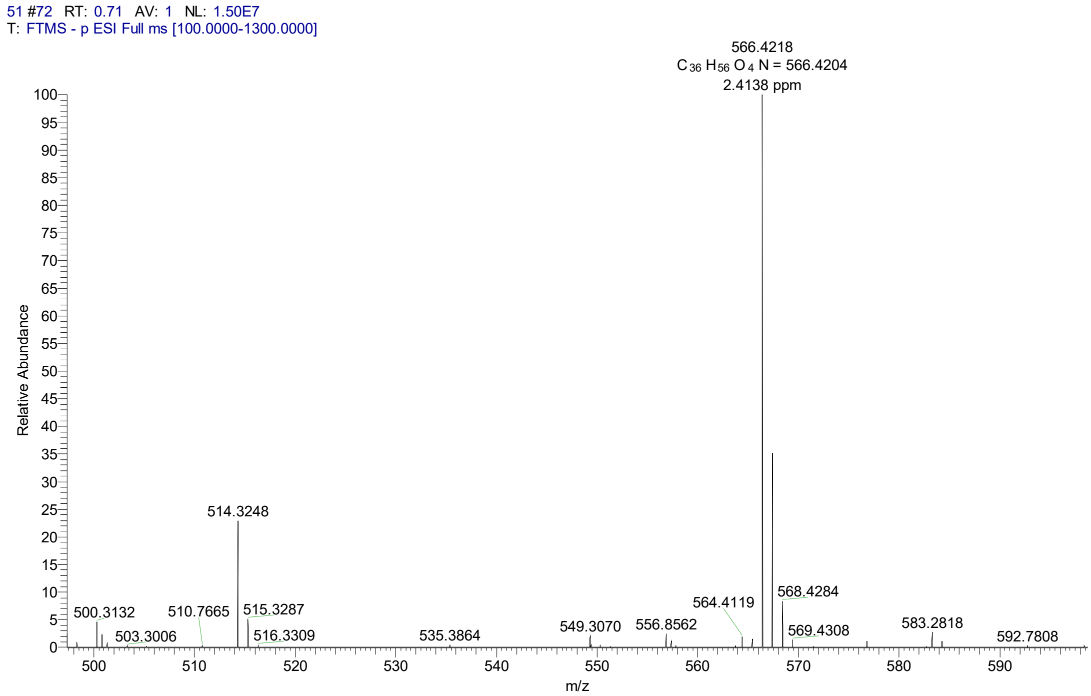

Figure S21. HRMS spectrum of target compound $\mathbf{A_6}$.

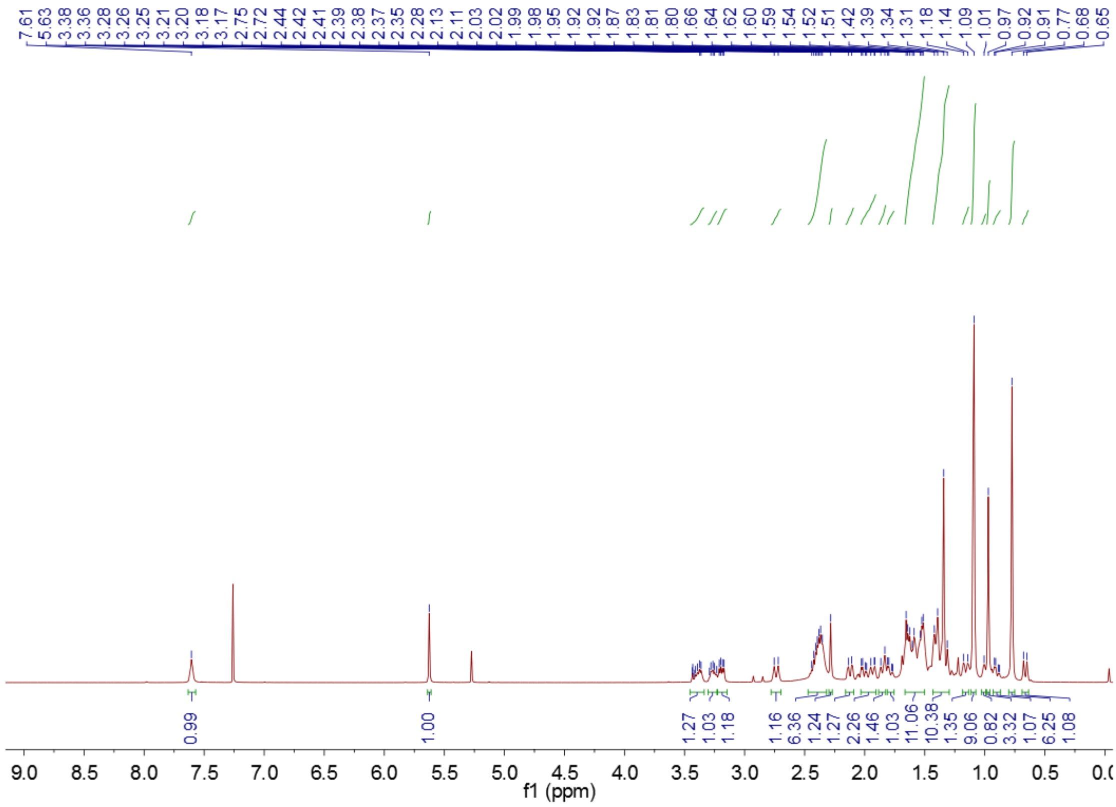

Figure S22. ^{1}H NMR spectrum ($CDCl_3$, 400 MHz) of target compound **A_7.**

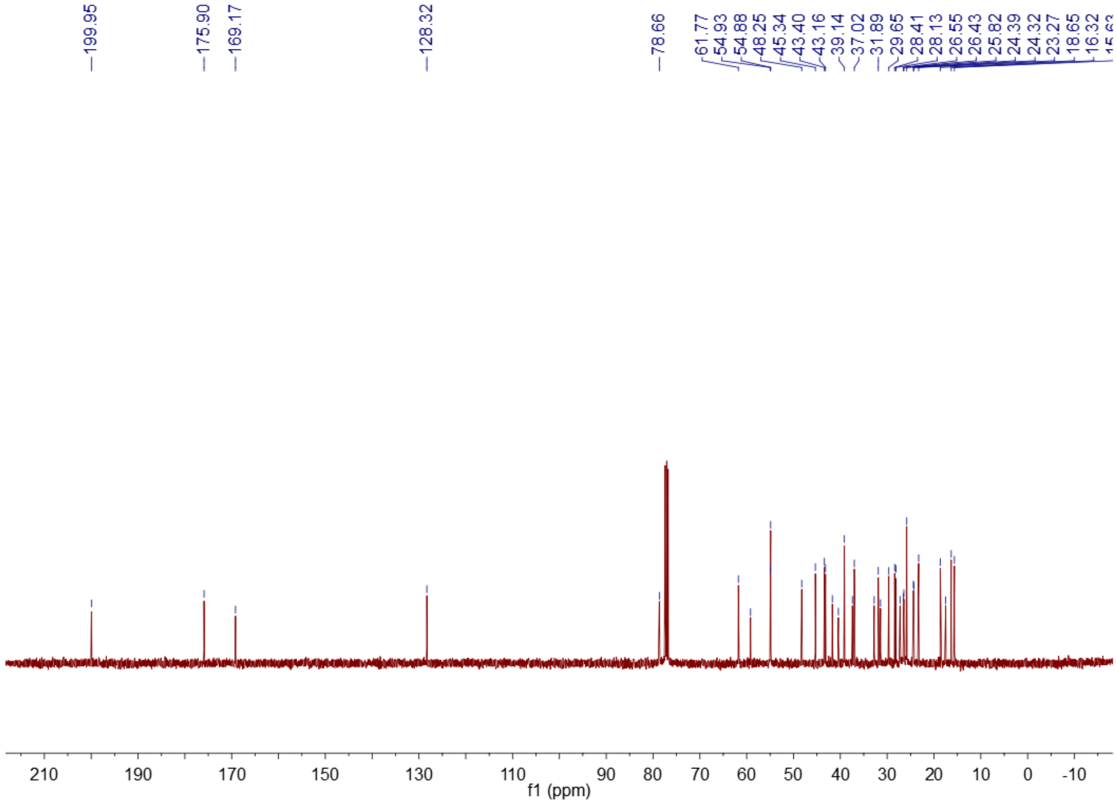

Figure S23. ^{13}C NMR spectrum ($CDCl_3$, 101 MHz) of target compound **A_7.**

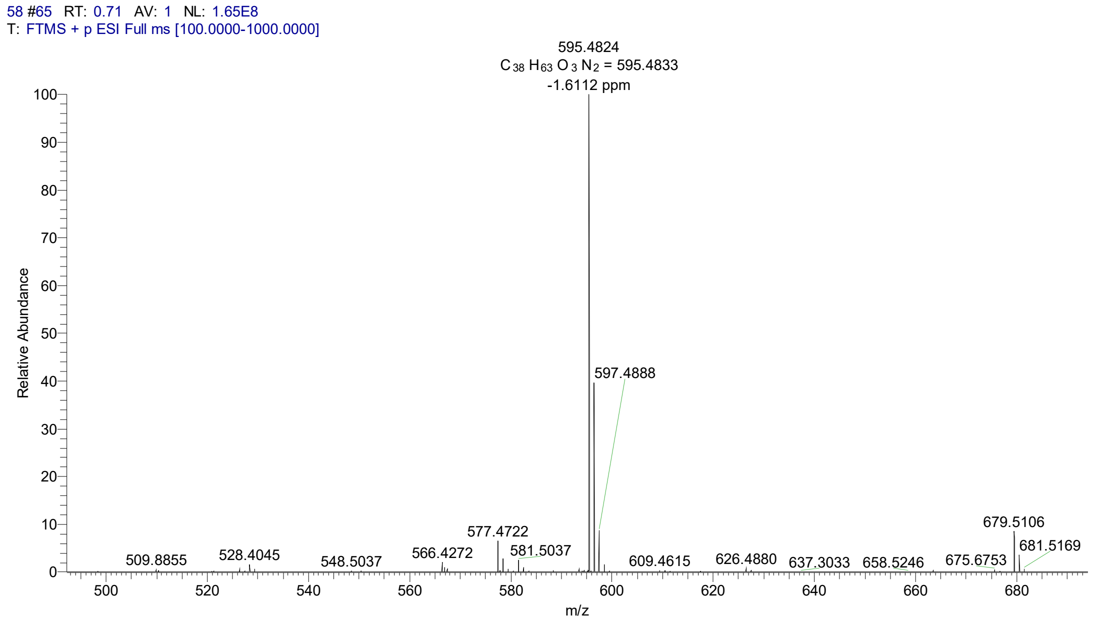

Figure S24. HRMS spectrum of target compound **A₇.**

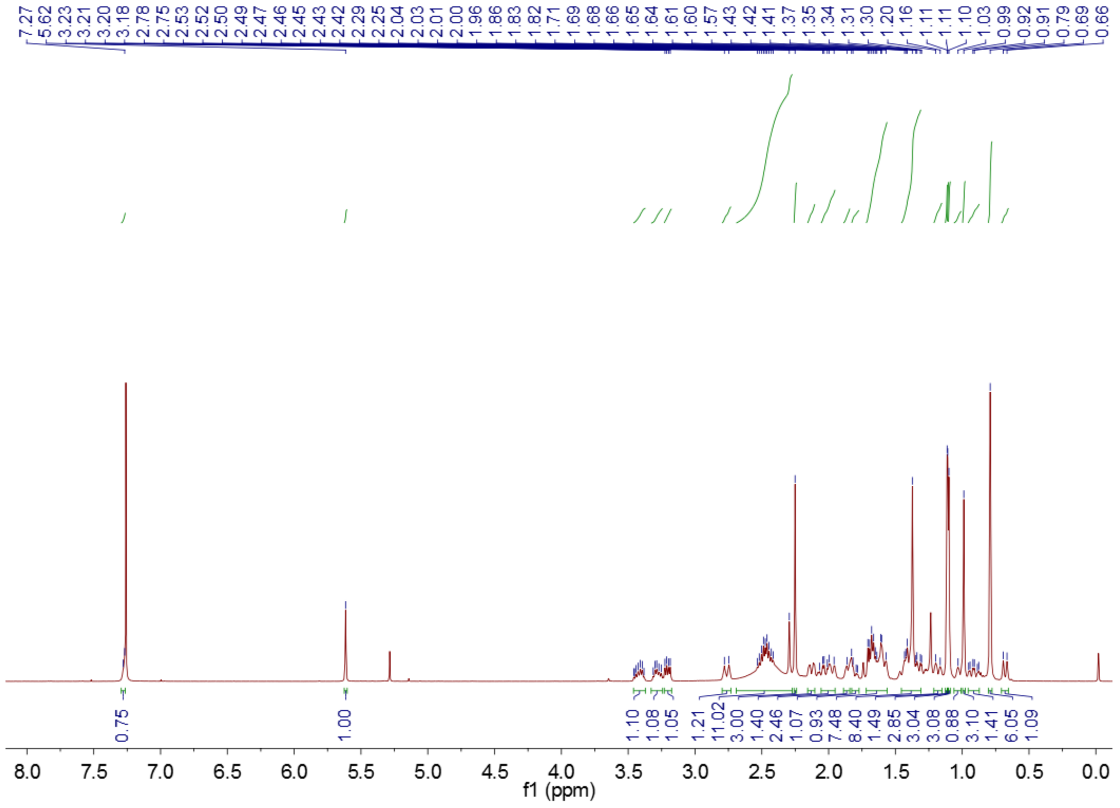

Figure S25. ^{1}H NMR spectrum ($CDCl_3$, 400 MHz) of target compound **A₈.**

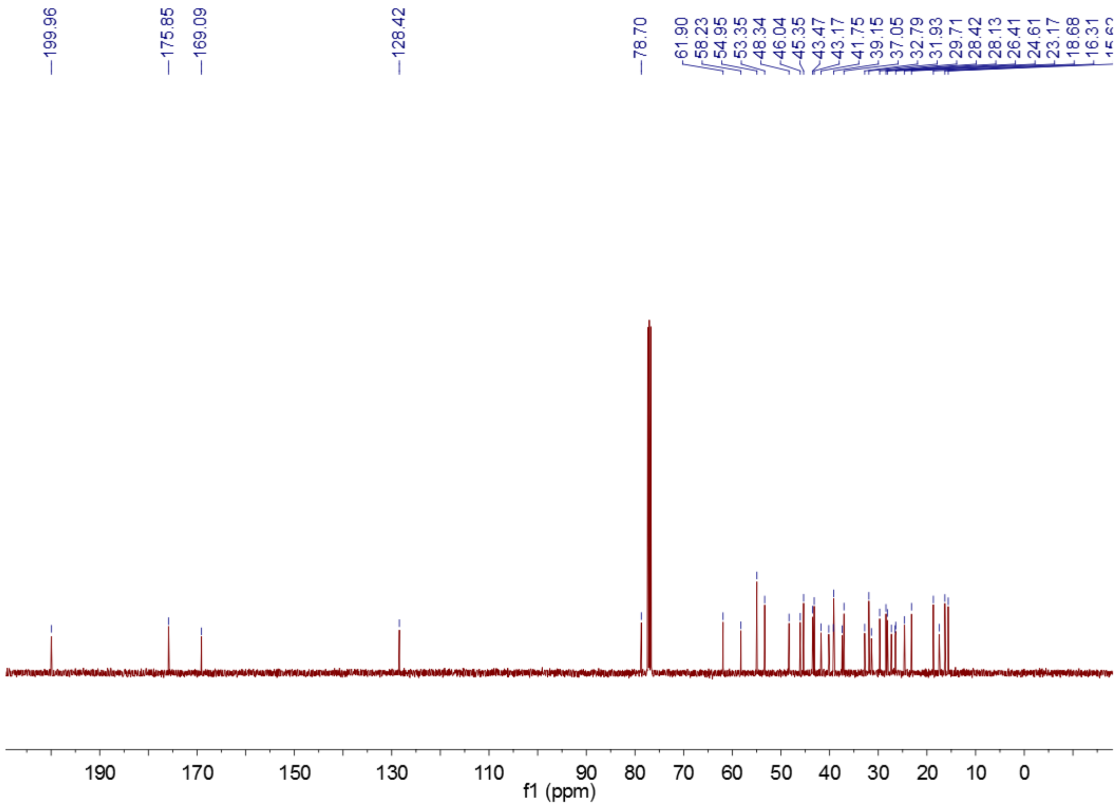

Figure S26. ^{13}C NMR spectrum ($CDCl_3$, 101 MHz) of target compound **A_8.**

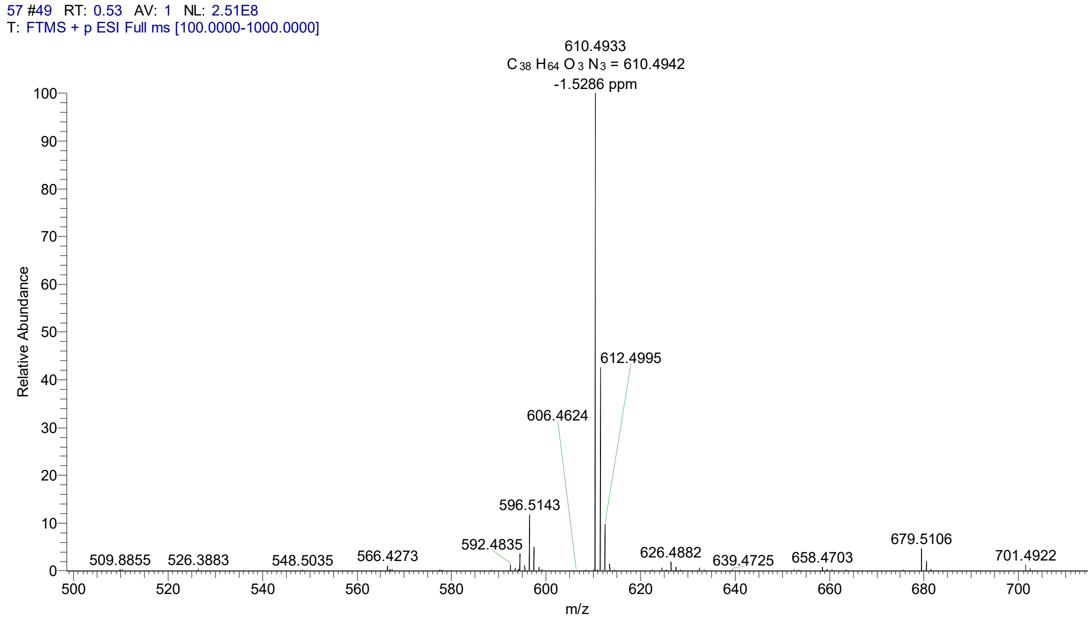

Figure S27. HRMS spectrum of target compound **A_8.**

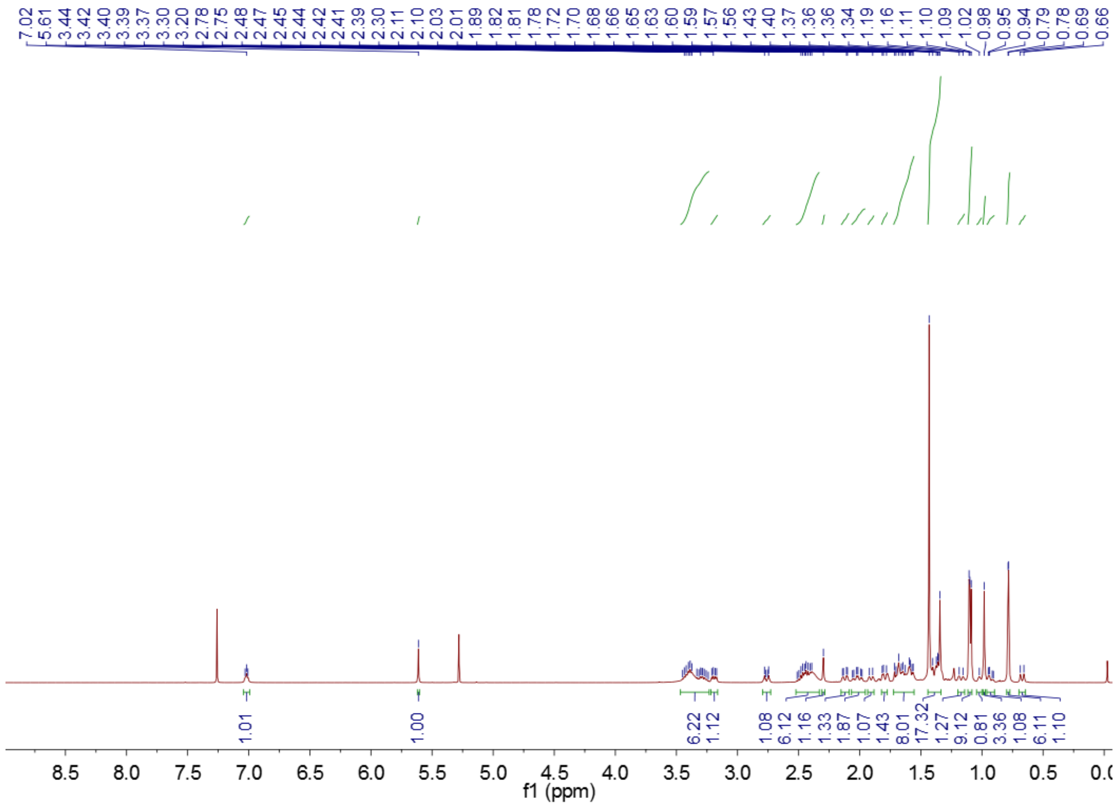

Figure S28. ^{1}H NMR spectrum ($CDCl_3$, 400 MHz) of target compound **A_9.**

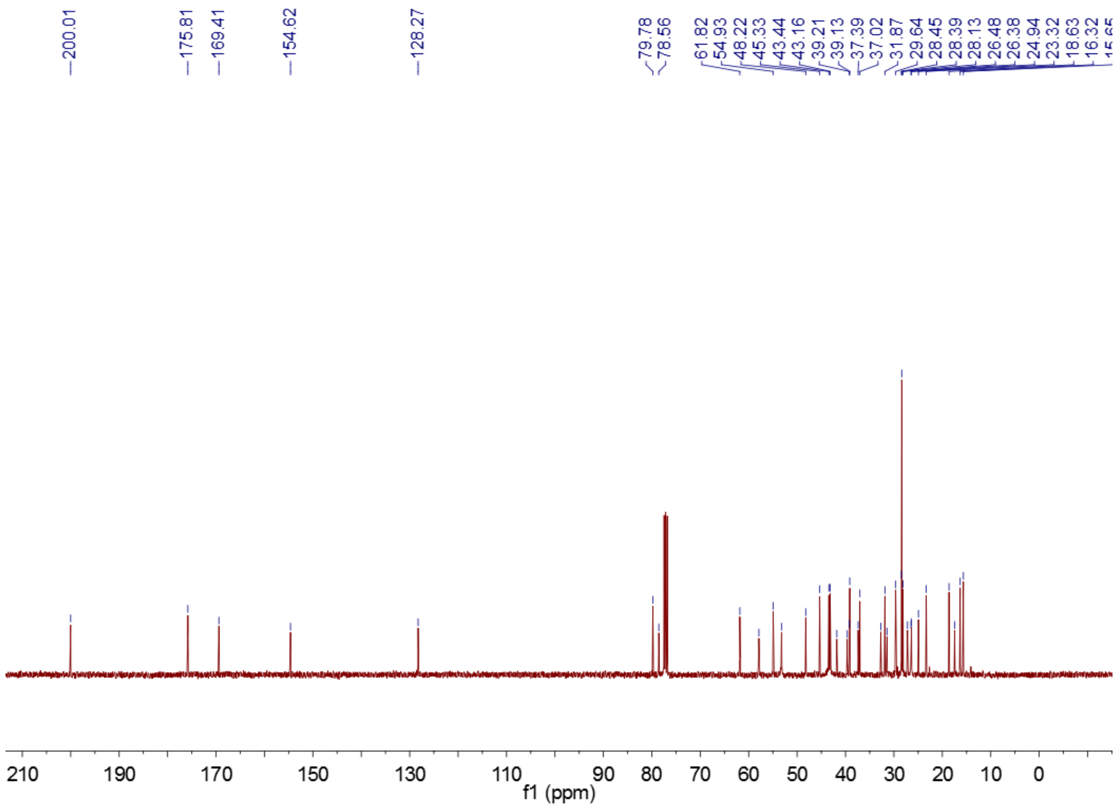

Figure S29. ^{13}C NMR spectrum ($CDCl_3$, 101 MHz) of target compound **A_9.**

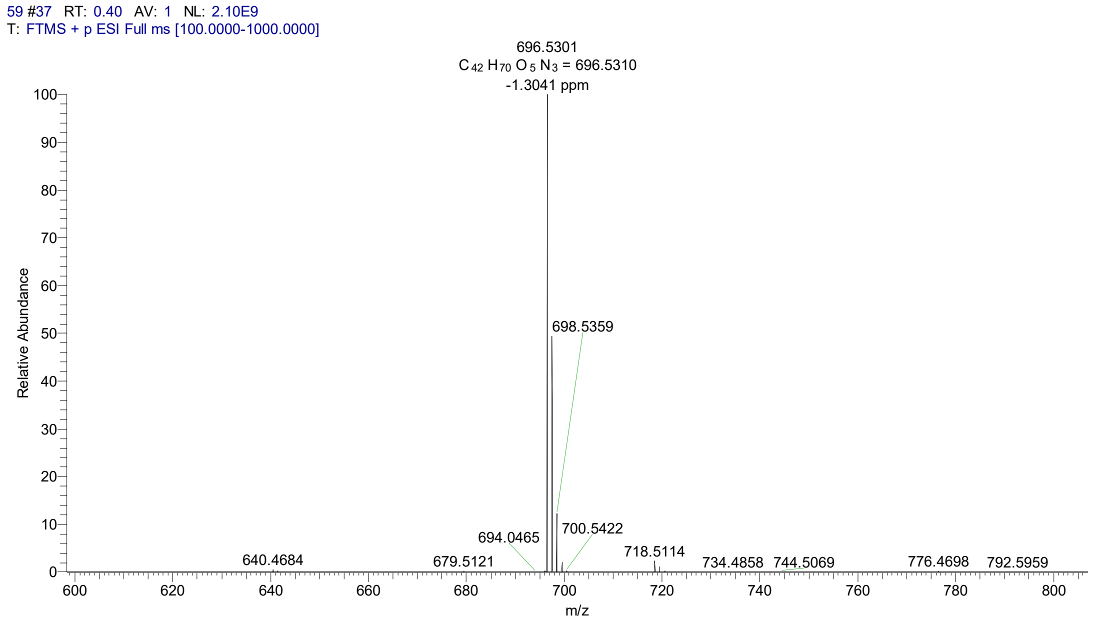

Figure S30. HRMS spectrum of target compound $\mathbf{A_9}$.

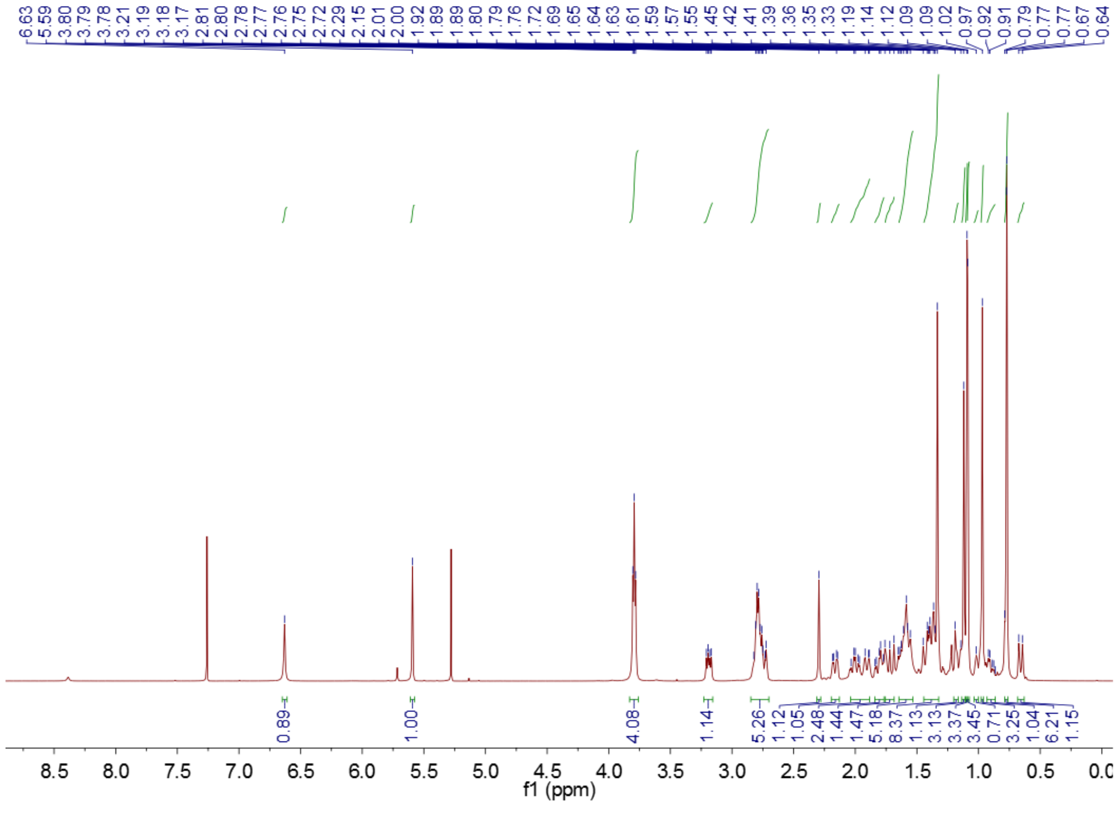

Figure S31. ^{1}H NMR spectrum (CDCl$_3$, 400 MHz) of target compound $\mathbf{A_{10}}$.

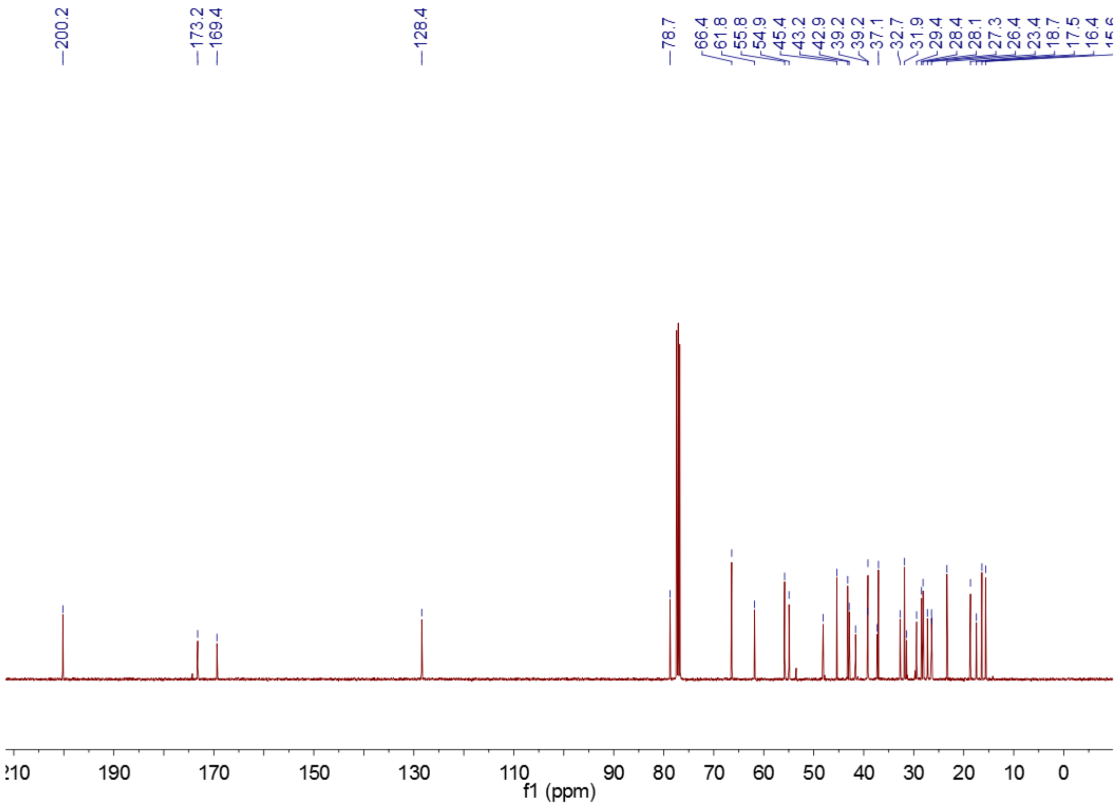

Figure S32. ^{13}C NMR spectrum ($CDCl_3$, 101 MHz) of target compound $\mathbf{A_{10}}$.

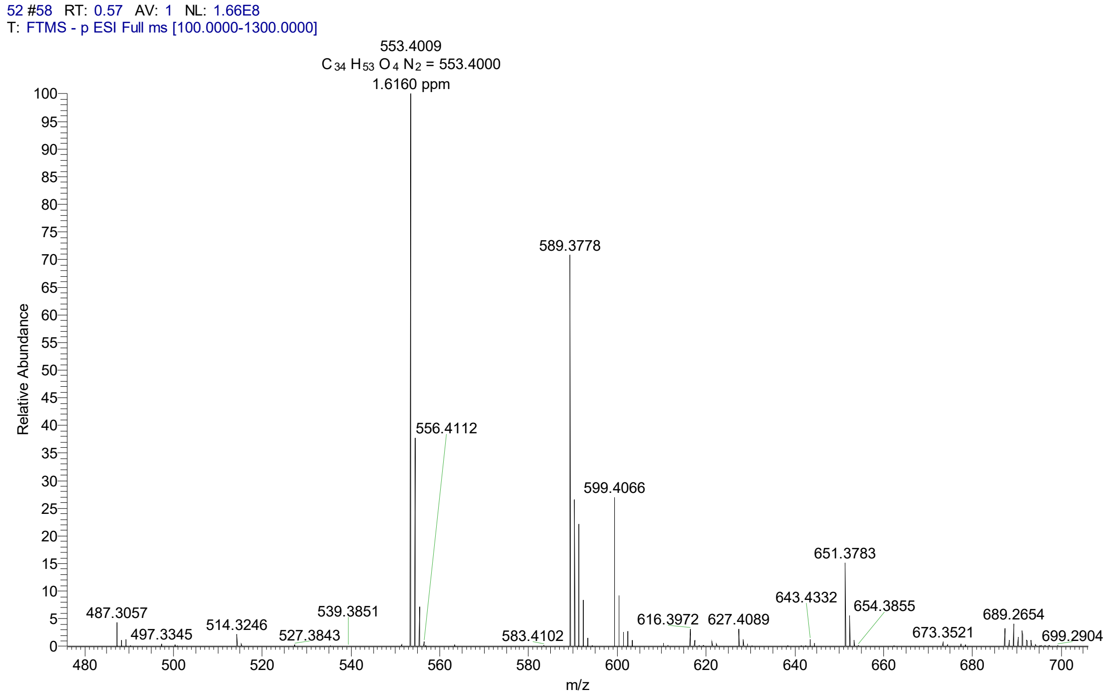

Figure S33. HRMS spectrum of target compound $\mathbf{A_{10}}$.

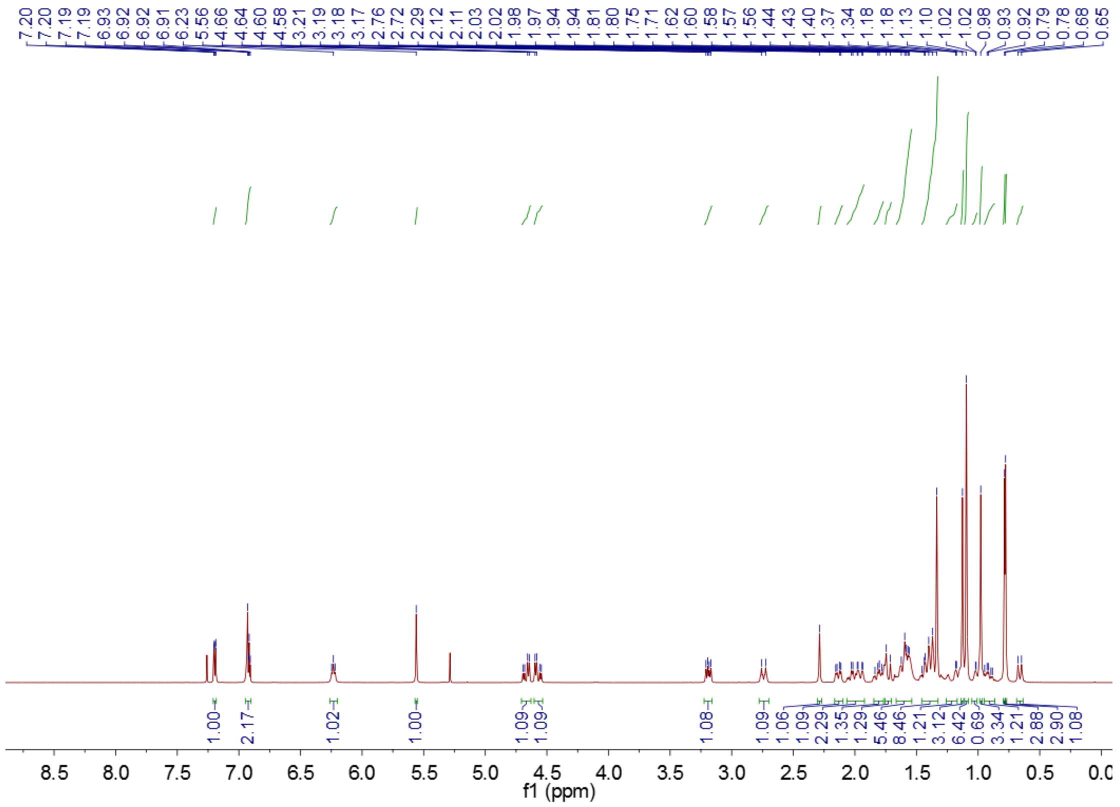

Figure S34. ^{1}H NMR spectrum ($CDCl_3$, 400 MHz) of target compound $\mathbf{A_{11}}$.

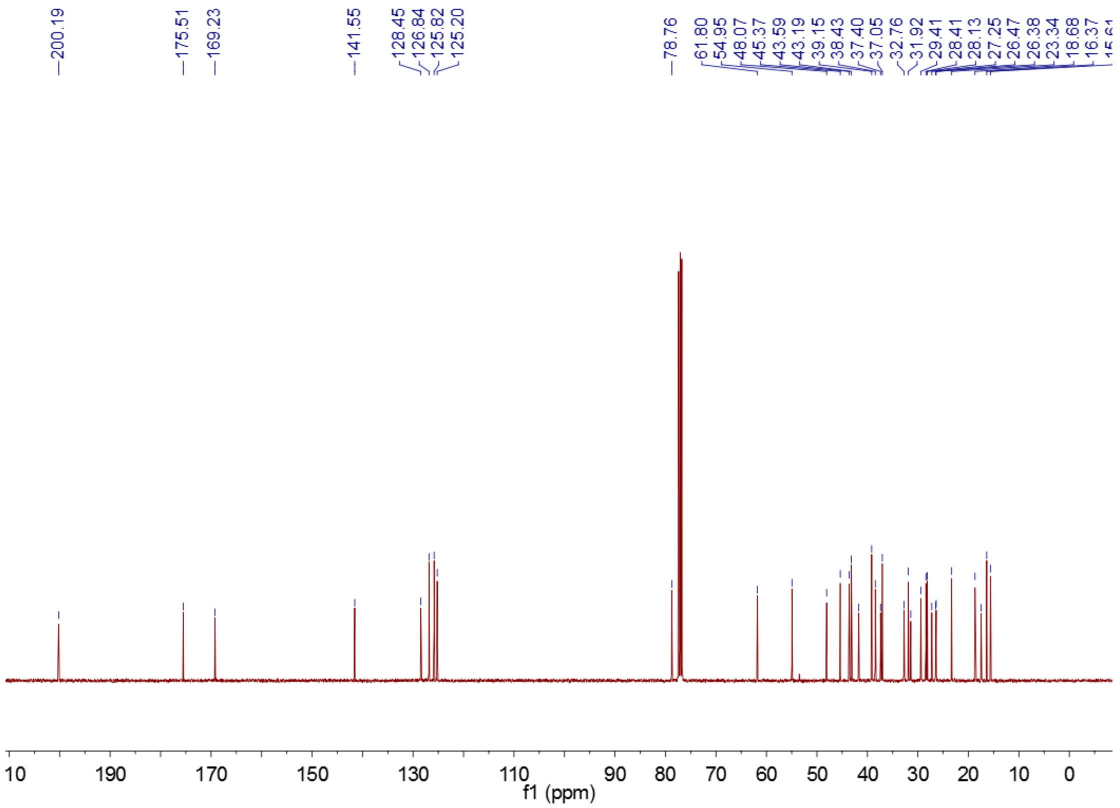

Figure S35. ^{13}C NMR spectrum ($CDCl_3$, 101 MHz) of target compound $\mathbf{A_{11}}$.

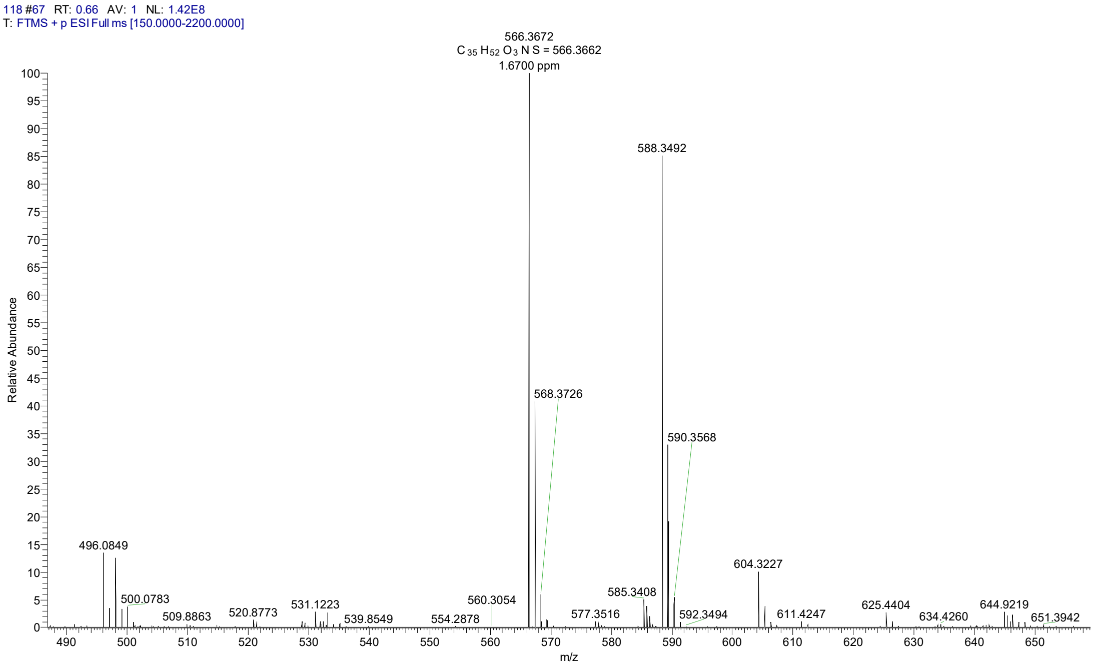

Figure S36. HRMS spectrum of target compound **A₁₁.**

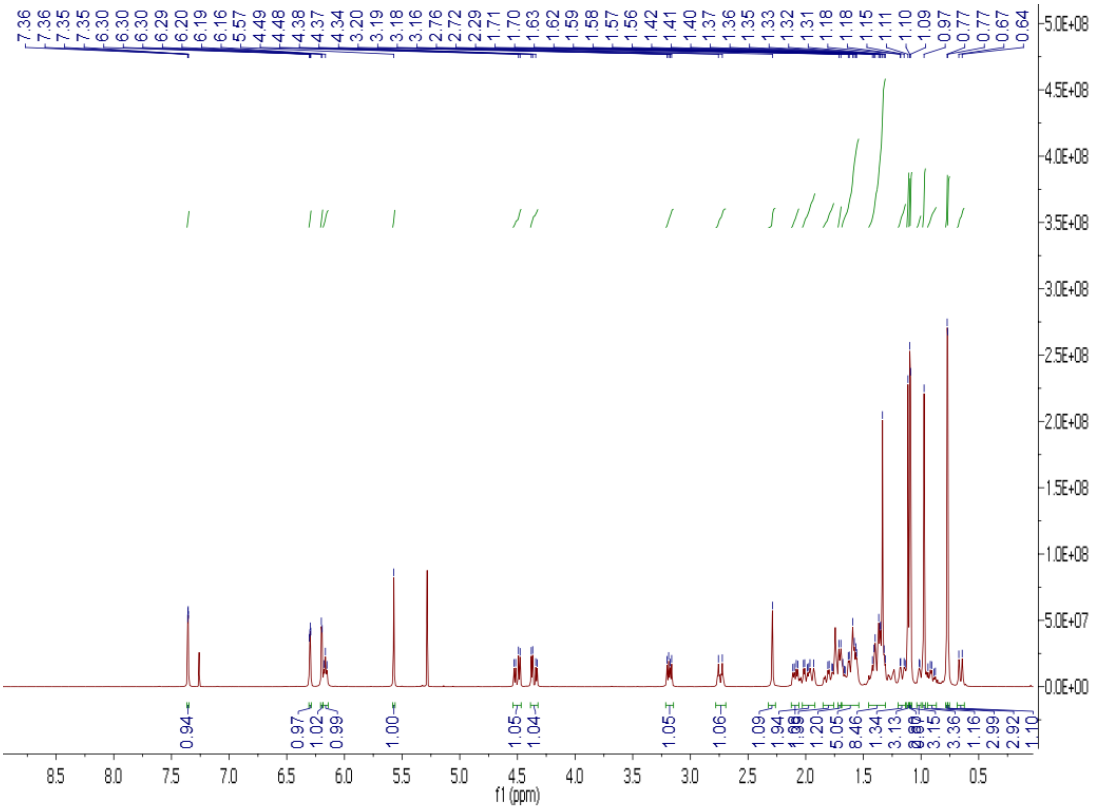

Figure S37. ^{1}H NMR spectrum ($CDCl_3$, 400 MHz) of target compound $\mathbf{A_{12}}$**.**

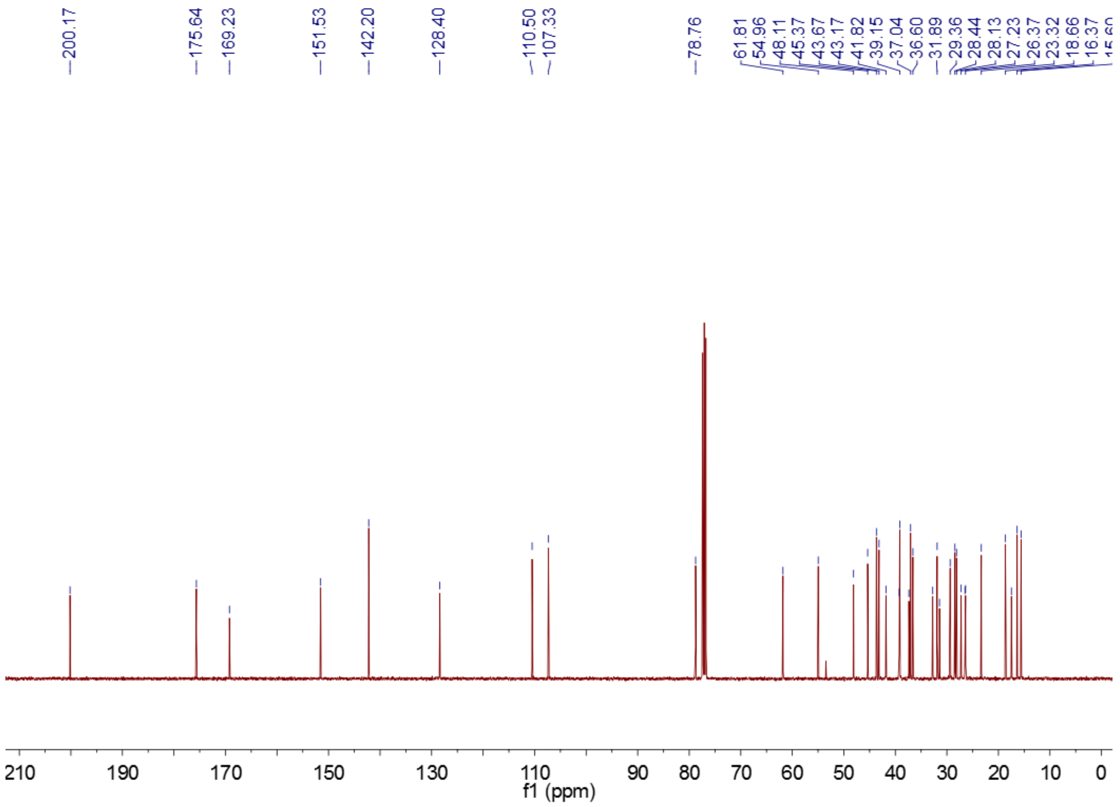

Figure S38. ^{13}C NMR spectrum ($CDCl_3$, 101 MHz) of target compound $\mathbf{A_{12}}$.

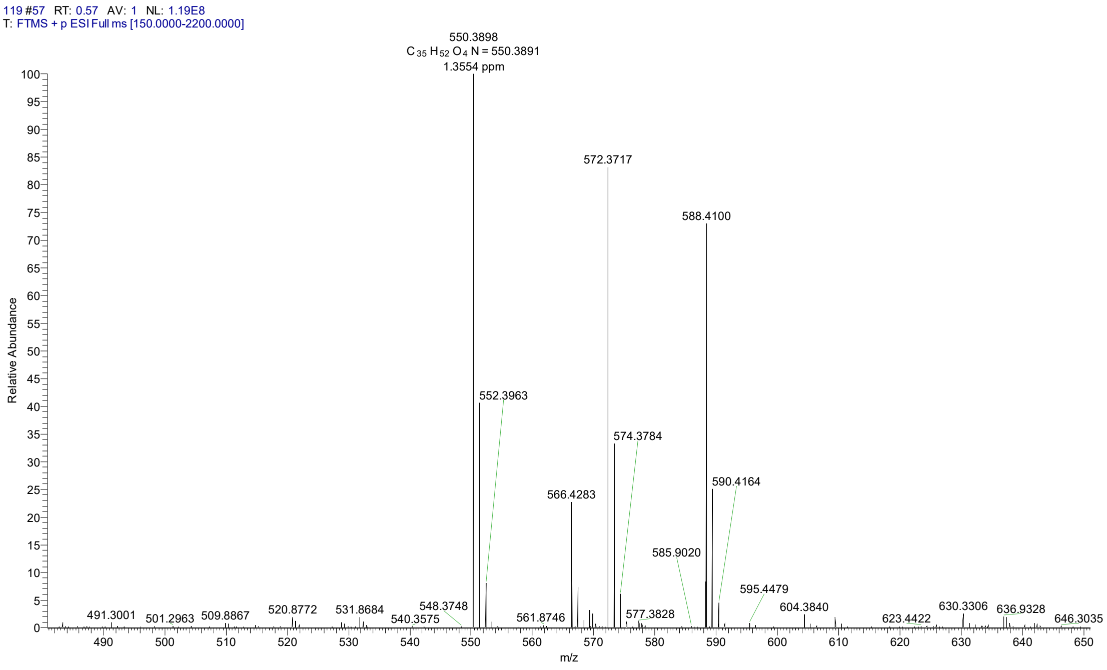

Figure S39. HRMS spectrum of target compound $\mathbf{A_{12}}$.

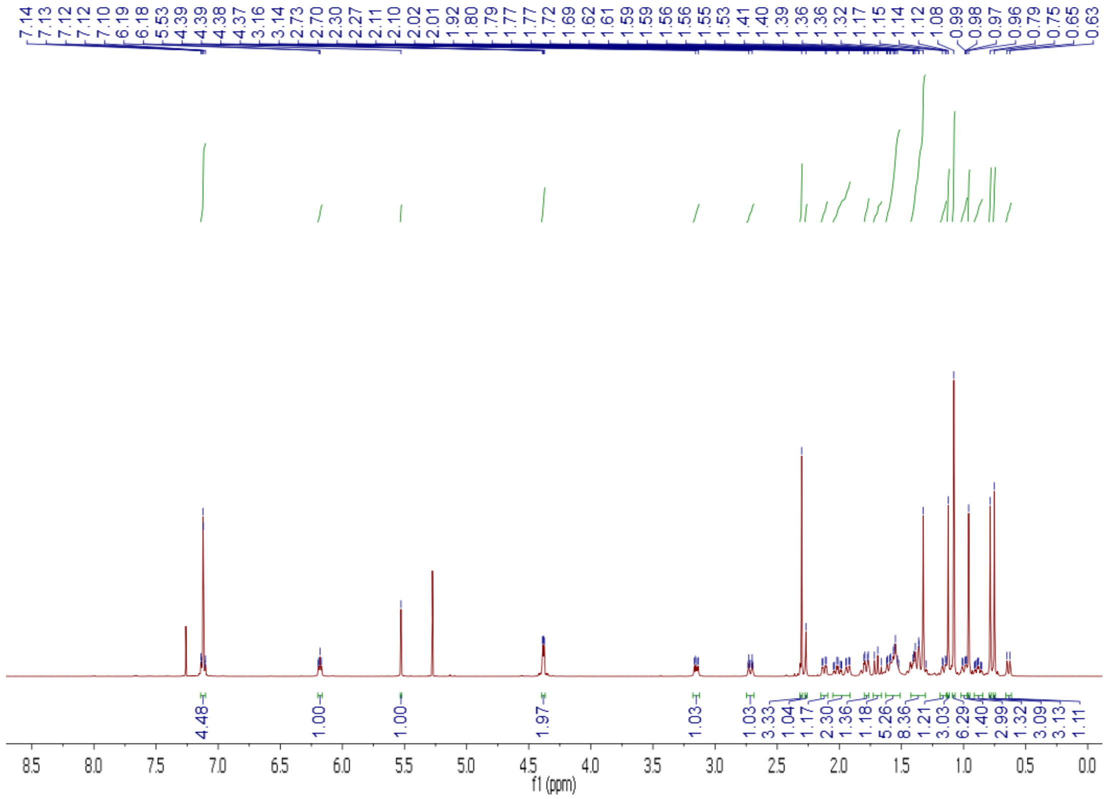

Figure S40. ^{1}H NMR spectrum ($CDCl_3$, 400 MHz) of target compound **A_{13}.**

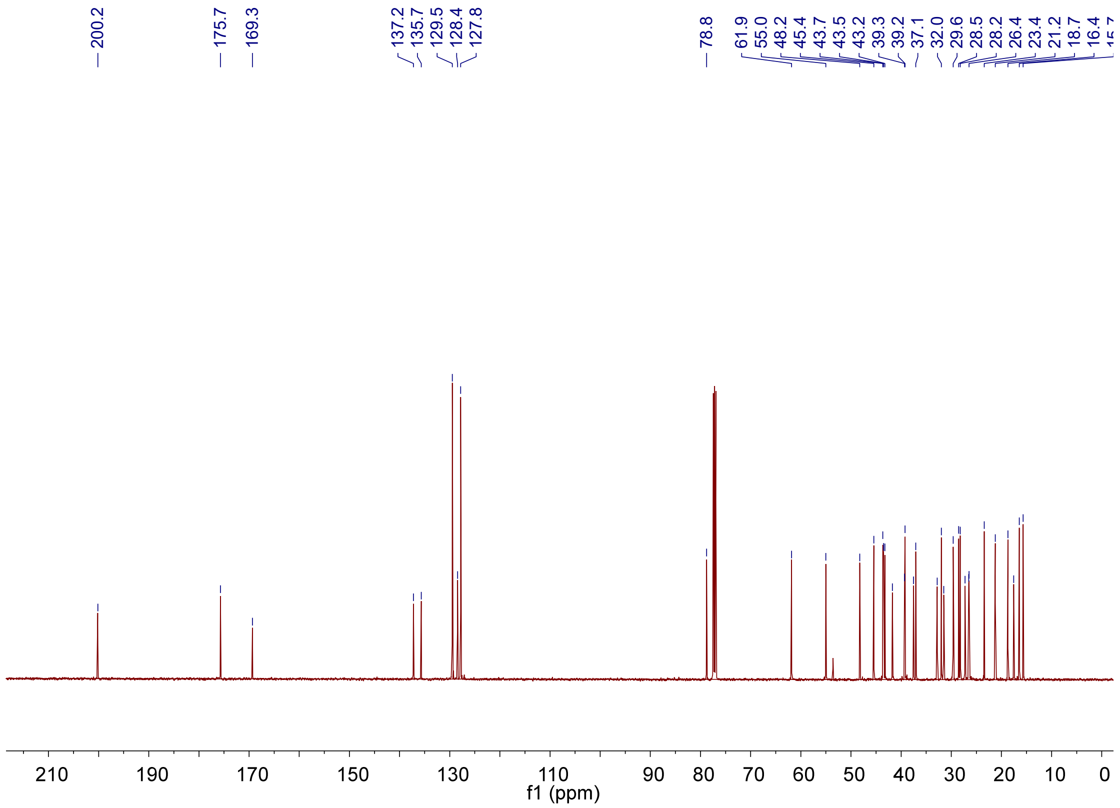

Figure S41. ^{13}C NMR spectrum ($CDCl_3$, 101 MHz) of target compound **A_{13}.**

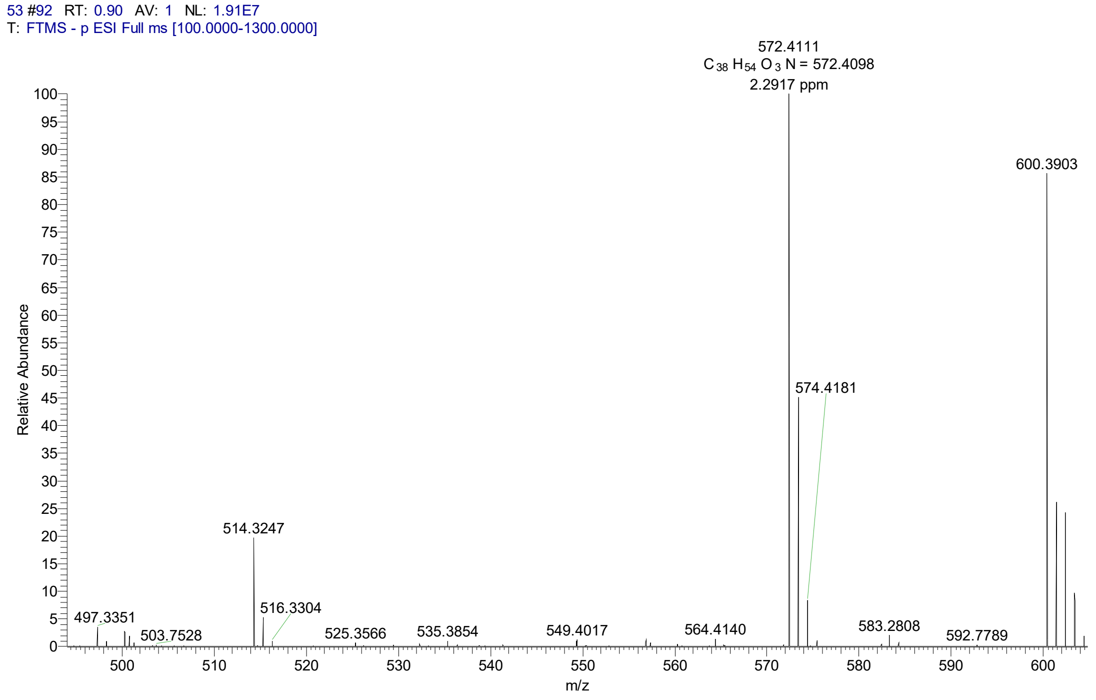

Figure S42. HRMS spectrum of target compound **A_{13}.**

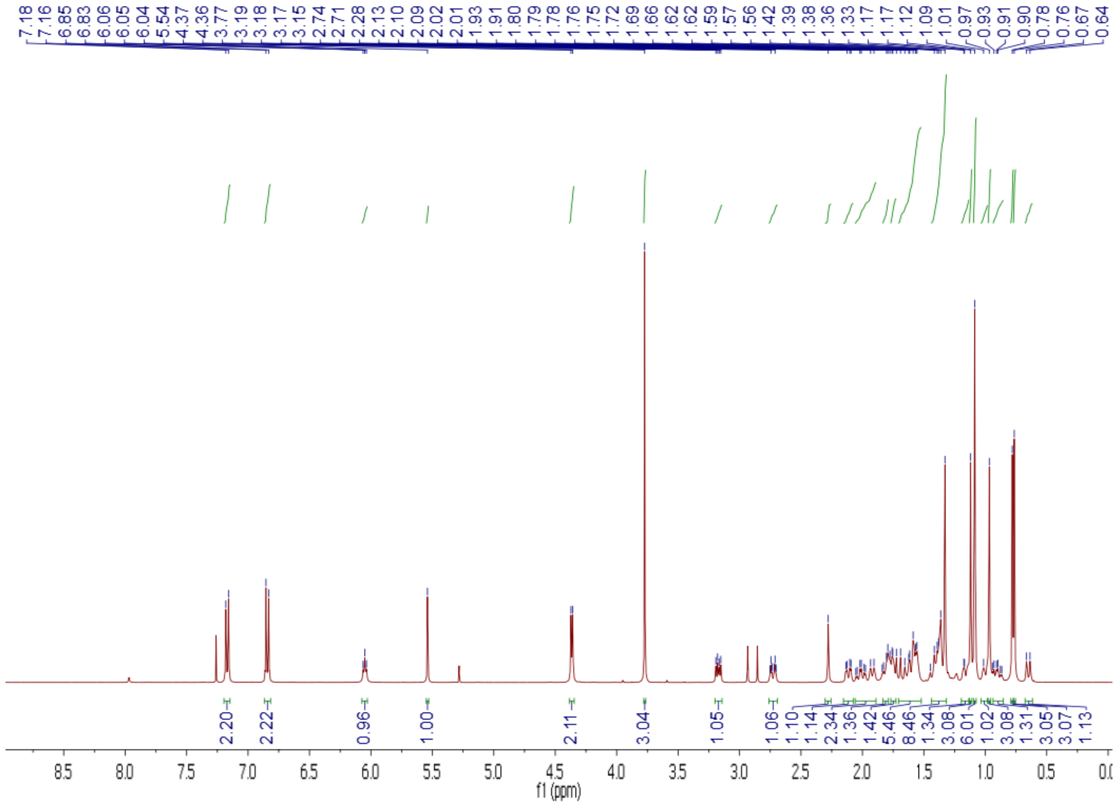

Figure S43. ^{1}H NMR spectrum ($CDCl_3$, 400 MHz) of target compound **A_{14}.**

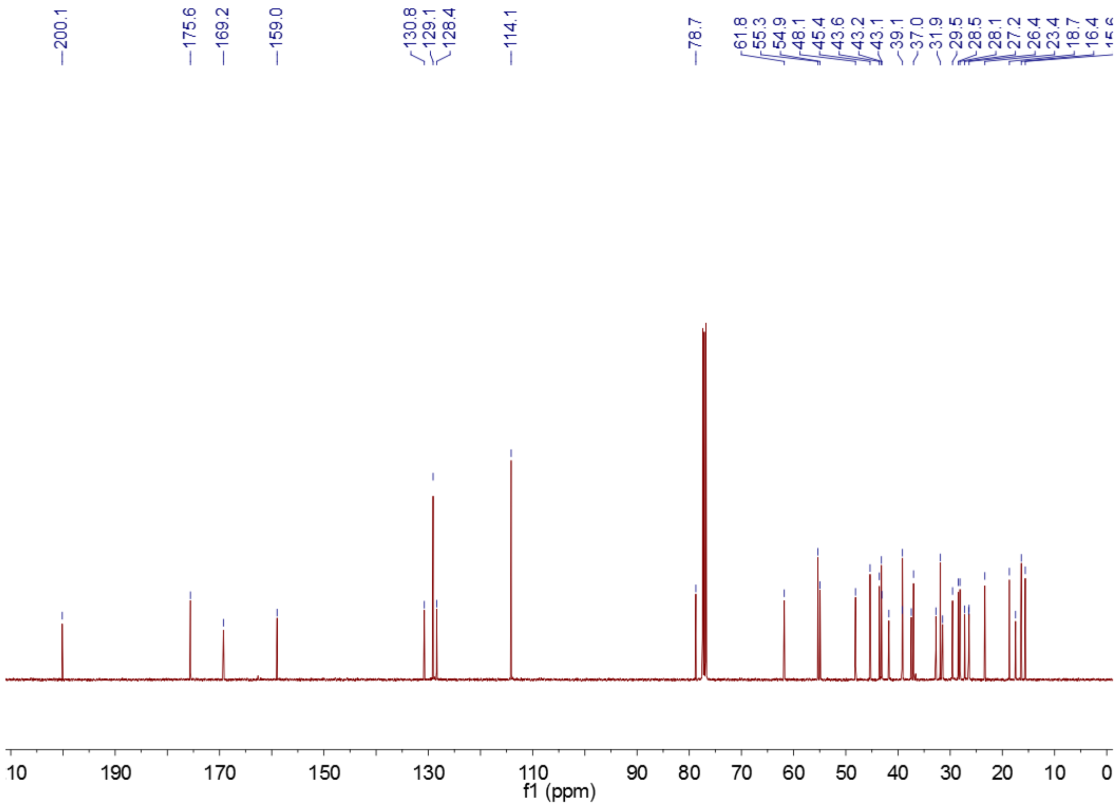

Figure S44. ^{13}C NMR spectrum ($CDCl_3$, 101 MHz) of target compound $\mathbf{A_{14}}$.

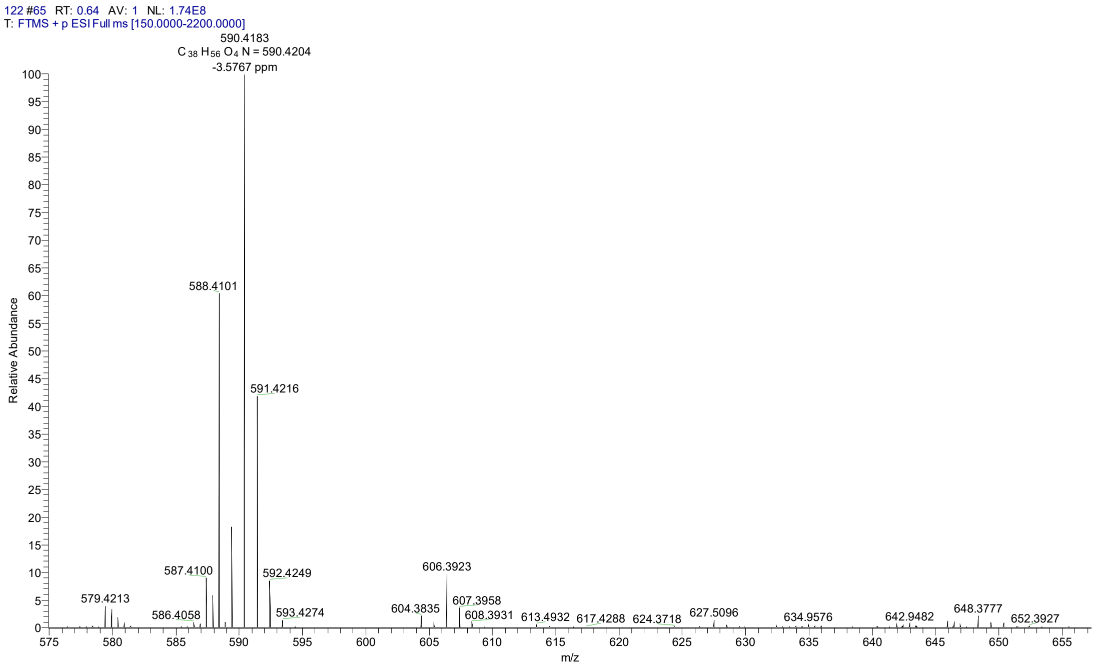

Figure S45. HRMS spectrum of target compound $\mathbf{A_{14}}$.

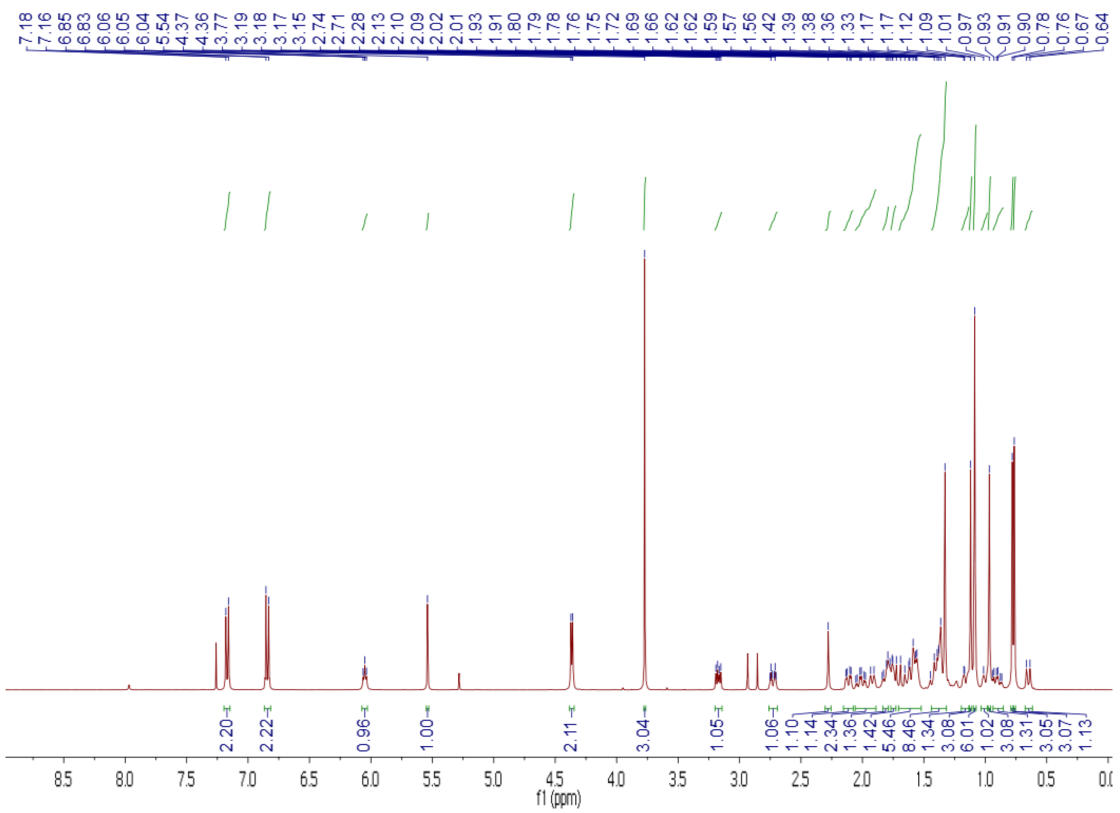

Figure S46. ^{1}H NMR spectrum ($CDCl_3$, 400 MHz) of target compound $\mathbf{A_{15}}$.

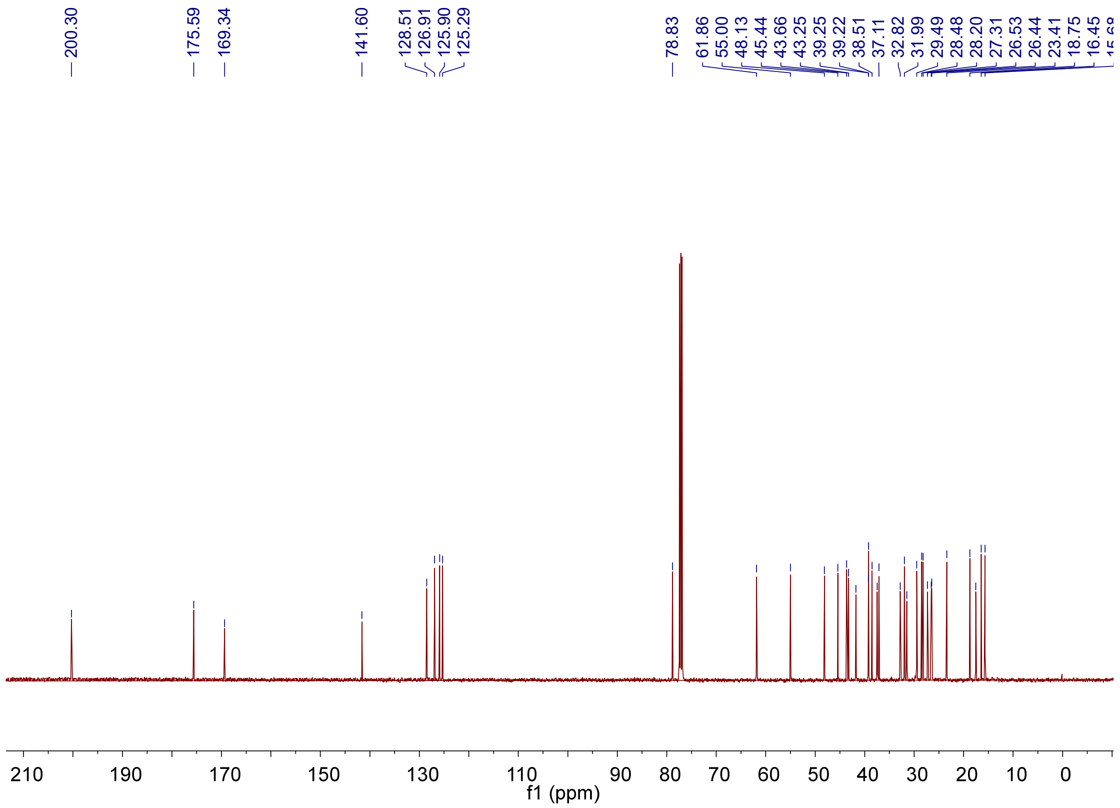

Figure S47. ^{13}C NMR spectrum ($CDCl_3$, 101 MHz) of target compound $\mathbf{A_{15}}$.

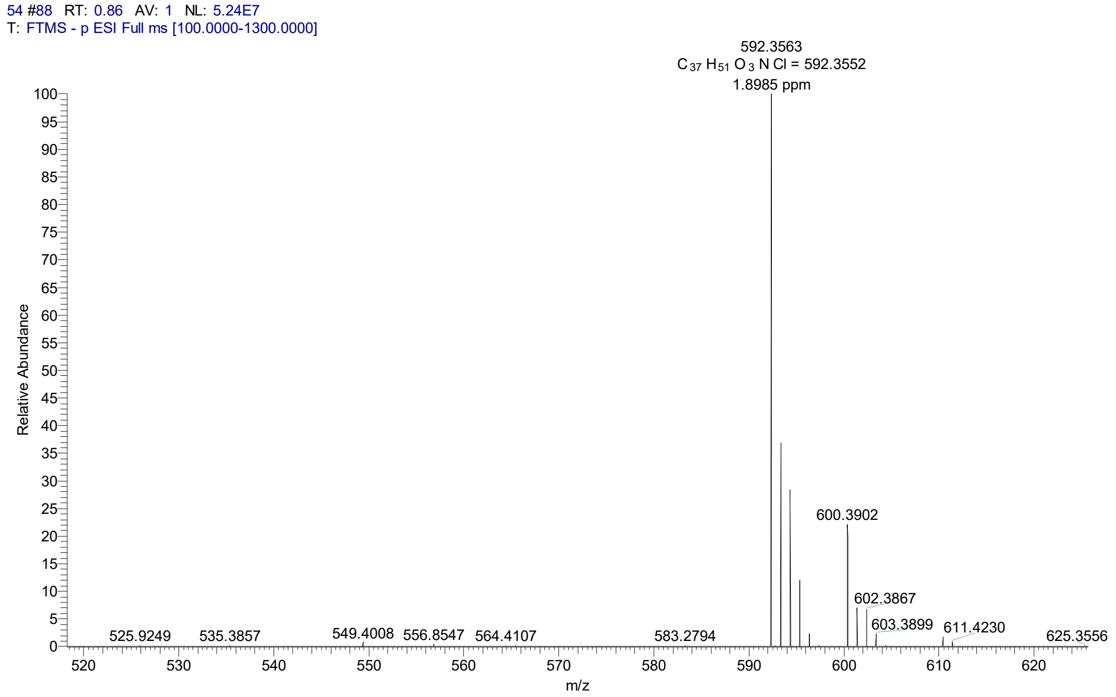

Figure S48. HRMS spectrum of target compound **A$_{15}$.**

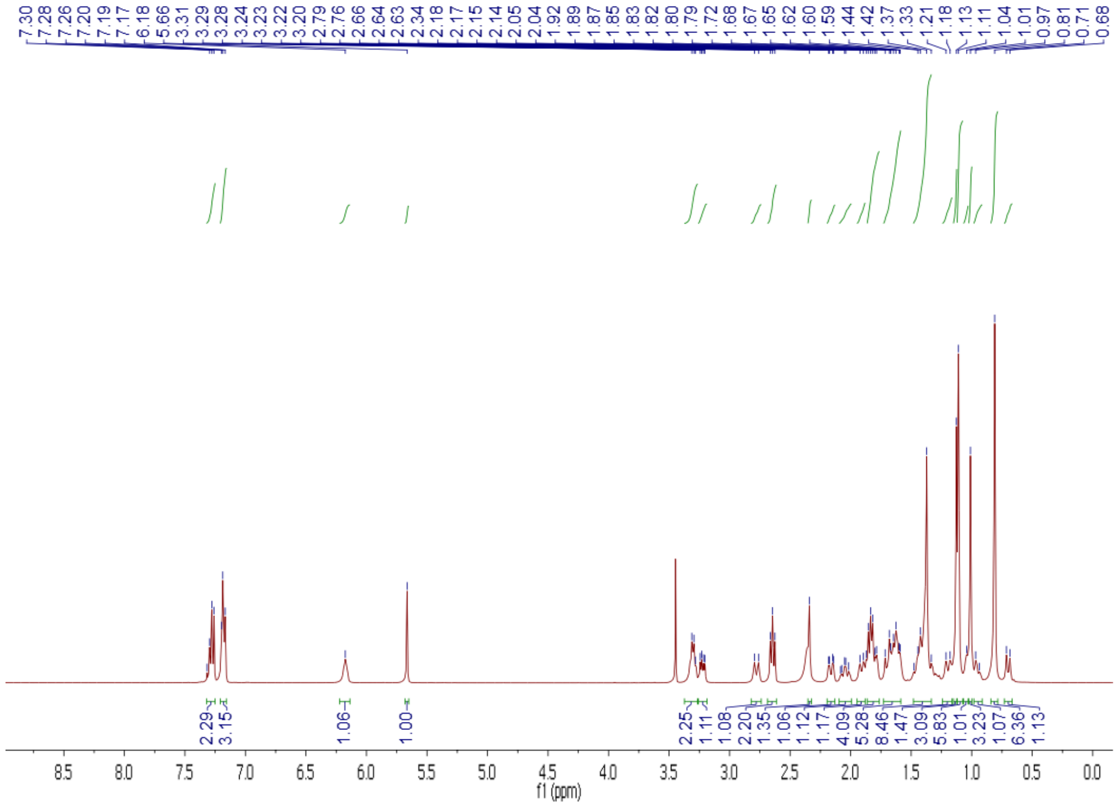

Figure S49. ^{1}H NMR spectrum (CDCl$_3$, 400 MHz) of target compound **A$_{16}$.**

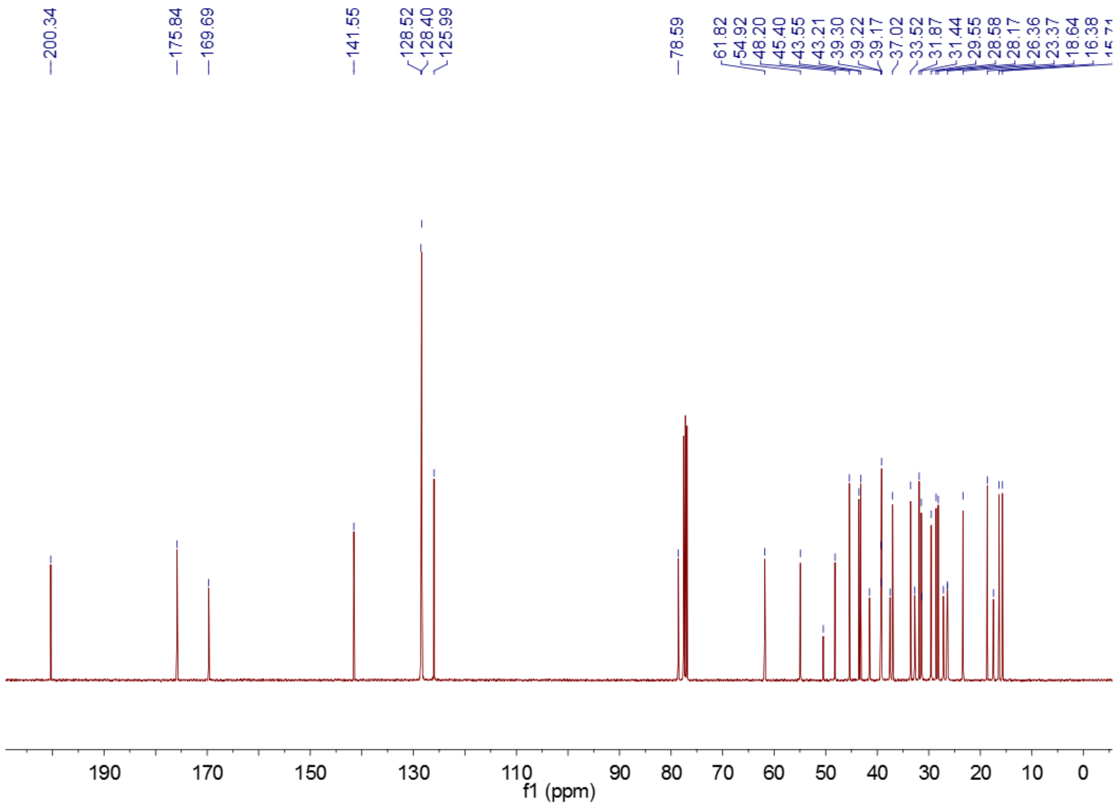

Figure S50. ^{13}C NMR spectrum ($CDCl_3$, 101 MHz) of target compound $\mathbf{A_{16}}$.

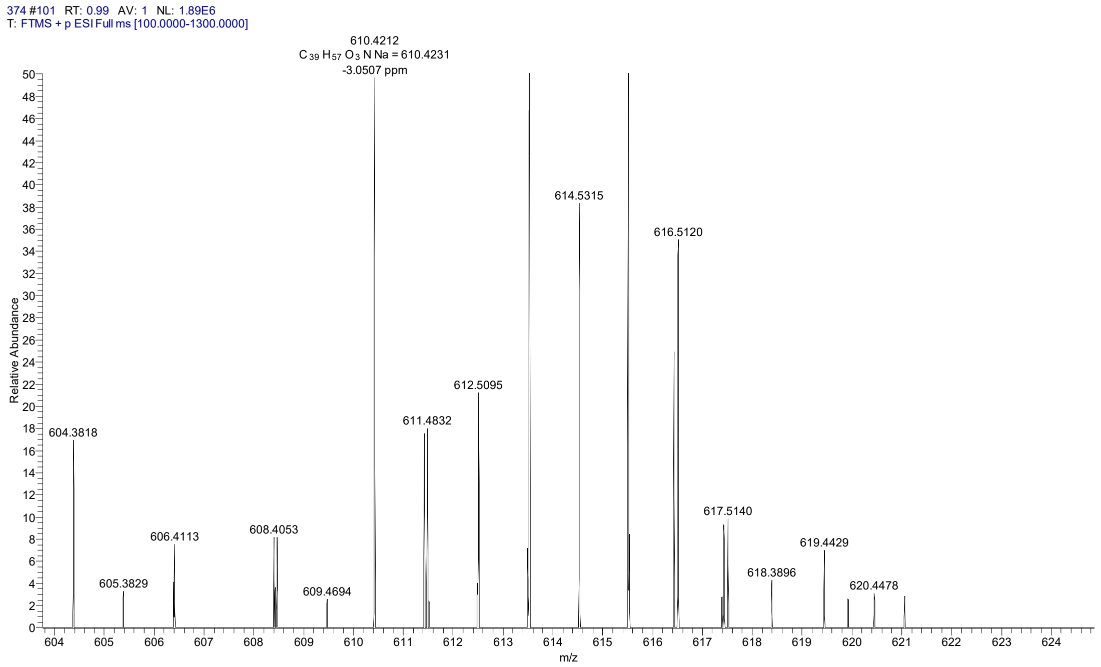

Figure S51. HRMS spectrum of target compound $\mathbf{A_{16}}$.

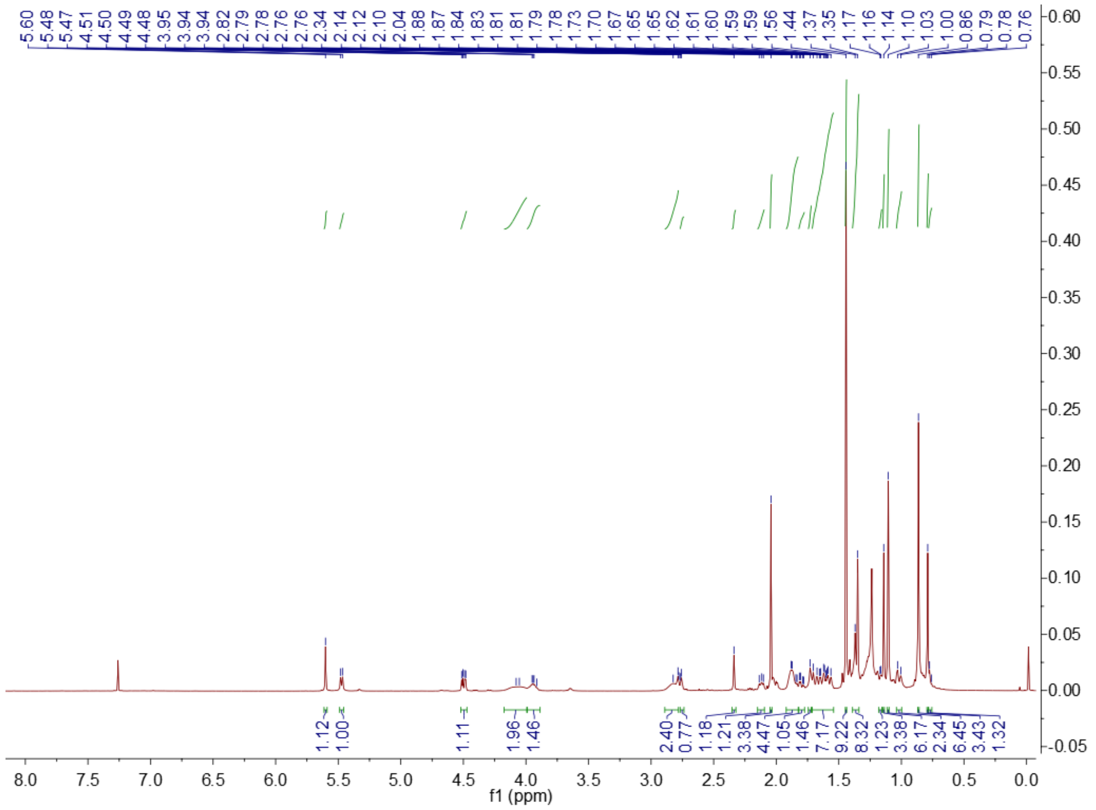

Figure S52. ^{1}H NMR spectrum ($CDCl_3$, 500 MHz) of target compound $\mathbf{A_{17}}$.

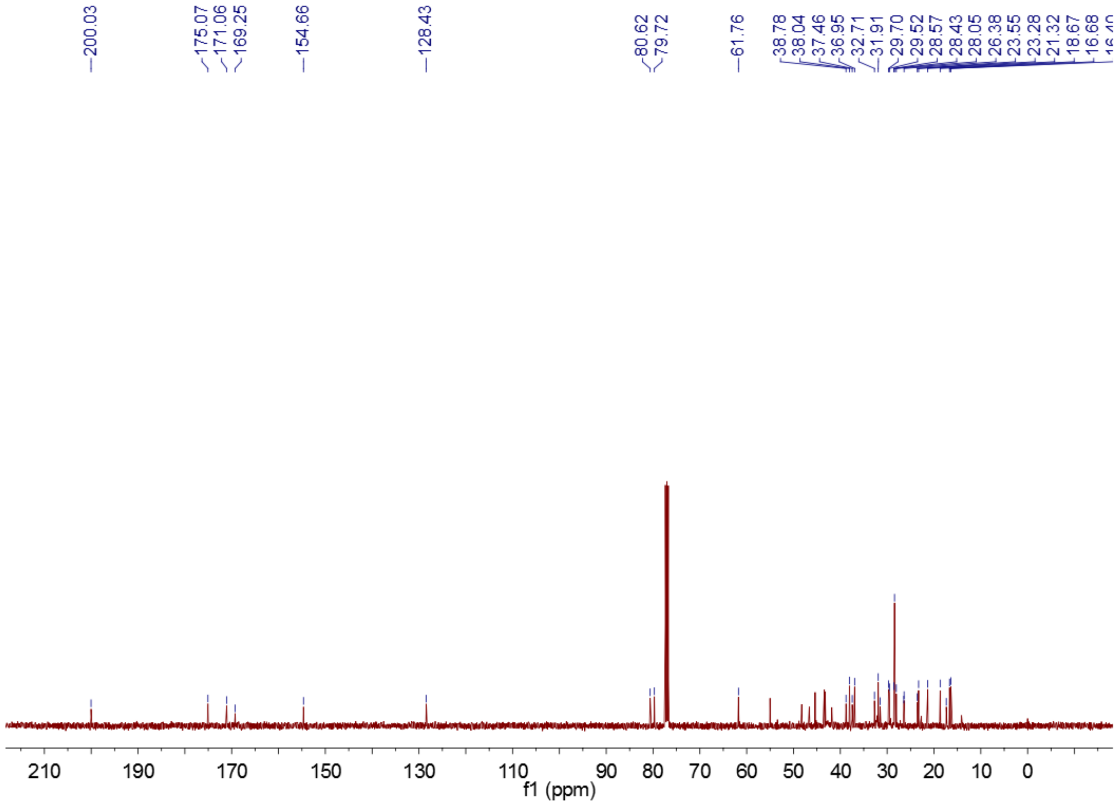

Figure S53. ^{13}C NMR spectrum ($CDCl_3$, 126 MHz) of target compound $\mathbf{A_{17}}$.

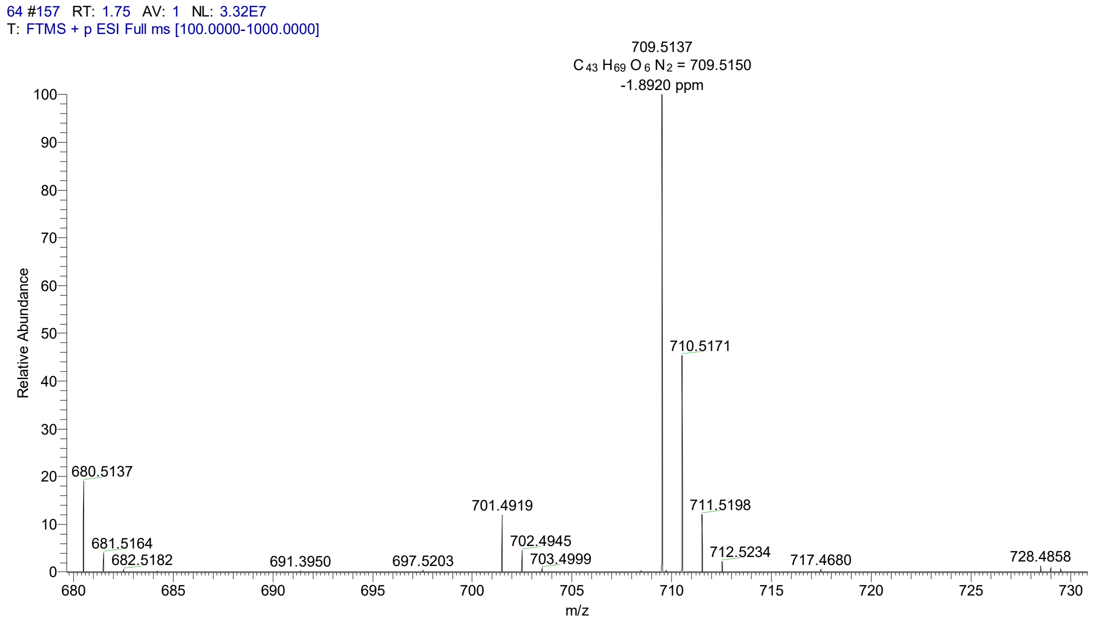

Figure S54. HRMS spectrum of target compound $\mathbf{A_{17}}$.

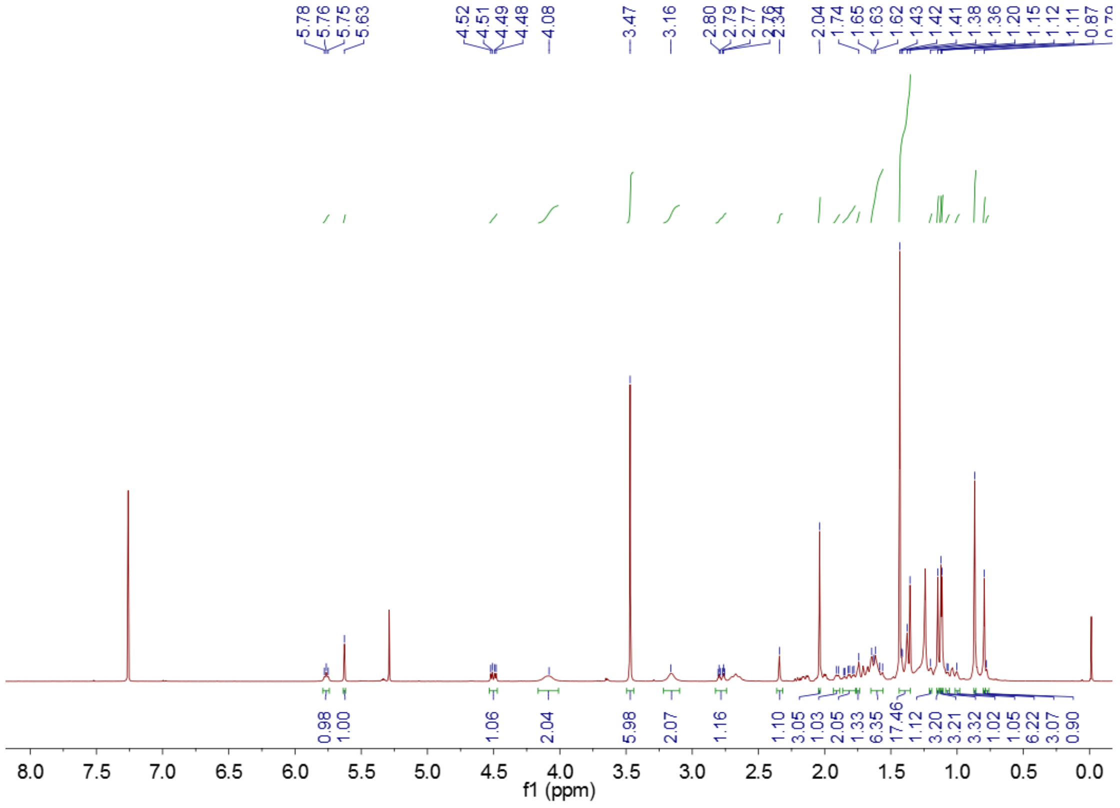

Figure S55. ^{1}H NMR spectrum ($CDCl_3$, 400 MHz) of target compound $\mathbf{A_{18}}$.

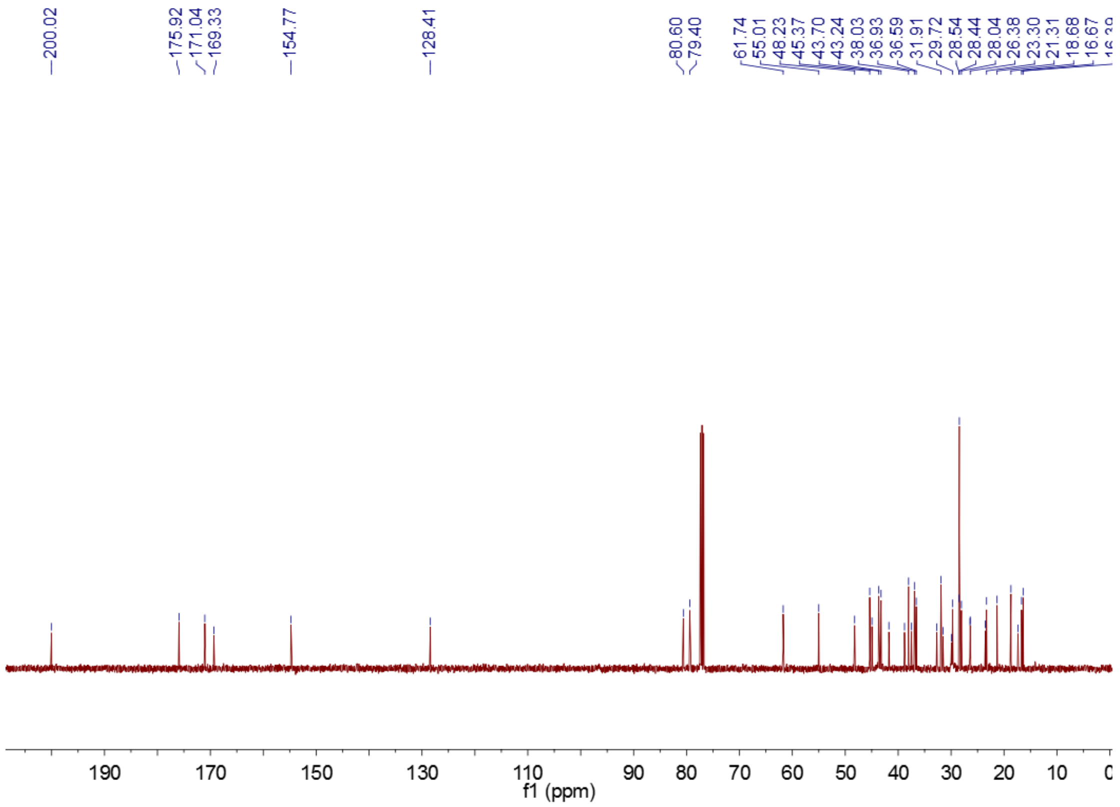

Figure S56. ^{13}C NMR spectrum ($CDCl_3$, 101 MHz) of target compound **A_{18}.**

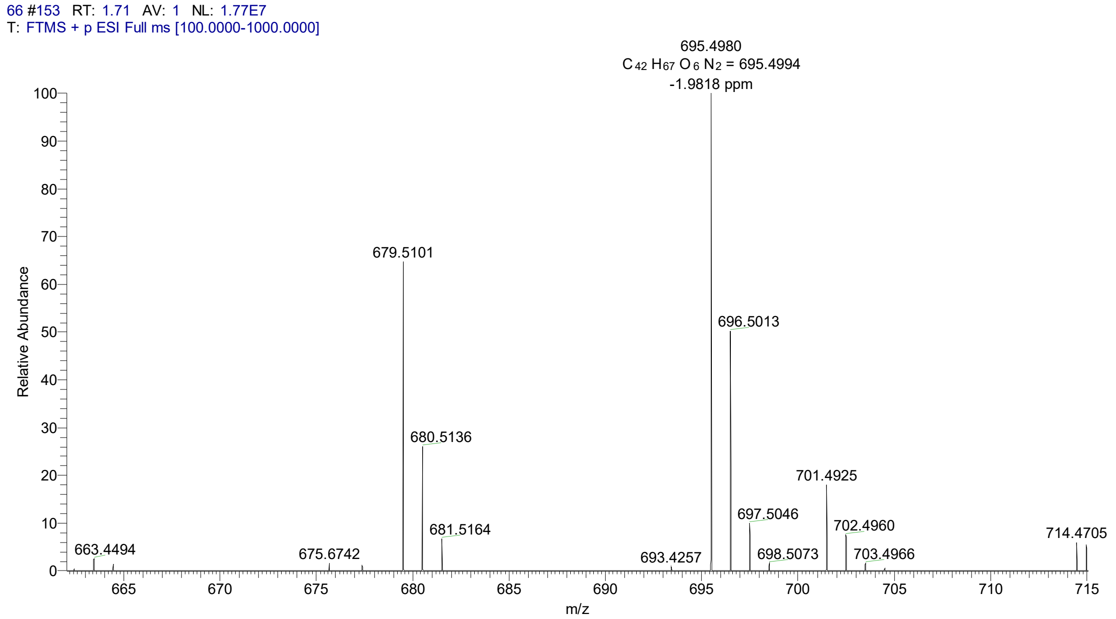

Figure S57. HRMS spectrum of target compound **A_{18}.**

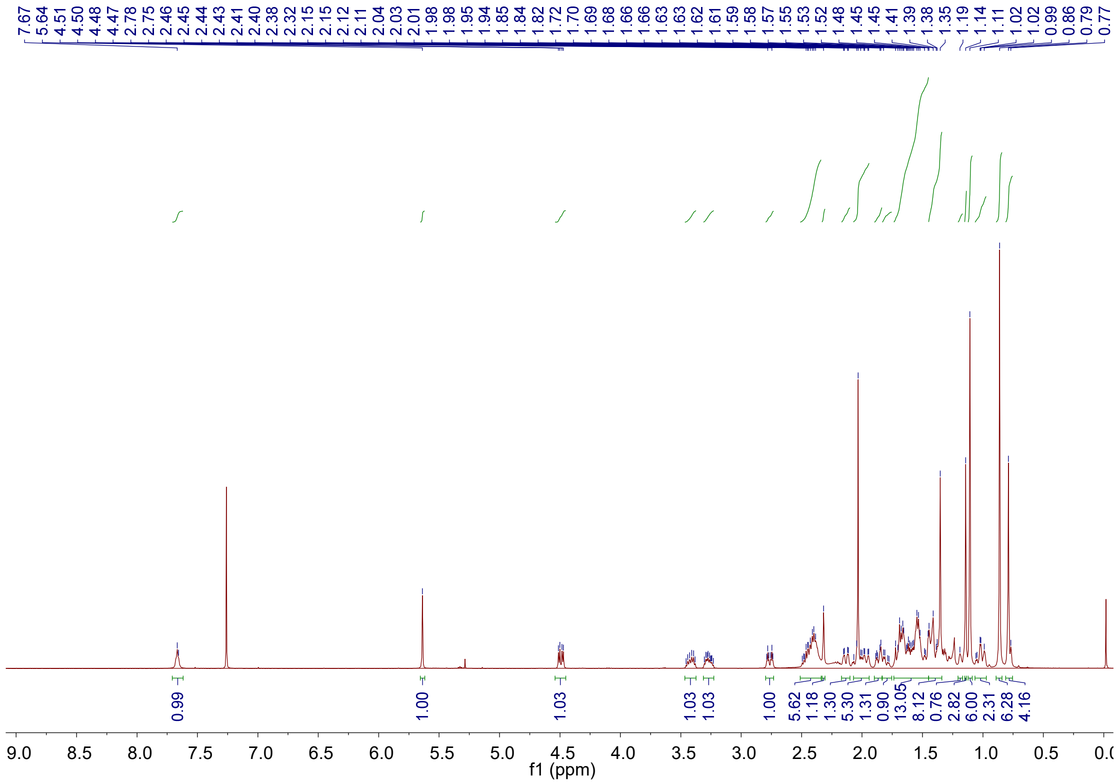

Figure S58. ^{1}H NMR spectrum ($CDCl_3$, 400 MHz) of target compound **A_{19}.**

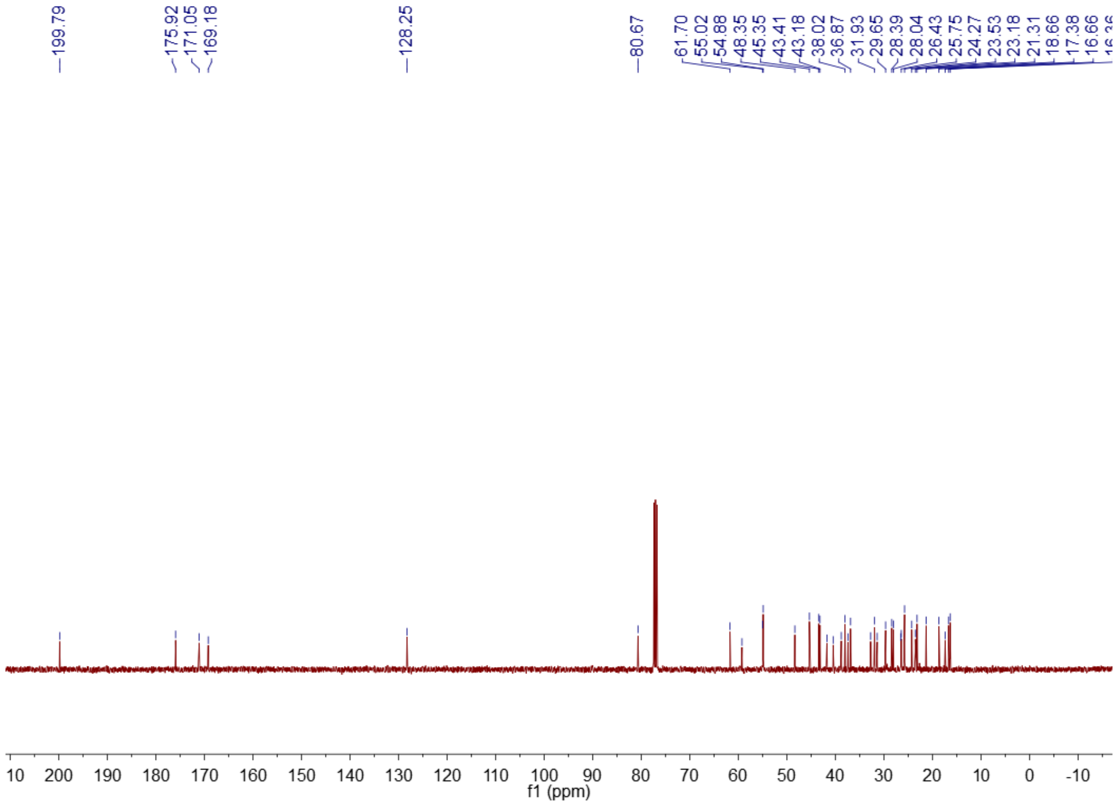

Figure S59. ^{13}C NMR spectrum ($CDCl_3$, 101 MHz) of target compound **A_{19}.**

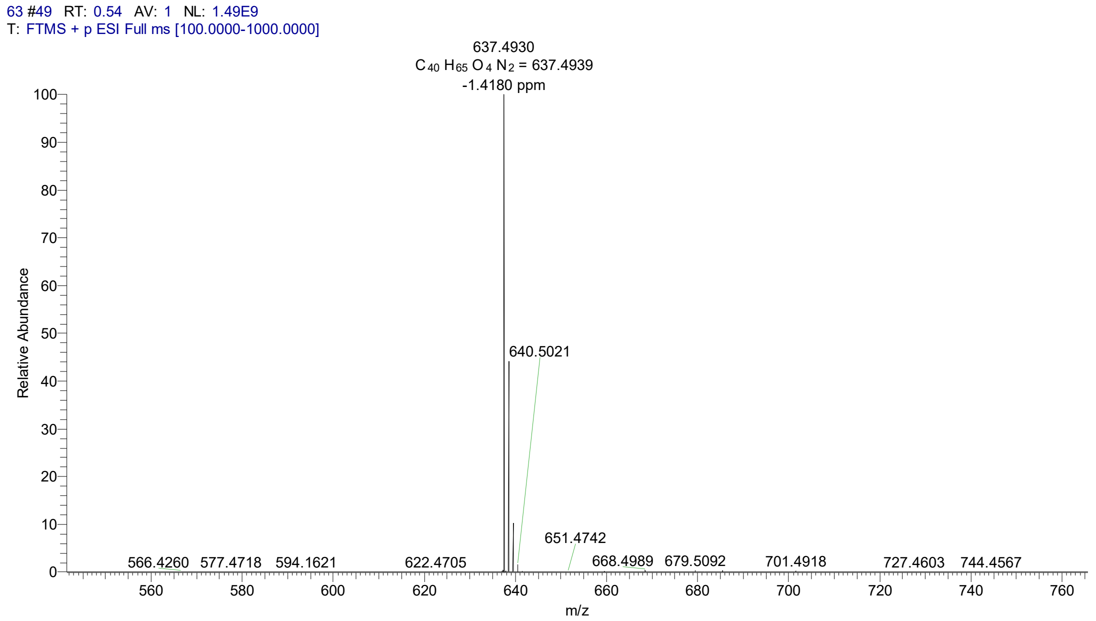

Figure S60. HRMS spectrum of target compound **A19.**

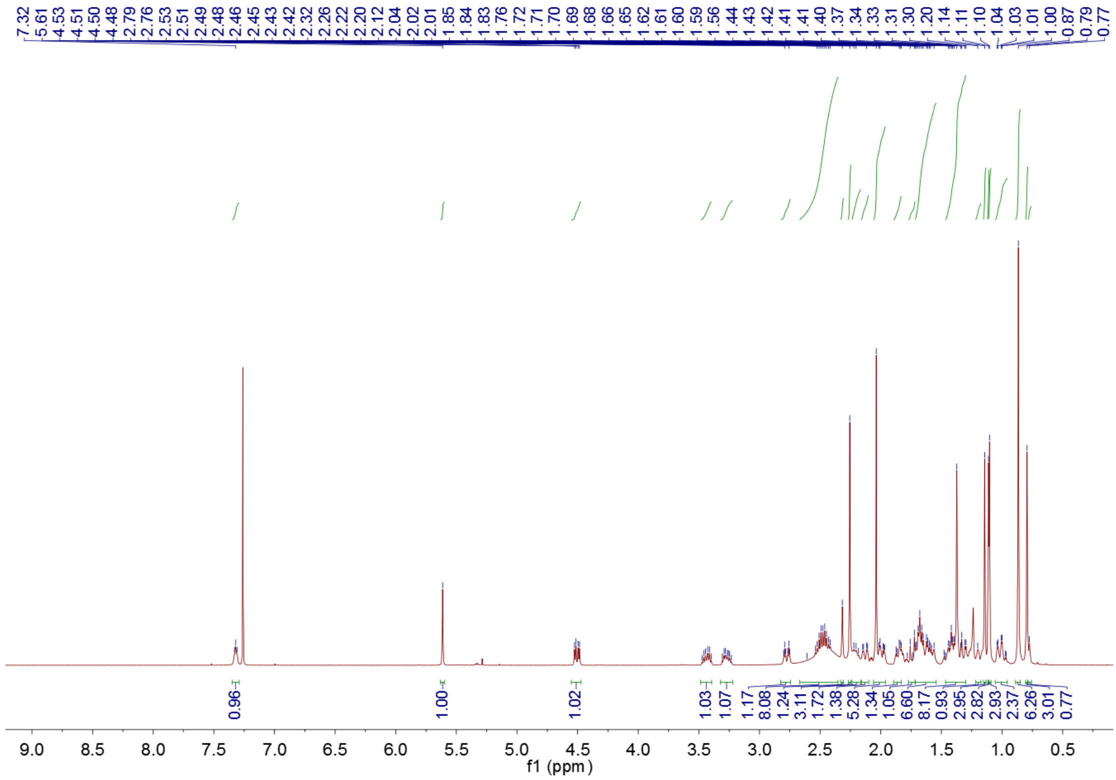

Figure S61. ^{1}H NMR spectrum ($CDCl_3$, 400 MHz) of target compound **A20.**

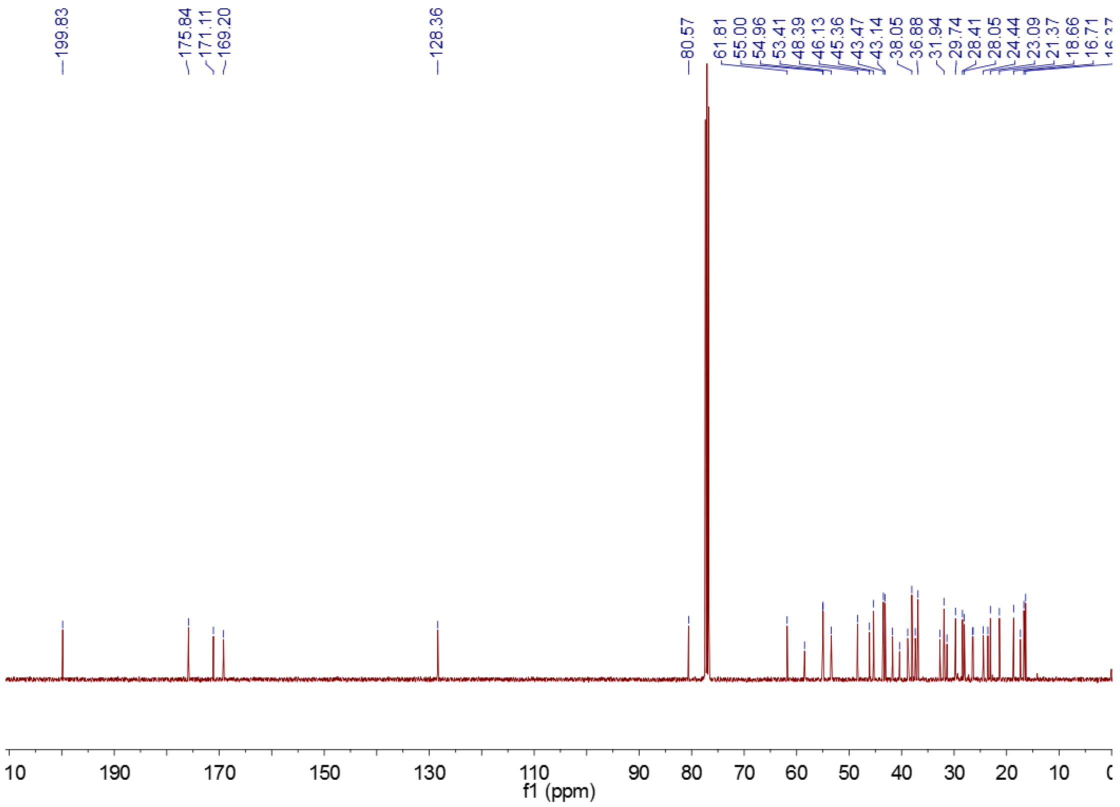

Figure S62. ^{13}C NMR spectrum ($CDCl_3$, 101 MHz) of target compound $\mathbf{A_{20}}$.

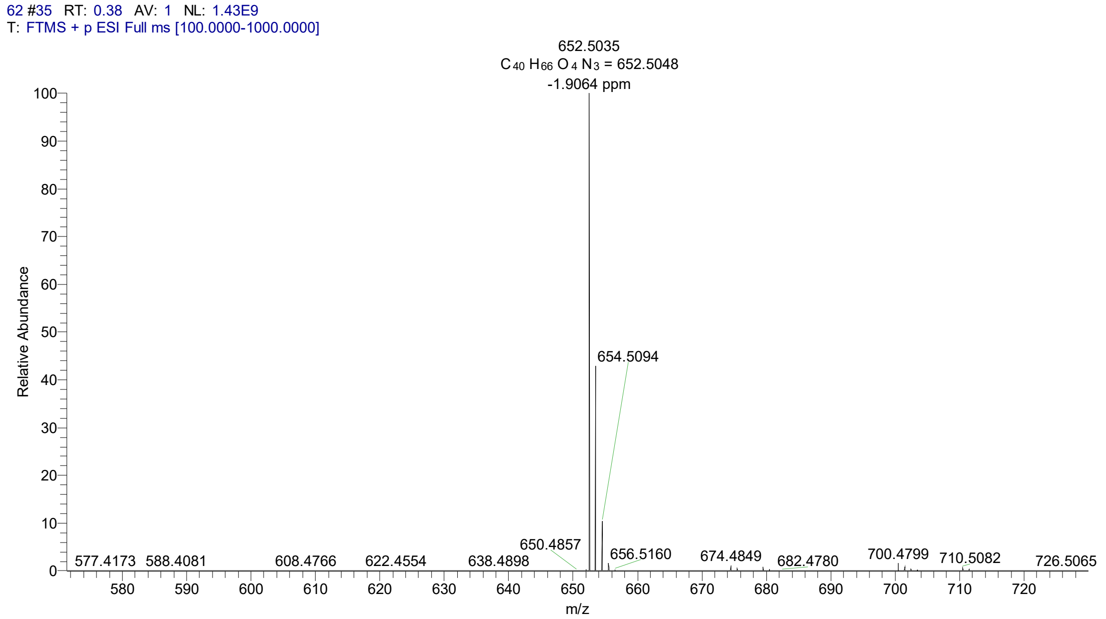

Figure S63. HRMS spectrum of target compound $\mathbf{A_{20}}$.

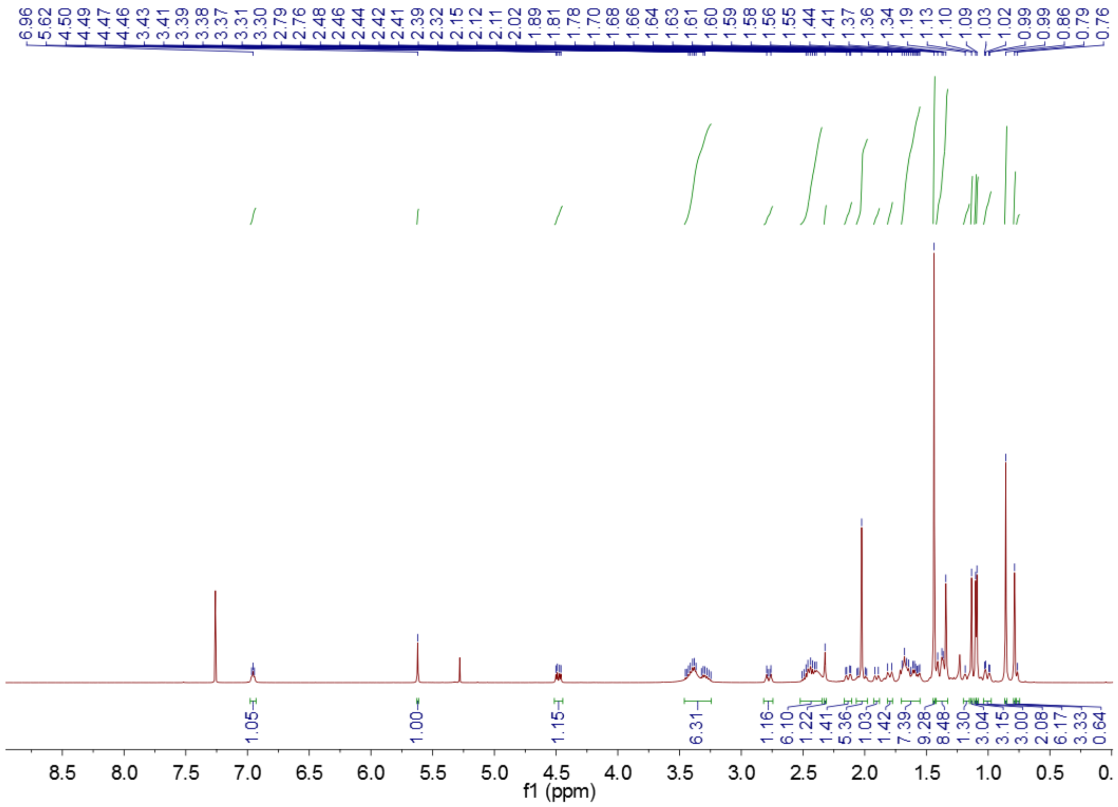

Figure S64. ^{1}H NMR spectrum ($CDCl_3$, 400 MHz) of target compound $\mathbf{A_{21}}$.

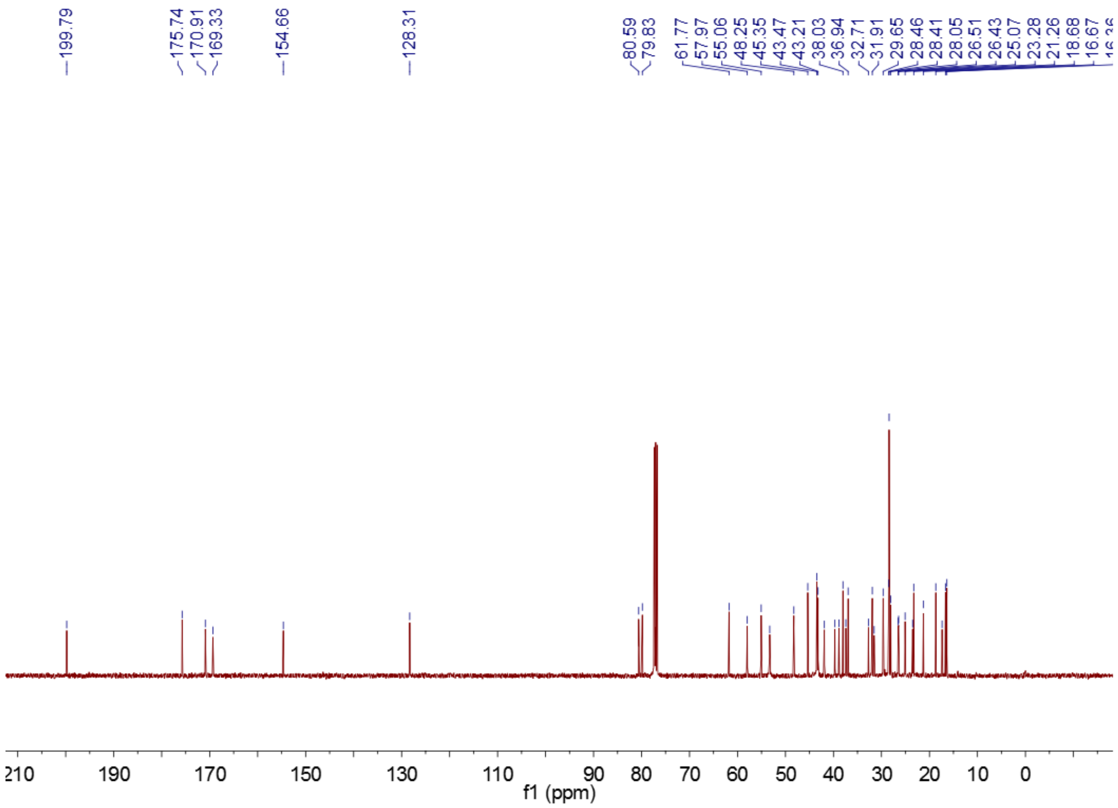

Figure S65. ^{13}C NMR spectrum ($CDCl_3$, 101 MHz) of target compound $\mathbf{A_{21}}$.

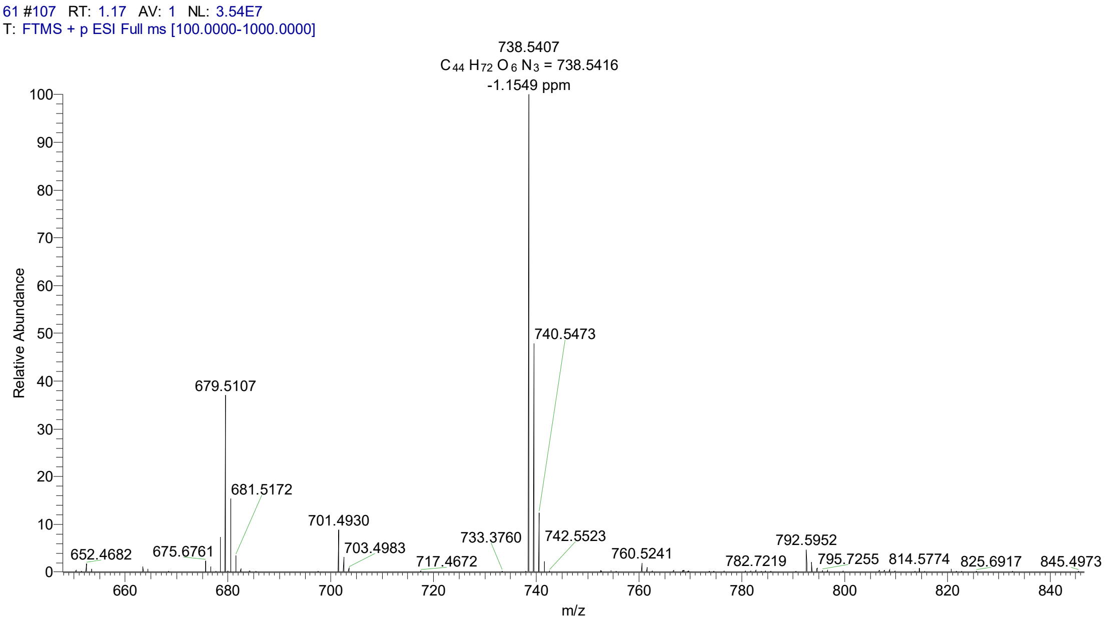

Figure S66. HRMS spectrum of target compound $\mathbf{A_{21}}$.

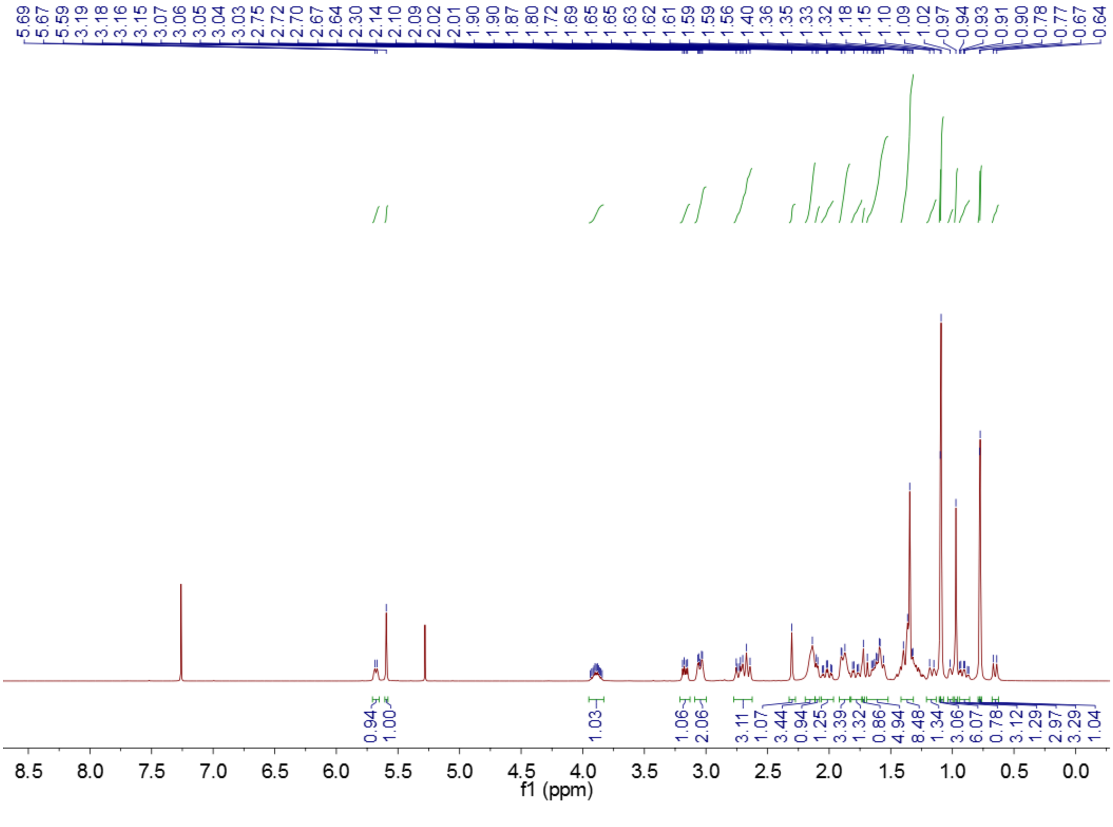

Figure S67. ^{1}H NMR spectrum ($CDCl_3$, 400 MHz) of target compound $\mathbf{A_{22}}$.

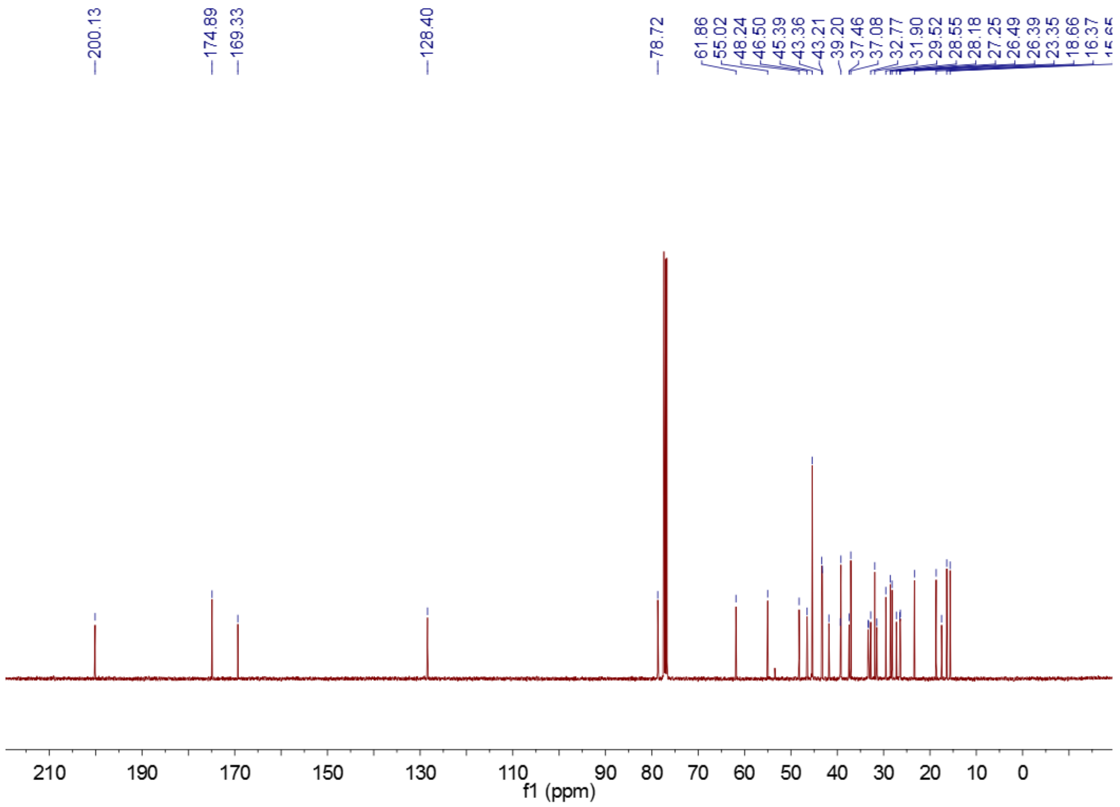

Figure S68. ^{13}C NMR spectrum ($CDCl_3$, 101 MHz) of target compound $\mathbf{A_{22}}$.

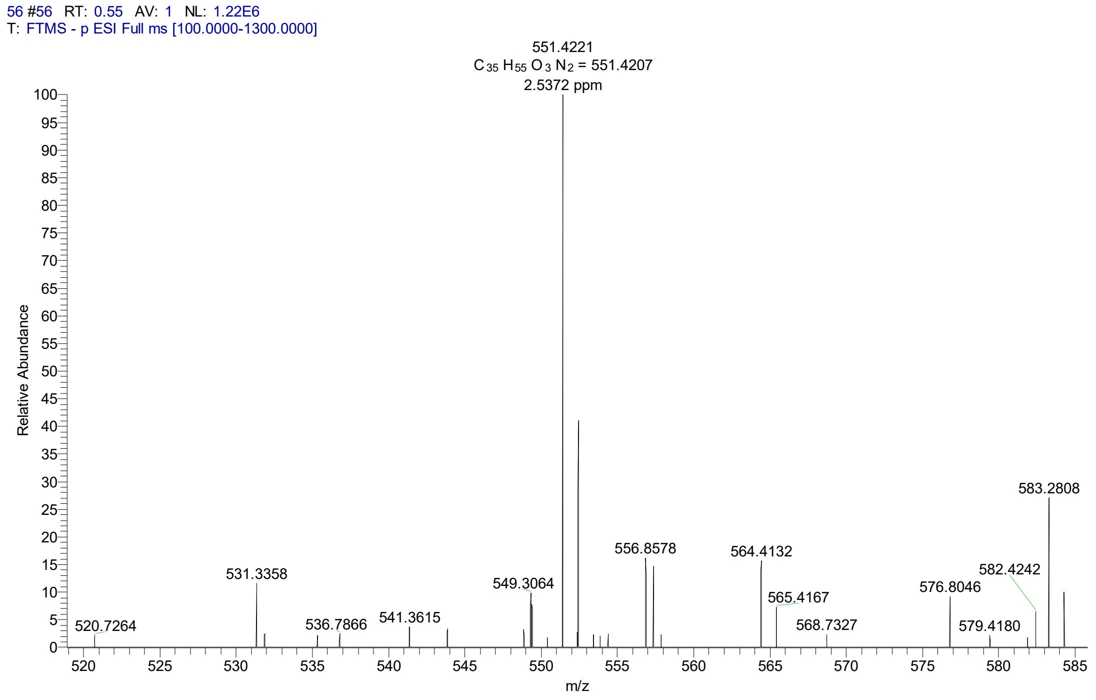

Figure S69. HRMS spectrum of target compound $\mathbf{A_{22}}$.

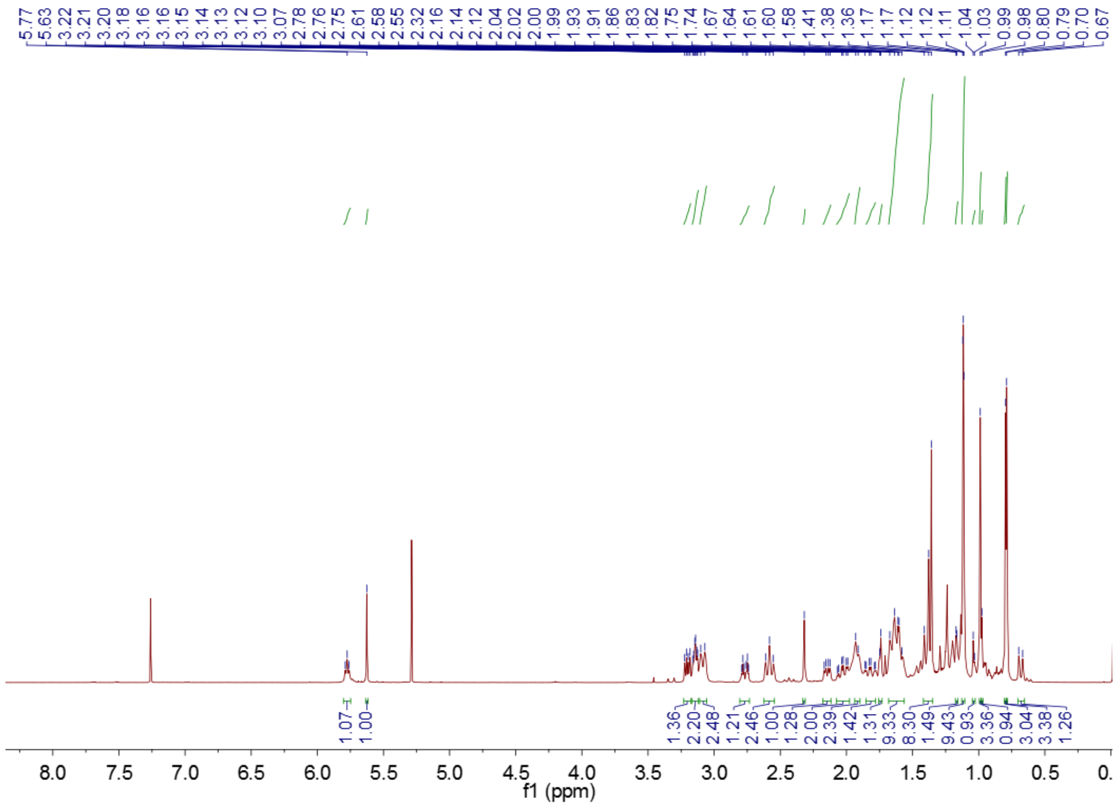

Figure S70. ^{1}H NMR spectrum ($CDCl_3$, 400 MHz) of target compound $\mathbf{A_{23}}$.

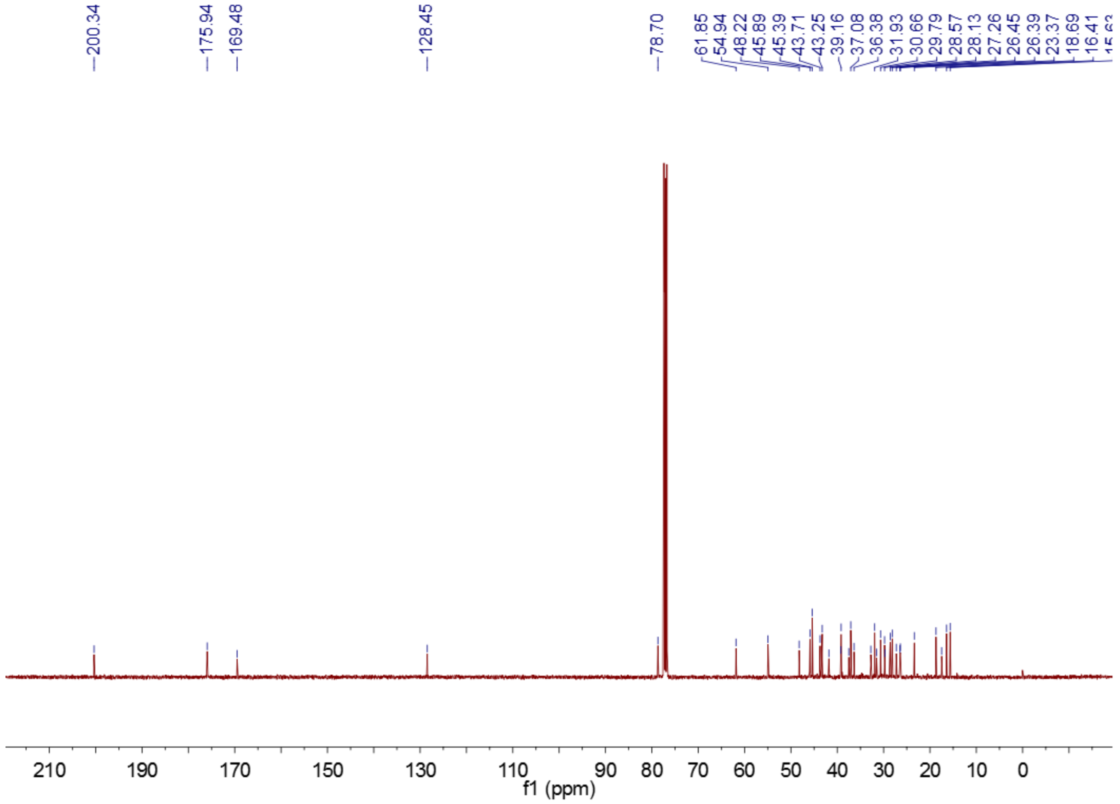

Figure S71. ^{13}C NMR spectrum ($CDCl_3$, 101 MHz) of target compound $\mathbf{A_{23}}$.

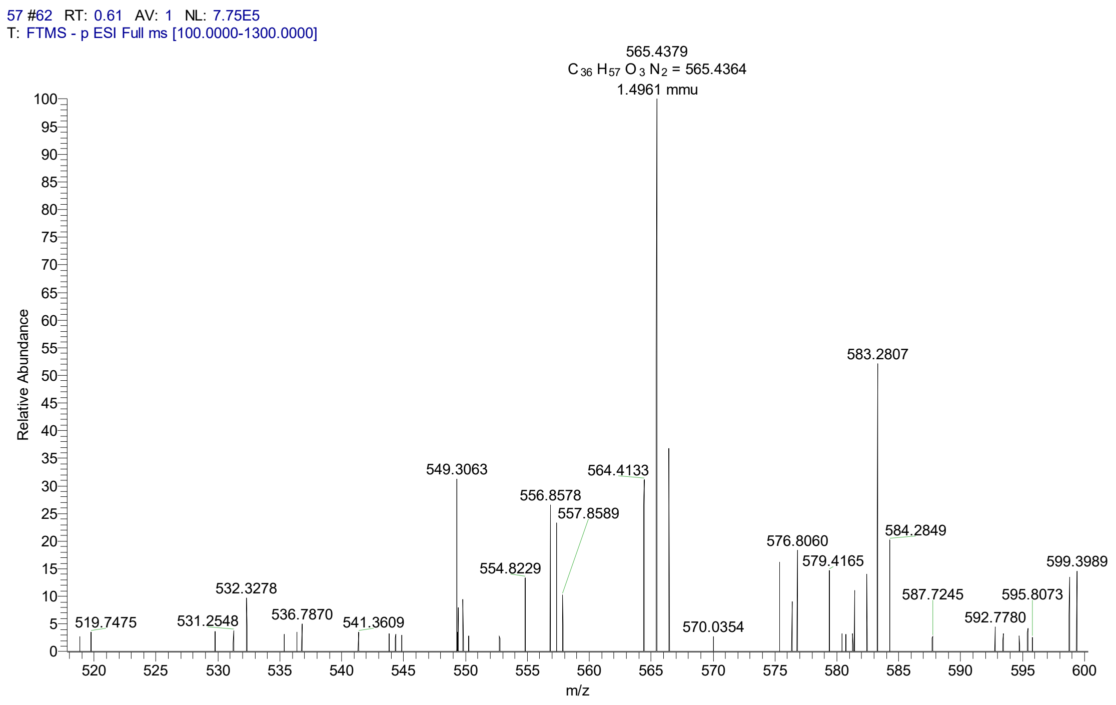

Figure S72. HRMS spectrum of target compound **A₂₃.**

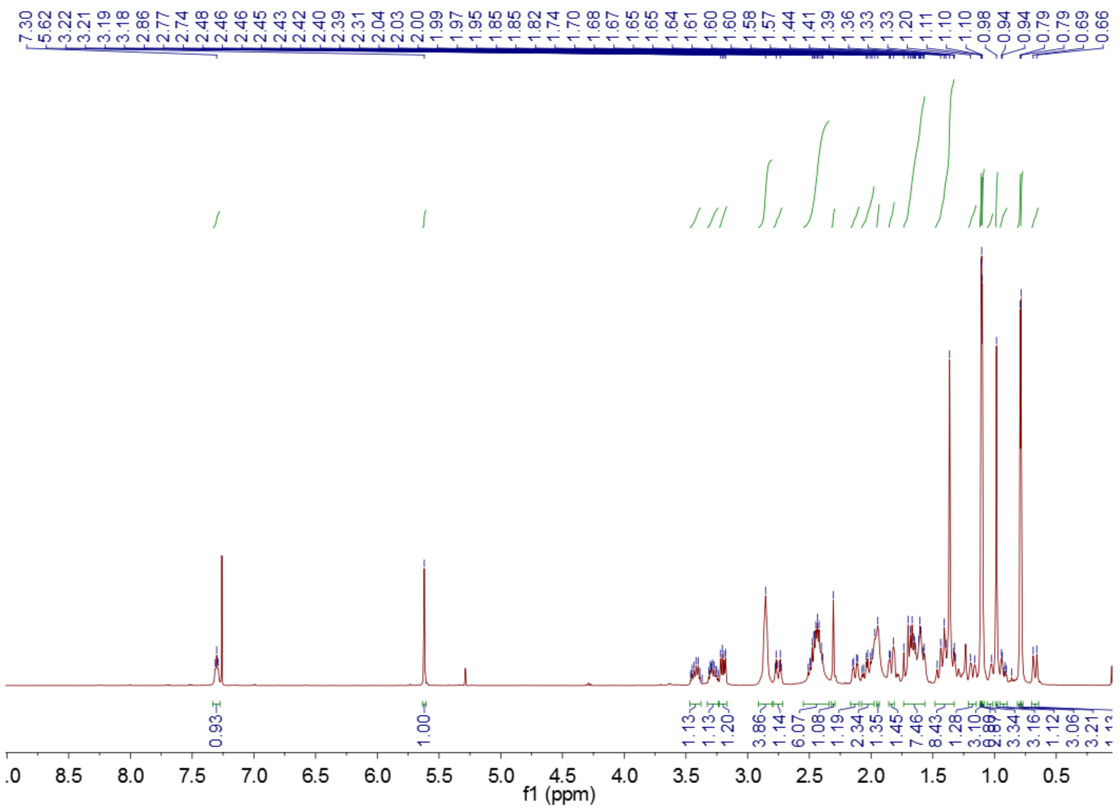

Figure S73. ^{1}H NMR spectrum ($CDCl_3$, 400 MHz) of target compound **A_{24}.**

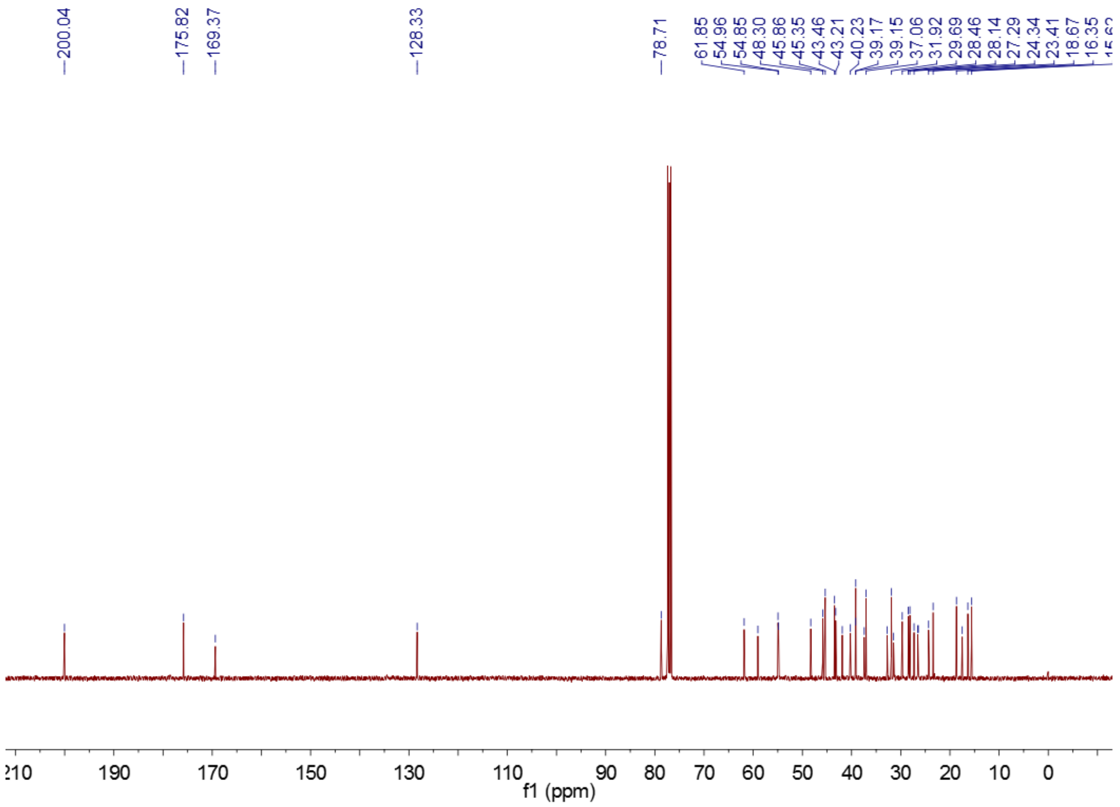

Figure S74. ^{13}C NMR spectrum ($CDCl_3$, 101 MHz) of target compound $\mathbf{A_{24}}$.

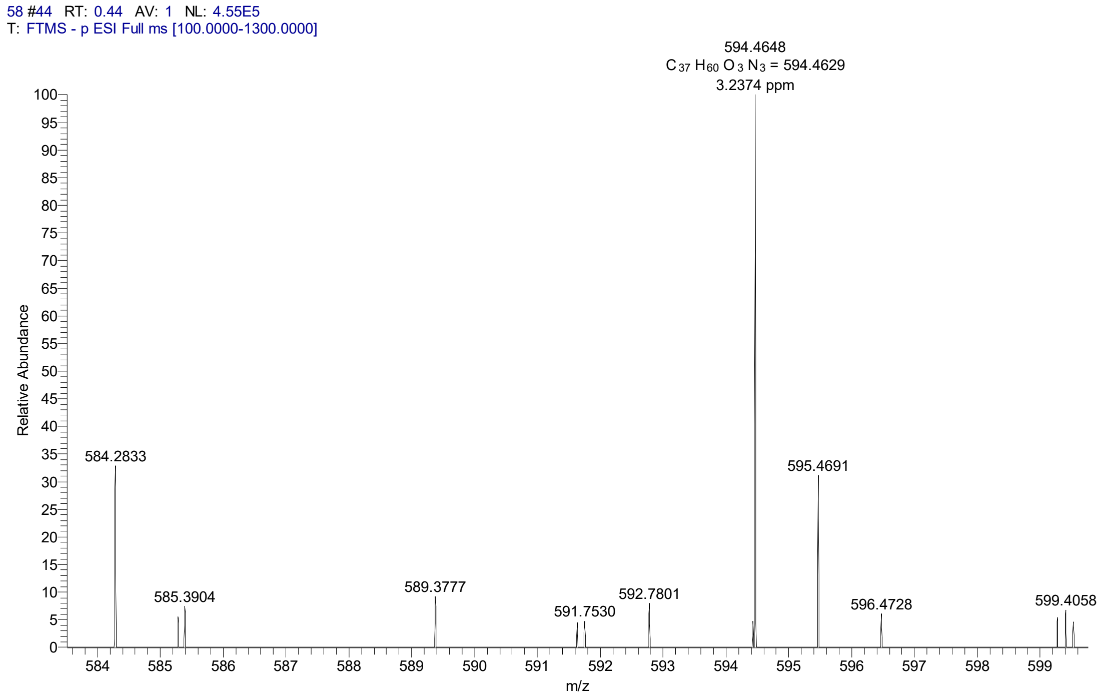

Figure S75. HRMS spectrum of target compound $\mathbf{A_{24}}$.

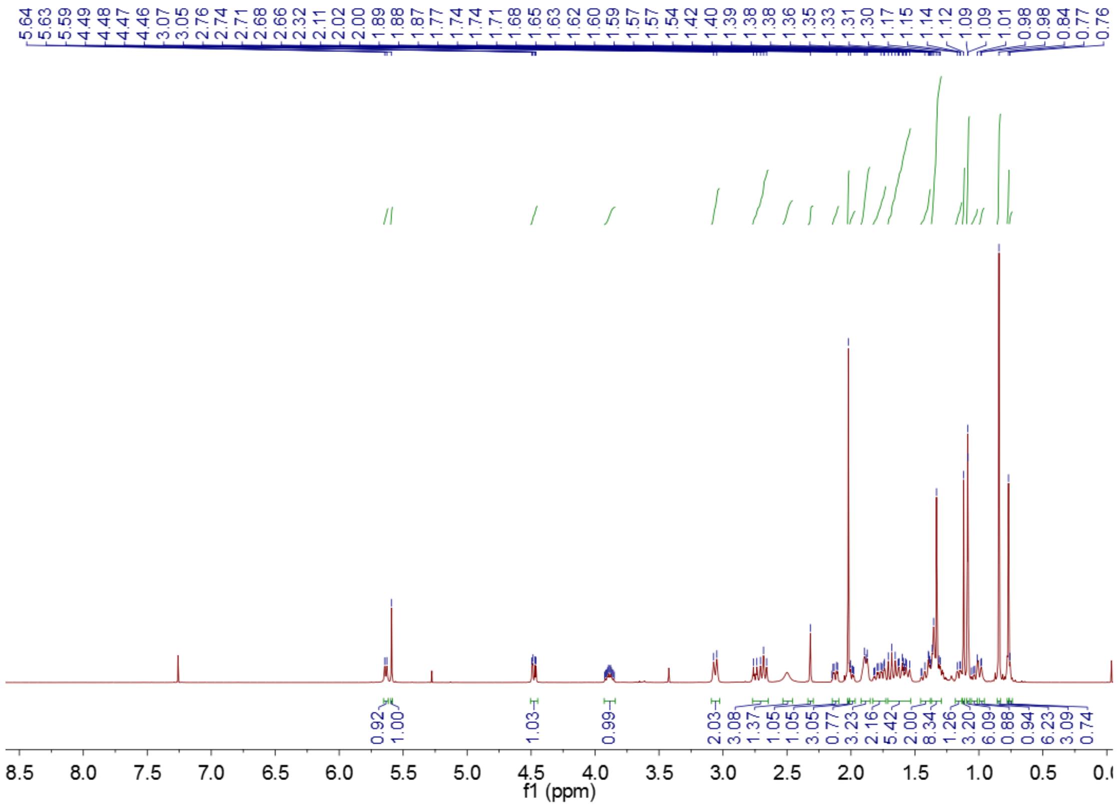

Figure S76. ^{1}H NMR spectrum ($CDCl_3$, 500 MHz) of target compound $\mathbf{A_{25}}$.

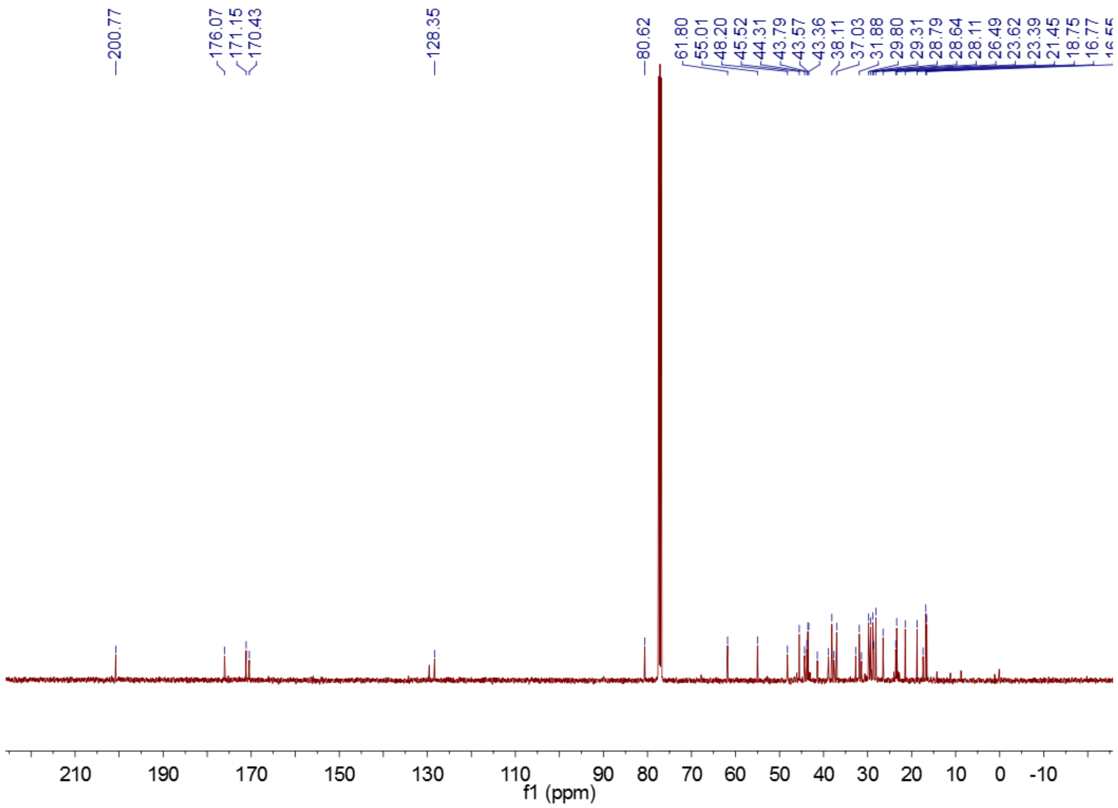

Figure S77. ^{13}C NMR spectrum ($CDCl_3$, 126 MHz) of target compound $\mathbf{A_{25}}$.

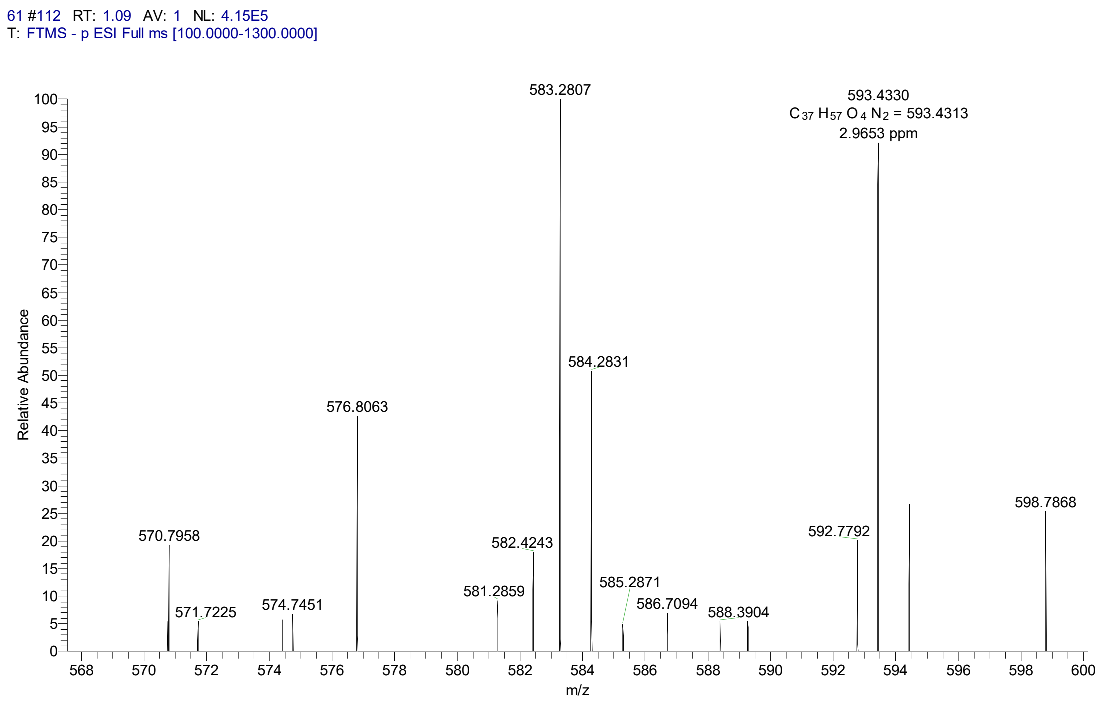

Figure S78. HRMS spectrum of target compound **A$_{25}$.**

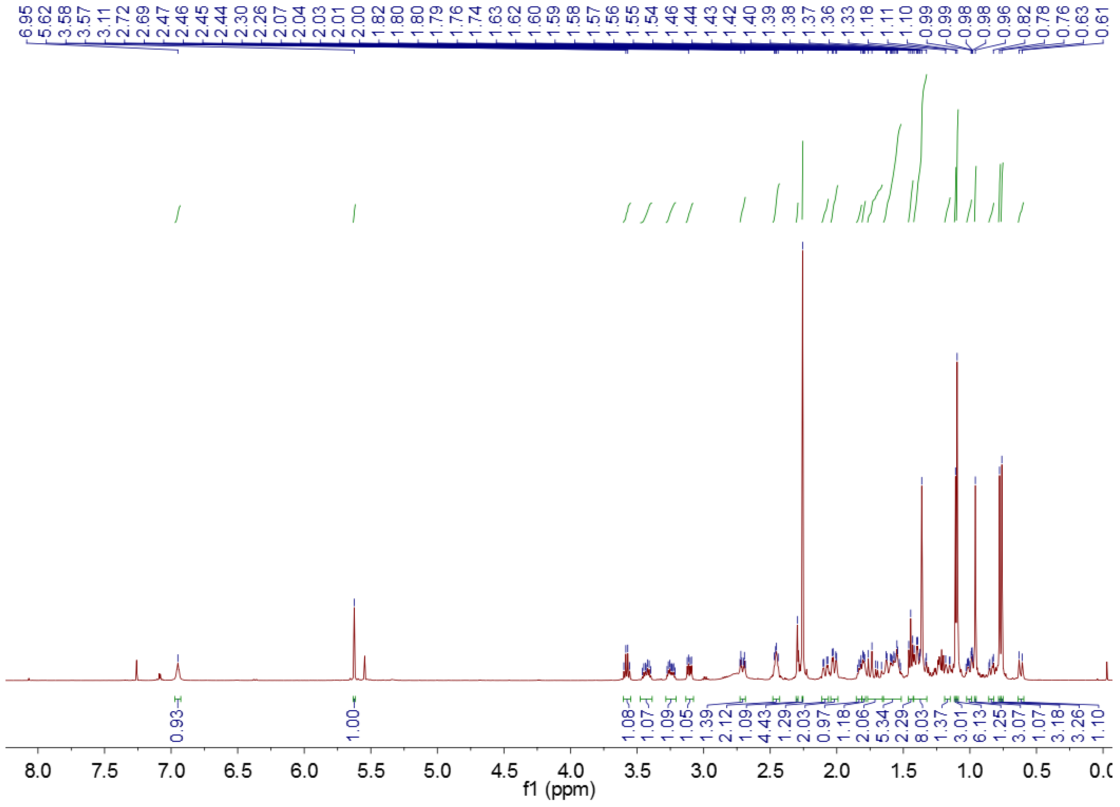

Figure S79. ^{1}H NMR spectrum ($CDCl_3$, 500 MHz) of target compound **A$_{26}$.**

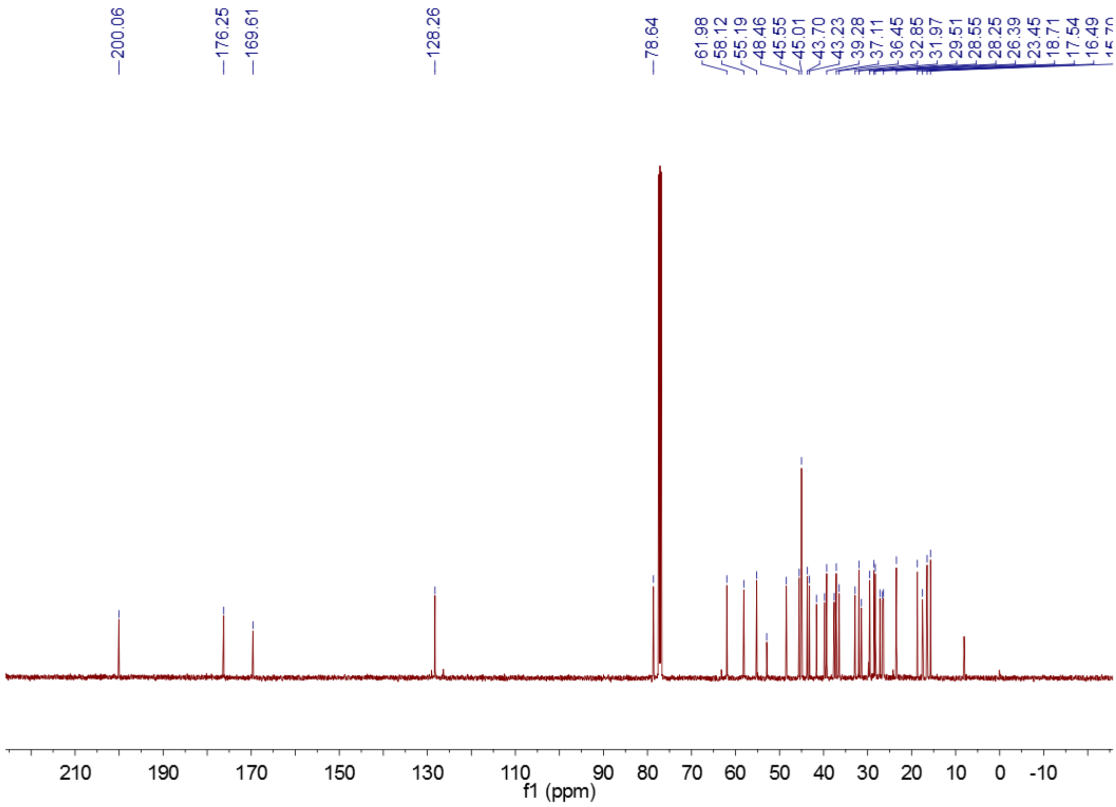

Figure S80. ^{13}C NMR spectrum ($CDCl_3$, 126 MHz) of target compound **A_{26}.**

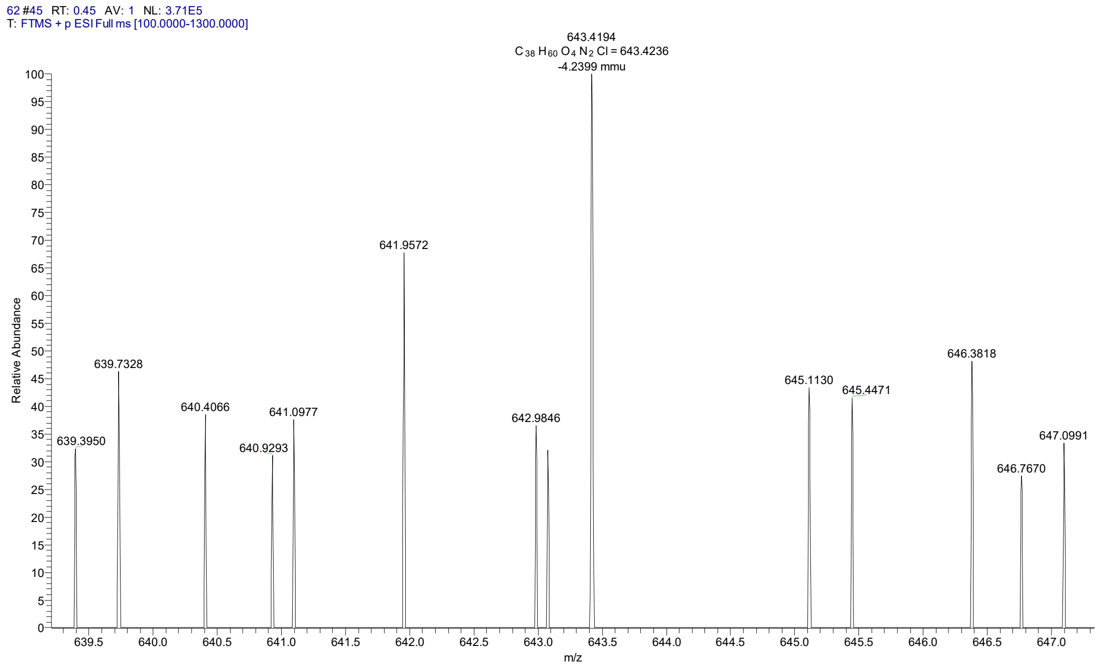

Figure S81. HRMS spectrum of target compound **A_{26}.**

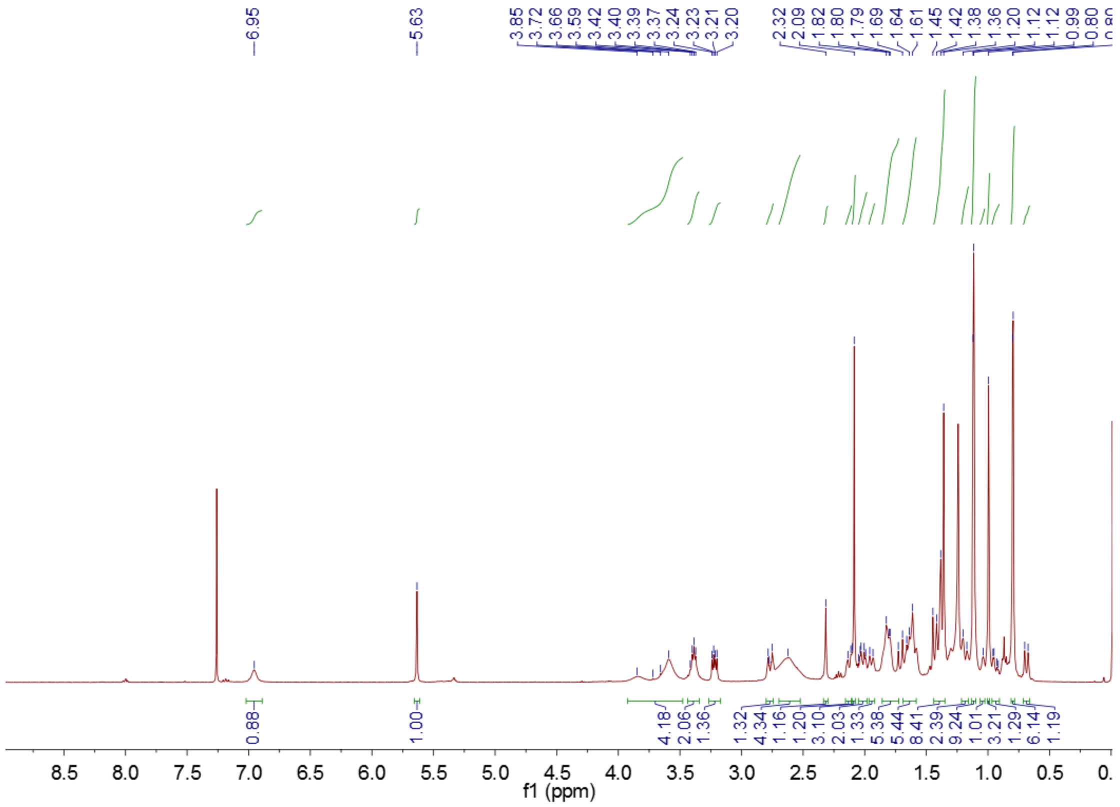

Figure S82. ^{1}H NMR spectrum ($CDCl_3$, 400 MHz) of target compound $\mathbf{A_{27}}$.

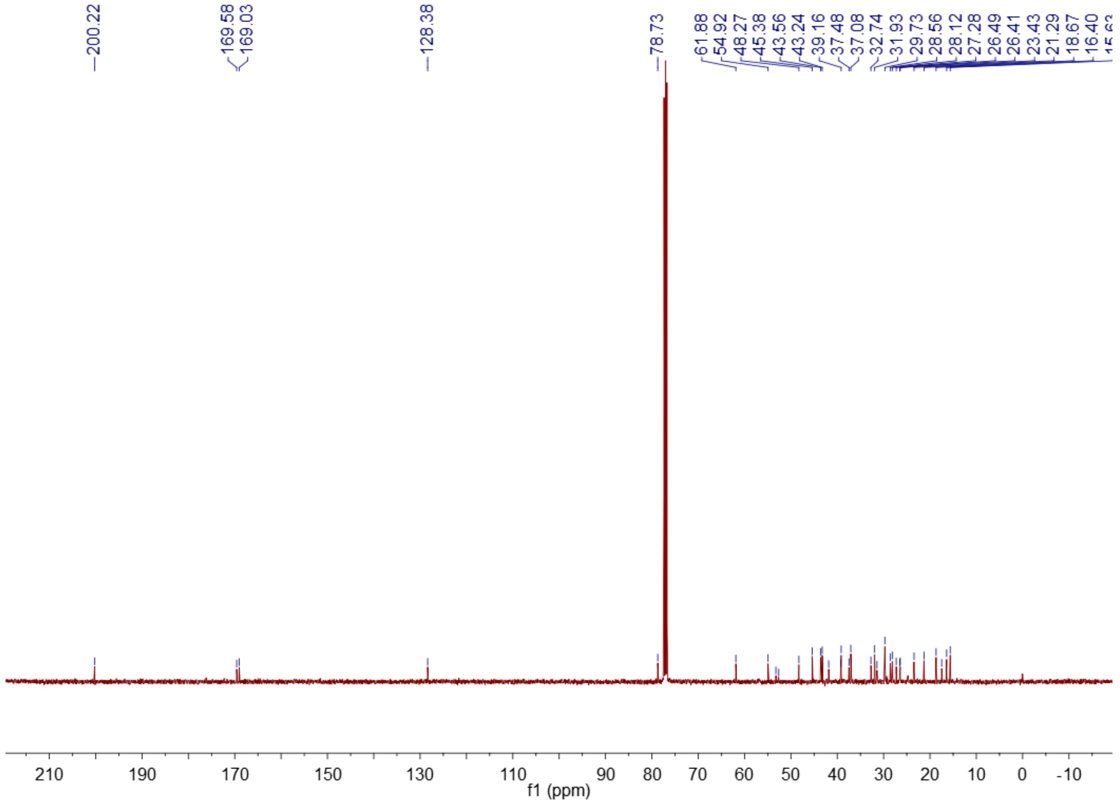

Figure S83. ^{13}C NMR spectrum ($CDCl_3$, 101 MHz) of target compound $\mathbf{A_{27}}$.

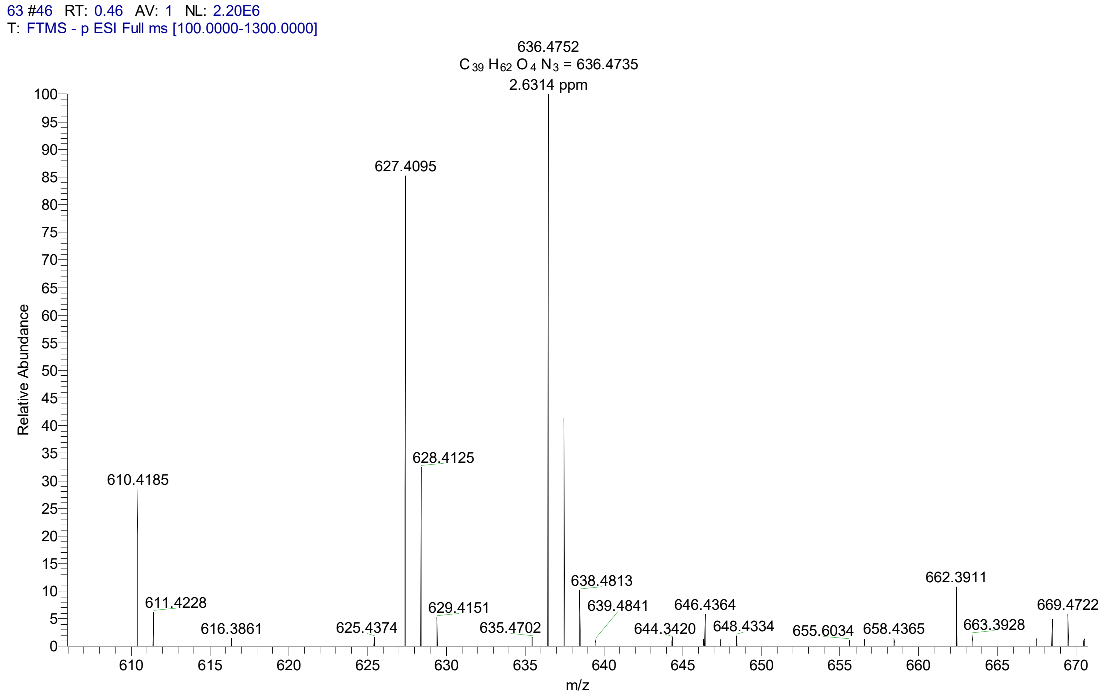

Figure S84. HRMS spectrum of target compound $\mathbf{A_{27}}$.

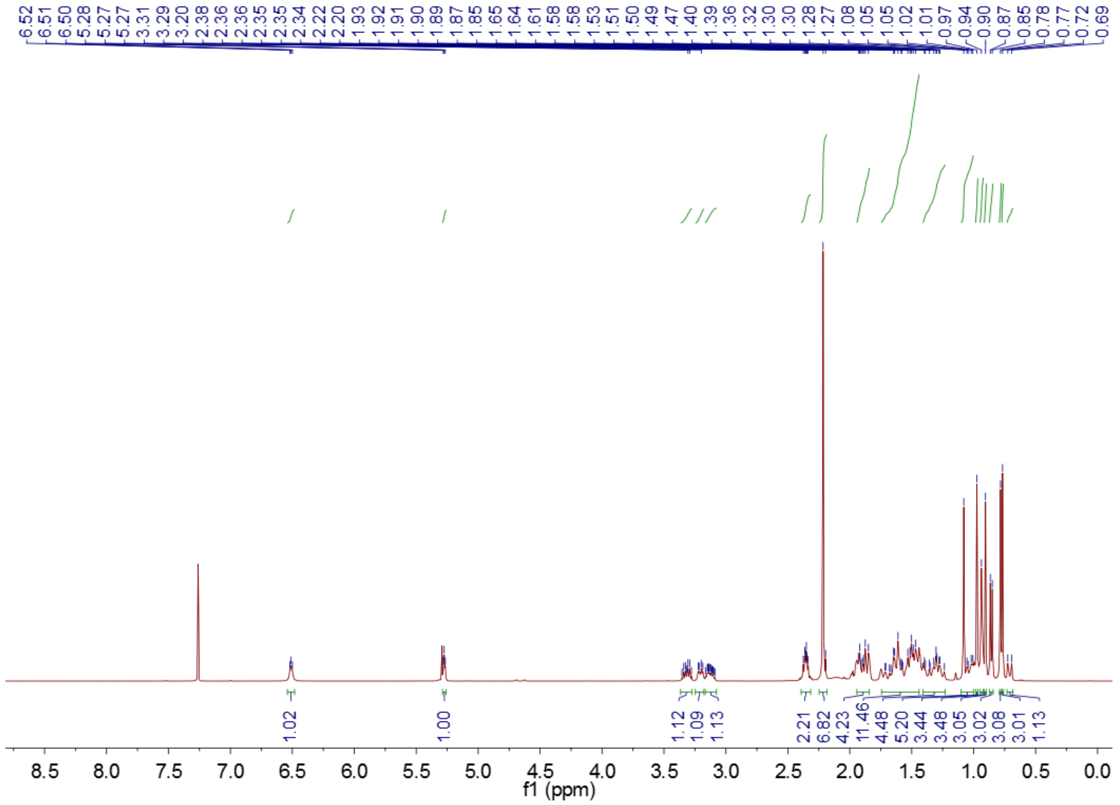

Figure S85. ^{1}H NMR spectrum ($CDCl_3$, 400 MHz) of target compound $\mathbf{B_1}$.

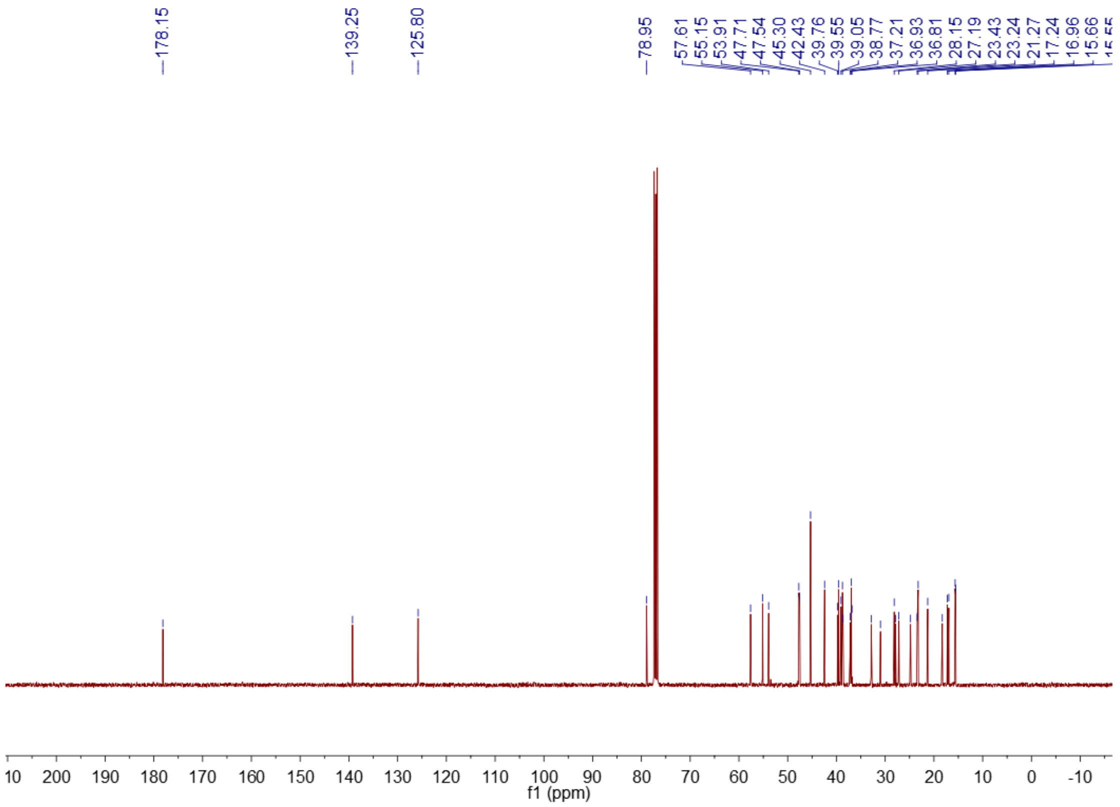

Figure S86. ^{13}C NMR spectrum ($CDCl_3$, 101 MHz) of target compound $\mathbf{B_1}$.

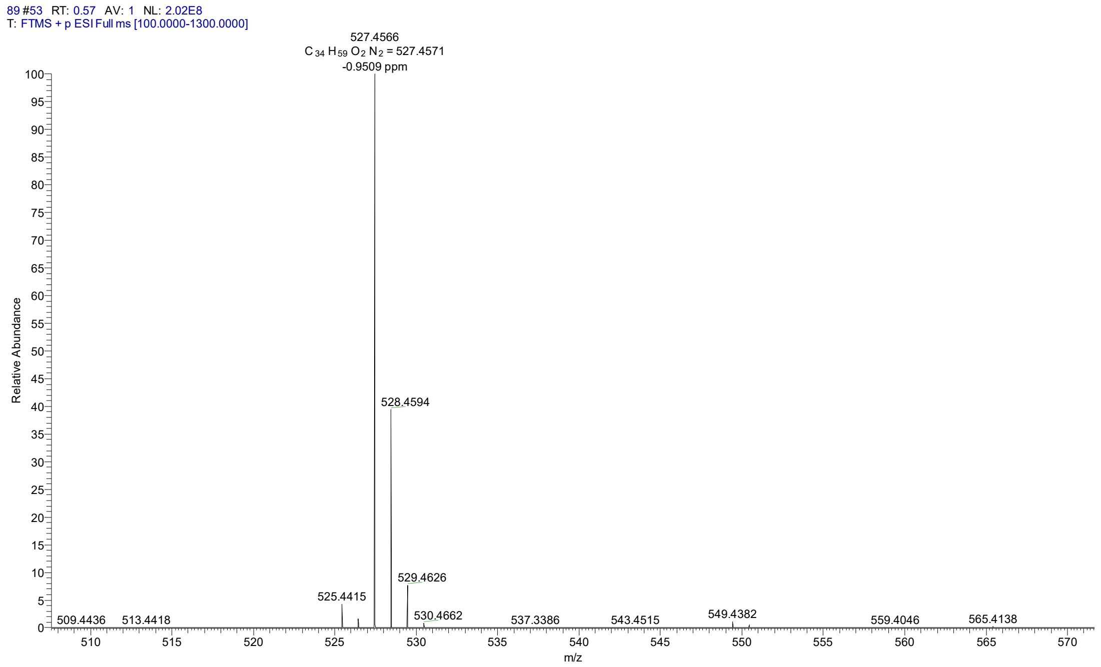

Figure S87. HRMS spectrum of target compound $\mathbf{B_1}$.

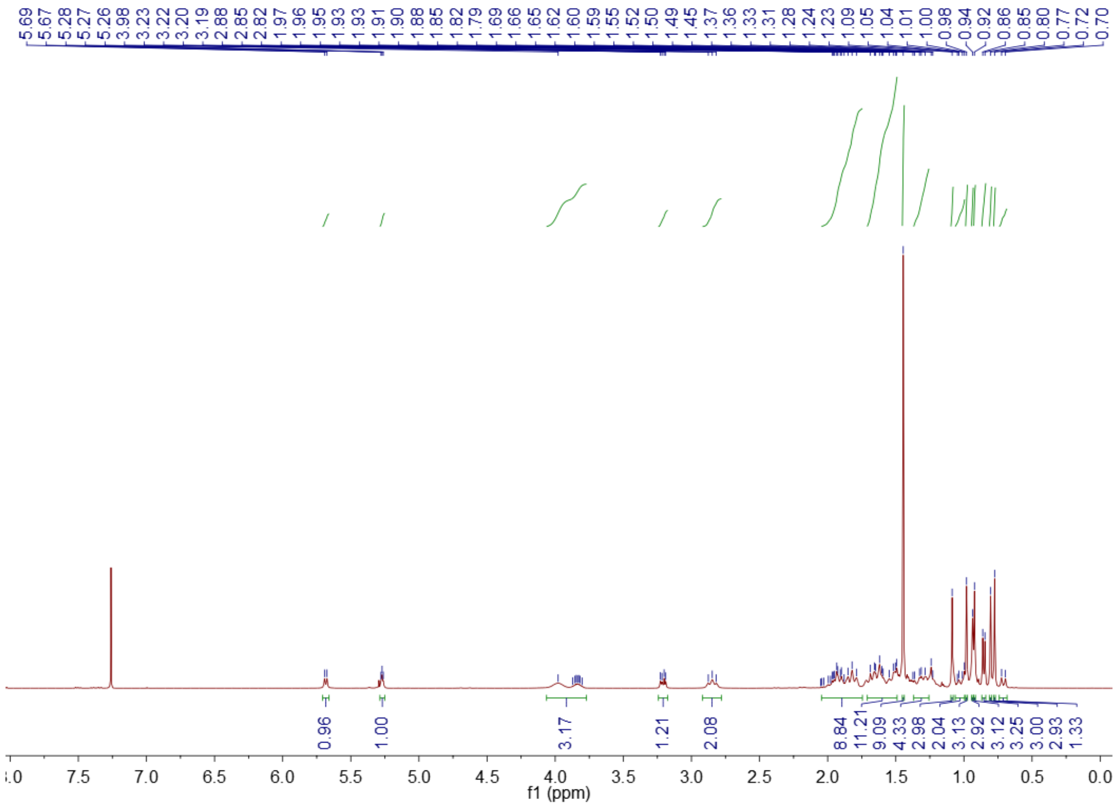

Figure S88. ^{1}H NMR spectrum ($CDCl_3$, 400 MHz) of target compound $\mathbf{B_2}$.

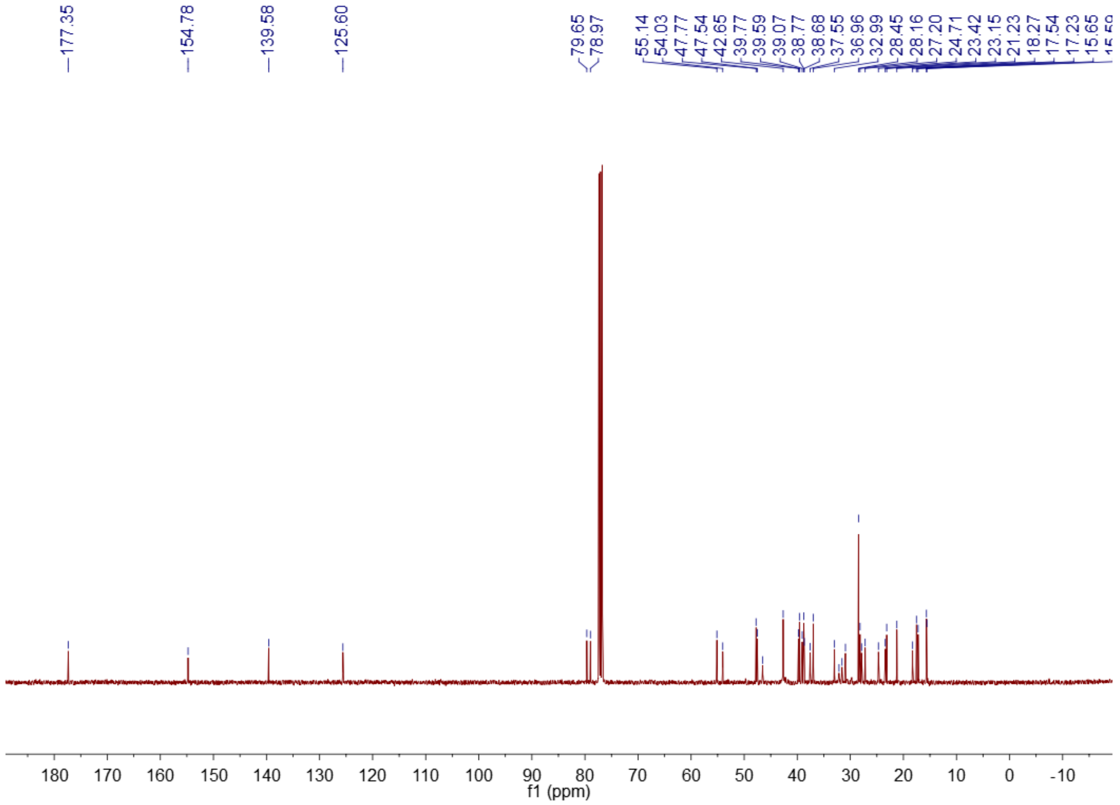

Figure S89. ^{13}C NMR spectrum ($CDCl_3$, 101 MHz) of target compound $\mathbf{B_2}$.

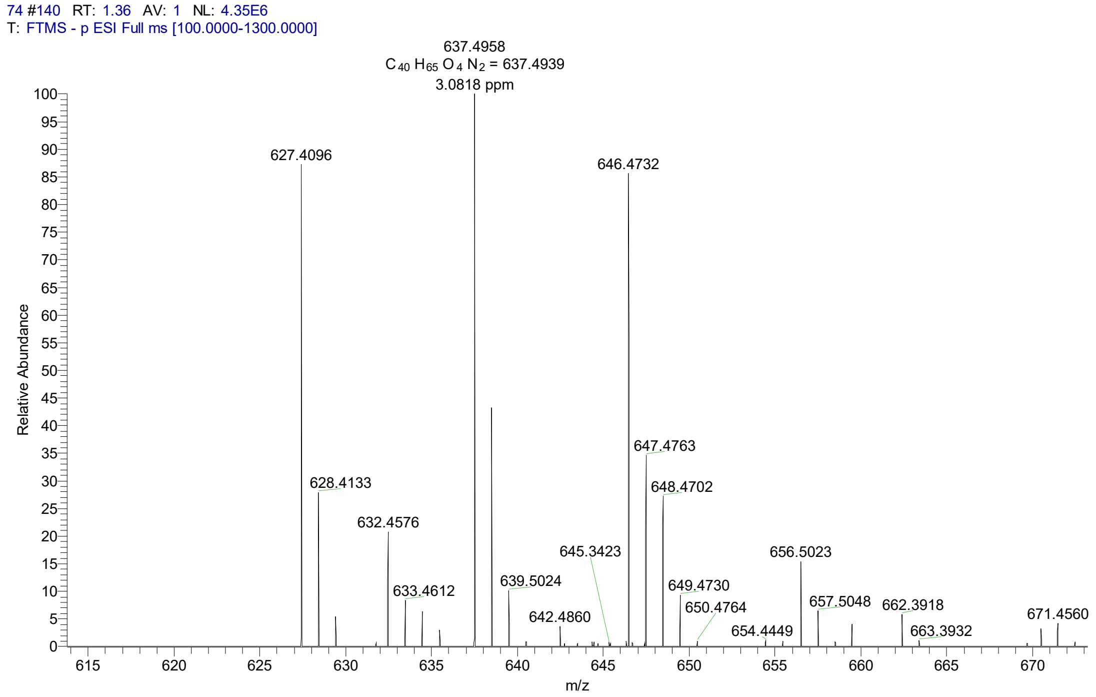

Figure S90. HRMS spectrum of target compound **B$_2$.**

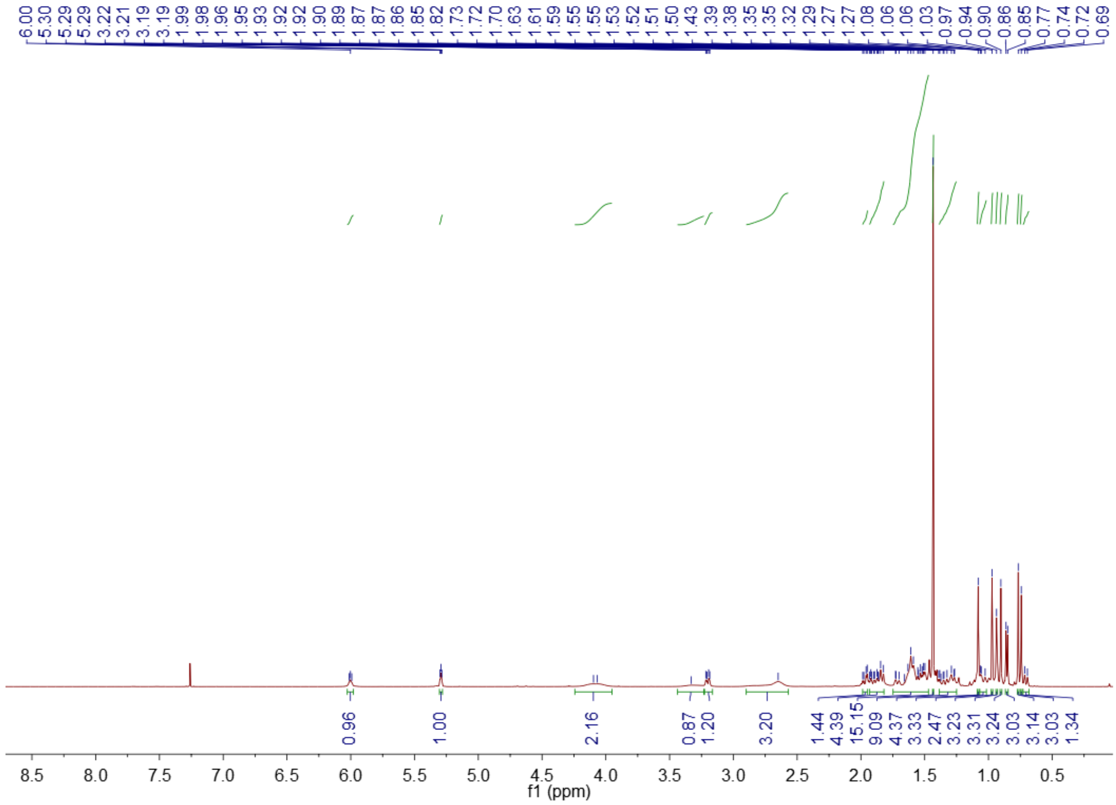

Figure S91. ^{1}H NMR spectrum (CDCl$_3$, 500 MHz) of target compound **B$_3$.**

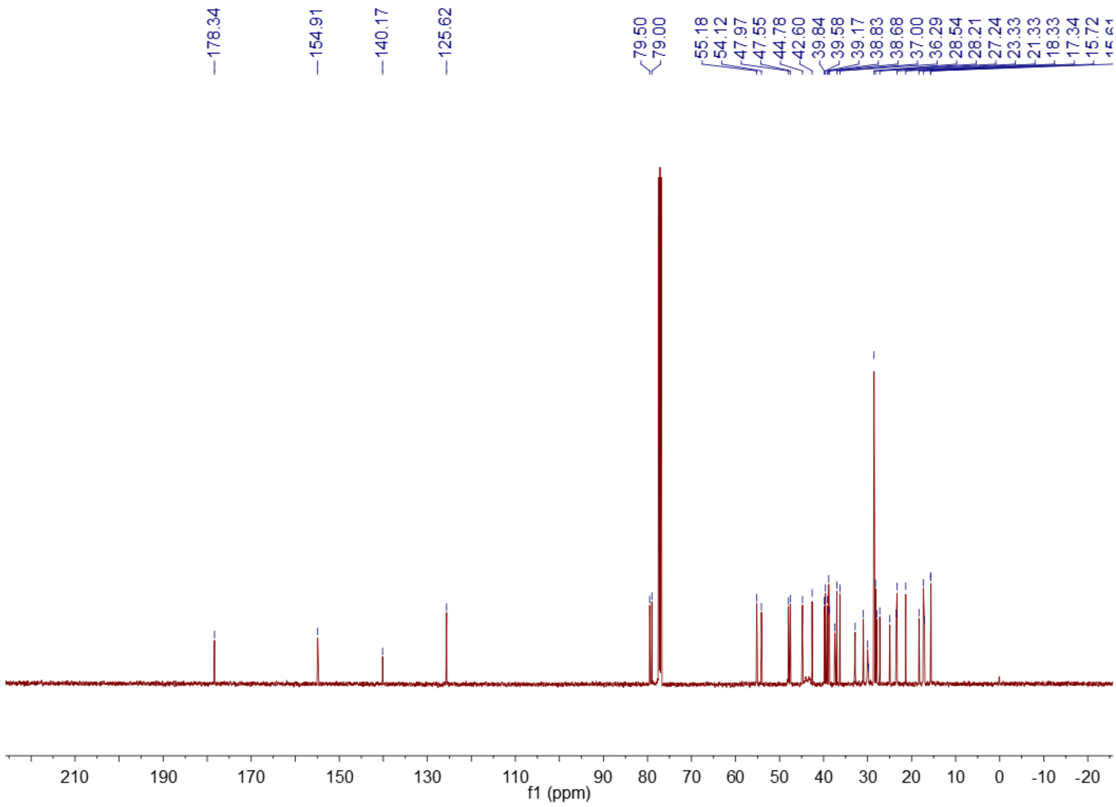

Figure S92. ^{13}C NMR spectrum ($CDCl_3$, 126 MHz) of target compound **B_3.**

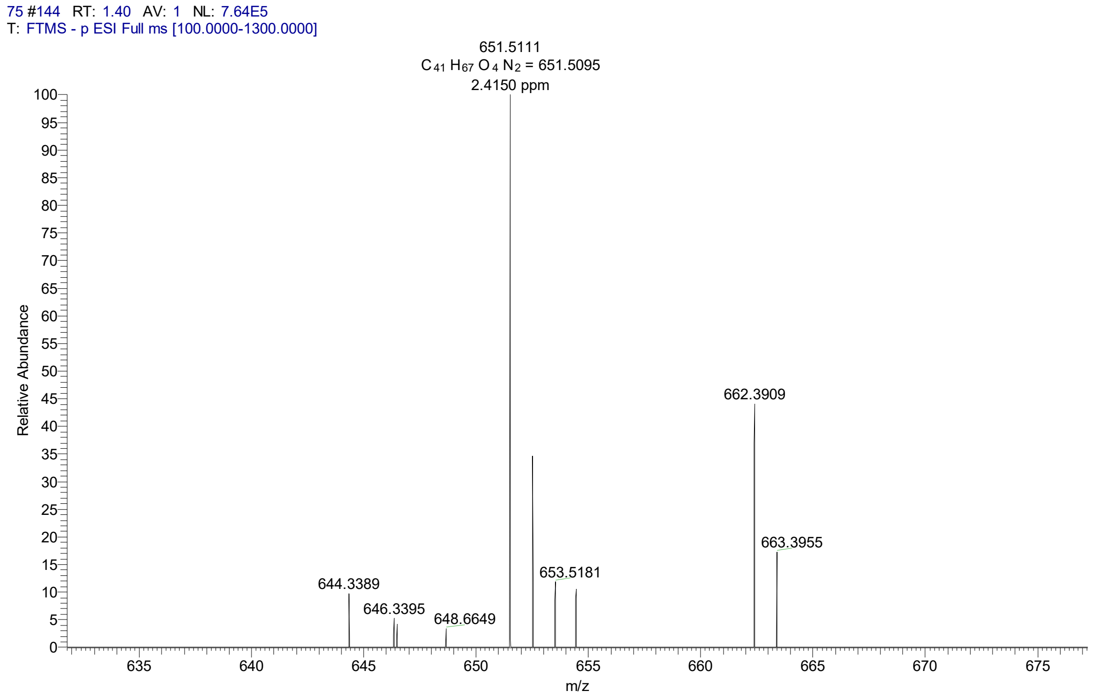

Figure S93. HRMS spectrum of target compound **B_3.**

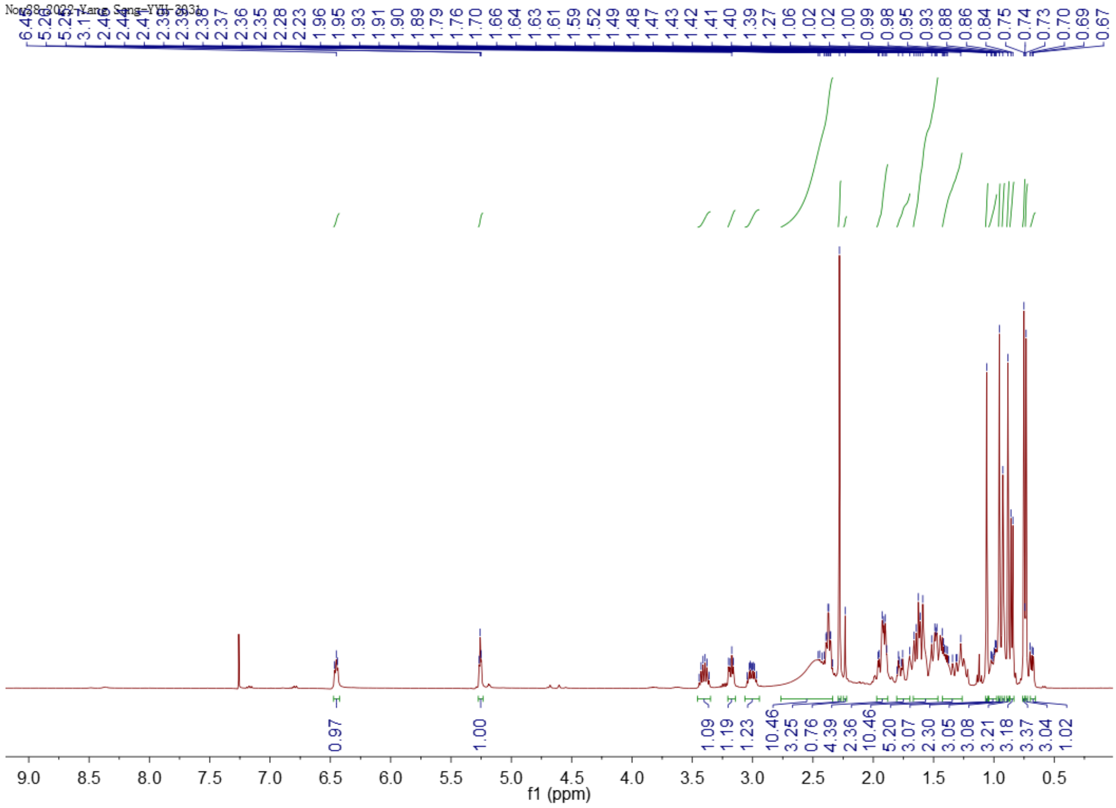

Figure S94. ^{1}H NMR spectrum ($CDCl_3$, 400 MHz) of target compound **B_4.**

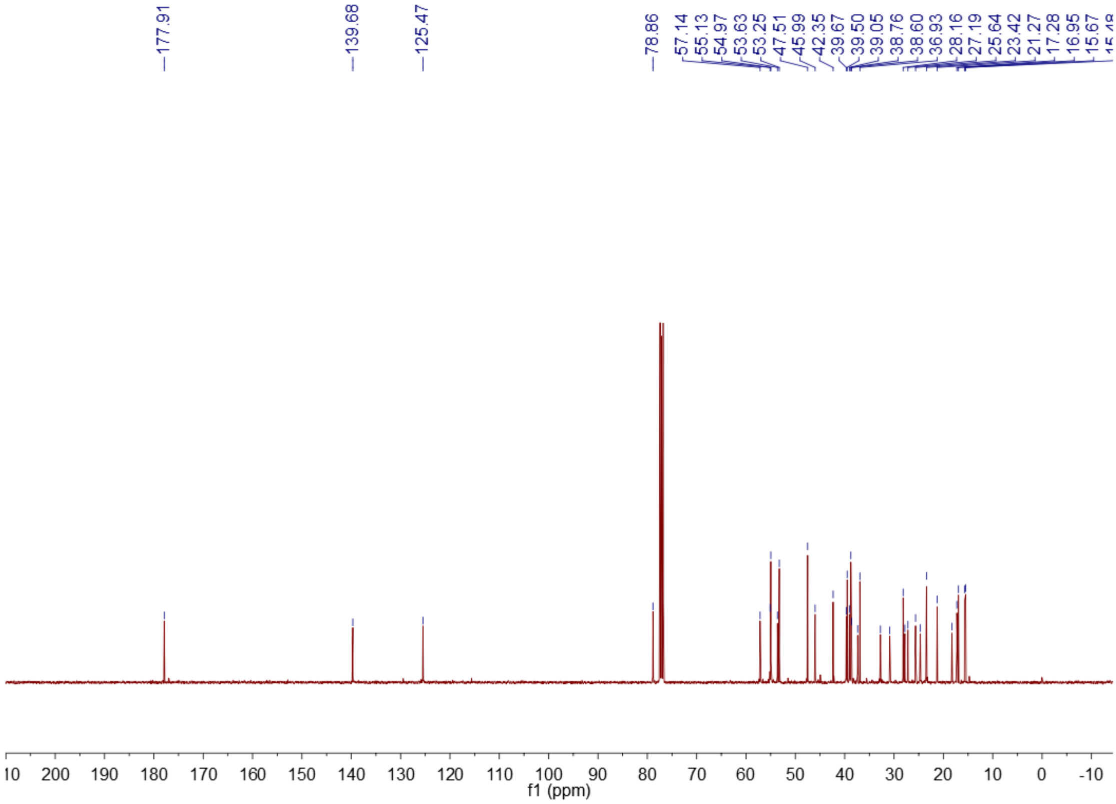

Figure S95. ^{13}C NMR spectrum ($CDCl_3$, 101 MHz) of target compound **B_4.**

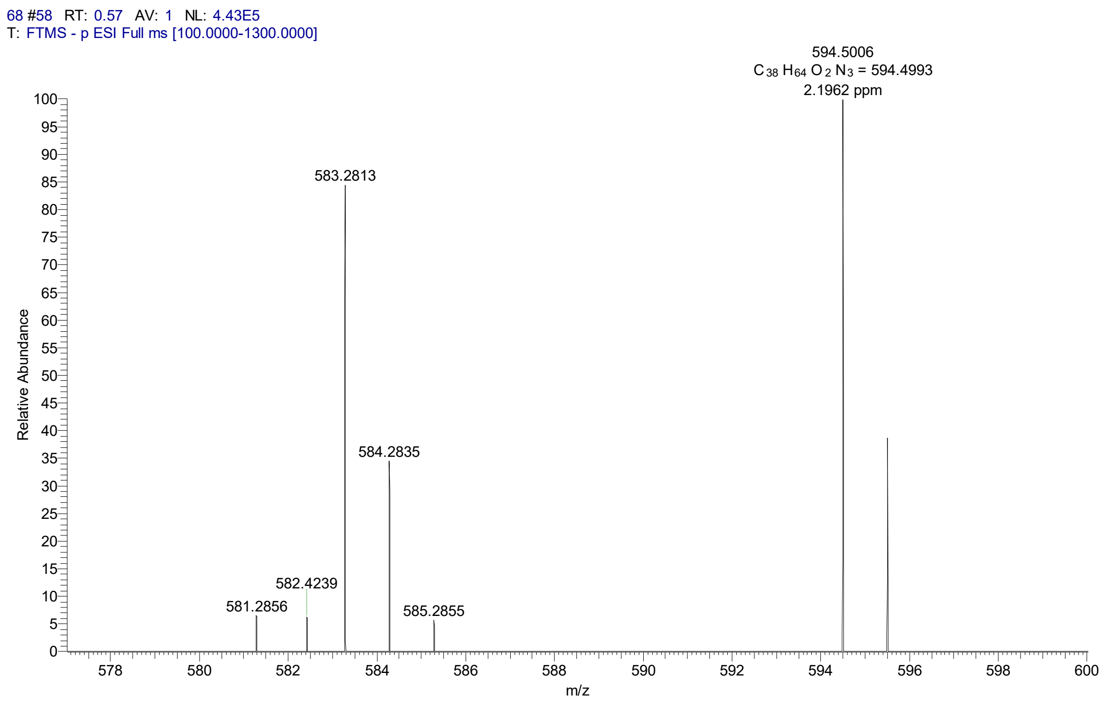

Figure S96. HRMS spectrum of target compound **B$_4$.**

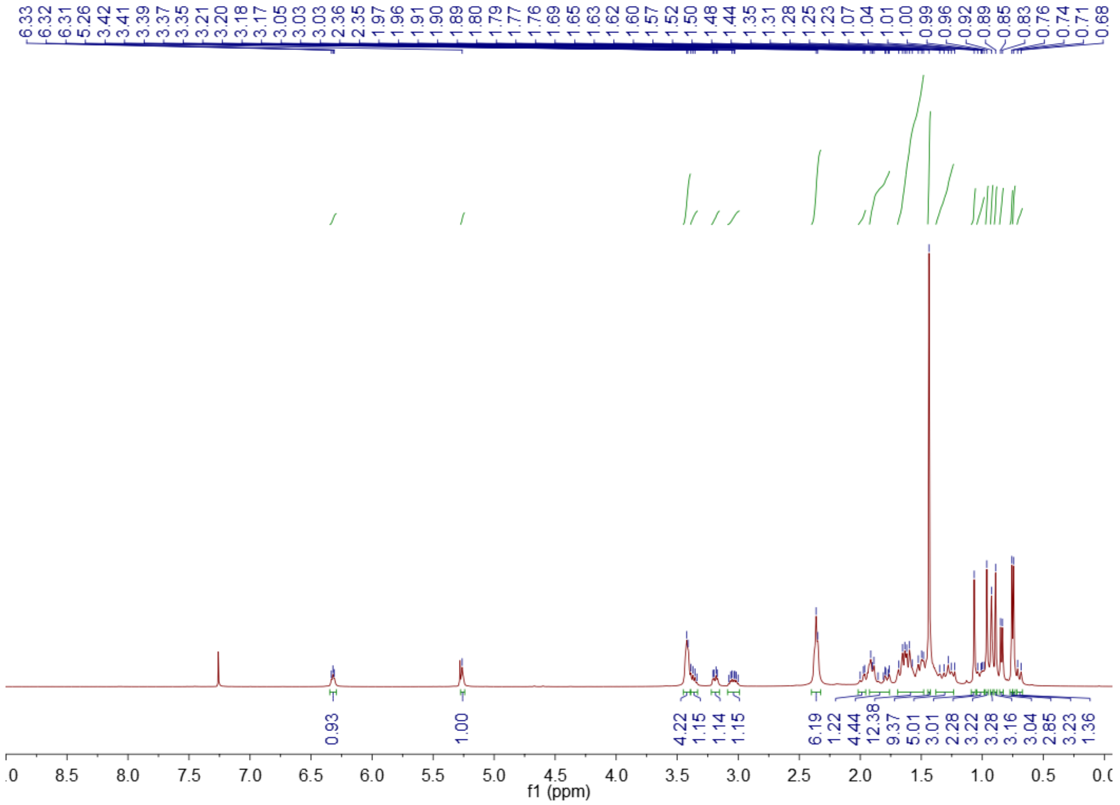

Figure S97. ^{1}H NMR spectrum ($CDCl_3$, 400 MHz) of target compound **B$_5$.**

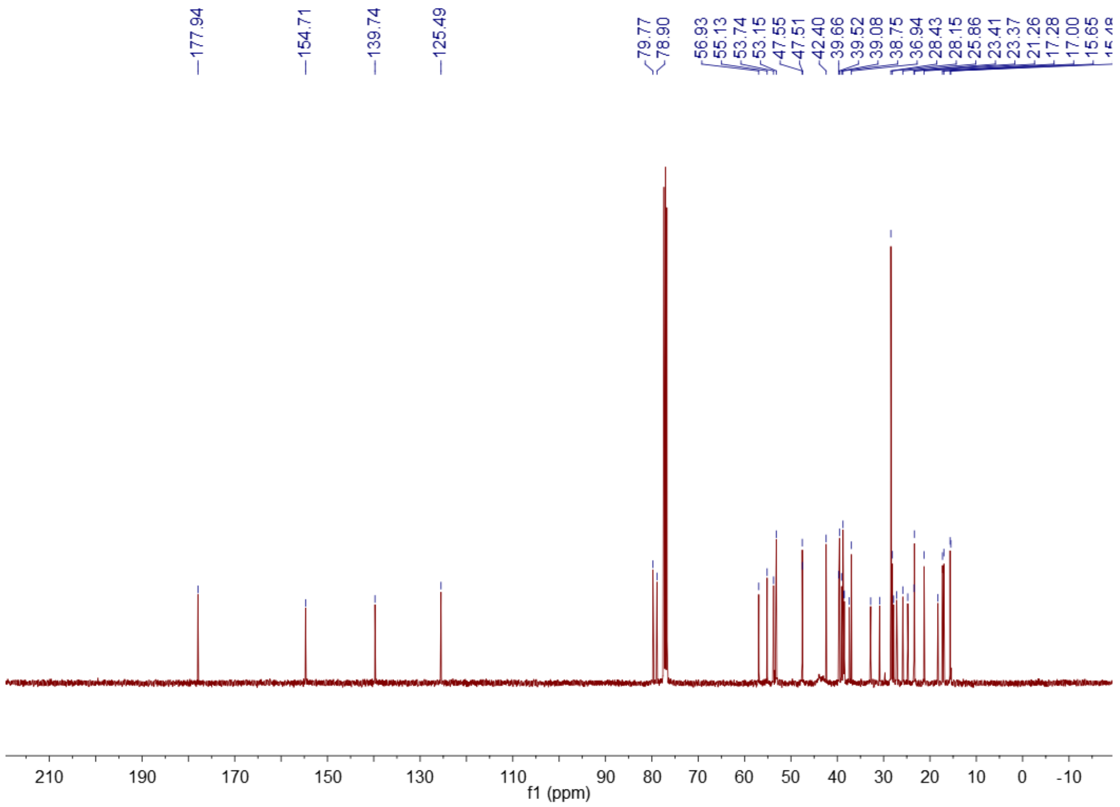

Figure S98. ^{13}C NMR spectrum ($CDCl_3$, 101 MHz) of target compound **B_5.**

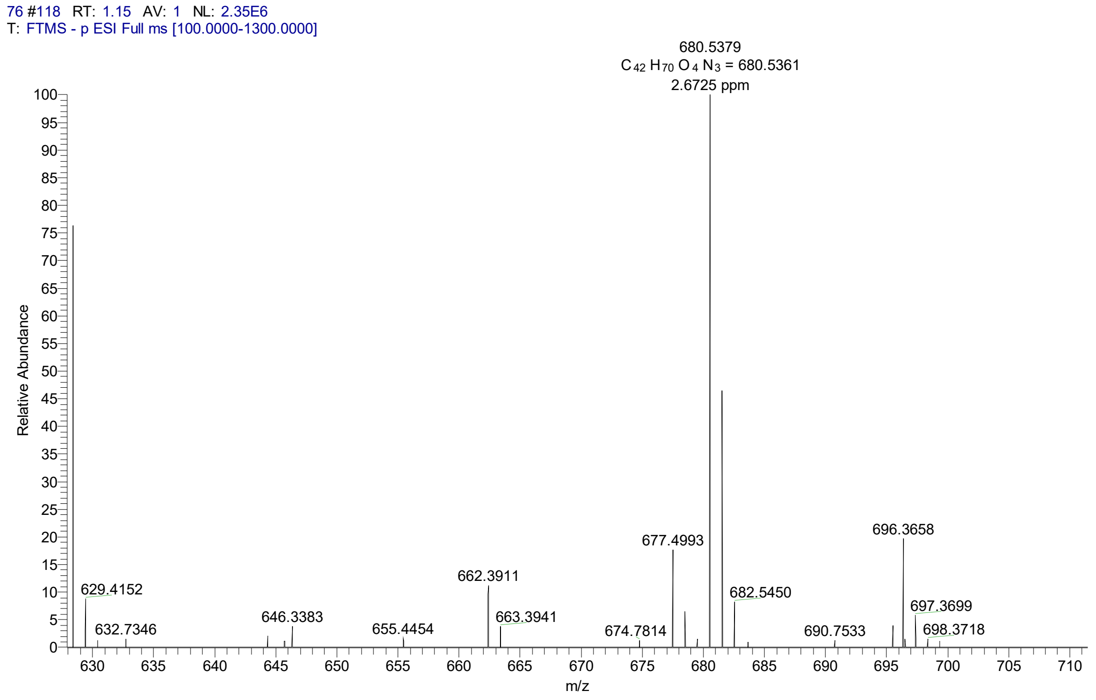

Figure S99. HRMS spectrum of target compound **B_5.**

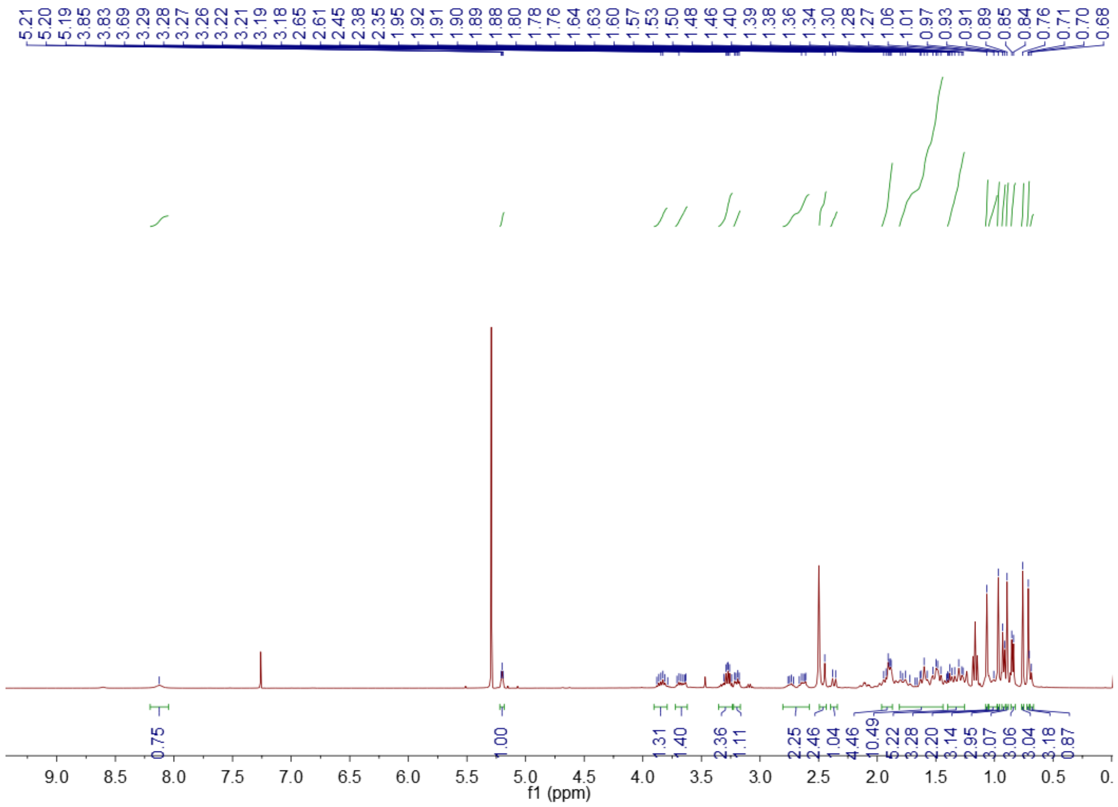

Figure S100. ^{1}H NMR spectrum ($CDCl_3$, 500 MHz) of target compound $\mathbf{B_6}$.

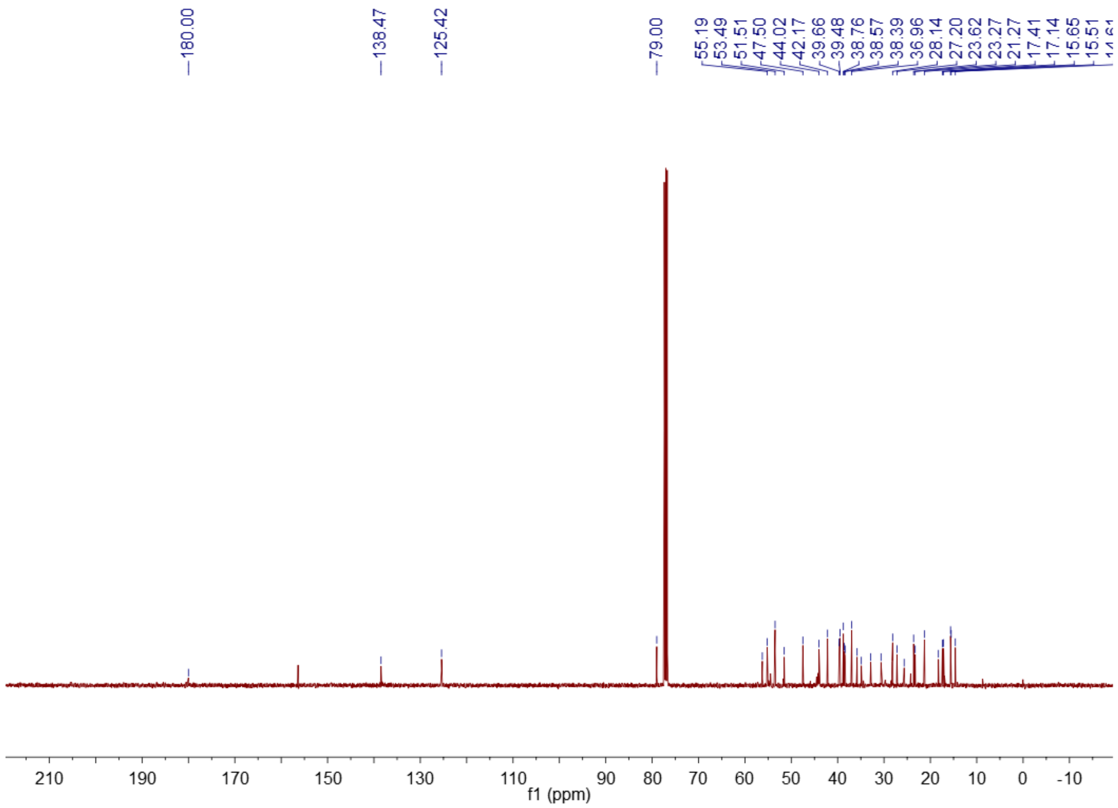

Figure S101. ^{13}C NMR spectrum ($CDCl_3$, 126 MHz) of target compound $\mathbf{B_6}$.

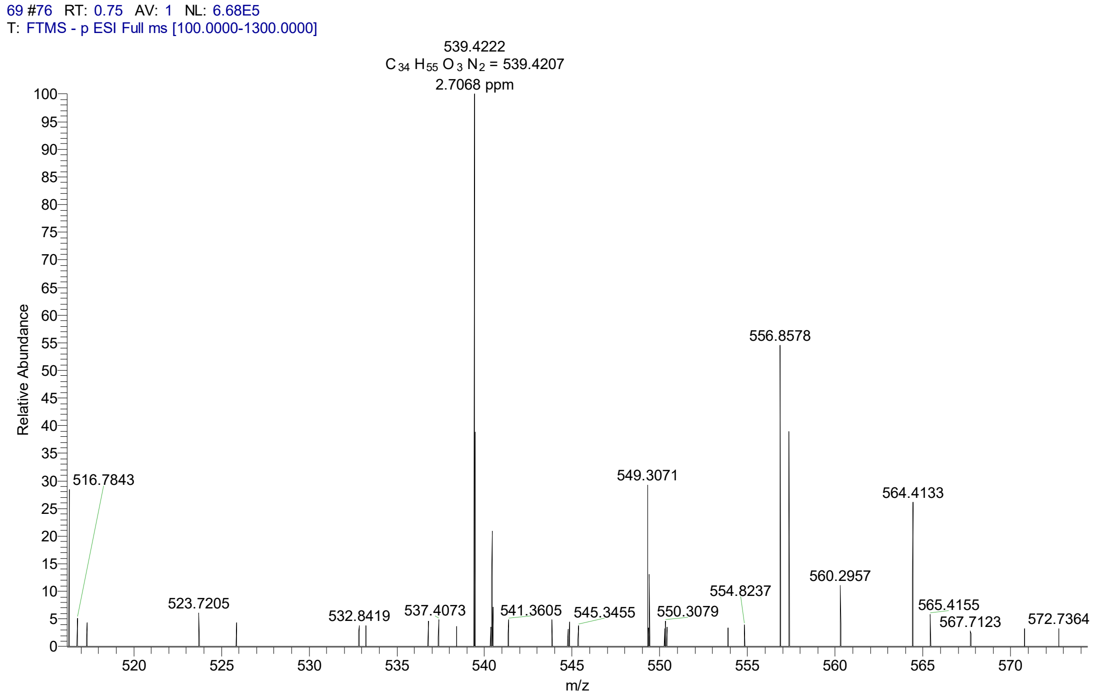

Figure S102. HRMS spectrum of target compound **B₆.**

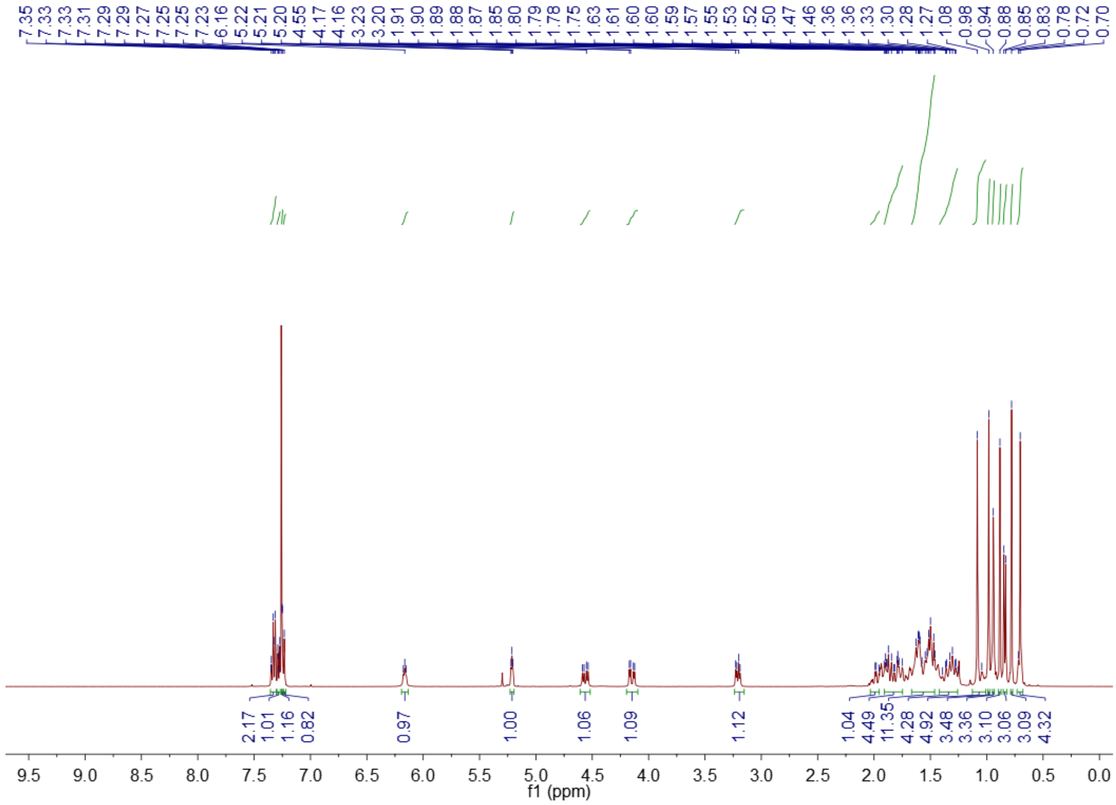

Figure S103. ^{1}H NMR spectrum ($CDCl_3$, 400 MHz) of target compound **B₇.**

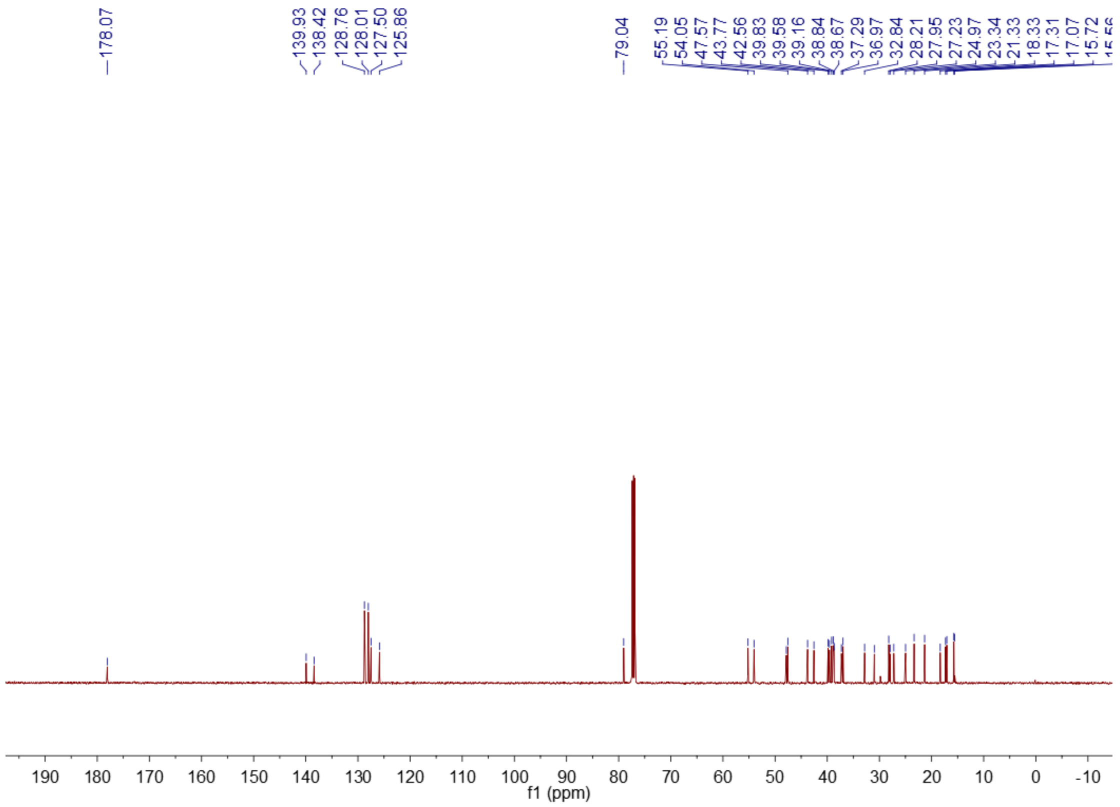

Figure S104. ^{13}C NMR spectrum ($CDCl_3$, 126 MHz) of target compound **B_7.**

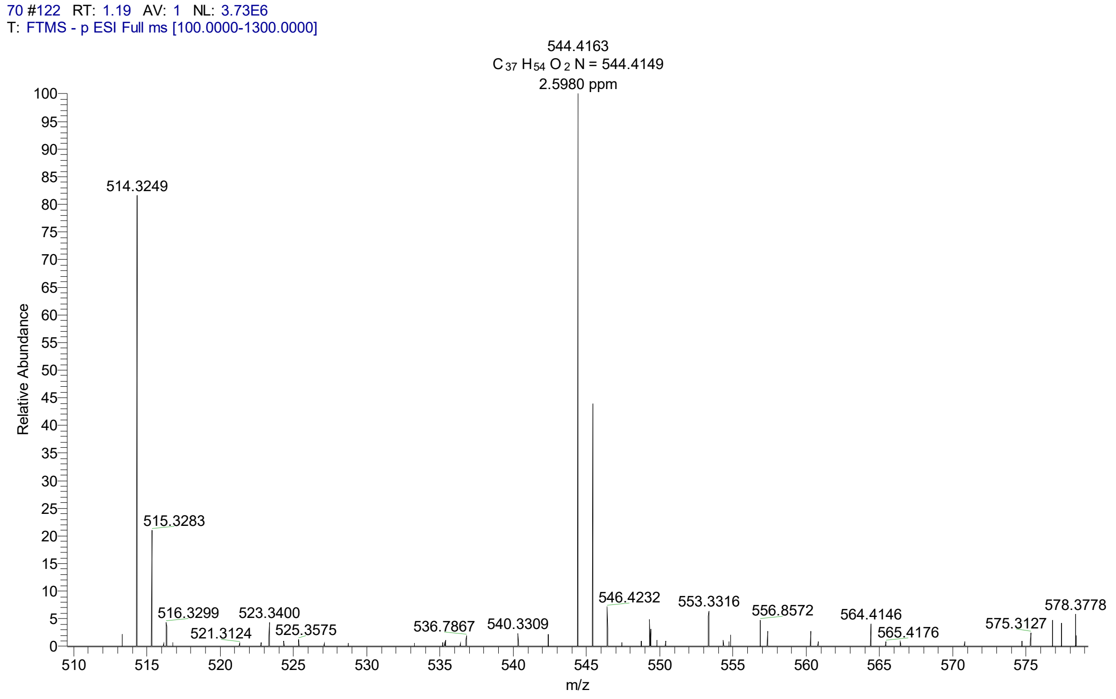

Figure S105. HRMS spectrum of target compound **B_7.**

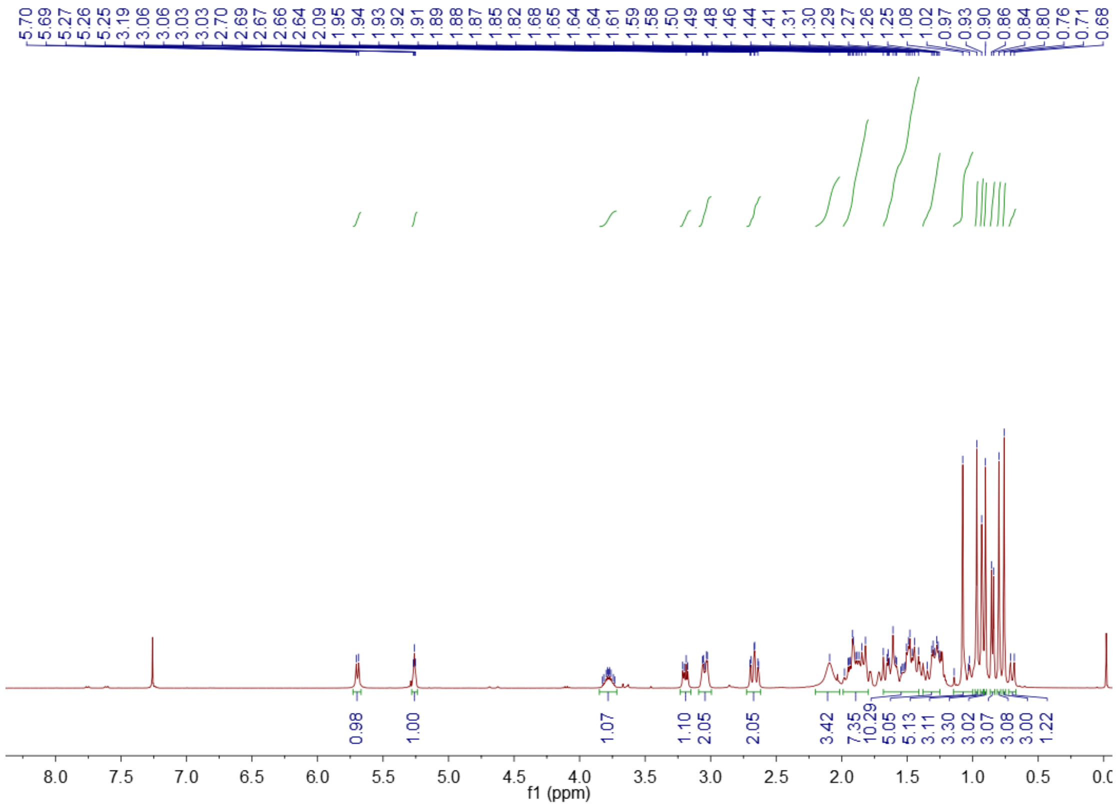

Figure S106. ^{1}H NMR spectrum ($CDCl_3$, 400 MHz) of target compound **B_8.**

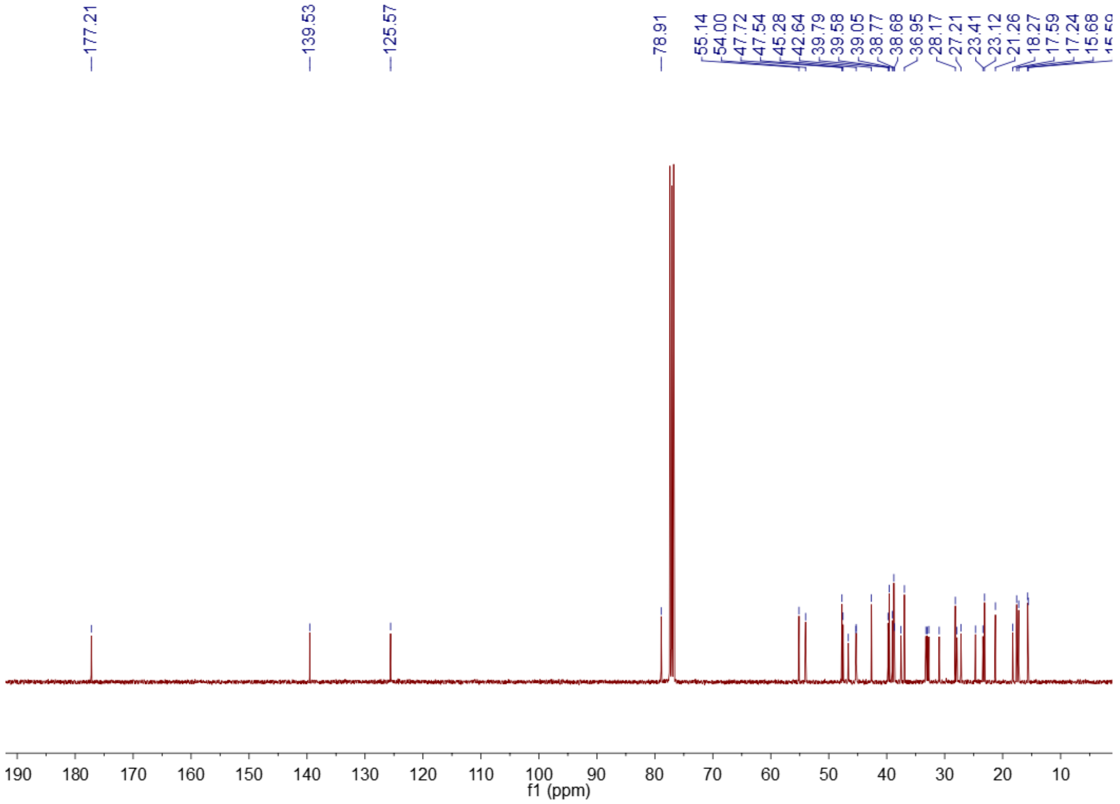

Figure S107. ^{13}C NMR spectrum ($CDCl_3$, 101 MHz) of target compound **B_8.**

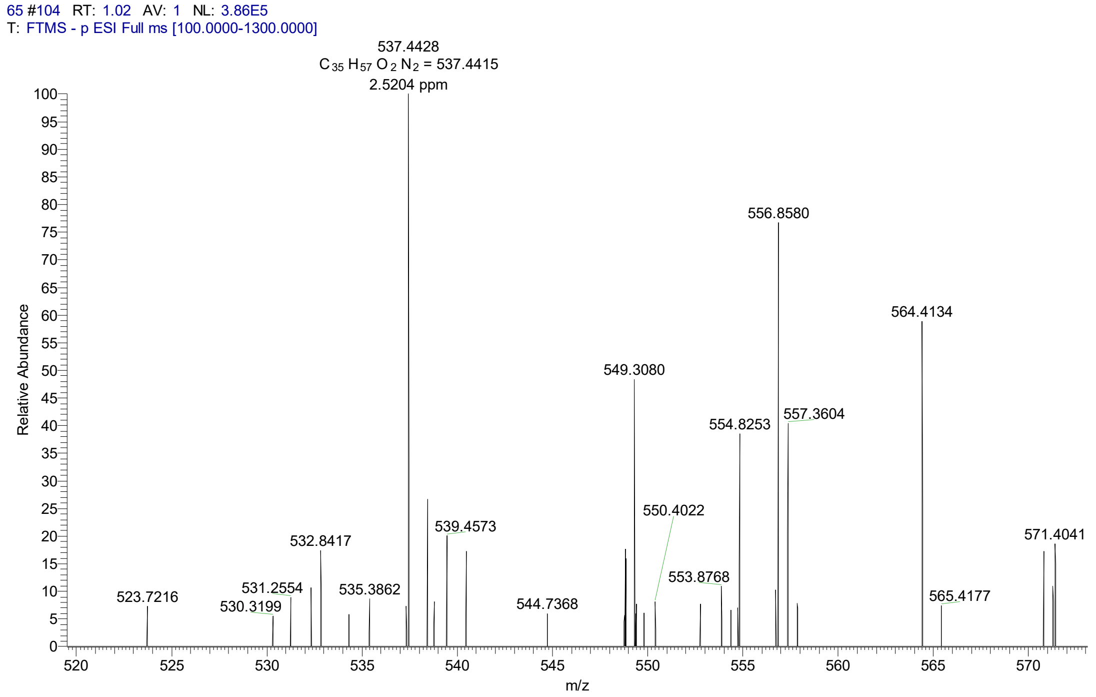

Figure S108. HRMS spectrum of target compound **B8.**

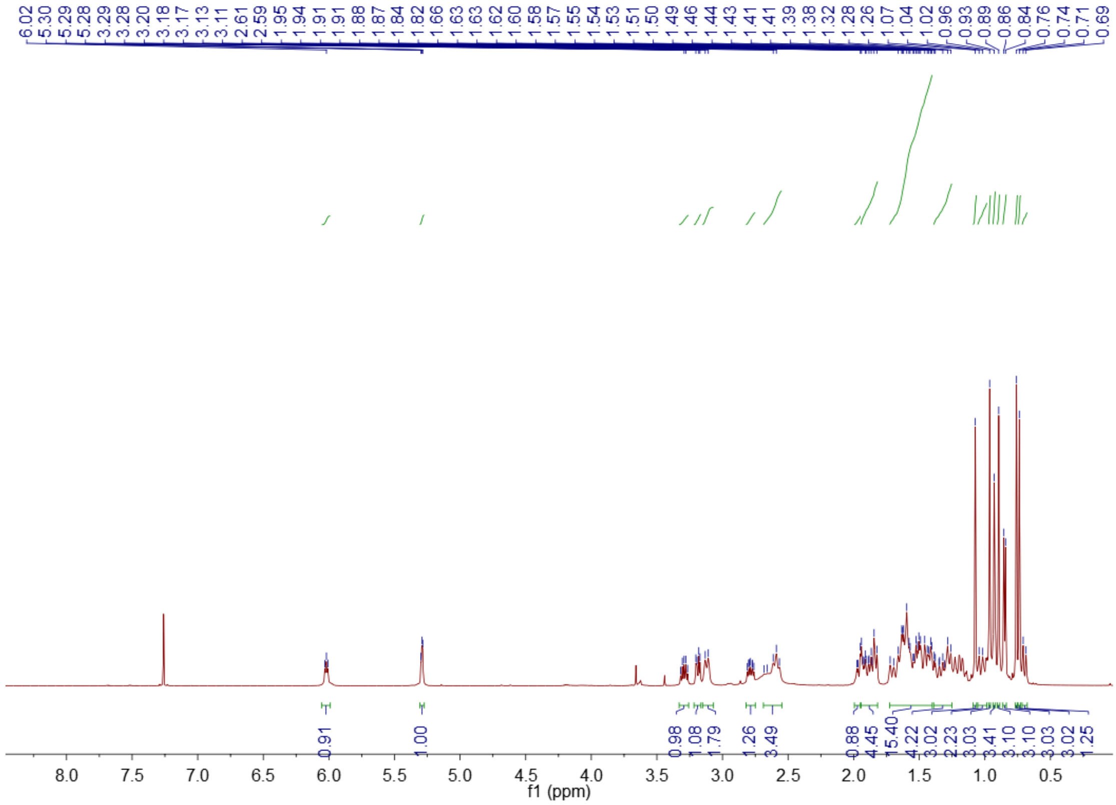

Figure S109. ^{1}H NMR spectrum ($CDCl_3$, 500 MHz) of target compound **B9.**

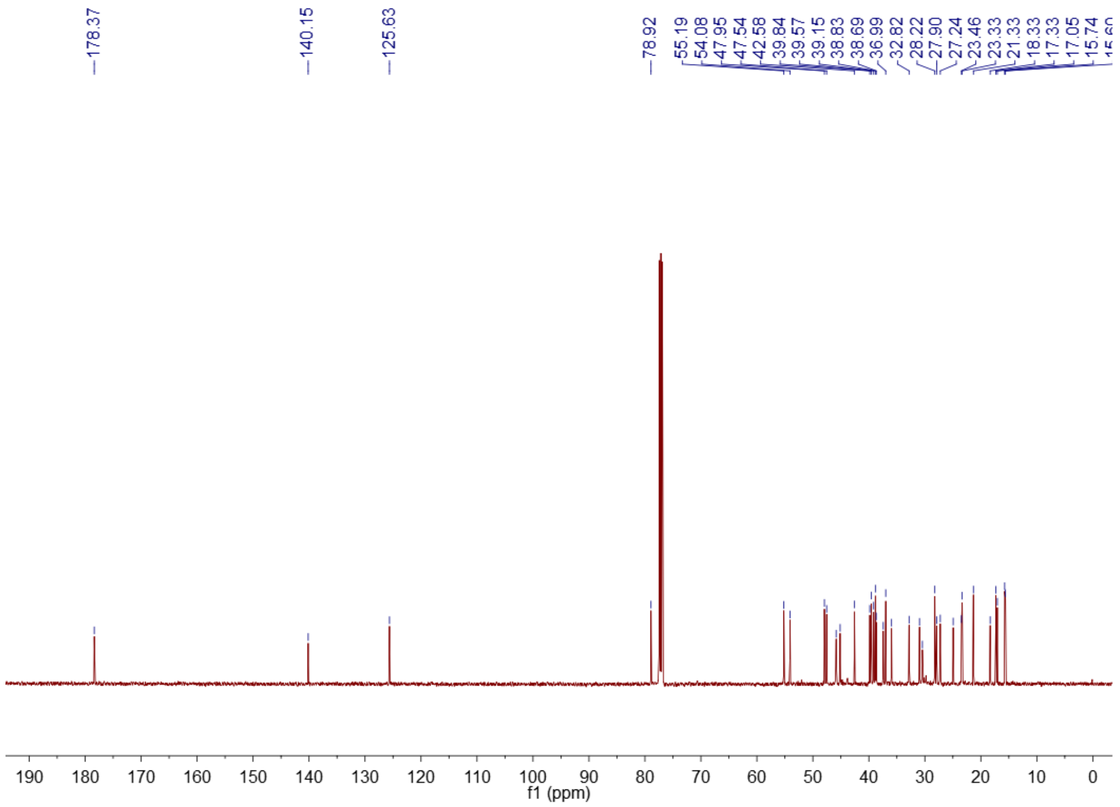

Figure S110. ^{13}C NMR spectrum ($CDCl_3$, 126 MHz) of target compound **B₉.**

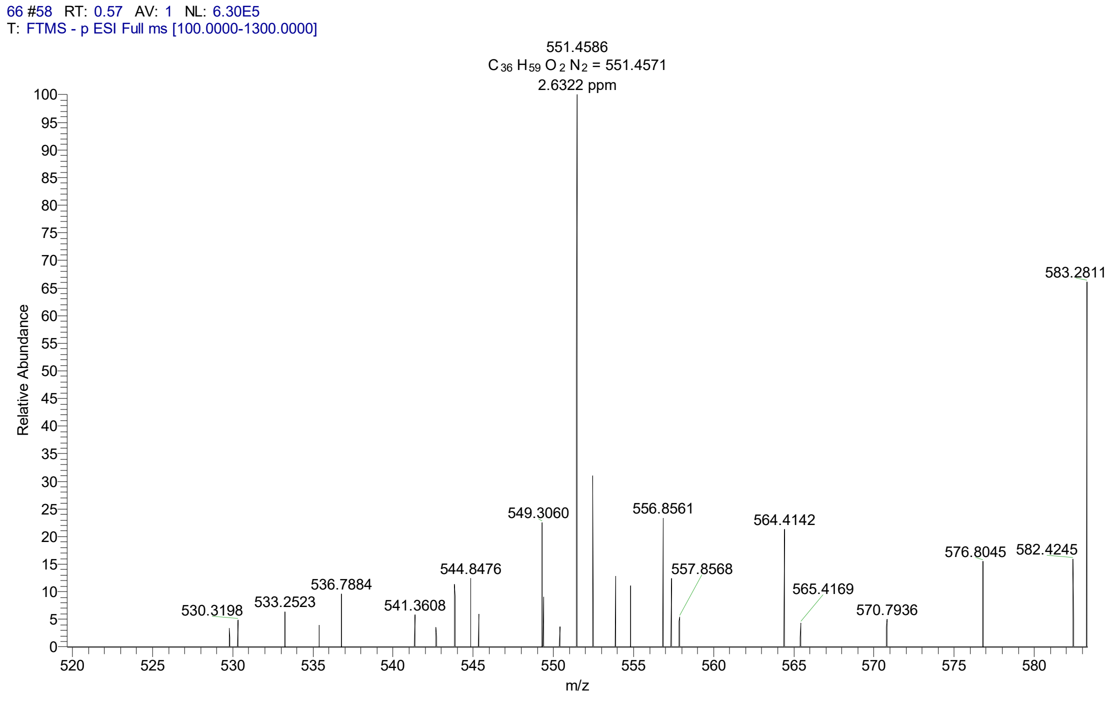

Figure S111. HRMS spectrum of target compound **B₉.**

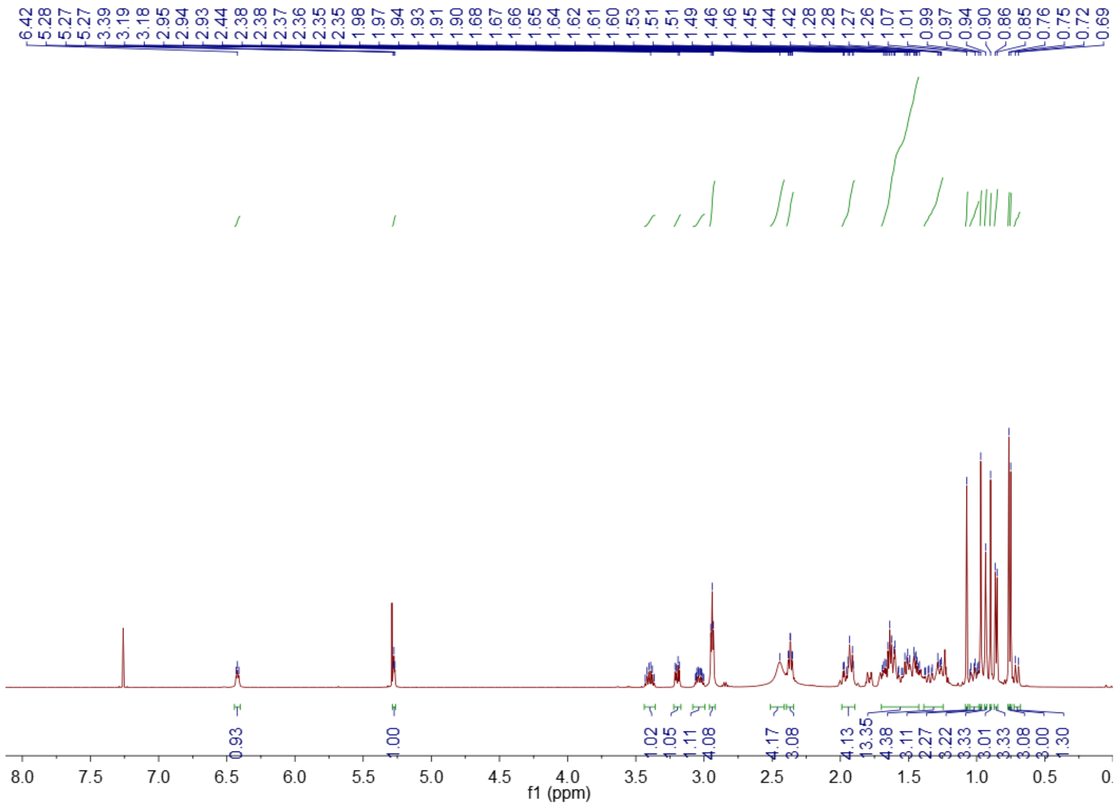

Figure S112. ^{1}H NMR spectrum ($CDCl_3$, 500 MHz) of target compound $\mathbf{B_{10}}$.

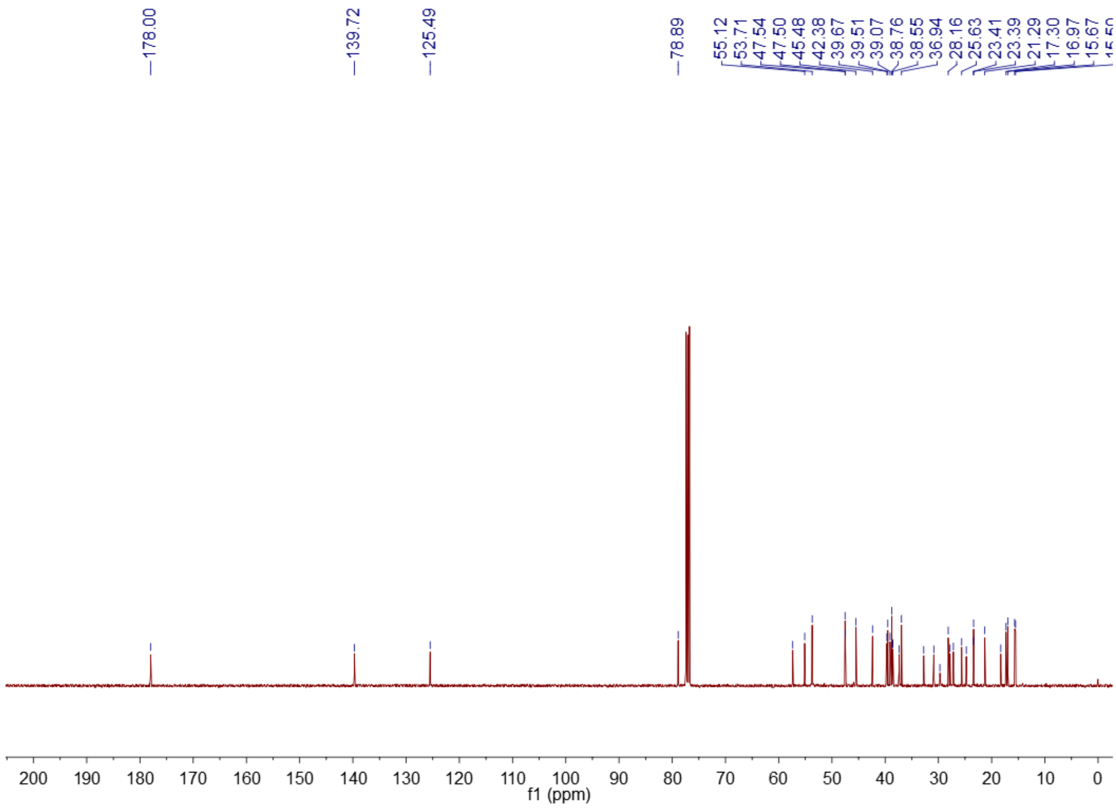

Figure S113. ^{13}C NMR spectrum ($CDCl_3$, 101 MHz) of target compound $\mathbf{B_{10}}$.

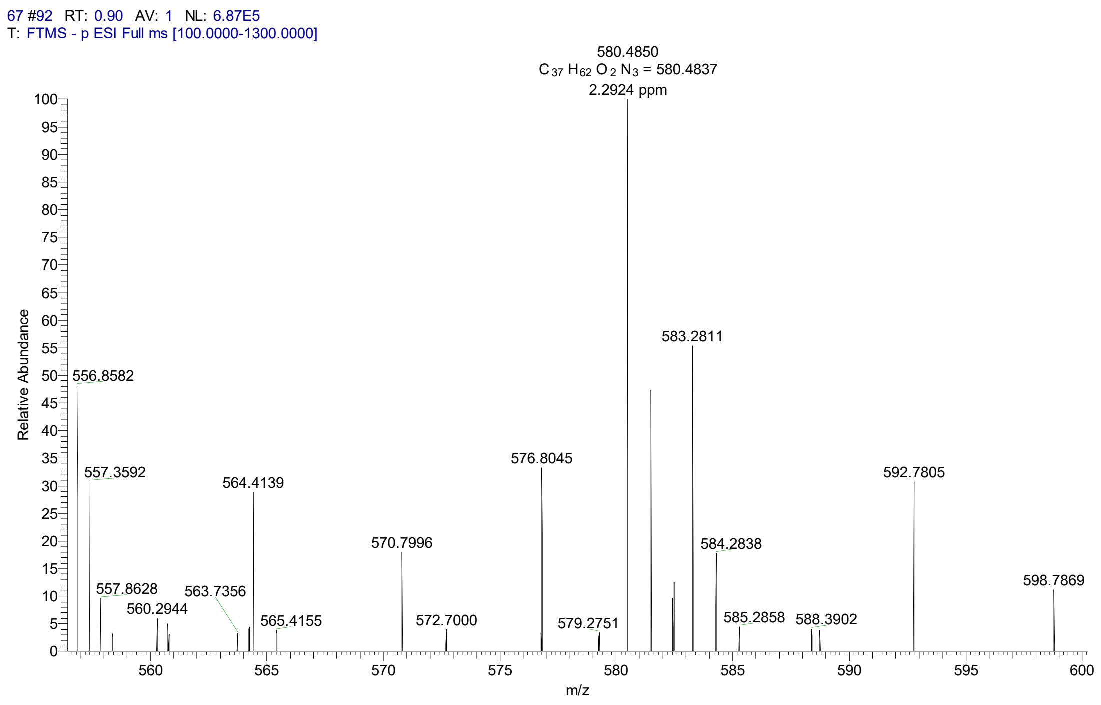

Figure S114. HRMS spectrum of target compound $\mathbf{B_{10}}$.

3. *In vivo* phytotoxicity of rice results (Figure S1).

The compound $\mathbf{A_{22}}$ on plants was non-toxic at doses of 200 mg L^{-1} and 500 mg L^{-1}.

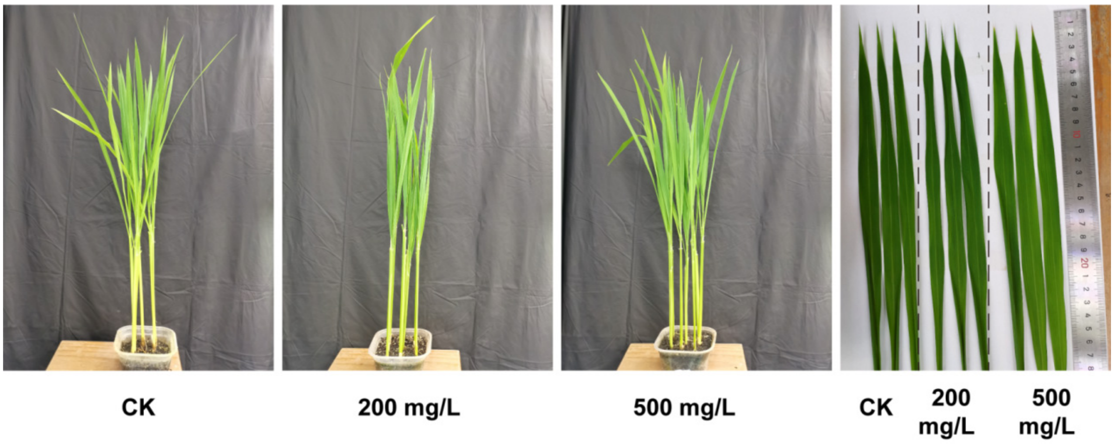

Figure S115. Toxicity of compound $\mathbf{A_{22}}$ to rice *in vivo* at 200 and 500 mg L^{-1}.
